# Use of Novel Strategies to Develop Guidelines for Management of Pyogenic Osteomyelitis in Adults: A WikiGuidelines Group Consensus Statement

**DOI:** 10.1001/jamanetworkopen.2022.11321

**Published:** 2022-05-10

**Authors:** Brad Spellberg, Gloria Aggrey, Meghan B. Brennan, Brent Footer, Graeme Forrest, Fergus Hamilton, Emi Minejima, Jessica Moore, Jaimo Ahn, Michael Angarone, Robert M. Centor, Kartikeya Cherabuddi, Jennifer Curran, Kusha Davar, Joshua Davis, Mei Qin Dong, Bassam Ghanem, Doug Hutcheon, Philipp Jent, Minji Kang, Rachael Lee, Emily G. McDonald, Andrew M. Morris, Rebecca Reece, Ilan S. Schwartz, Miranda So, Steven Tong, Christopher Tucker, Noah Wald-Dickler, Erica J. Weinstein, Riley Williams, Christina Yen, Shiwei Zhou, Todd C. Lee

**Affiliations:** 1 Los Angeles County+University of Southern California (USC) Medical Center, Los Angeles; 2 Montgomery Medical Associates PC, Rockville, Maryland; 3 University of Wisconsin Hospital and Clinics, William S. Middleton Memorial Veterans Hospital, Madison; 4 Providence Portland Medical Center, Portland, Oregon; 5 Rush University Medical Center, Chicago, Illinois; 6 North Bristol NHS Trust, Bristol, United Kingdom; 7 Department of Clinical Pharmacy, University of Southern California School of Pharmacy, Los Angeles; 8 Providence Little Company of Mary Medical Center, San Pedro, California; 9 Department of Orthopaedic Surgery, Michigan Medicine, University of Michigan, Ann Arbor; 10 Northwestern University Medical Center, Chicago, Illinois; 11 Department of Medicine, Birmingham Veterans Affairs (VA) Medical Center, Birmingham, Alabama; 12 Department of Medicine, University of Florida, Gainesville; 13 Division of Infectious Diseases, Department of Internal Medicine, Michigan Medicine, Ann Arbor; 14 Menzies School of Health Research and Charles Darwin University, Darwin, Australia; 15 New York Health and Hospitals Bellevue Hospital, New York, New York; 16 King Abdulaziz Medical City, Jeddah, Saudi Arabia; 17 Department of Infectious Diseases, Inselspital Bern University Hospital, Bern, Switzerland; 18 University of Texas Southwestern, Dallas; 19 Department of Medicine, Division of Infectious Diseases, University of Alabama at Birmingham, Birmingham; 20 Clinical Practice Assessment Unit, Department of Medicine, McGill University, Montreal, Canada; 21 Department of Medicine, Division of Infectious Diseases, Sinai Health, University Health Network, and University of Toronto, Toronto, Canada; 22 Section of Infectious Diseases, Department of Medicine, West Virginia University School of Medicine, Morgantown; 23 Division of Infectious Diseases, Department of Medicine, University of Alberta, Edmonton, Canada; 24 Sinai Health System-University Health Network Antimicrobial Stewardship Program, UHN and Leslie Dan Faculty of Pharmacy, University of Toronto, Toronto, Canada; 25 Victorian Infectious Diseases Service, Royal Melbourne Hospital and University of Melbourne, at the Peter Doherty Institute for Infection and Immunity, Melbourne, Australia; 26 Hospital Medicine, Magnolia Regional Health Center, Corinth, Mississippi; 27 Division of Infectious Diseases, Department of Medicine and Center for Clinical Epidemiology and Biostatistics, Department of Biostatistics, Epidemiology, and Informatics, Perelman School of Medicine, University of Pennsylvania, Philadelphia; 28 Pharmacy Service, Oklahoma City VA Health Care System, Oklahoma City, Oklahoma

## Abstract

**Importance:**

Traditional approaches to practice guidelines frequently result in dissociation between strength of recommendation and quality of evidence.

**Objective:**

To construct a clinical guideline for pyogenic osteomyelitis management, with a new standard of evidence to resolve the gap between strength of recommendation and quality of evidence, through the use of a novel open access approach utilizing social media tools.

**Evidence Review:**

This consensus statement and systematic review study used a novel approach from the WikiGuidelines Group, an open access collaborative research project, to construct clinical guidelines for pyogenic osteomyelitis. In June 2021 and February 2022, authors recruited via social media conducted multiple PubMed literature searches, including all years and languages, regarding osteomyelitis management; criteria for article quality and inclusion were specified in the group’s charter. The GRADE system for evaluating evidence was not used based on previously published concerns regarding the potential dissociation between strength of recommendation and quality of evidence. Instead, the charter required that clear recommendations be made only when reproducible, prospective, controlled studies provided hypothesis-confirming evidence. In the absence of such data, clinical reviews were drafted to discuss pros and cons of care choices. Both clear recommendations and clinical reviews were planned with the intention to be regularly updated as new data become available.

**Findings:**

Sixty-three participants with diverse expertise from 8 countries developed the group’s charter and its first guideline on pyogenic osteomyelitis. These participants included both nonacademic and academic physicians and pharmacists specializing in general internal medicine or hospital medicine, infectious diseases, orthopedic surgery, pharmacology, and medical microbiology. Of the 7 questions addressed in the guideline, 2 clear recommendations were offered for the use of oral antibiotic therapy and the duration of therapy. In addition, 5 clinical reviews were authored addressing diagnosis, approaches to osteomyelitis underlying a pressure ulcer, timing for the administration of empirical therapy, specific antimicrobial options (including empirical regimens, use of antimicrobials targeting resistant pathogens, the role of bone penetration, and the use of rifampin as adjunctive therapy), and the role of biomarkers and imaging to assess responses to therapy.

**Conclusions and Relevance:**

The WikiGuidelines approach offers a novel methodology for clinical guideline development that precludes recommendations based on low-quality data or opinion. The primary limitation is the need for more rigorous clinical investigations, enabling additional clear recommendations for clinical questions currently unresolved by high-quality data.

## Key Points

**Question:**

Can a novel methodology using collaborative research coordinated online be successfully applied to the development of a guideline for the diagnosis and treatment of a common infectious disease, pyogenic osteomyelitis?

**Findings:**

This consensus statement and systematic review using a novel WikiGuidelines methodology addresses 7 questions regarding the management of osteomyelitis, resulting in the establishment of 2 clear recommendations (concerning oral antibiotic therapy for pyogenic osteomyelitis and duration of therapy) and 5 clinical reviews that outline a present lack of adequate, hypothesis-confirming data.

**Meaning:**

These results suggest that this novel, egalitarian methodology enables a clear separation of established care standards based on hypothesis-confirming evidence from practice preferences that are based on lower quality or no evidence.

## Teaser

This consensus statement and systematic review from the WikiGuidelines Group, an online collaborative research initiative, examines standards of evidence for pyogenic osteomyelitis care and offers clinical recommendations.

## Introduction

An important limitation of traditional clinical guidelines is the frequent dissociation between quality of evidence and strength of recommendations.^1,2,3,4,5,6^ As a result, some past guideline recommendations have endorsed harmful care, which was only subsequently recognized when high-quality, prospective controlled trials were conducted.^7^ To overcome this limitation, we developed a novel approach, called WikiGuidelines, to establish clear recommendations only when high-quality, hypothesis-confirming evidence is available (see group charter in Supplement 1).

Our initial social media poll revealed a desire for renewed guidance on a common infectious disease, pyogenic osteomyelitis. Pyogenic osteomyelitis occurs at a rate of approximately 20 cases per 100 000 person-years, with rates rising among patients with diabetes and older patients, as well as those with prosthetic joints.^8,9,10^ In low- and lower-middle-income countries (LMIC), osteomyelitis may be more common in younger patients as a result of traumatic injury.^11^ Nevertheless, the global economic burden of osteomyelitis is considerable for high-income countries and LMIC.^9,10,12,13,14^

Osteomyelitis is an ancient disease, with the earliest documented case in an unfortunate, 250-million-year-old dimetredon with a fractured spinal shaft.^15^ In the modern era, radiography, surgical methods, and antibiotics have revolutionized its management. However, these successful interventions have resulted in long-standing diagnostic and therapeutic paradigms that have guided treatment despite lacking strong evidence, including the need for diagnostic x-rays and intravenous-only antibiotic therapy for all patients.^16^ Recent studies have begun to challenge these dogmas.^16,17,18^ This guideline focuses on data regarding management of pyogenic osteomyelitis in adults (see Supplement 2 for the complete guidelines).

## Methods

The WikiGuidelines Group formed on Twitter by participants who were dissatisfied with traditional guideline methodologies. The group constructed a charter that specifically chose not to use the GRADE system for evaluating strength of evidence based on previously published concerns regarding bias, poor interrater reliability, and, most importantly, the dissociation between strength of recommendation and quality of evidence (Supplement 1).^1,2,3,4,5,6,7^

Instead, the group sought to incorporate the “humility of uncertainty”^7^ by only providing clear recommendations when reproducible, high-quality, hypothesis-confirming evidence is available, requiring at a minimum: (1) 1 properly conducted, adequately powered randomized controlled trial (RCT); and (2) at least 1 other concordant, prospective, controlled clinical study—either a second RCT, a quasi-experimental pre-post study, a pragmatic nonrandomized trial, or a carefully conducted historically controlled study. In the absence of such data, the charter requires provision of clinical reviews that discuss care choices. However, recognizing the core ethical and clinical principle of “first do no harm,” authors could recommend against the routine provision of unsubstantiated care as part of clinical reviews. We also sought to incorporate principles of high value care (ie, right care, right place, right cost) and health care quality (ie, safe, effective, patient-centered, timely, efficient, equitable).^19^

Drafting members participated in reviews for 7 questions regarding the diagnosis and management of pyogenic osteomyelitis. For each question, members conducted their own literature review using PubMed, including all years and languages, with key words that varied by the question being asked. Articles were assessed for quality and inclusion by criteria specified in the charter. References from identified articles were also searched for potential inclusion. When divergent opinions on article interpretation or clinical practice existed among the authors, we did not attempt to force consensus; rather, in accord with the charter, we sought to transparently highlight those diverging opinions by discussing care alternatives. For answers based on more than 1 relevant RCT, meta-analysis was conducted using Review Manager 5.4.1 (Cochrane Collaboration).

## Results

The consortium that established the WikiGuidelines Charter consisted of 63 participants from 8 countries: Australia, Canada, Colombia, Saudi Arabia, Spain, Switzerland, the United Kingdom, and the US. These participants included physicians, pharmacists, and microbiologists with expertise in general internal and hospital medicine, pediatrics, infectious diseases, orthopedic surgery, pharmacology, and medical microbiology.

The participants addressed 7 questions regarding the diagnosis and management of pyogenic osteomyelitis but found data sufficient to establish clear recommendations for only 2: oral antibiotic therapy for pyogenic osteomyelitis and duration of therapy. In contrast, 5 questions were addressed with clinical reviews in the absence of high-quality data: diagnosis of pyogenic osteomyelitis, management of osteomyelitis underlying pressure ulcers, appropriate timing of empirical therapy, rational selection of antimicrobial options, and use of serial biomarkers or imaging studies to evaluate therapeutic response.

### Question 1: How Should the Diagnosis of Osteomyelitis Be Established?

#### Clinical Review (Insufficient Quality of Evidence to Enable a Clear Recommendation)

##### Osteomyelitis Without Prosthetic Joint Infections (PJI)

Based on observational studies, we do not recommend the routine use of plain x-rays (because of inadequate sensitivity, specificity) or computed tomography scans (inadequate sensitivity) for all patients with a possible diagnosis of osteomyelitis (Table 1; eTable 1 in Supplement 2) as they may result in unnecessary radiation and use of resources. However, these studies may be helpful if a fracture or other noninfectious cause of bone pain (eg, tumor, foreign object) is prioritized on the differential diagnosis, and/or the pretest probability of osteomyelitis is lower (eg, ≤15%). Magnetic resonance imaging (MRI) and certain tagged white cell scans are the most accurate imaging modalities for diagnosing osteomyelitis. Inflammatory biomarkers are insufficiently accurate, and we do not recommend their routine use for osteomyelitis diagnosis. Blood cultures have variable sensitivity, but if the patient has systemic symptoms or risk factors for bacteremia (eg, intravenous drug use), isolating likely pathogens (eg, *Staphylococcus aureus*) can be helpful to target with therapy and potentially obviate the need for bone biopsy. If available, bone biopsy for histopathology is highly accurate if positive, but cannot rule out osteomyelitis if negative. Culture of biopsy specimens of the affected bone may help identify etiology and target antimicrobial therapy.

**Table 1.** Pooled Point Estimates of Sensitivity, Specificity, and Likelihood Ratios for Diagnostic Tests for Osteomyelitis

Test	Sensitivity, %	Specificity, %	Positive LR^a^	Negative LR^a^	Reference
Osteomyelitis without PJI					
X-rays	70	82	3.9	0.4	Llewellyn et al,^45^ 2019
CT scans	70	90	7.0	0.3	Llewellyn et al,^45^ 2019
MRI	96	81	5.1	0.05	Llewellyn et al,^45^ 2019
Nuclear medicine scintigraphy^b^	84	71	2.9	0.2	Llewellyn et al,^45^ 2019
White cell tagged scans	87	95	17.4	0.1	Llewellyn et al,^45^ 2019
PET	85	93	12.1	0.2	Llewellyn et al,^45^ 2019
SPECT	95	82	5.3	0.06	Llewellyn et al,^45^ 2019
ESR	49-79	50-80	1.6-3.8	0.3-0.4	Ryan et al,^46^ 2019; Ghassibi et al,^47^ 2021; Wu et al,^48^ 2020
CRP	45-76	59-71	1.1-2.6	0.3-0.8	Ryan et al,^46^ 2019; Ghassibi et al,^47^ 2021; Wu et al,^48^ 2020
Biopsy (histopathology)	52	>99	>50	0.5	Pupaibool et al,^49^ 2015
DFO					
X-rays	62	78	2.8	0.5	Llewellyn et al,^50^ 2020
MRI	93-96	75-84	3.7-6.0	0.05-0.09	Llewellyn et al,^50^ 2020; Lauri et al,^51^ 2017
Nuclear medicine scintigraphy^b^	85	68	2.7	0.2	Llewellyn et al,^50^ 2020
White cell tagged scans	91-92	75-92	3.6-11.5	0.09-0.1	Lauri et al,^51^ 2017
PET	84	93	12.0	0.2	Llewellyn et al,^50^ 2020
ESR	60-81	56-90	1.4-8	0.2-0.7	Xu et al,^52^ 2020; Moallemi et al,^53^ 2020; Lavery et al,^54^ 2019; Victoria van Asten et al,^55^ 2016
CRP	49-76	55-80	1.1-3.8	0.3-0.9	Xu et al,^52^ 2020; Moallemi et al,^53^ 2020; Lavery et al,^54^ 2019; Markanday,^56^ 2015
Probe-to-bone	87	83	5.1	0.2	Lam et al,^57^ 2015
PJI^c^					
X-rays	14	70	0.5	1.2	Sconfienza et al,^58^ 2019
MRI	65-94	73-99	2.4->50	0.06-0.5	Sconfienza et al,^58^ 2019; Galley et al,^59^ 2020; Schwaiger et al^60^ 2020
Nuclear medicine scintigraphy^b^	83-94	69-90	2.7-9.4	0.07-0.2	Ikeuchi et al,^61^ 2013; Nagoya et al,^62^ 2008; Ouyang et al,^63^ 2014
White cell tagged scans	93-100	91-100	10->50	0.08-<0.01	Erba et al,^64^ 2014; Teiler et al,^65^ 2020
PET	82-95	39-87	1.3-7.3	0.06-0.5	Kiran et al,^66^ 2019; Kwee et al,^67^ 2008; Jin et al,^68^ 2014
ESR	75	70-87	2.5-5.8	0.3-0.4	Berbari et al,^69^ 2010; Pérez-Prieto et al,^70^ 2017
CRP	88-97	74	3.4-3.7	0.04-0.2	Berbari et al,^69^ 2010; Pérez-Prieto et al,^70^ 2017
IL-6	97	91	10.8	0.03	Berbari et al,^69^ 2010
Synovial WBC count	88	93	12.6	0.1	Qu et al,^71^ 2014
Synovial PMN %	90	88	7.5	0.1	Qu et al,^71^ 2014
Synovial culture	62	94	10.3	0.4	Lee et al,^72^ 2017

Abbreviations: CRP, C-reactive protein rate; CT, computerized tomography; DFO, diabetic foot osteomyelitis; ESR, erythrocyte sedimentation rate; IL-6, interleukin-6; LR, likelihood ratio; MRI, magnetic resonance imaging; PET, positron emission tomography; PJI, prosthetic joint infection; PMN, polymorphonuclear; SPECT, single photon emission computed tomography; WBC, white blood cell.

^a^ A positive LR ≥5 is helpful and ≥10 is very helpful at shifting posttest probabilities; a negative LR ≤0.2 is helpful and ≤0.1 is very helpful at shifting posttest probabilities.

^b^ Excluding tagged white cell studies, which are considered separately.

^c^ Because there is no identified optimal referent standard for the diagnosis of PJI, sensitivity, specificity, and LRs for tests for PJI should be considered to be uncertain estimates.

##### Diabetic Foot Osteomyelitis (DFO)

Based on observational studies, plain x-rays have low sensitivity and specificity for diagnosing DFO (Table 1; eTable 1 in Supplement 2). The probe-to-bone (PTB) test is simple, noninvasive, and has reasonable sensitivity and specificity as a diagnostic method for DFO, which may preclude the need for imaging in some settings. MRI and certain tagged white cell scans are the most accurate imaging modalities for diagnosing DFO, although their specificities are lower than their sensitivities. Inflammatory biomarkers are insufficiently accurate and we do not recommend their routine use for diagnosis. If available, percutaneous bone biopsy for deep microbiological cultures may help target antimicrobial therapy; surface cultures are not accurate and not recommended.

##### Osteomyelitis With PJI

There is no established, accurate referent standard diagnostic test for PJI. Certain tagged white cell scans are the most accurate imaging studies for PJI (Table 1; eTable 1 in Supplement 2); however, given the limitations of individual tests, published algorithms are sometimes recommended to establish the diagnosis. Data are limited and inadequate to compare the relative accuracies of competing algorithms. Practically, the diagnosis is typically made from a combination of history, physical examination, imaging studies to assess alternate causes of pain and instability, inflammatory markers, synovial fluid analysis, and/or operative specimens. Molecular diagnostic testing is a promising approach, but data are mixed and inadequate to recommend for or against its use as of 2022.

### Question 2: What Is the Appropriate Management for Osteomyelitis Underlying a Pressure Ulcer?

#### Clinical Review (Insufficient Quality of Evidence to Enable a Clear Recommendation)

Observational studies indicate that imaging and inflammatory biomarkers are not diagnostically accurate for osteomyelitis underlying a pressure ulcer and we do not recommend their routine use for this purpose. Antibiotics have not been shown to be of benefit (and may be of harm) in the absence of surgical wound closure, but osteomyelitis may increase the risk of surgical flap failure.^20,21^ Therefore, it may be preferable to avoid the routine use of antibiotic therapy for osteomyelitis underlying a pressure ulcer unless deep bone biopsy confirms osteomyelitis and surgical wound closure is planned, or the patient has accompanying sepsis syndrome or local soft tissue infection. Irrespective of antibiotic use, a multimodal therapeutic approach includes nutritional optimization, wound debridement and care, pressure off-loading, and psychosocial management.

### Question 3: When Should Empirical Therapy Be Administered in the Treatment of Osteomyelitis?

#### Clinical Review (Insufficient Quality of Evidence to Enable a Clear Recommendation)

Some observational studies suggest that administration of antibiotics prior to bone biopsy or surgical management may modestly decrease yield of bone cultures for patients with osteomyelitis, including DFO and PJI. Thus, presuming other microbiological methods (eg, blood cultures) have not already established a microbial etiology, it is reasonable to consider deferring antimicrobial therapy initiation until bone and/or joint microbiological samples are obtained for clinically stable patients. However, other studies are not concordant, and histopathology results are unlikely to be affected by prior short-term antibiotics. Decisions regarding the delay of empirical therapy therefore balance potential harm due to the risk of progression of life-threatening infection (eg, sepsis) or impending spinal cord compression against the potential benefit of microbiological data.

### Question 4: Are There Preferred Antibiotics With Which to Treat Osteomyelitis?

#### Clinical Review (Insufficient Quality of Evidence to Enable a Clear Recommendation)

##### Which Empirical Antimicrobial Agents Are Preferred for Osteomyelitis?

Based on data from observational studies, if antibiotic therapy cannot be delayed until culture availability, it is reasonable to empirically cover aerobic gram-positive cocci, especially *S aureus,* and gram-negative bacilli (Table 2; eTable 2 in Supplement 2). Many practitioners routinely provide anaerobic coverage for DFO; however, comparative data are not available to establish the clinical benefit or harm of this approach. Inclusion of empirical therapy targeting methicillin-resistant *S aureus* (MRSA) or *Pseudomonas aeruginosa* depends on the presence of specific risk factors (addressed below). In all cases, local susceptibility patterns, patient-specific risk factors, and prior culture data influence the choice of antibiotic selection. Culture results can be used to tailor empirical therapy when possible.

**Table 2.** Reasonable Empirical Antimicrobial Therapy Options With Published Data^a^

Types of osteomyelitis	Empirical IV antibiotics^b^	Alternative empirical IV antibiotics	Empirical oral antibiotics^c^
Osteomyelitis without a retained implant	Ceftriaxone ± vancomycin	Alternative to β lactam: fluoroquinolone Alternative to vancomycin: linezolid, daptomycin, or clindamycin	TMP-SMX Clindamycin^d^ Linezolid^d^ Fluoroquinolone Doxycycline^e^ ± rifampin
Diabetic foot osteomyelitis	Ampicillin-sulbactam Amoxicillin-clavulanate Ceftriaxone^f^ ± metronidazole ± vancomycin	Alternative to β lactam: fluoroquinolone^f^ ± metronidazole Alternative to vancomycin: linezolid, daptomycin, or clindamycin	Amoxicillin-clavulanate TMP-SMX Clindamycin^d^ Linezolid^d^ Fluoroquinolone or doxycycline^e^ ± metronidazole^f^ ± rifampin
Osteomyelitis with a retained implant (including PJI)			
<3 mos since procedure (early)	Antipseudomonal β lactam or ceftriaxone + vancomycin^g^	Alternative to β lactam: fluoroquinolone Alternative to vancomycin: linezolid, daptomycin, or clindamycin	Fluoroquinolone ± rifampin If gram-positive confirmed: TMP-SMX or clindamycin^d^ or linezolid^d^ or doxycycline^e^ ± rifampin
≥3 mos after procedure (later onset)	Ceftriaxone + vancomycin^g^	Alternative to β lactam: fluoroquinolone Alternative to vancomycin: linezolid, daptomycin, or clindamycin	TMP-SMX Clindamycin^d^ Linezolid^d^ Fluoroquinolone Doxycycline^e^ ± rifampin

Abbreviations: PJI, prosthetic joint infection; TMP-SMX, trimethoprim-sulfamethoxazole.

^a^ This table addresses reasonable therapies with published data to be administered in the absence of available Gram stain, culture, histopathology, or other guiding information that enable targeted therapy. Biopsies should be obtained for such information prior to initiation of therapy when the risk-benefit ratio is favorable. See question 3 in the Results for a thorough discussion of initiation of empirical therapy vs waiting for biopsy information to target therapy. In all cases, antibiotic selection should be adjusted based on local sensitivities for likely target pathogens. This table is not meant to indicate that other therapeutic options cannot be considered for specific patients based on clinical circumstances.

^b^ Add empirical anti–methicillin-resistant *S aureus* (MRSA) coverage (eg, vancomycin) and/or replace ceftriaxone with an antipseudomonal β lactam (eg, cefepime, piperacillin-tazobactam) if specific risk factors for MRSA (eg, colonization, prior MRSA infection, health care exposure with endemic MRSA) and/or *P aeruginosa* (exposed to prior courses of antibiotics, prior cultures with *P aeruginosa,* gangrenous wounds, recent surgical procedures, specific sites of infection such as malignant otitis externa) are present, respectively (see question 4 in the Results). When such risk factors are present, the authors unanimously preferred the use of noncarbapenem anti-pseudomonal options for stewardship reasons, unless there is a specific concern for extended-spectrum β-lactamase pathogens. Similarly, anti-anaerobic coverage is not routinely needed, but if the wound is gangrenous or there is specific concern for anaerobic infection, metronidazole may be added, or ceftriaxone replaced with ampicillin-sulbactam or amoxicillin-clavulanate. Finally, for patients in whom a MRSA active agent is deemed unnecessary, some authors preferred to add an anti-staphyloccocal β-lactam (eg, oxacillin, cloxacillin, nafcillin, cefazolin) to ceftriaxone.

^c^ See question 5 in the Results for full discussion of oral therapy, including selection of agents and timing of initiation. Rifampin may be important to add to fluoroquinolones when treating *S aureus* infections, and possibly when treating *Pseudomonas* or *Acinetobacter* infections, to reduce emergence of resistance. Other uses of rifampin are discussed in question 4 of the Results.

^d^ As clindamycin and linezolid have no reliable gram-negative coverage, they should only be used when the clinician is confident that the infection is not likely caused by a gram-negative pathogen; if there are concerns that gram-negative pathogens may be causing the infection, they should be administered with the addition of a second agent that covers gram-negative pathogens.

^e^ There are less published data for doxycycline; however, it has been used with anecdotal success and was used in a minority of patients in the OVIVA trial,^10^ so it may be an alternative agent in individual patients.

^f^ Anaerobic coverage is routinely added by many practitioners; however, data are not available to demonstrate whether it adds clinical benefit or not.

^g^ While many authors would initiate empirical anti-pseudomonal therapy, some authors do not believe that anti-pseudomonal coverage is routinely needed for early PJI infection based on the frequency with which the organism is locally encountered. Most authors who would initiate rifampin preferred to wait until oral transition, but some authors would consider initiating empirical IV rifampin. If rifampin use is being considered, it may be prudent to wait until bacteremia is cleared (if present) and surgical source control is achieved (if necessary), to reduce the risk of treatment failure.^73^ See question 4 in the Results for a discussion of empirical pseudomonal therapy and of the potential benefits and/or risks of adjunctive rifampin therapy.

##### When Should Antimicrobial Coverage Targeting MRSA Be Included?

Based on culture data from observational studies, inclusion of empirical anti-MRSA coverage depends on local prevalence and patient-specific risk factors, such as known colonization status (which is the biggest individual risk factor), prior positive cultures, and health care exposure. In a setting with low MRSA incidence, no known MRSA colonization or prior positive cultures, and minimal health care contact, it is reasonable to withhold empirical MRSA coverage.

##### When Should Antimicrobial Coverage Against *P Aeruginosa* Be Included?

Based on culture data from observational studies, routine use of empirical antipseudomonal therapy for osteomyelitis is unnecessary. Such agents are added in the presence of specific risk factors, including patients with chronic wounds who have: (1) been exposed to multiple prior courses of antibiotics; (2) previously had cultures positive for *P aeruginosa*; (3) gangrenous wounds; (4) had a recent surgical procedure (eg, within 3 months, as with early PJI); or (5) specific sites of infection particularly associated with pseudomonal infection (eg, malignant otitis externa).

##### Does Bone Penetration of an Antimicrobial Agent Matter Clinically, and Should It Be Used to Select Therapy?

Outcome data related to antibiotic bone penetration are limited for osteomyelitis. Thus, theoretical bone penetration (eTable 3 in Supplement 2) is not the primary driver of antibiotic selection; published clinical outcomes data are more relevant.

##### Does Adjunctive Rifampin Alter Osteomyelitis Treatment Outcomes; for Which Organisms Is Rifampin Therapy Potentially Useful, and If It Is Used, Is There a Preferred Dosing?

Some observational studies and small RCTs suggest that addition of rifampin to standard therapy may improve long-term outcomes by reducing relapse of osteomyelitis, with or without retained implants or hardware. However, other observational studies and 1 small RCT are contrary. Overall, the data are mixed and remain uncertain (eFigures 1 and 2 in Supplement 2). The use of rifampin in this setting is based on culture results (principally targeting gram-positive cocci or nonfermenting gram-negative bacilli) and individual patient risk-benefit considerations, acknowledging the uncertainty of the efficacy data, side effects, and potential drug interactions (especially those disrupting stable, chronic medications, such as oral anticoagulants or opiates). Studies have not elucidated optimal total daily dosing, except that 450 to 600 mg per dose likely increases pharmacodynamic target attainment and adherence compared with 300 mg multiple daily dosing.^22,23,24,25,26^

##### What Is the Role of Long-Acting Glycopeptide Antibiotics in Treating Osteomyelitis?

One RCT and several small, largely single-center, observational studies have examined the role of 2 long-acting glycopeptides, dalbavancin and oritavancin, for the treatment of osteomyelitis.^27,28^ In these studies, the long-acting agents performed similarly to comparator regimens. There are no data supporting their superiority, so the use of these agents is based on risk-benefit considerations, as well as cost and complexity vs other regimens for individual patients and health system contexts.

### Question 5: Is Oral Therapy Appropriate for the Treatment of Osteomyelitis, and If So, What Are Reasonable Patient Selection Criteria for Administration?

#### Clear Recommendation

Based on 8 concordant RCTs comparing intravenous (IV) to oral therapy^17,29,30,31,32,33,34,35^ (Figure; eFigure 3 in Supplement 2) and 9 RCTs in which oral therapy was predominantly used in both arms,^36,37,38,39,40,41,42,43,44^ we recommend oral antibiotic therapy with a drug and/or dose used in published studies as a reasonable option for osteomyelitis of any type (ie, hematogenous, prosthetic, and contiguous, the latter including vertebral and DFO) for patients who: (1) are clinically stable (hemodynamically and at the site of infection, eg, no spinal instability); (2) have adequate source control (ie, not requiring further procedural drainage and without persistent bacteremia); (3) are likely to absorb oral medications from a functioning gastrointestinal tract; (4) have an available regimen used in published osteomyelitis studies to cover likely target pathogens; and (5) have no psychosocial reasons that preclude the safe use of oral therapy. There is no required minimum duration of IV lead-in; patients may be switched to oral therapy when all the above criteria are met, even at the empirical therapy stage. Specific drug options and doses are discussed in the detailed review section (Table 3; eTables 4 and 5 in Supplement 2).

**Figure.**
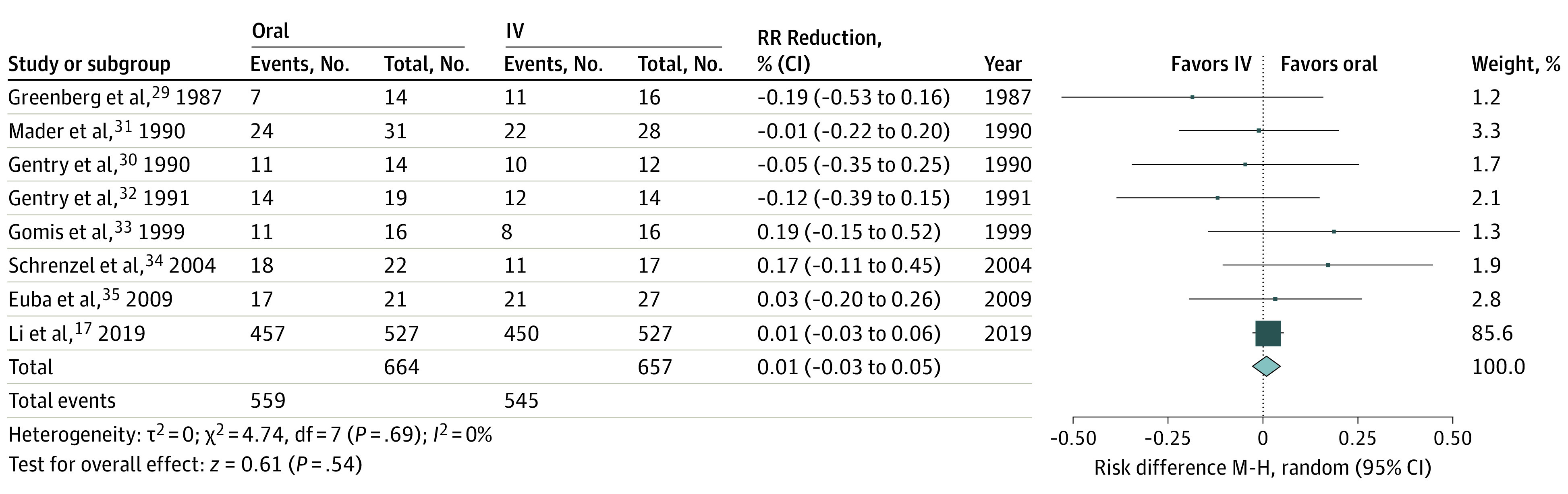
Random-Effects Meta-analysis Forest Plot of Randomized Clinical Trials Comparing Long-term Clinical Success Rates of Oral vs Intravenous (IV) Antibiotic Therapy for Osteomyelitis in Adults Reproduced with permission from the *American Journal of Medicine*.^18^

**Table 3.** Summary of Oral Antibiotic Doses Used in Published Studies for Osteomyelitis

Drug	Dose	Comments
Ciprofloxacin	500-750 mg twice daily	Higher dose for pseudomonas
Levofloxacin	750 mg once daily	L-enantiomer of ofloxacin, the latter of which was widely studied for osteomyelitis
TMP-SMX	7.5-10 trimethoprim mg/kg/d divided twice or thrice daily (eg, 2 DS tablets twice daily for a 70 kg adult)	Most studies used 7.5-10 mg/kg/d, 2 studies used 4-6 mg/kg/d, with lower cure rates in 1 of them
Clindamycin	600 mg 3 times/d; 900 mg 3 times/d or 600 mg 4 times/d for larger patients	450 mg 4 times/d may be used but was not favored in published studies
Linezolid	600 mg twice daily	Standard dosing, monitor for reversible hematotoxicity after 2 weeks, and irreversible neurotoxicity after 4 wks
Amoxicillin/ clavulanate	500 mg 3 times/d or 875 mg twice daily	Specifically for DFO
Rifampin	600 mg once daily	Doses studied include 600 once daily, 900 mg once daily or 600 mg twice daily, unclear if efficacy or toxicity differs; 300 mg doses may be less desirable due to lower AUC levels and less convenience for patients
Fosfomycin^a^	4-16 g per day	Various doses studied with formulations available outside the US, not studied with the sachet powder formulation in the US

Abbreviations: DFO, diabetic foot osteomyelitis; TMP-SMX, trimethoprim-sulfamethoxazole.

^a^ There are no published data for the treatment of osteomyelitis with the sachet powder oral formulation of fosfomycin available in the US.

### Question 6: What Is the Role and Optimal Utilization of Serial Biomarkers and/or Imaging Studies for Assessing Treatment Response in Osteomyelitis?

#### Clinical Review (Insufficient Quality of Evidence to Enable a Clear Recommendation)

In the absence of RCTs, observational studies have generally found that neither serial inflammatory biomarkers (eg, erythrocyte sedimentation rate, C-reactive protein) nor routinely repeated imaging accurately predict long-term treatment success for osteomyelitis or PJI for individual patients, nor have they been shown to meaningfully alter treatment decisions beyond clinical observation. Thus, following inflammatory biomarkers and repeated imaging may not offer benefit or contribute to high value care in most patients. Nonetheless, repeated imaging may be useful for patients who are clinically failing therapy to inform source control attempts, identify mechanical complications such as pathological fracture, and/or to trigger reconsideration of the initial diagnosis.

### Question 7: What Is the Appropriate Duration of Therapy for Typical Cases of Osteomyelitis?

#### Clear Recommendation

##### Osteomyelitis (Including DFO) Without a Retained Implant

Based on 2 RCTs (eFigure 4 in Supplement 2)^39,44^ and concordant observational studies, we recommend a maximum of 6 weeks of antibiotic therapy for hematogenous or contiguous pyogenic osteomyelitis (including DFO), assuming adequate source control (ie, no undrained abscesses too large to be treated with antibiotics alone, possibly ≥2-3 cm in diameter) and no retained prosthetic implant (Table 4; eTable 6 in Supplement 2). Insufficient data are available to establish a clear recommendation for durations shorter than 6 weeks (see clinical review below).

**Table 4.** Summary of Antibiotic Durations for Osteomyelitis

Condition	Clear recommendation	Clinical review
Osteomyelitis without retained implant (including DFO)	Maximum 6 wks	3-4 wks may be adequate with debridement; confirmatory studies desired
Osteomyelitis with total resection of infected bone	None	No antibiotics is a reasonable option; not recommended for use exceeding 5 d
PJI with DAIR	None	All participating experts preferred 12 wks; a confirmatory, second study is needed to enable a clear recommendation
PJI with exchange	None	12 wks favored by some experts Other experts believed equipoise remains for 6 vs 12 wks 6 wks may be reasonable for non–*S aureus* pathogens, particularly for 1-stage exchanges 6 wks may be reasonable for 2-stage exchange, although there is controversy about the need for further antibiotics after the second stage (reimplantation)

Abbreviations: DAIR, debridement, antibiotics, and implant retention; DFO, diabetic foot osteomyelitis; PJI, prosthetic joint infection.

#### Clinical Review (Insufficient Quality of Evidence to Enable a Clear Recommendation)

##### Osteomyelitis (Including DFO) Without a Retained Implant

Based on small RCTs, 3 or 4 weeks may be a reasonable duration of antibiotics for debrided osteomyelitis, whether hematogenous or contiguous (including DFO); however, confirmatory data are desired. Based on observational studies and 1 small RCT, it is reasonable to refrain from antibiotic use after total resection of infected bone if the treating physicians are confident that all infected bone has been resected. If administered, we do not recommend exceeding 2 to 5 days of therapy if there is no complicating soft tissue infection.

##### Osteomyelitis With a Retained Implant (Including PJI)

Based on the Duration of Antibiotic Treatment in Prosthetic Osteo-articular infection (DATIPO) RCT,^43^ participating experts unanimously agree that 12 is preferred to 6 weeks of antibiotics for PJI treated with debridement, antibiotics, and implant retention (DAIR). Some experts also clearly preferred 12 weeks of antibiotics for PJI treated with prosthetic exchanges. However, others believed that equipoise remains between 6 vs 12 weeks for these patients, particularly if *S aureus* is not the etiologic pathogen, or for 1-stage exchanges or 2-stage revisions with negative cultures prior to implantation.

Duration of therapy for other infected implants is not clear. A reasonable strategy, without evidence for or against, may be to treat with antibiotics until the bone heals sufficiently enough that the implants can be removed, such as in cases of fracture. Finally, chronic oral suppressive therapy may be considered for patients for whom the risks and benefits of curative surgery is deemed unacceptable; however, available data have not defined the risks and benefits of this approach well to this point.

## Discussion

Based on the results of recent studies, the current approach to pyogenic osteomyelitis and PJI management can increasingly incorporate newer diagnostic and therapeutic concepts. Such changes include recognizing the low value and high cost and burden that plain x-rays incur if routinely ordered for all patients with possible osteomyelitis, reducing or eliminating the routine ordering of low-value, low-accuracy blood biomarkers (eg, inflammatory markers), increasing adoption of oral step-down therapy, and limiting the duration of therapy to the shortest established to be necessary for optimizing cure in RCTs (eg, not more than 6 weeks for osteomyelitis without a prosthetic implant, 12 weeks for PJI treated with DAIR). These changes incorporate considerations of high value care and implementation in LMIC and resource-constrained settings, and thus are applicable across diverse care environments.

### Limitations

The main limitation of this study was that the establishment of only 2 clear recommendations highlights the need for additional high-quality studies of osteomyelitis. In particular, studies are needed regarding new approaches to diagnostics; to elucidate the comparative effectiveness of various antimicrobial options, including adjunctive rifampin or anaerobic therapy; to identify which patients are more likely to relapse after completion of therapy; to further clarify antibiotic durations of therapy; to define the role and optimal methodologies of surgical management; and to define the role of nonantimicrobial adjunctive strategies (eg, hyperbaric oxygen therapy). We also seek to incorporate authors from LMIC countries in future revisions to ensure the WikiGuidelines are broadly applicable to these settings.

## Conclusions

WikiGuidelines represent a novel approach to guideline construction, clearly delineating evidenced-based recommendations from opinions based on lower-quality data. Resulting changes in management of pyogenic osteomyelitis include recognizing the low value and high burden that plain x-rays incur if routinely ordered for all patients, reducing the routine ordering of low value, low accuracy blood biomarkers, increasing adoption of oral therapy, and limiting the duration of therapy to the shortest necessary for optimizing cure.

These guidelines are based on published data available as of March 1, 2022. Clinicians who believe other evidence should be considered may contact any of the authors to initiate possible revisions, which the authors intend to complete in close to real-time. The authors understand that no clinical trial can extrapolate to all possible patient care scenarios. Thus, we expect that these guidelines should not establish medicolegal standards of care or replace clinician judgment for individual patients.

## Notes

Supplement 1.
WikiGuidelines Charter

Supplement 2. **eAppendix.** WikiGuidelines Overview, Executive Summary, and Discussion **eFigure 1.** Forest Plot of RCTs Comparing Success Rates of Patients Treated With Adjunctive Rifampin or Not for S. Aureus Osteomyelitis, With or Without Prosthetic Implants **eFigure 2.** Forest Plots of Subsets of RCTs Comparing Rifampin vs No Rifampin for Only Osteomyelitis Without PJI or Only Osteomyelitis With PJI **eFigure 3.** Random Effects Meta-analysis Forest Plot of RCTs Comparing Long-term Clinical Success Rates of Oral vs IV Antibiotic Therapy for Osteomyelitis in Adults **eFigure 4.** Random Effects Forest Plot of RCTs Comparing Shorter vs Longer Courses of Antibiotic Therapy for Vertebral Osteomyelitis and DFO in Adults **eTable 1.** Pooled Point Estimates of Sensitivity, Specificity, and Likelihood Ratios for Diagnostic Tests for Osteomyelitis **eTable 2.** Reasonable Empiric Antimicrobial Therapy Options with Published Data **eTable 3.** Antibiotic Concentrations in Bone **eTable 4.** Treatment Success Rates in Observational Studies of Oral Treatment of Osteomyelitis With or Without Infected Prosthesis in Adults **eTable 5.** Summary of Oral Antibiotic Doses Used in Published Studies for Osteomyelitis **eTable 6.** Summary of Antibiotic Durations for Osteomyelitis

Supplement 3.
Nonauthor Collaborators
