# Supplementary material for: Use of Novel Strategies to Develop Guidelines for Management of Pyogenic Osteomyelitis in Adults: A WikiGuidelines Group Consensus Statement
Source: JAMA Netw Open. 2022 May 10;5(5):e2211321. doi: 10.1001/jamanetworkopen.2022.11321 (PMC9092201; doi:10.1001/jamanetworkopen.2022.11321)
Supplement: Supplement 1. — WikiGuidelines Charter [file jamanetwopen-e2211321-s001.pdf]

Spellberg B, Aggrey G, Brennan MB, et al. Use of novel strategies to develop guidelines for management of pyogenic osteomyelitis in adults:a WikiGuidelines group consensus statement. *JAMA Netw Open*. 2022;5(5):e2211321. doi:10.1001/jamanetworkopen.2022.11321

WikiGuidelines Charter

4-29-21

I. Guidelines Principles

A. Perspective

1. WikiGuidelines seek to provide clinical practice guidelines with a pragmatic focus that are broadly applicable to "real world," practical settings, including outside of academic medical centers
2. The “customer” or intended end-user of WikiGuidelines are clinicians providing patient care in diverse geographical and economic settings, the majority of whom are generalists or specialists, rather than subspecialists
3. WikiGuidelines will incorporate principles of High Value Care (right care, right place, right cost) and Healthcare Quality (Safe, Effective, Patient-Centered, Timely, Efficient, Equitable)
4. WikiGuidelines provide Clear Recommendations (defined in section I.C.2) for questions addressed by hypothesis-confirming evidence (defined in section I.B)
5. For questions for which there is no hypothesis-confirming evidence, WikiGuidelines provide thoughtful suggestions and practical options based on the best available evidence (Clinical Review, defined in section I.C.3), rather than making Clear Recommendations
6. WikiGuidelines will acknowledge and call-out limitations of available data, and specific areas in need of future study
7. WikiGuidelines will focus on “real-world,” practical implementation, including:
    a. Emphasizing effectiveness (i.e., seen broadly at a population level) over efficacy (i.e., seen in carefully controlled experiments)
    b. Emphasizing principles of systems-based-practice: recognition that care for individual patients is delivered in the context of a larger system of healthcare, with considerations of feasibility, opportunity cost, and economic impact of care options
    c. The perspective of general and non-specialty practitioners, whether outpatient or inpatient, is a critically important focus, blended with subspecialty expertise
    d. Recognition that healthcare is a team-activity, and that all aspects of the team should be considered in the guidelines—the focus should not be on individual subspecialty physicians, nor their billing practices
    e. Recognition that feasibility of implementation and opportunity cost are important components of systems-based practice, and high intensity care has the potential to divert resources from other aspects of healthcare, and this cost should be included when factoring in the risks:benefits of individual Clear Recommendations or Clinical Reviews

B. Evidence Standards

1. Hypothesis-confirming evidence is defined as based on ALL of the following:
   a. At least two controlled, prospective interventional clinical studies that are adequately sized/powered and have undergone peer-review and are publicly posted, including at a minimum:
      1) One properly conducted, adequately powered randomized controlled trial (RCT); AND
      2) At least one other prospective, controlled clinical study, either a second RCT, a quasi-experimental pre-post study, a pragmatic clinical trial, or a carefully conducted historically controlled study
      3) Concordance from a supermajority (3/4) of the Drafting Committee during a meeting (see section III.E) that the above studies are valid, sufficiently generalizable, have a low risk of bias, and are without substantial flaws that preclude confidence in the conclusions relevant to the question being asked
      4) Totality of the evidence
         a) In assessing totality of the evidence to determine if it meets hypothesis-confirming standards, any discordance of available prospective study results should be considered
         b) Discordant results may still enable a totality summary to be considered hypothesis-confirming, if the discordance is a clear minority of the totality of data, and/or has a clear distinction in patient population from the majority data
         c) In all cases, discordant data should be explicitly discussed in the body of the guideline
   b. Hypothesis-generating evidence is anything not meeting hypothesis-confirming definition—when there is dispute about the level of evidence, the WikiGuidelines will err on the side of calling the evidence hypothesis-generating so as to not overstate the quality of evidence
   c. Areas where more evidence is required will be highlighted to help prioritize future research.

C. Template for Answering WikiGuideline Questions
1. Structure of Each Question Answered
   a. Questions to be answered will be selected by the Drafting Committee
   b. Each answer will begin with a clearly delineated indication of whether the answer is a Clear Recommendation (defined in I.C.2) or Clinical Review (defined in I.C.3)
   c. The Clear Recommendation/Clinical Review will be followed by a 1 or 2 sentence consensus, summary answer
   d. The consensus answer is followed by a 50-100 word Executive Summary
   e. It is desirable to graphically illustrate the results of the prospective studies (e.g., via forest plots, summary tables, visual abstracts, etc), and these data summaries are placed below the Executive Summary

   f. The Executive Summary/data figures/tables are followed by a broader discussion and synthesis of the literature with citations, leading to the conclusions from a totality of the evidence
   g. Each broader discussion will conclude with a section titled “Limitations of Data”, which will summarize important limitations that preclude extrapolation to other patients, and will also highlight areas of unmet need for future study
2. Clear Recommendations
   a. Clear Recommendations are written only when questions have been addressed by published hypothesis-confirming evidence (as defined in section II.B)
   b. Clear Recommendations offer specific guidance, and therefore should use words like “recommended” or “not recommended”
   c. When the distinction between hypothesis-confirmatory and hypothesis-generating evidence is unclear, Clinical Reviews will be written instead of Clear Recommendations to avoid overstating the level of evidence
   d. If during the drafting process, >25% of Drafting Committee members disagree that the prospective studies can be used to meet hypothesis-confirming evidence criteria (e.g., due to flaws in study design, execution, study power, discordant studies, etc), the question should be answered as a Clinical Review
3. Clinical Review
   a. Clinical Reviews are written when the available evidence is hypothesis-generating (i.e., does not meet hypothesis-confirming standards as defined in section II.B)
   b. Clinical Reviews do NOT offer definitive guidance, and should avoid words like “recommended”, “should”, “strongly preferred”, etc.
   c. Clinical Reviews focus on care choices, and typically discuss pros and cons and care options, using words like, “may”, “some clinicians prefer”, “in some cases”, “reasonable to consider”, “generally” etc.
   d. Clinical Reviews may take stronger language to recommend against taking action (e.g., “should not”), due to the principle of “first do no harm”, which sets a lower bar for decisions not to take action than decision to take action
   e. Clinical Review sections begin with a statement that hypothesis-confirming evidence is not available to answer the question
   f. Clinical Reviews identify, as possible, areas where future research could allow for Clear Recommendations
4. General Rules Applicable to Both Clear Recommendations and Clinical Reviews
   a. For all answers, areas of disagreement among drafters and public commenters will be specifically acknowledged in the text, including the rationale provided, the specific context of disagreement, and a general assessment of how much disagreement existed among the Drafting Committee (e.g., approximately how many, or what proportion, believed one position or the other)
   b. In contrast to traditional guidelines, there is no assessment of the strength of recommendations offered—there is either a Clear Recommendation made, or a Clinical Review written

D. Additional Sections to be Included

1. All guidelines documents will include a list of Drafting Committee members, and their disclosures, at the beginning of the document
2. The first written section of all guidelines documents will begin with the caveat: “WikiGuidelines evidence standards are summarized in its Charter (Appendix). These guidelines are intended only to provide insight into the opinions of the participating clinicians, and are not intended to establish care mandates, serve as medical-legal standards of care, or to replace individual clinician judgment for individual patients.”
3. All guidelines documents will conclude with the additional caveat: “These guidelines are based on published data available as of {list date of posting of the most recent guidelines}. These guidelines will be updated regularly by the Drafting Committee as additional data become available. WikiGuidelines participants understand that no clinical trial can ever be extrapolated to all possible patient care scenarios. Thus, it is our explicitly stated expectation that these guidelines not be used to replace individual clinician judgment for individual patients.”

II. Conflicts of Interest & Financial Considerations in Guidelines

A. Conflicts of Interest (COI)

1. WikiGuidelines recognizes that there are both individual and specialty-level conflicts of interest, and that while individual-level conflicts of interest typically garner concern among guideline authors, the specialty-level conflicts may play an even greater role in affecting recommendations for care
2. Specialty level COI will be addressed by attempting to include generalist members at all levels of WikiGuidelines governance, including intentionally including them on Drafting Committees, and by focusing on generalists as the customer of the guidelines, incorporating considerations of High Value Care and Healthcare Quality, and avoiding physician professional fee billing considerations when assessing evidence (see section I for each of these)
3. Drafting Committee members must disclose in the written guideline the total sum of private, third-party private (i.e., not primary employers) financial payments or equity during the last 24 months that are related to the practice of medicine (disclosed as <$10,000 per year, or ≥$10,000 per year), and their degree, practice affiliation/title, geographical area of practice, and clinical specialty
4. Individuals who have received ≥$10,000 of funding from, or own that level of equity in, any third-party, non-government source related to any given guidelines topic (including funding to conduct research) must recuse themselves from participating in discussions and drafting any sections related to the reimbursed matter
5. All products (drugs, devices, etc) mentioned in the guidelines should be referred to by their generic name
6. While not explicitly precluded, consideration should be given to whether individuals who are authors on specialty society guidelines should also be members of Drafting

Committees for the same conditions

B. Economic Considerations in Guidelines
1. Considerations of the economic impact of the recommendations made in the WikiGuidelines is encouraged, consistent with the focus of the guidelines (e.g., system-based practice, opportunity cost, High Value Care, and Quality standards) per section I.A
2. Such considerations should include variations in the economic considerations (e.g., drug costs) and logistical considerations (e.g., availability) between countries
3. When considering cost-efficacy analyses, WikiGuidelines will incorporate understanding that cost-efficacy does not equate to cost-reducing, that one party's cost is usually another's revenue (so is the perspective the healthcare system, the payer, the provider, etc?), and that cost-efficacy from the perspective of a societal payer is distinct from and frequently contradictory to the feasibility of implementation at the level of healthcare delivery
4. Non-industry studies of cost-efficacy will generally be preferred to industry studies because of the vested interests present in the assumptions of value

III. Governance

A. Open Source
1. The goal of WikiGuidelines is to allow providers to contribute to the content in close to real-time
2. Open source contributors add their contribution by contacting WikiGuidelines Drafting Committee members (section II.E), as opposed to directly modifying the guidelines

B. WikiGuidelines Members
1. Membership
   a. Any qualified practitioner with an active professional license in a relevant clinical field, including MD, DO, PharmD, or allied health professionals (e.g., NP, PA, CRNA, LCSW, etc) may participate as a Member
   b. For liability reasons, the Board of Directors may require practitioners sign an attestation as to the validity and active status of the professional license
   c. Each Member has 1 vote in WikiGuidelines Member meetings
   d. There is no limit to the number of practitioners who may participate as Members
   e. New Members may join by volunteering to the Steering Committee (see II.D), which may establish vetting criteria at the direction of the Board of Directors (see II.C)
   f. Removal of Members will be automatic if individual practitioners lose their relevant professional license
   g. Members may also be removed by a super-majority (3/4 vote) of all of the Members who participate in a meeting called with that issue specifically pre-set as an agenda item (presuming a quorum is met)

h. Members may also be removed by a unanimous vote of the Steering Committee or Board of Directors for cause, for reasons of ethical breaches, or for sustained non-participation

2. Member Meetings
   a. Member meetings will be called by consensus or majority vote of the Board of Directors (II.C) or the Steering Committee (II.D)
   b. Member meetings will require a quorum of a minimum of 10 Members or 20% of Members, whichever is larger
   c. Member meetings may be by web-access or phone, via social media, or email, and votes may be tabulated by any convenient format (verbal counting, email, web-based polls)
   d. Member meetings will be led by the Chair of the Steering Committee (see II.D), who will set the agenda and moderate the meeting
   e. Votes will be counted among members participating in the specific meeting, as long as a quorum is met, with non-participating members marked as absent and their votes counted is if abstained (not counted in either numerator or denominator)
   f. Meeting minutes will be kept and curated by the Chair of the Steering Committee or their designee
3. Member Responsibilities
   a. The Members will suggest disease areas to draft new guidelines—this does not require a vote; rather any individual member may submit a request to the Steering Committee with a rationale and list of supportive members with relevant expertise
   b. Members will serve as participants on the Steering Committee, Drafting Committee, and IT/Social Media Committee
   c. The Members may change the content of this Charter by supermajority (3/4) vote, presuming a quorum is met
   d. Once the organization becomes an incorporated non-profit, Members may also be required to pay annual dues by a super-majority (>3/4) vote of the Members, accompanied by a unanimous vote of the Board of Directors

C. WikiGuidelines Board of Directors

1. Board of Director Members (i.e., Directors)
   a. Once WikiGuidelines proceeds to the status of a non profit entity, a Board of Directors will be appointed, to consist of five members to start
   b. The initial Board will be selected by consensus of the Members, along with input from experts on non profit organizations, including legal and tax accounting
   c. The Board will select a Chair from among its members by consensus, or if consensus is not achieved, by majority vote
   d. Directors will serve 5 year terms, without term limits
   e. The Steering Committee (section II.D) will prepare lists of Members or other external experts interested in serving on the Board prior to holding elections every 5 years

        f. Directors may be removed for cause due to concerns of ethical breaches or failure to carry out Director duties by a unanimous vote of other Directors or a supermajority vote (>3/4) of Members

2. Board of Director Meetings
    a. A quorum of the Board of Directors meetings will require the presence of the Chair and at least 2/3 of the Board members (including the Chair)
    b. Meetings will be called by the Chair or by the request of any 2 Directors (including the Chair)
    c. Meetings may be held by web-access or phone, via social media, or email, and motions or votes may be tabulated by any convenient format (verbal counting, email, web-based polls)
    d. Board meetings will occur at least annually, and ad hoc as determined by the Board
    e. Meeting minutes will be kept and curated by the Chair or their designee
    f. Votes will be counted among Directors participating in the specific meeting, as long as a quorum is met, with non-participating members marked as absent and their votes counted is if abstained (not counted in either numerator nor denominator)
3. Director Responsibilities
    a. Directors will oversee activities of all Committees and all financial activities of the organization
    b. Directors will curate and oversee the Charter, including proposing revisions to Members which require a super-majority (3/4) vote of the Members to pass
    c. Directors may create qualification criteria for selection of Steering Committee members or Members
    d. Directors may remove Members or Steering Committee members as specified in sections II.B or II.D, respectively
    e. Directors will give an annual update to Members on the status of WikiGuidelines

D. WikiGuidelines Steering Committee

1. Membership
    a. The Members will select an initial 7-member Steering Committee based on popular vote
    b. Steering Committee members will serve 3 year terms, without term limits
    c. For the initial Steering Committee, terms may vary from 2 to 4 years to avoid all members potentially cycling off at once
    d. Members of the Steering Committee may also serve as members of the Board of Directors
    e. To support triennial elections, the Steering Committee will procure names of those who wish to be considered at the next election
    f. Resignations or removals during the 3 year term will be back-filled at an ad hoc meeting by majority votes of the WikiGuidelines Members

a. The Steering Committee will elect a Chair from among its members by majority vote
b. Once selected, the Chair may be changed during the three-year term of service by a super-majority (>3/4) vote of the Steering Committee, Board of Directors, or the Members
c. Steering Committee members may be removed for cause, including ethical breeches or failure to carry out Steering Committee responsibilities, based on a super-majority (>3/4) vote of Steering Committee members, Board of Directors, or the Members

2. Responsibilities
   a. Select topics to focus on for new guideline documents
   b. Tabulate/track WikiGuidelines members
   c. Call ad hoc meetings of the Members when needed to address important governance decisions or at the request of the Board or Members
   d. Create the agenda for the Member meetings
   e. Schedule triennial elections for new Steering Committee terms
   f. Select members of the Drafting Committee for each disease area
   g. Give final approval by consensus, or by majority vote only in the absence of consensus, to the first draft of each WikiGuideline before it is publicly posted
3. Meetings
   a. Steering Committee Meetings will be called by the Chair, or based on a motion from any two members of the Committee or at the request of the Board of Directors
   b. Meetings may be held by web-access or phone, via social media, or email, and motions or votes may be tabulated by any convenient format (verbal counting, email, web-based polls)
   c. Meeting motions may be made by email to the group
   d. A quorum for meetings will require the Chair and at least 4 other of the 7 members (total 5 members)
   e. The agenda for the meeting will be prepared in advance by the Chair
   f. Any member, including the Chair, may make motions
   g. Each member has 1 vote
   h. Committee decisions will generally be by consensus, but if there is disagreement, a majority vote will be required
   i. Votes will be counted among members participating in the specific meeting, as long as a quorum is met, with non-participating members marked as absent and their votes counted is if abstained (not counted in either numerator nor denominator)
   j. Meeting minutes will be kept and curated by the Chair or their designee

E. Drafting Committees
1. Membership

   a. For each topic selected by the Members or Steering Committee, a separate Drafting Committee will be established
   b. Drafting Committees may be constituted of any number of members, although generally having no more than 15-20 members may be desired for practical, operational purposes
   c. Initial members of each Drafting Committee will be selected by the Steering Committee, with input from Members
   d. There is no term limit for Drafting Committee members for each disease area
   e. Members may be removed by a super-majority vote (3/4) of the Drafting Committee, Steering Committee, Members, or Board of Directors
   f. Members may be added by majority vote of the Drafting Committee
   g. The members of the Drafting Committee will select a Chair by majority vote
   h. The Chair of the Drafting Committee may be removed by super-majority (>3/4) vote of the Steering Committee, Board of Directors, or Members

2. Responsibilities
   a. Drafting Committees will create the first draft of each WikiGuideline, including defining which questions to ask, and drafting Clear Recommendations or Clinical Reviews in response to each question
   b. The posted version of the initial draft of each guideline should be approved by consensus of the Drafting Committee (as well as approval by the Steering Committee, see II.D)—if consensus cannot be achieved among the Drafting Committee, a super-majority (3/4) vote can result in posting the document
   c. All members of the Drafting Committee are expected to contribute to conceptualization and drafting of the guideline document, and to be willing to be responsible for the entire content of the document, even sections that they did not write or contribute to, in accord with ICJME standards for publication authorship criteria
   d. Once posted, Drafting Committees will be responsible for curating the guideline in near real-time
   e. Drafting Committee members will serve as contact point for Members or external clinicians who wish to suggest revisions, alterations, additions, or deletions to the guideline
   f. Revisions, alterations, additions, or deletions will be curated by consensus, or if no consensus, by majority vote
   g. The Drafting Committee for each guidelines will serve as a Publications Committee for that specific guideline
      1) Decisions regarding publications will be made as above for the Drafting Committee
      2) The Drafting Committee may choose to create a separate Publications Subcommittee for its specific guideline, using a standard voting process as described above for the Drafting Committee
      3) Disputes about publication matters (e.g., where to submit, authorship, etc) will be resolved by the Steering Committee

3. Meetings
    a. Meetings of the Drafting Committees will be called by the Chairs, or based on a motion from any two members of the Committee
    b. A quorum for meetings will require the Chair and at least 2/3 of the members
    c. The agenda for the meeting will be prepared in advance by the Chair
    d. Meetings may be held by web-access or phone, via social media, or email, and motions or votes may be tabulated by any convenient format (verbal counting, email, web-based polls)
    e. Any member, including the Chair, may make motions
    f. Meeting motions may be made by email to the group
    g. Each member has 1 vote
    h. Committee decisions will generally be by consensus, but if there is disagreement, a majority vote will be required
    i. Votes will be counted among members participating in the specific meeting, as long as a quorum is met, with non-participating members marked as absent and their votes counted is if abstained (not counted in either numerator nor denominator)

F. IT/Media Committee

1. Membership
    a. The IT/Media Committee may be constituted of any number of members, although generally having no more than 7-10 members may be desired for practical, operational purposes
    b. Initial members will be selected by the Steering Committee, with input from WikiGuidelines Members
    c. The Members or Steering Committee may alter membership of any the IT/Media Committee by majority vote, for reasons of ethical breach, failure to carry out IT/Media Committee responsibilities
    d. There is no term to participating in the IT/Media Committee
    e. Members may be removed by a super-majority (3/4) vote of the IT/Media Committee, Steering Committee, Members, or Board of Directors
    f. Members may be added by majority vote of the IT/Media Committee
    g. The members of the IT/Media Committee will select a Chair by majority vote
2. Responsibilities
    a. The IT/Media Committee will be responsible for designing, establishing, and maintaining the WikiGuidelines home website, and for social media outreach
    b. As new guidelines are drafted the IT/Media Committee will assist the Drafting Committees in posting and curating the guidelines
3. Meetings
    a. Meetings will be called by the Chair, based on a motion from any two members of the Committee
    b. Meeting motions may be made by email to the group
    c. A quorum for meetings will require the Chair and at least 2/3 of the members

d. The agenda for the meeting will be prepared in advance by the Chair
e. Meetings may be held by web-access or phone, via social media, or email, and motions or votes may be tabulated by any convenient format (verbal counting, email, web-based polls)
f. Any member, including the Chair, may make motions
g. Each member has 1 vote
h. Committee decisions will generally be by consensus, but if there is disagreement, a majority vote will be required
i. Meeting minutes will be kept and curated by the Chair or their designee

G. Financing WikiGuidelines

1. WikiGuidelines may accept donations or advertising as needed to fund its non profit activities
2. Any revenue will be reinvested into operations; no distributions will be made to members
3. If WikiGuidelines activities become sufficiently burdensome or time-consuming, the Board of Directors and Steering Committee can consider whether payments should be made to participants to compensate them for time
4. Support staff may eventually be employed to assist with aspects of the day to day operations.
