# Supplementary material for: Use of Novel Strategies to Develop Guidelines for Management of Pyogenic Osteomyelitis in Adults: A WikiGuidelines Group Consensus Statement
Source: JAMA Netw Open. 2022 May 10;5(5):e2211321. doi: 10.1001/jamanetworkopen.2022.11321 (PMC9092201; doi:10.1001/jamanetworkopen.2022.11321)
Supplement: Supplement 2. — eAppendix. WikiGuidelines Overview, Executive Summary, and Discussion eFigure 1. Forest Plot of RCTs Comparing Success Rates of Patients Treated With Adjunctive Rifampin or Not for S. Aureus Osteomyelitis, With or Without Prosthetic Implants eFigure 2. Forest Plots of Subsets of RCTs Comparing Rifampin vs No Rifampin for Only Osteomyelitis Without PJI or Only Osteomyelitis With PJI eFigure 3. Random Effects Meta-analysis Forest Plot of RCTs Comparing Long-term Clinical Success Rates of Oral vs IV Antibiotic Therapy for Osteomyelitis in Adults eFigure 4. Random Effects Forest Plot of RCTs Comparing Shorter vs Longer Courses of Antibiotic Therapy for Vertebral Osteomyelitis and DFO in Adults eTable 1. Pooled Point Estimates of Sensitivity, Specificity, and Likelihood Ratios for Diagnostic Tests for Osteomyelitis eTable 2. Reasonable Empiric Antimicrobial Therapy Options with Published Data eTable 3. Antibiotic Concentrations in Bone eTable 4. Treatment Success Rates in Observational Studies of Oral Treatment of Osteomyelitis With or Without Infected Prosthesis in Adults eTable 5. Summary of Oral Antibiotic Doses Used in Published Studies for Osteomyelitis eTable 6. Summary of Antibiotic Durations for Osteomyelitis [file jamanetwopen-e2211321-s002.pdf]

Supplemental Online Content

Spellberg B, Aggrey G, Brennan MB, et al; WikiGuidelines Group. Use of novel strategies to develop guidelines for management of pyogenic osteomyelitis in adults: a WikiGuidelines Group consensus statement. *JAMA Netw Open.* 2022;5(5):e2211321. doi:10.1001/jamanetworkopen.2022.11321

eAppendix. WikiGuidelines Overview, Executive Summary, and Discussion

eFigure 1. Forest Plot of RCTs Comparing Success Rates of Patients Treated With Adjunctive Rifampin or Not for S. Aureus Osteomyelitis, With or Without Prosthetic Implants

eFigure 2. Forest Plots of Subsets of RCTs Comparing Rifampin vs No Rifampin for Only Osteomyelitis Without PJI or Only Osteomyelitis With PJI

eFigure 3. Random Effects Meta-analysis Forest Plot of RCTs Comparing Long-term Clinical Success Rates of Oral vs IV Antibiotic Therapy for Osteomyelitis in Adults

eFigure 4. Random Effects Forest Plot of RCTs Comparing Shorter vs Longer Courses of Antibiotic Therapy for Vertebral Osteomyelitis and DFO in Adults

eTable 1. Pooled Point Estimates of Sensitivity, Specificity, and Likelihood Ratios for Diagnostic Tests for Osteomyelitis

eTable 2. Reasonable Empiric Antimicrobial Therapy Options with Published Data

eTable 3. Antibiotic Concentrations in Bone

eTable 4. Treatment Success Rates in Observational Studies of Oral Treatment of Osteomyelitis With or Without Infected Prosthesis in Adults

eTable 5. Summary of Oral Antibiotic Doses Used in Published Studies for Osteomyelitis

eTable 6. Summary of Antibiotic Durations for Osteomyelitis

This supplemental material has been provided by the authors to give readers additional information about their work.

© 2022 Spellberg B et al. *JAMA Network Open.*

eAppendix. WikiGuidelines Overview, Executive Summary, and Discussion

Disclaimer

WikiGuidelines evidence standards are summarized in its Charter. These guidelines are intended only to provide insight into the opinions of the participating clinicians, and are not intended to establish care mandates, serve as medical-legal standards of care, or to replace individual clinician judgment for individual patients.

Introduction

An important limitation of traditional clinical guidelines is the frequent dissociation between quality of evidence and strength of recommendations.[1-6] As a result, some past guideline recommendations have endorsed harmful care, which was only subsequently recognized when high quality, prospective controlled trials were conducted.[7] To overcome this limitation, we developed a novel approach called WikiGuidelines, which establish Clear Recommendations only when high quality, hypothesis-confirming evidence is available (see Charter).

Our initial social media poll revealed a desire for renewed guidance on a common infectious disease, pyogenic osteomyelitis. Pyogenic osteomyelitis occurs at a rate of approximately 20 cases per 100,000 person-years, with rates rising among diabetic and elderly patients, and those with prosthetic joints.[8-10] In low- and lower-middle-income countries (LMIC), osteomyelitis may be more common in younger patients as a result of traumatic injury.[11] Nevertheless, the global economic burden of osteomyelitis is considerable for high income and LMIC.[9,10,12-14]

Osteomyelitis is an ancient disease, with the earliest documented case in an unfortunate, 250 million year old dimetredon with a fractured spinal shaft.[15] In the modern era, radiography, surgical methods, and antibiotics have revolutionized its management. However, these successful interventions have resulted in long-standing diagnostic and therapeutic paradigms despite lack of strong evidence, including the need for diagnostic X-rays and intravenous-only antibiotic therapy for all patients.[16] Recent studies have begun to challenge these dogmas (e.g., is routinely obtaining plain X-rays high value, can oral antibiotics be administered?[16-18]). This guideline focuses on data regarding management of pyogenic osteomyelitis in adults.

© 2022 Spellberg B et al. *JAMA Network Open.*

Methods.

The WikiGuidelines group formed on Twitter by participants who were dissatisfied with traditional guideline methodologies. The group constructed a Charter that specifically chose not to use the GRADE system for evaluating strength of evidence due to previously published concerns regarding bias, poor interrater reliability, and, most importantly, the dissociation between strength of recommendation and quality of supporting evidence.[1-7]

Instead, WikiGuidelines seek to incorporate the "humility of uncertainty"[7] by only offering Clear Recommendations when reproducible, high-quality, hypothesis-confirming evidence is available. High quality, hypothesis-confirming data is based on, at a minimum: 1) one properly conducted, adequately powered randomized controlled trial (RCT); AND 2) at least one other concordant, prospective, controlled clinical study, either a second RCT, a quasi-experimental pre-post study, a pragmatic non-randomized clinical trial, or a carefully conducted historically controlled study. In the absence of such data, WikiGuidelines offer guidance based on Clinical Reviews that discuss care choices. However, recognizing the core principle of "first do no harm," authors could recommend against the routine provision of unsubstantiated care as part of Clinical Reviews. We also sought to incorporate principles of High Value Care (i.e., right care, right place, right cost) and healthcare quality (i.e., safe, effective, patient-centered, timely, efficient, equitable).[19]

Drafting members for each question conducted their own literature review using PubMed, including all years and all languages, with key words that varied by the question being asked. Articles were assessed for quality/inclusion by criteria specified in the Charter. References from identified articles were also searched for potential inclusion. When divergent opinions on article interpretation or clinical practice existed among the authors, we did not attempt to force consensus; rather, in accord with the Charter, we sought to transparently highlight those diverging opinions by discussing care alternatives. For answers based on more than one relevant RCT, meta-analysis was conducted using Review Manager 5.4.1 (RevMan, Cochrane Collaboration, UK).

The consortium that established the WikiGuidelines Charter consisted of 63 participants from 8 countries: Australia, Canada, Colombia, Saudi Arabia, Spain, Switzerland, the United Kingdom, and the United States of America. These participants included physicians, pharmacists, and microbiologists, with expertise in General Internal Medicine/Hospital Medicine, Pediatrics, Infectious Disease, Orthopedic Surgery, Pharmacology, and Medical Microbiology.

The participants addressed seven questions regarding the diagnosis and management of pyogenic osteomyelitis but found data sufficient to establish Clear Recommendations for only two: 1) oral antibiotic therapy for pyogenic osteomyelitis, and 2) duration of therapy. In contrast, 5 questions were addressed with Clinical Reviews in the absence of high-quality data: diagnosis of pyogenic osteomyelitis, management of osteomyelitis underlying pressure ulcers, appropriate timing of empiric therapy, rational selection of antimicrobial options, and use of serial biomarkers or imaging studies to evaluate therapeutic response.

© 2022 Spellberg B et al. *JAMA Network Open.*

Executive Summary of Clear Recommendations or Clinical Review Conclusions for Osteomyelitis

1. How should the diagnosis of osteomyelitis be established?
Clinical Review *(insufficient quality of evidence to enable a Clear Recommendation)*:
a. Osteomyelitis without Prosthetic Joint Infections (PJI)
Based on observational studies, we do not recommend the routine use of plain X-rays (inadequate sensitivity, specificity) or CT scans (inadequate sensitivity) for all patients with a possible diagnosis of osteomyelitis (Table 1) as they may result in unnecessary radiation and use of resources. However, these studies may be helpful if a fracture or other non-infectious cause of bone pain (e.g., tumor, foreign object, etc.) is prioritized on the differential diagnosis, and/or the pre-test probability of osteomyelitis is lower (e.g., ≤15%). Magnetic resonance imaging (MRI) and certain tagged white cell scans are the most accurate imaging modalities for diagnosing osteomyelitis. Inflammatory biomarkers are insufficiently accurate, and we do not recommend their routine use for osteomyelitis diagnosis. Blood cultures have variable sensitivity but if the patient has systemic symptoms or risk factors for bacteremia (e.g., intravenous drug use), isolating likely pathogens (e.g., *Staphylococcus aureus*) can be helpful to target therapy, and potentially obviate the need for bone biopsy. If available, bone biopsy for histopathology is highly accurate if positive, but has poor sensitivity to rule out osteomyelitis if negative. Culture of biopsy specimens of the affected bone may help identify etiology and target antimicrobial therapy.

b. Diabetic Foot Osteomyelitis (DFO)
Based on observational studies, plain X-rays have low sensitivity and specificity for diagnosing DFO (Table 1, with references). The probe-to-bone (PTB) test is simple, non-invasive, and has reasonable sensitivity and specificity as a diagnostic method for DFO, which may preclude the need for imaging in some settings. MRI and certain tagged white cell scans are the most accurate imaging modalities for diagnosing DFO, although their specificities are lower than their sensitivities. Inflammatory biomarkers are insufficiently accurate, and we do not recommend their routine use for diagnosis. If available, percutaneous bone biopsy for deep microbiological cultures may help target antimicrobial therapy; surface cultures are not accurate and not recommended.

c. Osteomyelitis with PJI
There is no established, accurate referent standard diagnostic test for PJI. Certain tagged white cell scans are the most accurate imaging studies for PJI (Table 1, with references), however given the limitations of individual tests, published algorithms are sometimes recommended to establish the diagnosis. Data are limited and inadequate to compare the relative accuracies of competing algorithms. Practically, the diagnosis is typically made from a combination of history, physical exam, imaging studies to assess alternate causes of pain and instability, inflammatory markers, synovial fluid analysis, and/or operative specimens. Molecular diagnostic testing is a promising approach, but data are mixed and inadequate to recommend for or against its use as of 2021.

© 2022 Spellberg B et al. *JAMA Network Open.*

2. What is the appropriate management for osteomyelitis underlying a pressure ulcer?

Clinical Review *(insufficient quality of evidence to enable a Clear Recommendation)*:
Observational studies indicate that imaging and inflammatory biomarkers are not diagnostically accurate for osteomyelitis underlying a pressure ulcer and we do not recommend their routine use for this purpose. Antibiotics have not been shown to be of benefit, and may be of harm, in the absence of surgical wound closure, but osteomyelitis may increase the risk of surgical flap failure.[20,21] Therefore, it may be preferable to avoid the routine use of antibiotic therapy for osteomyelitis underlying a pressure ulcer unless deep bone biopsy confirms osteomyelitis and surgical wound closure is planned, or the patient has accompanying sepsis syndrome or local soft tissue infection. Irrespective of antibiotic use, a multi-modal therapeutic approach includes nutritional optimization, wound debridement and care, pressure off-loading, and psychosocial management.

3. When should empiric therapy be administered in the treatment of osteomyelitis?

Clinical Review *(insufficient quality of evidence to enable a Clear Recommendation)*:
Some observational studies suggest that administration of antibiotics prior to bone biopsy or surgical management may modestly decrease yield of bone cultures for patients with osteomyelitis, including DFO and PJI. Thus, presuming other microbiological methods (e.g., blood cultures) have not already established a microbial etiology, it is reasonable to consider deferring antimicrobial therapy initiation until bone/joint microbiological samples are obtained for clinically stable patients. However, other studies are not concordant, and histopathology results are unlikely to be affected by prior short-term antibiotics. Decisions regarding the delay of empiric therapy therefore balance potential harm due to the risk of progression of life-threatening infection (e.g., sepsis) or impending spinal cord compression against the potential benefit of microbiological data.

4. Are there preferred antibiotics with which to treat osteomyelitis?

Clinical Review *(insufficient quality of evidence to enable a Clear Recommendation)*:

a. <u>Which empiric antimicrobial agents are preferred for osteomyelitis?</u>
Based on data from observational studies, if antibiotic therapy cannot be delayed until culture availability, it is reasonable to empirically cover aerobic gram-positive cocci, especially *Staphylococcus aureus,* and gram-negative bacilli (Table 2). Many practitioners routinely provide anaerobic coverage for DFO, however comparative data are not available to establish the clinical benefit or harm of this approach. Inclusion of empiric therapy targeting methicillin-resistant *S. aureus* (MRSA) or *Pseudomonas aeruginosa* depends on the presence of specific risk factors (see sections b and c below, respectively). In all cases, local susceptibility patterns, patient-specific risk factors, and prior culture data influence the choice of antibiotic selection. Culture results can be used to tailor empiric therapy when possible.

b. <u>When should antimicrobial coverage targeting methicillin-resistant *S. aureus* (MRSA) be included?</u>

© 2022 Spellberg B et al. *JAMA Network Open.*

Based on culture data from observational studies, inclusion of empiric anti-MRSA coverage depends on local prevalence and patient-specific risk factors, such as known colonization status (which is the biggest individual risk factor), prior positive cultures, and healthcare exposure. In a setting with low MRSA incidence, no known MRSA colonization or prior positive cultures, and minimal healthcare contact, it is reasonable to withhold empiric MRSA coverage.

c. When should antimicrobial coverage against *P. aeruginosa* be included?
Based on culture data from observational studies, routine use of empiric antipseudomonal therapy for osteomyelitis is unnecessary. Such agents are added in the presence of specific risk factors, including patients with chronic wounds who have: 1) been exposed to multiple prior courses of antibiotics; 2) previously had cultures positive for *P. aeruginosa*; 3) gangrenous wounds; 4) had a recent surgical procedure (e.g., <3 months, as with early PJI); or 5) specific sites of infection particularly associated with pseudomonal infection (e.g., malignant otitis externa).

d. Does bone penetration of an antimicrobial agent matter clinically, and should it be used to select therapy?
Outcome data related to antibiotic bone penetration are limited for osteomyelitis. Thus, theoretical bone penetration (Table 3, with references) is not the primary driver of antibiotic selection; published clinical outcomes data are more relevant.

e. Does adjunctive rifampin alter osteomyelitis treatment outcomes; for which organisms is rifampin therapy potentially useful, and if it is used, is there a preferred dosing?
Some observational studies and small RCTs suggest addition of rifampin to standard therapy may improve long-term outcomes by reducing relapse of osteomyelitis, with or without retained implants/hardware. However, other observational studies and one small RCT are contrary. Overall, the data are mixed and remain uncertain (Figures 1-2). The use of rifampin in this setting is based on culture results (principally targeting gram-positive cocci or non-fermenting gram-negative bacilli) and individual patient risk:benefit considerations, acknowledging the uncertainty of the efficacy data, side effects, and potential drug interactions (especially those disrupting stable, chronic medications, such as oral anticoagulants or opiates). Studies have not elucidated optimal total daily dosing, except that 450-600 mg per dose likely increases pharmacodynamic (PD) target attainment and adherence compared to 300 mg multiple daily dosing.[22-26]

f. What is the role of long-acting glycopeptide antibiotics in treating osteomyelitis?
One RCT and several small, largely single-center, observational studies have examined the role of two long-acting glycopeptides, dalbavancin and oritavancin, for the treatment of osteomyelitis.[27,28] In these studies, the long-acting agents performed similarly to comparator regimens. There are no data supporting their superiority, so the use of these agents is based on risk:benefit considerations, as well as cost and complexity vs. other regimens for individual patients and health system contexts.

© 2022 Spellberg B et al. *JAMA Network Open.*

5. Is oral therapy appropriate for the treatment of osteomyelitis, and if so, what are reasonable patient selection criteria for administration?

Clear Recommendation:
Based on eight concordant RCTs comparing intravenous (IV) to oral therapy[17,29-35] (Figure 3) and nine RCTs in which oral therapy was predominantly used in both arms,[36-44] we recommend oral antibiotic therapy with a drug/dose used in published studies as a reasonable option for osteomyelitis of any type (i.e., hematogenous, prosthetic, and contiguous, the latter including vertebral and DFO) for patients who: 1) are clinically stable (hemodynamically and at the site of infection, e.g., no spinal instability); 2) have adequate source control (i.e., not requiring further procedural drainage and without persistent bacteremia); 3) are likely to absorb oral medications from a functioning gastrointestinal (GI) tract; 4) have an available regimen used in published osteomyelitis studies to cover likely target pathogens; and 5) have no psychosocial reasons that preclude the safe use of oral therapy. There is no required minimum duration of IV lead-in; patients may be switched to oral therapy when all the above criteria are met, even at the empiric therapy stage. Specific drug options and doses are discussed in the detailed review section (Table 4, Figure 3, Table 5).

6. What is the role and optimal utilization of serial biomarkers and/or imaging studies for assessing treatment response in osteomyelitis?

Clinical Review *(insufficient quality of evidence to enable a Clear Recommendation)*:
In the absence of RCTs, observational studies have generally found that neither serial inflammatory biomarkers (e.g., erythrocyte sedimentation rate [ESR], C-reactive protein [CRP]) nor routinely repeated imaging accurately predict long-term treatment success for osteomyelitis or PJI for individual patients, nor do they meaningfully alter treatment decisions beyond clinical observation. Thus, following inflammatory biomarkers and repeated imaging may not offer benefit or contribute to high value care in most patients. Nonetheless, repeated imaging may be useful for patients who are clinically failing therapy to inform source control attempts, identify mechanical complications such as pathological fracture, and/or to trigger reconsideration of the initial diagnosis.

7. What is the appropriate duration of therapy for typical cases of osteomyelitis?

Clear Recommendation:
<u>Osteomyelitis (including DFO) without a Retained Implant</u>
Based on two RCTs (Figure 4),[39,44] and concordant observational studies, we recommend a maximum of 6 weeks of antibiotic therapy for hematogenous or contiguous pyogenic osteomyelitis (including DFO), assuming adequate source control (i.e., no undrained abscesses too large to be treated with antibiotics alone, possibly ≥ 2-3 cm in diameter) and no retained prosthetic implant (Table 6). Insufficient data are available to establish a Clear Recommendation for durations shorter than 6 weeks (see Clinical Review below).

Clinical Review *(insufficient quality of evidence to enable a Clear Recommendation)*:

© 2022 Spellberg B et al. *JAMA Network Open.*

a. Osteomyelitis (including DFO) without a Retained Implant

Based on small RCTs, 3 or 4 weeks may be a reasonable duration of antibiotics for debrided osteomyelitis, whether hematogenous or contiguous (including DFO); however, confirmatory data are desired. Based on observational studies and one small RCT, it is reasonable to refrain from antibiotic use after total resection of infected bone if the treating physicians are confident that all infected bone has been resected. If administered in this setting, we do not recommend exceeding 2-5 days of therapy if there is no complicating soft tissue infection.

b. Osteomyelitis with a Retained Implant (including PJI)

Based on the Duration of Antibiotic Treatment in Prosthetic Osteo-articular infection (DATIPO) RCT, participating experts unanimously agree that 12 is preferred to 6 weeks of antibiotics for PJI treated with debridement, antibiotics, and implant retention (DAIR).[43] Some experts also clearly prefer 12 weeks of antibiotics for PJI treated with prosthetic exchanges. However, others believe that equipoise remains between 6 vs. 12 weeks for these patients, particularly if *S. aure*us is not the etiologic pathogen, or for 1-stage exchanges, or 2-stage revisions with negative cultures prior to implantation.

Duration of therapy for other infected implants is not clear. A reasonable strategy, without evidence for or against, may be to treat with antibiotics until the bone heals sufficiently enough that the implants can be removed, such as in cases of fracture. Finally, chronic oral suppressive therapy may be considered for patients for whom the risk:benefit of curative surgery is deemed unacceptable; however, available data do not well-define the risks:benefits of this approach.

© 2022 Spellberg B et al. *JAMA Network Open.*

Detailed Responses

Question 1: How should the diagnosis of osteomyelitis be established?

Executive Summary:
Osteomyelitis without PJI
Based on observational studies, plain X-rays have inadequate sensitivity and specificity, and CT scans have inadequate sensitivity, for routine use to diagnose of osteomyelitis (Table 1, with references). Furthermore, routinely obtaining X-rays or CT scans exposes patients to excess radiation, and occupies considerable cost and radiology technician time, which can delay care for other patients. Hence, we do not recommend their routine use to evaluate for osteomyelitis in all patients but agree that X-rays are rational if other diseases/injuries (e.g., fracture, tumor, foreign body, etc.) are prioritized on the differential diagnosis, and/or if a patient has a low (e.g., ≤15%) pre-test probability, such that a negative X-ray is sufficient to rule out osteomyelitis without obtaining an MRI. MRI and certain tagged white cell scans have the highest overall accuracy of routinely available diagnostic tests for osteomyelitis. For patients who cannot receive an MRI (e.g., incompatible cardiac devices), positron emission tomography (PET) scans are an alternative, and CT scans may be useful if positive (high specificity, lower sensitivity). Inflammatory biomarkers are not sufficiently accurate, or additive to imaging, to diagnose osteomyelitis. Therefore, we do not recommend their routine use. Blood cultures are relatively non-invasive and inexpensive, and if positive may obviate the need to proceed to more invasive microbiological testing (e.g., bone biopsies). Bone biopsies may be difficult to obtain depending on the practice setting; however, biopsy histopathology is highly specific and if positive is helpful to rule in osteomyelitis. Unfortunately, due to their low sensitivity, negative bone biopsy results may not be helpful to rule out osteomyelitis. As importantly, biopsy histopathological information and special stains can be critical to identify atypical infectious syndromes (e.g., granulomas, acid-fast bacilli (AFB), or silver stain) or alternative diagnoses. Biopsy cultures that are obtained aseptically are useful to target antimicrobial therapy. Very limited data are available to assess the accuracy of molecular diagnostics for osteomyelitis outside the context of PJI; their overall cost-efficacy and clinical benefit are unclear, although they may be rational to attempt in patients who are clinically failing therapy and for whom traditional methods have failed to establish a microbial diagnosis.

DFO
Based on observational studies, plain X-rays have low sensitivity and specificity for diagnosing DFO (Table 1). Of note, plain X-ray sensitivity likely increases with time, as new bony erosions are expected to occur without treatment. The probe-to-bone (PTB) test is simple, non-invasive, requires minimal resource utilization, and has reasonable sensitivity and specificity, making it a good initial test for diagnosing DFO, which may preclude the need to progress to imaging. Some experts have suggested that the combination of plain X-ray and PTB has higher sensitivity than PTB alone; however, limited data suggest an overall lower accuracy of combination testing compared to PTB alone. Based on observational studies, MRI is the most sensitive routinely available imaging test for DFO, although its specificity is lower, and false positives may occur particularly in neuropathic feet. In patients who cannot receive an MRI (e.g., incompatible cardiac devices), PET scan or certain tagged white blood cell scans have the best operating characteristics; one caveat is that performance characteristics of tagged white blood cell scans

may be lower in settings of poor tissue perfusion. Inflammatory biomarkers have not been found to be reliably accurate, or additive to imaging. Therefore, we do not recommend their routine use. Surface swabs are inaccurate for diagnosing DFO or establishing etiologic organisms in bone and should not be ordered. If available, bone biopsies with histopathology and culture help confirm the diagnosis and target antimicrobial therapy.

Osteomyelitis with PJI

No single test has been established as a referent standard for the diagnosis of PJI, and accuracy of individual tests are variable in observational studies. Thus, a multi-modal, algorithmic approach is typically used to diagnose PJI. However, studies are not available to enable a recommendation of one published algorithm over any other. Numerous observational studies have evaluated novel approaches to establishing the microbial etiology of PJI, including sonication and liquid culture, polymerase chain reaction (PCR), and next generation sequencing methods. Such data are mixed, and of sufficiently low quality to preclude a recommendation for or against their use, particularly given the excess resources they require. Molecular methods may be rational to attempt when more traditional methods have failed to establish a microbial diagnosis, particularly if the patient is not clinically responding to antimicrobial therapy. High quality, prospective studies are needed to improve the diagnosis of PJI.

Table 1: Pooled Point Estimates of Sensitivity, Specificity, and Likelihood Ratios for Diagnostic Tests for Osteomyelitis

Test	Sensitivity	Specificity	+LR*	-LR*	Reference
**Osteomyelitis without PJI**					
X-rays	70%	82%	3.9	0.4	45
CT Scans	70%	90%	7.0	0.3	45
MRI	96%	81%	5.1	0.05	45
Nuclear Medicine Scintigraphy†	84%	71%	2.9	0.2	45
White Cell Tagged Scans	87%	95%	17.4	0.1	45
PET	85%	93%	12.1	0.2	45
SPECT	95%	82%	5.3	0.06	45
ESR	49%-79%	50-80%	1.6-3.8	0.3-0.4	46-48
CRP	45%-76%	59%-71%	1.1-2.6	0.3-0.8	46-48
Biopsy (histopathology)	52%	>99%	>50	0.5	49
**DFO**					
X-rays	62%	78%	2.8	0.5	50
MRI	93%-96%	75%-84%	3.7-6.0	0.05-0.09	50,51
Nuclear Medicine Scintigraphy†	85%	68%	2.7	0.2	50
White Cell Tagged Scans	91%-92%	75%-92%	3.6-11.5	0.09-0.1	51
PET	84%	93%	12.0	0.2	50
ESR	60%-81%	56%-90%	1.4-8	0.2-0.7	52-55
CRP	49%-76%	55%-80%	1.1-3.8	0.3-0.9	52-54,56
Probe-to-bone	87%	83%	5.1	0.2	57

© 2022 Spellberg B et al. *JAMA Network Open.*

**PJI**‡					
X-rays	14%	70%	0.5	1.2	[58]
MRI	65%-94%	73%-99%	2.4->50	0.06-0.5	[58-60]
Nuclear Medicine Scintigraphy†	83%-94%	69%-90%	2.7-9.4	0.07-0.2	[61-63]
White Cell Tagged Scans	93%-100%	91%-100%	10->50	0.08-<0.01	[64,65]
PET	82%-95%	39%-87%	1.3-7.3	0.06-0.5	[66-68]
ESR	75%	70%-87%	2.5-5.8	0.3-0.4	[69,70]
CRP	88%-97%	74%	3.4-3.7	0.04-0.2	[69,70]
IL-6	97%	91%	10.8	0.03	[69]
Synovial WBC Count	88%	93%	12.6	0.1	[71]
Synovial PMN%	90%	88%	7.5	0.1	[71]
Synovial Culture	62%	94%	10.3	0.4	[72]

PJI, prosthetic joint infection; LR, likelihood ratio; CT, computerized tomography; PET, positron emission tomography; SPECT, single photon emission computed tomography; MRI, magnetic resonance imaging; ESR, erythrocyte sedimentation rate; CRP, C-reactive protein rate; DFO, diabetic foot osteomyelitis; IL-6, Interleukin-6; WBC, white blood cell; PMN, polymorphonuclear

*A positive LR ≥5 is helpful and ≥10 is very helpful at shifting post-test probabilities; a negative LR ≤ 0.2 is helpful and ≤ 0.1 is very helpful at shifting post-test probabilities.

†Excluding tagged white cell studies, which are considered separately.

‡Because there is no identified optimal referent standard for the diagnosis of PJI, sensitivity, specificity, and LRs for tests for PJI should be considered to be uncertain estimates.

© 2022 Spellberg B et al. *JAMA Network Open.*

Overall Summary:

No RCTs have been published that define optimal diagnostic strategies for osteomyelitis. However, numerous observational studies of various designs, sizes, and quality have been published that evaluated the accuracy of various diagnostic modalities.

Osteomyelitis without PJI

Imaging Studies (Table 1)

Plain Films
The primary utility of plain X-rays in evaluation of patients with osteomyelitis is to exclude other diagnoses, such as fractures, metallic foreign bodies, or malignancies.[73] Periosteal elevation, the most common X-ray finding suggesting osteomyelitis, is neither sensitive nor specific for osteomyelitis.[73,74] It can be caused by any condition in which there is inflammation of the tissue layers above the bone, as well as tumors, or even stress fractures.[73,74] Furthermore, the earliest bony change in osteomyelitis is marrow edema, which cannot be detected by X-rays. As such, plain X-rays are typically normal during the earlier phases of osteomyelitis and X-ray findings such as cortical destruction and bony erosions are rare, and typically found after prolonged periods of infection.[73-75] However, the sensitivity of X-rays does likely increase with time in untreated infections due to progressive bony erosion.

Llewellyn et al. conducted the most recent and comprehensive, systematic review and meta-analysis of various imaging modalities at diagnosing osteomyelitis across all body sites.[45] Eighty-one studies were included. Plain X-rays had a pooled (95% CI) sensitivity and specificity of only 70% (62%-79%) and 82% (70%-90%), respectively, resulting in poor positive and negative likelihood ratios (3.9 and 0.4, respectively). There was no noted variation in study accuracy by type of osteomyelitis or body site. Thus, X-rays are neither sensitive nor specific for the diagnosis of osteomyelitis.

Computerized Tomography (CT) Scans
CT scans may detect cortical disruption of bone and play a role in identifying target sites for needle biopsies in osteomyelitis. However, CT scans cannot detect marrow edema, making them insensitive for the diagnosis of osteomyelitis, particularly in earlier phases.[73,75,76] Llewellyn et al. found that CT scans had a pooled (95% CI) sensitivity and specificity of 70% (40%-89%) and 90% (58%-98%), respectively, for diagnosing osteomyelitis.[45] These results indicate that a positive CT scan may be helpful to diagnose osteomyelitis (positive likelihood ratio of 7.0), but a negative CT scan is less helpful in ruling it out (negative likelihood ratio of 0.3).

Nuclear Medicine Studies
Similarly to plain X-rays and CT scans, Llewellyn et al. reported that nuclear medicine scintigraphy studies of various types (excluding certain tagged white cell scans, which are considered separately, below) had relatively poor pooled (95% CI) sensitivity and specificity at 84% (72%-91%) and 71% (58%-81%), yielding positive and negative likelihood ratios of 2.9 and 0.2 respectively, for diagnosing osteomyelitis.[45] Thus, such scans may be modestly helpful if negative, depending on pre-test probability, but do not substantively alter pre-test probability if positive. Similarly, in a study of 30 patients with possible osteomyelitis, 15 of whom were subsequently confirmed to have osteomyelitis affecting a range of bones, and 15 with other

inflammatory, malignant, or traumatic/degenerative injuries, bone scans were positive in all nine patients with osteomyelitis in whom they were obtained.[77] However, they were also falsely positive in 11 of 12 (specificity 8%) patients who were ultimately diagnosed with other conditions.[77] These results are consistent with findings from a more recent study in which the accuracy of triple phase bone scanning and single photon emission computerized tomography (SPECT) scanning were poor to mixed for diagnosing osteomyelitis (ranging from 60-90% sensitivity/specificity, likelihood ratios <5).[78]

Llewellyn et al. reported superior accuracy of certain tagged white cell scans, positron emission tomography (PET) scans, and SPECT scans for diagnosing osteomyelitis.[45] Specifically, tagged white cell scans had a pooled (95% CI) sensitivity and specificity of 87% (75%-94%) and 95% (85%-98%), yielding positive and negative likelihood ratios of 17.4 and 0.1, respectively. PET scans had pooled (95% CI) sensitivity and specificity of 85% (72%-93%) and 93% (83%-97%), yielding positive and negative likelihood ratios of 12.1 and 0.2, respectively.[45] SPECT scans had a pooled (95% CI) sensitivity and specificity of 95% (88%-98%) and 82% (62%-93%), yielding positive and negative likelihood ratios of 5.3 and 0.06, respectively. Thus, tagged white cell and PET scans are more specific than SPECT scans, and the latter are more sensitive.

MRI

MRI is the most accurate, generally available radiographic method to identify osteomyelitis, with sensitivities in excess of 90-95% and specificities of 80-90%, resulting in positive likelihood ratios of 5-10 and negative likelihood ratios of 0.056-0.125.[45,50,73,74,76,79] Furthermore, MRIs do not expose the patient to ionizing radiation, and become positive for osteomyelitis substantially earlier than X-ray and CT imaging.[75]

In their recent review of 81 studies, Llewellyn et al. reported that the pooled (95% CI) sensitivity and specificity of MRI for diagnosing osteomyelitis were 96% (92%-98%) and 81% (71%-88%), respectively (positive and negative likelihood ratios of 5.1 and 0.05, respectively).[45] MRI has also been found to be accurate in diagnosing skull-base osteomyelitis. In one study of patients with malignant otitis externa, the sensitivity and specificity of MRI using specific diffusion weighted imaging cut-offs was 86-90% and 79-90%, respectively (positive and negative likelihood ratios of ~6 and ~0.1 respectively).[80] Similarly, in a case series of patients with skull base osteomyelitis or tumors, MRIs were able to distinguish infection from malignancy.[81]

Summary of Radiographic Studies for Osteomyelitis without PJI

In summary, a consistent and substantial body of literature has found that plain X-rays, CT scans, and various forms of scintigraphy (excepting tagged white cell scans) are relatively inaccurate for diagnosing osteomyelitis. X-rays and CT scans can be specific when bony destruction is encountered without an alternative explanation, but these findings are late, may be rarely encountered, and cannot be distinguished from other destructive bony processes without further investigation (e.g., biopsy, surgery). Bone scans can be sensitive but are highly non-specific for osteomyelitis.

While plain X-rays are often recommended to be routinely obtained as an initial diagnostic tool in various guidelines sources, we emphasize that this practice may be rooted in historical inertia rather than published data. Indeed, one review of guidelines has acknowledged that this recommendation by others is based on low quality evidence.[82] As plain X-rays are not

accurate for diagnosing osteomyelitis, their primary role may be in diagnosing a patient for whom there is a prioritized concern for non-infectious causes of disease, such as fracture or other mechanical causes of bone pain. In a patient with a low initial pre-test probability of osteomyelitis (e.g., 15% or less), a negative X-ray can preclude the need for a diagnostic MRI (shifting post-test probability to ≤ 5%). However, one might question the need for an MRI in such a situation, irrespective of X-ray results.

We emphasize that obtaining numerous relatively low-cost tests adds considerable overall cumulative cost to healthcare. More importantly, obtaining a high volume of such tests occupies a considerable amount of radiology technician time, which can create backlogs and delays in care for other patients. Thus, the notion that X-rays are less expensive or resource intensive than other options may be a false conclusion given the high volume of such studies, and lack of adequate consideration regarding the overall time required for radiology technicians to obtain and process such films, the clinician time taken to interpret them, and importantly, their low overall diagnostic value.

Overall, MRIs appear to be the most sensitive of all radiographic studies, and they have the advantage of not exposing patients to ionizing radiation. PET scans and tagged white cell scans may be more specific than MRIs, but are less sensitive, do expose patients to ionizing radiation, and may be more expensive depending on the care setting. Thus, MRIs are a reasonable, primary radiographic modality to diagnose osteomyelitis. PET and tagged white cell scans may be alternatives in circumstances where patients cannot receive an MRI, presuming that completing an empiric course of antimicrobial therapy is deemed a less acceptable option than establishing a definitive diagnosis of osteomyelitis.

<u>Biomarkers: ESR, CRP, Procalcitonin, Others</u>

Most studies evaluating accuracy of inflammatory biomarkers in the modern era have used MRI as the referent standard for identifying osteomyelitis patients. Many of these studies have reported limited accuracy of ESR and CRP at diagnosing osteomyelitis. Finally, given the accuracy of MRI, virtually no study has defined a role for inflammatory biomarkers to further improve the accuracy of diagnoses compared to MRI—i.e., how does the inflammatory biomarker add to diagnosis when an MRI is already done, or will be done irrespective of the biomarker result?

For example, in an observational study of 133 patients with vertebral osteomyelitis, Ghassibi et al. evaluated the impact of specific pathogens on ESR, CRP, and other biomarker values.[47] The reference standard for confirming osteomyelitis diagnosis was MRI. The mean ESR and CRP were substantially greater than the normal value cut-off, but did not achieve meaningful accuracy (e.g., likelihood ratios all <5). The mean white blood cell (WBC) count in peripheral blood was only 12,300 per microliter, slightly greater than normal. The mean percent neutrophil count was also unhelpful. Biomarker values were higher for pyogenic causes, and *S. aureus* in particular, than for culture-negative, fungal, or tuberculous (TB) osteomyelitis. Only *S. aureus* and streptococci caused mean WBC counts to rise above normal levels. CRP was normal in half of patients and remained unhelpful to dichotomize patients into those with or without osteomyelitis. WBC count was normal in 86% of patients with culture negative, and 100% of fungal osteomyelitis. A variety of methods were used to calculate receiver operating curves (ROCs) for each of these biomarkers. None led to meaningful biomarker cut-offs. Corroborating these results with MRI as the referent standard, Scharrenberg et al. reported that CRP levels had limited ability to distinguish vertebral osteomyelitis from non-infectious causes,

© 2022 Spellberg B et al. *JAMA Network Open.*

including erosive spinal degeneration (e.g., erosive osteochondrosis), particularly post-operatively.[83]

Similarly, in a study of 30 patients with suspected osteomyelitis, 15 subsequently were determined to have osteomyelitis of various body parts, and the remaining patients were ultimately diagnosed with arthritis, myositis, trauma, sarcoma, or other inflammatory disorders.[77] Temperature, WBC count, and ESR did not distinguish infectious from non-infectious, inflammatory bone disorders.

In a larger study of 163 patients with traumatic extremity osteomyelitis, ESR and CRP were again poorly accurate at diagnosing osteomyelitis.[48] Finally, these results are reinforced in a study of 102 patients with pedal osteomyelitis who did not have diabetes.[46] Using a combination of MRI and SPECT scanning, combined with bone culture and histopathology as the referent standards, the optimal cut points of ESR and CRP achieved sensitivity/specificities of only 49%/79% and 45%/71%, respectively.[46]

Thus, overall, studies have not found that typical inflammatory biomarkers are accurate in diagnosing osteomyelitis. None have demonstrated that they can be used to avoid imaging studies or enhance diagnostic accuracy compared to imaging studies.

<u>Blood Cultures</u>

Numerous observational studies have described a wide range of sensitivity of blood cultures for establishing the microbial etiology of osteomyelitis of numerous types. A 2016 review described blood culture positive rates of 40-89% across 11 studies of patients with vertebral osteomyelitis.[84] Similarly, more recent case series reported 60%[85] or 31%[86] blood culture positivity for vertebral osteomyelitis. While negative blood cultures are not helpful, positive blood cultures that identify a likely pathogen may obviate the need to proceed to more invasive microbiological testing (e.g., bone biopsy, discussed below). Furthermore, blood cultures are relatively non-invasive and inexpensive. They are therefore reasonable to obtain in patients with systemic signs of infection and a high suspicion for pyogenic osteomyelitis.

<u>Biopsy & Culture</u>

Several small observational studies have evaluated the diagnostic accuracy of bone histopathology and/or microbial culture for osteomyelitis.

A meta-analysis of 7 prior observational studies including 482 patients evaluated the overall diagnostic accuracy of imaging-guided biopsy to diagnose vertebral osteomyelitis.[49] The overall sensitivity and specificity of biopsy was 52% and >99%, respectively, resulting in a remarkably high positive likelihood ratio of 52, and a poor negative likelihood ratio of 0.5. Thus, positive biopsy histopathology is extremely accurate for ruling in the diagnosis of osteomyelitis, but a negative biopsy is very poor at ruling out osteomyelitis.

Histopathology can also help identify osteomyelitis caused by atypical pathogens (e.g., via granulomas, special stains such as AFB and silver stains, and even particular pathogen-specific findings, such as *Coccidioides* spherules). Furthermore, even a positive Gram stain on histopathology, in the absence of a positive culture, can enable targeted antibiotic de-escalation or escalation when appropriate.

Histopathology might have a higher sensitivity than culture. For example, in a study of 30 patients with suspected osteomyelitis at various body sites, the authors conducted what they referred to as "fine needle bone biopsies".[77] However, the needle size used for the biopsies was 11 gauge, and hence the biopsies performed might be more accurately described as core biopsies

using modern vernacular. Fifteen patients were ultimately diagnosed with osteomyelitis; the referent standard in this study was triple phase bone scan, radiographic changes on serial plain X-rays, or finding of pus on biopsy under the periosteum. Thirteen of 15 patients diagnosed with osteomyelitis had positive histopathology on biopsy (sensitivity 87%). Of the 15 patients who did not have osteomyelitis, histopathology was accurately negative in 14 (specificity 93%). Of note, 1 patient had a false positive diagnosis of osteomyelitis on histopathology due to severe acute and chronic inflammation with bone necrosis, in what was subsequently determined to be an Ewing's sarcoma lesion. Nevertheless, this study reinforced the potential superiority of histopathology over culture as means to establish a diagnosis of osteomyelitis. Similarly, in a study of 29 CT-guided bone biopsies, only 21% resulted in positive cultures.[87]

Additionally, in a study of 84 patients who underwent CT-guided vertebral bone biopsy, histopathology was positive in 41% of biopsy samples, whereas culture was only positive in 19%.[88] Furthermore, among control patients who were biopsied due to suspicion of non-infectious causes (primarily cancer), 77% of the biopsies established an alternative, non-infectious diagnosis by histopathology. This study reinforces that the benefits of histopathology lie in its ability to diagnose both infectious and alternate, non-infectious etiologies.

However, in other studies of 88, 46, 142, 111, and 64 patients with vertebral, other axial, or extremity osteomyelitis who underwent biopsy, the positivity rate of culture was higher at 60%, 36%, 43%, 36%, and 31%, respectively.[89-93] While histopathology may be more sensitive, and enable diagnosis of alternative diseases, the benefit of culture results is that they frequently enable more appropriate targeting of antimicrobial therapy.[93] Finally, the site of biopsy may be relevant, given that another study found that biopsies of paravertebral soft tissues resulted in a higher diagnostic yield than bone biopsy (68% vs. 38% for endplate-disk biopsies).[92]

Whether all patients in whom osteomyelitis is a concern should be subjected to bone biopsy cannot be established from the literature. Ultimately, the low yield of bone biopsies may suggest that they are not routinely required in all patients, particularly, as is true for any procedure, because there are procedural risks for the patient. For example, in a study of 78 patients with vertebral osteomyelitis who underwent bone biopsy, only 10 patients had positive histopathology, 14 had positive culture, and eight were positive both by histopathology and culture.[94] Only 19 of the biopsies altered treatment; 15 patients underwent de-escalation, and antimicrobial therapy was expanded in four cases.

Overall, the advantages of bone biopsy include confirming the diagnosis, evaluating for alternative diagnoses (such as malignancy), helping to identify atypical cases (e.g., TB, fungal), providing initial guidance to adjust antimicrobial therapy based on Gram stain, and enabling targeted therapy if cultures are positive. Disadvantages include that bone biopsies require considerable resources and may be difficult to obtain in many patient settings, the procedure is invasive with risks of harm to the patient, it may establish the diagnosis in half or less cases, and even histopathology can rarely lead to incorrect diagnoses.[77] Nonetheless, in patients for whom a microbiological diagnosis has not been otherwise achieved, it may be reasonable to pursue a biopsy in patients who are not felt to be adequately responding to appropriate empiric therapy in order to identify resistant, unusual, or non-bacterial pathogens and to exclude alternative diagnoses.

Thus, obtaining a bone biopsy must be individualized on a case-by-case basis and may rationally vary by practice setting and patient characteristics. It is also unclear if multiple biopsy specimens would increase or alter sensitivity or specificity diagnostically, and this is an area that warrants study in the future.

© 2022 Spellberg B et al. *JAMA Network Open.*

Special Methods and Molecular Diagnostics

Limited studies are available to evaluate novel molecular diagnostic methods for osteomyelitis without PJI. Choi et al. reported that among 45 patients with vertebral osteomyelitis who underwent a bone biopsy or aspirate, 21 patients had true positive 16S RNA PCR tests, identifying pathogens, and three patients had false positive PCR tests.[95] In contrast, culture was true positive in 12 patients and false positive in one. Other case reports have also used PCR to identify unusual pathogens in osteomyelitis without PJI, but only in small numbers and without controls.[96-98] Although data are not available to confirm accuracy, it may be rational to attempt such molecular diagnostic methods particularly for culture-negative infections when patients are clinically not responding to empiric therapy and this testing is available.

DFO

Probe-to-Bone (PTB) Test

A systematic review included seven studies with 1,025 patients and compared the PTB test to reference standards (primarily bone biopsy).[57] The PTB test had a pooled (95% CI) sensitivity of 87% (75%-93%) and specificity of 83% (65%-93%), resulting in reasonable positive and negative likelihood ratios of 5 and 0.2, respectively. In a patient with low to moderate pre-test probability, this accuracy may be sufficient to exclude DFO without requiring an MRI or other imaging studies (e.g., 33% pre-test probability shifts to <10% post-test probability with a negative probe-to-bone test).

Some experts have suggested that the combination of a negative plain X-ray and PTB test enhances sensitivity compared to PTB alone. However, there are very limited data assessing the accuracy of the combination vs. either test alone. One study of the combination of PTB and plain x-ray did find an impressive sensitivity and specificity of 97% and 92%, respectively in diagnosing DFO.[99] However, in that study, the sensitivity and specificity of both tests alone were also unusually high (95% and 93% for PTB and 82% and 93% for plain X-ray, respectively). Thus, combining PTB with plain X-ray did not appreciably enhance accuracy compared to PTB alone. Furthermore, the authors note that there was an unusually high pre-test probability of DFO in the patients because they were performed at a referral center for this disease.

In a second study, which also had a high pre-test probability of DFO, combining the PTB test with plain X-rays reduced sensitivity and specificity compared to PTB alone. Specifically, Morales Lozano et al. evaluated the sensitivity and specificity of the PTB test with or without plain X-rays in a prospective study of patients with diabetic foot ulcers.[100] The PTB test had an impressive 98% sensitivity and 78% specificity for DFO. However, combining the PTB test with plain X-rays lowered the sensitivity to 89% and lowered the specificity to 77%. Thus, data appear insufficient to support use of combination plain X-rays and PTB test for DFO and suggest that PTB may perform adequately by itself. Further study is needed.

Imaging Studies (Table 1)

In a comprehensive systematic review of the accuracy of imaging studies specifically for the diagnosis of DFO, Llewellyn et al. determined that the pooled (95% CI) sensitivity and specificity of plain X-rays was only 62% (51%-72%) and 78% (63%-89%).[50] These results

yielded positive and negative likelihood ratios of 2.8 and 0.5, respectively.[50] Thus, the accuracy of plain X-rays for diagnosing DFO is poor, and they are of low value for this purpose. Nuclear medicine studies were more sensitive but less specific, resulting in poor overall accuracies. Specifically, the pooled (95% CI) sensitivity and specificity of various forms of scintigraphy (excluding tagged white cell scans) were only 85% (77%-90%) and 68% (56%-77%), resulting in positive and negative likelihood ratios of 2.7 and 0.2, respectively.

PET scans are more accurate, although they are also more resource intensive and less available. They also expose the patient to considerable ionizing radiation. Llewellyn et al. found their pooled (95% CI) sensitivity and specificity to be 84% (53%-96%) and 93% (76%-98%), respectively for DFO.[50] Based on only three studies, SPECT had superior sensitivity but much inferior specificity at 96% (76%-99%) and 55% (19%-86%), respectively. In a smaller systematic review of six studies of PET scans for diagnosing DFO, the sensitivity of the test ranged from 75% to 90% and specificity ranged from 75% to 98% in individual studies.[50] Similarly, Lauri et al. systematically reviewed the literature for diagnostic accuracy of PET scans and tagged white cell scans for DFO.[51] They found that PET scan sensitivity/specificity were 89% and 92%, respectively, and tagged white cell scan sensitivity and specificity were 91-92% and 75-92%, respectively, depending on the nature of the white cell label.

Overall, MRIs remain the most accurate routinely available imaging test for DFO while avoiding ionizing radiation. Llewellyn et al. reported a pooled sensitivity and specificity of 96% and 84%, respectively, resulting in positive and negative likelihood ratios of 6 and 0.05, respectively.[50] In their review, Lauri et al. reported a similar sensitivity and specificity of MRI for DFO at 93% and 75%, respectively.[51] Finally, in a third systematic review of 36 studies evaluating various imaging studies for diagnosing DFO, MRI was the most accurate study overall, and the authors reaffirmed its role as the preferred diagnostic modality due to accuracy and the avoidance of ionizing radiation.[50]

Inflammatory Biomarkers

Xu et al. evaluated a combination of PTB test plus inflammatory biomarkers for diagnosing 111 cases of DFO from among 204 patients with diabetic foot infections (DFI).[52] The referent standard for diagnosis was a positive bone biopsy. The authors found that WBC count, percent neutrophils in peripheral blood, and CRP were all inaccurate at distinguishing DFO from non-osteomyelitis cases. While ESR was the most accurate biomarker, it achieved an 83% sensitivity and 71% specificity at its best cut-point, which would not substantively alter post-test probabilities in individual patients unless the pre-test probability was already quite low (a 15% pre-test probability would become a 4% post-test probability).[52]

Moallemi et al. also found limited accuracy (sensitivity and specificities of 60%-70%, positive and negative likelihood ratios 2 and >0.5, respectively) for ESR and CRP in diagnosing DFO.[53]

Lavery et al. evaluated 353 patients with DFI, of which 176 had DFO.[54] Of note, they excluded patients with comorbid conditions that could have falsely elevated ESR and CRP results in an attempt to optimize testing accuracy. The referent standard was MRI or SPECT scan and bone biopsy. The investigators evaluated multiple cut-points of ESR and CRP and found none that yielded good discriminatory results. At their optimal cut points, sensitivity, and specificity of ESR were only 74% (95% CI, 67%-80%) and 56% (95% CI, 48%-63%), respectively; sensitivity and specificity of CRP were only 49% (95% CI, 41%-57%) and 80% (95% CI, 74%-86%), respectively. Especially given that these accuracies are inflated by

© 2022 Spellberg B et al. *JAMA Network Open.*

exclusion of patients with comorbidities associated with inflammation, these results cast considerable doubt on the utility of ESR and CRP as a diagnostic tool for DFO.

Finally, a study of 90 patients determined that procalcitonin had greater accuracy than ESR or CRP at diagnosing DFO, with MRI again the referent standard.[101] However, the overall levels of procalcitonin (PCT) were 0.13 +/- 0.02 ng/ml in patients with osteomyelitis, whereas typically levels of >0.5 are used to distinguish the need for continuation of antibiotics. Hence, the procalcitonin levels were quite low, and within the range that would not typically be used to support antibacterial therapy.

Several systematic reviews have also sought to define the accuracy of inflammatory biomarkers at diagnosing osteomyelitis, with conflicting findings. Victoria van Asten et al. reviewed eight studies of patients with DFO and found that only ESR was accurate at diagnosing osteomyelitis; CRP, procalcitonin, and various inflammatory cytokines (e.g., interleukins IL-2, IL-6, IL-8, and tumor necrosis factor (TNF)) were not.[55] They reported a higher sensitivity and specificity of ESR in this setting than other studies cited above, with sensitivity and specificity of 81% (95% CI, 71%-88%) and 90% (95% CI, 75%-96%), respectively. In contrast, in their review of the literature, Markanday found lower accuracies, reporting sensitivities and specificities for both ESR and CRP in the 70-80% range for DFO.[56]

Percutaneous Bone Biopsy (PBB)

PBB may be useful to help make the diagnosis of DFO and guide antimicrobial therapy when surgical intervention is not planned. An 11-study systematic review of patients with DFO found that the pooled proportion of culture-positive PBB was 84% (95% CI, 73%-91%).[102] There was extensive heterogeneity, however after excluding two studies with very high proportions of positive culture results, the pooled proportion of culture positive PBBs was more conservatively estimated at 77% (95% CI, 68%-85%).

While culture yields may be relatively higher for patients with suspected DFO compared to other sites of osteomyelitis,[103] the systematic review highlighted limitations that should be considered. First, studies seldom reported the technical aspects of biopsied procedures (e.g., needle gauge size). Second, ulcer severity scores were under-reported. Third, only one study provided methods for identifying or defining contaminants. Finally, concerns have been raised about lower culture yields when received antibiotics prior to the biopsy occurring. The relative timing of antibiotic exposure to biopsy is discussed in Section 2.

As for biopsies for osteomyelitis outside the context of DFO (see above), PBB specimens tend to have higher rates of positive histopathology than cultures. For example, Tardaguila-Garcia et al. compared the sensitivity and specificity of bone histopathology and culture in 52 patients for whom clinicians had a suspicion for DFO.[104] A limitation of this study was that no specific referent standard was used to identify confirmed osteomyelitis. Suspicion was based on a positive PTB test with serial plain films. Biopsies were obtained after cessation of antibiotics for 48-72 hours, and then after surgical debridement of surface materials. Thirty-six patients had a positive microbial culture from the bone biopsy, compared to 47 patients who had histopathological evidence of osteomyelitis. Thus, cultures were less sensitive than histopathology, but it is not possible to determine the precise accuracy of histopathology from a focal biopsy in the absence of a referent standard.

Furthermore, there may be considerable variation between pathologists when diagnosing osteomyelitis. In one study of four pathologists independently reviewing 39 cases of suspected osteomyelitis, the kappa coefficient for concordance was only 0.3 (1/3 correspondence rate),

which is indicative of only fair agreement.[105] Consistency may be improved when pathologists use a standardized framework for diagnosis.[106]

Similarly, concordance between surface swabs and deep bone biopsies are poor and underscore the lack of utility of surface swabs for diagnosing DFO. For example, Senneville et al. reported the results of bone biopsy from 76 patients with DFO.[107] The concordance of surface swab culture compared with a culture of a percutaneous bone biopsy specimen was only 23%. They subsequently published a review of two other studies comparing superficial swabs to bone or deep tissue cultures in which concordance was only 19% and 38%, respectively.[108] The investigators concluded that superficial swab cultures should not be performed. Similarly, concordance between surface swabs and deep bone biopsies are poor and underscore the lack of utility of surface swabs for diagnosing DFO.

Finally, non-culture-based molecular diagnostics are promising but cannot be routinely recommended as part of a biopsy panel at this time. A study by Malone et al. evaluating peptide nucleic acid fluorescence in situ hybridization (PNA FISH) of proximal tissue margins of surgical resections found 8/14 (57%) specimens without growth had a positive result.[109] Despite finding bacteria in proximal clean margins, there was no data suggesting worse outcomes. Also, contamination at the time of specimen collection or during subsequent handling could lead to false positive results. Time to results, cost, and interpretations are barriers to using these newer diagnostics.

As for other types of osteomyelitis, the number of biopsies to optimize diagnostic sensitivity and specificity is of interest for the future study.

Osteomyelitis with PJI

Overview

There is no uniformly accepted diagnostic criteria for PJI. There have been multiple attempts to develop diagnostic criteria for PJI, including the Musculoskeletal Infection Society (MSIS) initial definition in 2011, followed by the modified International Consensus on Musculoskeletal Infection (ICM) criteria initially in 2013, revised in 2018, and more recently, the European Bone and Joint Infection Society (EBJIS), now endorsed by MSIS as well.[110-114] These definitions are based on clinical characteristics, blood biomarkers, synovial fluid studies, microbiology tests, and histology results with various cut off values to define PJI. In these various classifications, there are a few criteria that, if present alone, are proposed to confirm the presence of PJI. Combinations of findings and various cutoff values are considered for probable infection or to rule out infection. Furthermore, in the absence of a referent standard for diagnosis, it is difficult to determine the true accuracy of any of the proposed schema for diagnosing PJI.

Additional caveats include potential variation in accuracy of diagnostic lab criteria based on the timing of the laboratory studies relative to surgery, because the diagnostic lab criteria can be influenced by the post-operative state itself or variations in surgical management. Furthermore, while PJI of the hips and knees are the most common and widely studied, the utility of the diagnostic criteria may differ among other arthroplasties.

Clinical Signs

PJIs can present in many ways, ranging from asymptomatic loosening of the joint, to fever, joint redness, and systemic sepsis. However, the presence of a sinus tract directly communicating

with the joint, or external visualization of the prosthesis, is considered by diagnostic algorithms to be definitive for the diagnosis of PJI. Aside from intraoperative cultures obtained for the purpose of guiding antimicrobial therapy, further diagnostic studies are not considered necessary.[110-113,115,116] We emphasize that there are no good data to establish the accuracy of the presence or absence of a sinus tract, nor to validate it as a referent standard for diagnosis. However, it is a commonly accepted standard in clinical practice.

Imaging Studies

Published sensitivity and specificity of individual studies for diagnosing PJI (Table 1) should be considered cautiously given the absence of an optimal referent standard. Thus, these numbers are uncertain estimates, which may also account for the wide variations in reported sensitivity and specificity.

Several reviews have concluded that WBC scintigraphy and MRIs may have the best overall accuracy of the various radiologic techniques for detecting PJI.[58,117] Plain X-rays are not accurate for the diagnosis, with sensitivity and specificities reportedly as low as 14% and 70%, respectively.[58] X-rays may be indicated, however, in the evaluation of a painful prosthetic knee joint to exclude non-infectious pathology and hardware complications. CT scans may have superior accuracy, although data are limited, and the scatter from the prosthetic material may affect interpretability.

Nuclear medicine scintigraphy and tagged white cell scans have had sensitivities and specificities for PJI ranging from 69%-94% across numerous studies.[58] Tagged white cell scans had the highest accuracy among nuclear medicine studies, with sensitivities and specificities >90%.[58,64,65] PET and SPECT scans may have promise in diagnosing PJI; however, the data are limited and somewhat mixed. In one study of 130 patients with painful prosthetic hips, PET scan was 95% sensitive but only 39% specific for detecting PJI.[66] Plate et al. reported a sensitivity of 78% and specificity of 94% for SPECT for diagnosing osteomyelitis, including PJI among 26 cases.[118] In contrast, Wenter et al. reported a sensitivity of 86% but a specificity of only 67% for diagnosing 101 cases of PJI among 215 patients with prosthetic complications.[119]

In a systematic review of 13 studies evaluating the accuracy of various diagnostic tests for PJI, PET scans had a sensitivity and specificity ranging from 80%-90% each.[120] In another systematic review of 11 studies, PET scans had a sensitivity and specificity of 82% and 87%, respectively, but with statistical heterogeneity between the included studies.

In a review of four studies of MRI for PJI, the sensitivity ranged from 65%-92% and specificity ranged from 85%-99% for knee PJI, and while sensitivity and specificity were 94% and 97% for hip PJI.[58] Other studies have been concordant, with sensitivities of 78%-86% and a specificity of 73%-90%.[59,60]

Inflammatory Biomarkers

Ahmad et al. conducted a meta-analysis from 278 clinical studies comprising 27,754 patients with PJI and found that the pooled sensitivity and specificity for diagnosing PJI was only 75% and 70% for ESR and 88% and 74% for CRP.[120] IL-6 accuracy was higher, with a sensitivity and specificity of 97% and 91%. However, IL-6 levels are not available in most hospital laboratories. Finally, Berbari et al. conducted a meta-analysis of 30 studies (n = 1,270 patients with PJI) and reported pooled sensitivity and specificities of 75% and 87% for ESR and 97% and 74% for CRP.[69] In other studies of PJI, CRP has been well described to be falsely negative, particularly for patients with more indolent pathogens.[70]

© 2022 Spellberg B et al. *JAMA Network Open.*

D-dimer, a fibrin degradation product, has recently been evaluated as a potential biomarker for PJI. For example, a single-center prospective study of 245 patients total undergoing primary or revision arthroplasty for aseptic or septic failure were included, and all had D-dimer, ESR, and CRP drawn pre-operatively.[121] Using a cutoff of 850 ng/ml, D-dimer was found to have a sensitivity of 89% and a specificity of 93%, significantly higher than either ESR or CRP in this study. While this biomarker may be promising, the fact that D-dimer is known to be elevated in many conditions, including various types of thrombosis and hematoma, raises concerns about the generalizability of this single report. Further studies are needed to determine the validity of these findings.

Synovial Fluid Studies

Included in all of the major PJI algorithmic definitions are synovial fluid WBC count, and percentage of polymorphonuclear cells (PMNs) with varying diagnostic cut-offs proposed, based on numerous observational studies.[110-114] It is important to note that the various synovial WBC cutoffs for PJI are much lower than those for native septic arthritis.[122] Additionally, the synovial fluid WBC count and differential have been shown to change over time, with synovial fluid WBC count and neutrophil percentages significantly elevated early in the post-operative period, which may lead to false positive results depending on the cutoff used.[123] Qu et al. performed a meta-analysis including 15 studies and 2,787 patients and found pooled sensitivity/specificity for diagnosing PJI using synovial fluid WBC count was 88%/93%, and for synovial fluid PMN% the sensitivity/specificity were 90%/88%, respectively.[71]

At the time of arthrocentesis, synovial fluid is often sent for microbiologic culture. There is significant heterogeneity in culture techniques across institutions, microbiology labs, and internationally, and thus the diagnostic utility of culture can vary. However, in a meta-analysis by Lee et al. of five studies including 509 patients evaluating the utility of culture in diagnostic arthrocentesis, the sensitivity was found to be poor at 62%, but the specificity was high at 94%.[72] It is unknown whether direct inoculation into blood culture bottles might improve diagnostic yield as it does for other sterile sites.

Synovial Fluid Leukocyte Esterase (LE)

LE is an enzyme secreted by activated neutrophils at the site of an infection and can be detected on a colorimetric test strip similar to that used in the detection of urinary tract infections, with the advantage of being inexpensive and providing real-time results if used in a point-of-care fashion.[124] Li et al. recently published an updated meta-analysis evaluating the diagnostic accuracy of LE for PJI.[125] They identified 17 studies involving a total of 1,963 patients (including 571 PJIs) and obtained a pooled sensitivity and specificity of 90% and 96%, respectively, for diagnosing PJI. A small study of 61 patients demonstrated the test retained its specificity even in the setting of adverse local tissue reactions seen after metal-on-metal total hip arthroplasty, which is known to potentially generate purulent synovial fluid.[126] One caveat is that the test is invalidated by blood contamination in synovial fluid without centrifugation prior to use.[115] It has been included in the recent MSIS and ICM definitions for PJI.

Other Synovial Fluid Biomarkers

Alpha defensin is a small peptide that is also secreted by activated neutrophils in the setting of infection, exhibiting antimicrobial effects against a spectrum of pathogens.[115] In the same meta-analysis mentioned above by Li et al., the diagnostic validity of alpha defensin in PJI was

© 2022 Spellberg B et al. *JAMA Network Open.*

examined.[125] The review identified 21 studies with a total of 1,928 patients (and 650 PJIs), of which eight studies used a lateral flow assay, 12 studies used a laboratory-based immunoassay, and one study did not report on the testing methods. The pooled sensitivity and specificity of alpha defensin for diagnosing PJI were 89% and 96%, though significant heterogeneity was observed between samples due to differences in patient sample size and method of detection. False positives can occur in metallosis or in acute gout.[127] This test is included in the updated MSIS and ICM criteria for PJI, though its use in arthroplasty at sites other than the hip or knee, or its cost effectiveness are not yet known.[115]

While serum and synovial fluid levels of CRP have been shown to correlate, a recent meta-analysis by Wang et al. included six studies comprising a total of 456 participants, and found a pooled sensitivity and specificity of 92% and 90% of synovial fluid CRP for PJI, which was superior to serum CRP.[128] However, there was heterogeneity in the platforms and cutoff values used, and larger studies are needed to confirm the utility prior to its implementation routinely in the diagnosis of PJI. Similarly, synovial fluid IL-6 has been to be more specific than serum IL-6 levels. A meta-analysis of 17 studies describing PJI diagnosis using serum and synovial fluid IL-6 demonstrated that synovial fluid IL-6 had a sensitivity and specificity of 91% and 90%, which was notable for a higher sensitivity than serum IL-6 and comparable specificity.[129] IL-6 is likely not available for use routinely in most clinical laboratories, but may be in the future if further studies evaluate the optimal cutoff for use. While many of these synovial fluid biomarkers show promise, it is not yet known whether they improve the diagnosis of PJI compared to more conventional tests such as synovial WBC count, PMN%, and histopathology, or whether they will prove to be cost-effective.

<u>Intraoperative Testing: Histopathology</u>

Histologic exam of intraoperative frozen section to assess for acute inflammation is another diagnostic tool used by surgeons, particularly when pre-operative results are equivocal for PJI, or at time of revision surgery to avoid implanting a new joint into an infected site. In 2013, Tsaras et al. performed a systematic review and meta-analysis of studies comparing the performance of frozen section histology to simultaneously obtained microbiologic culture at the time of revision hip or knee arthroplasty.[116] The review of 26 studies, including 3,269 patients of which 796 (24.3%) had a culture-positive PJI, found that the positive likelihood ratio was an impressive 12.0 for ruling in PJI, whereas the negative likelihood ratio was a less impressive 0.23. They reported no difference when comparing studies using thresholds of five vs. ten PMNs per high-power field. There was significant heterogeneity among pooled studies, which is at least partially reflective of the highly operator-dependent nature of frozen section sample preparation and interpretation. Another meta-analysis by Zhao et al. also found no statistical difference in the diagnostic odds ratio when comparing a cutoff threshold of five vs. ten PMNs per high-power field.[130] Thus, data suggest that a diagnostic threshold of either five or ten PMNs per high-power field in each of five high-power fields can help diagnose or rule out PJI at the time of revision arthroplasty. It is unknown whether these thresholds apply to joints other than the hip or knee, or the performance other than at the time of revision arthroplasty.[131]

Additionally, lower virulence organisms, such as *Cutibacterium acnes* (formerly *Propionibacterium acnes*), may fail to induce a neutrophil response or acute inflammation, and consequently, the sensitivity of this method in these situations is likely lower. However, neutrophilic infiltrates and, thus false positive results, can also be seen in the setting of periprosthetic fracture or inflammatory arthritis in the absence of infection.[115]

© 2022 Spellberg B et al. *JAMA Network Open.*

Culture and Gram Stain

A prospective study of 117 patients who underwent revision hip or knee arthroplasties, performed for septic or aseptic reasons, compared the performance of tissue cultures vs. swabs in diagnosing PJI. The study reported a higher accuracy for tissue cultures relative to swab cultures (sensitivity 93% vs. 70%, and specificity 98% vs. 89%, respectively).[132] In a prospective study by Atkins et al. evaluating 297 patients who underwent revision hip or knee replacement at a single institution, three or more positive cultures were reported to have a sensitivity of 66% and specificity of 99.6% when compared to the presence of acute inflammatory cells in specimens examined histologically.[133] Through the use of mathematical modeling, they suggested that obtaining five or six intraoperative tissue specimens for culture would result in a sensitivity of >80% and a specificity of >90% for detecting PJI with two or more specimens positive for the same organism. They also found that Gram stains had a very low sensitivity of only 6%, though with a specificity of >99%. Thus, negative Gram stains or culture results are not recommended to be used to rule out PJI.

Special Methods and Molecular Diagnostics

Few observational studies have assessed the role of special methods and molecular diagnostics for PJI. Sonication of device material removed or debrided during PJI surgical management may be used in microbiology laboratories to culture etiologic pathogens.[134] Stephan et al. evaluated 90 patients with PJI to determine if prior antibiotics affected the yield of sonication-based culture methods.[135] They found that cultures were positive in 86%, 81%, and 87% of patients who received peri-operative antibiotic prophylaxis, therapeutic antibiotics for ≥1 day prior to surgery, or no antibiotics prior to surgery, respectively. Thus, they reported no impact of prior antibiotics on sonication-based culture yield for PJIs. A more recent method to liberate bacteria from beneath biofilms in lieu of sonication involves addition of dithiothreitol to the prosthetic material.[134] That method is less established than sonication but may result in similar diagnostic yield, which may be higher than culture results without sonication.[134] Indeed, either sonication and addition of dithiothreitol has been shown to result in higher positive culture rates compared to cultures without these biofilm disruption methods.[120,136-139]

However, studies are not uniform, and some have indicated that sonication does not increase culture yield compared to adequate culture of periprosthetic tissue.[140,141] Furthermore, there is extra cost and technician time required for sonication and dithiothreitol methods, and this extra cost and time may or may not meet cost-effectiveness thresholds in various clinical settings.[142]

A recent systematic review of more modern concepts discussed PCR, sequencing, and metagenomics methods for establishing the microbial etiology of PJI.[134] In individual studies, molecular diagnostics have been able to achieve higher rates of microbial identification than traditional culture. As reviewed,[134] some studies have reported that multiplex PCR and next generation metagenomic sequencing not only to have superior sensitivity and high specificity compared to traditional culture methods, they were also faster than traditional microbiological methods.[143-147] However, the data are mixed, as multiple other studies have found that traditional culture performed similarly to molecular methods.[134,141,148-154]

In a meta-analysis, the sensitivity and specificity of 16s RNA PCR was pooled across 15 observational studies of patients with PJI.[155] The pooled (95% CI) sensitivity and specificity were 70% (67%-73%) and 93% (91%-94%). Sonication of the culture material before

application of 16s RNA slightly increased accuracy, with a sensitivity and specificity of 76% and 93%, respectively. In a second meta-analysis of 12 studies of 16s RNA PCR, the pooled (95% CI) sensitivity was 81% (73%-87%) and specificity was 94% (94%-97%).[156] Antecedent antibiotics reduced the sensitivity of the PCR assay (71% vs. 94%). Furthermore, the study found that sensitivity varied based on the method used, with Illumina sequencing achieving higher specificity than other methods (96% vs. 83%). In a third meta-analysis of nine studies of sonication plus PCR, pooled sensitivities and specificities were 75% (95% CI, 71%-81%) and 96% (95% CI, 94%-97%).[157] Thus, the sensitivity and specificity of such molecular methods for establishing the microbial etiology of PJI appears to be approximately 70%-75% and 90%-95%, respectively.

The primary advantage of broader, non-biased molecular sequencing methods may be to identify unusual or fastidious organisms that are difficult to culture by traditional methods.[158,159] However, a complication of these results is that it can be difficult to determine if the detected organism is an etiologic pathogen, and no reference standard is available to clarify this issue.

© 2022 Spellberg B et al. *JAMA Network Open.*

Question 2: What is the appropriate management for osteomyelitis underlying a pressure ulcer?

Executive Summary:
No RCTs and only a limited number of observational studies have evaluated the optimal management of osteomyelitis underlying a pressure ulcer. Deep bone biopsies often demonstrate fibrotic bony remodeling, without histopathological evidence of osteomyelitis, even in bone exposed for months to years. Unfortunately, biomarkers (e.g., ESR, CRP), imaging studies (e.g., X-rays, CT scans, nuclear medicine, MRI), and surface cultures are not accurate at diagnosing osteomyelitis underlying a pressure ulcer and we do not recommend routinely obtaining such studies. Histopathology of deep bone biopsy is the referent standard for diagnosis. However, there is no evidence that it is important to make a diagnosis of osteomyelitis underlying a pressure ulcer unless there is a plan to surgically close the wound, as studies have not identified a therapeutic benefit, and suggest harm, of antibiotics in the absence of surgical wound closure. If there is no plan to surgically close the wound, therefore, routinely obtaining a bone biopsy and/or administering antibiotic therapy due to concerns of osteomyelitis is unlikely to be of benefit, and may be harmful.

If there is a plan to surgically close the wound, a reasonable overall multidisciplinary management plan includes: nutritional optimization; local wound care via specialists (e.g., advanced practice nursing); debridement to remove necrotic material; bone biopsy to determine the histologic and microbiologic diagnosis of osteomyelitis or not; only if osteomyelitis is present, administration of antimicrobial therapy targeted by the deep bone biopsy (as underlying osteomyelitis may increase the risk of flap failure); surgical wound closure; pressure offloading; and addressing the psychosocial drivers leading to wound development and resulting in an increased risk of flap failure (e.g., malnutrition, smoking). In the absence of planned surgical wound closure, the multidisciplinary plan should remain the same, except for the lack of need for bone biopsy and antibiotic administration. When antimicrobials are administered, no data exist to guide selection of IV vs. oral administration or to support durations of therapy beyond 2 to 6 weeks. We emphasize that short durations (e.g., ≤1 week) of antibiotics are reasonable to treat acute soft tissue infections around a pressure ulcer, or acute sepsis syndrome, as opposed to osteomyelitis underlying the ulcer.

Overall Summary:

No RCTs have been published that define optimal diagnostic or management strategies for osteomyelitis underlying a pressure ulcer.[20,21] However, observational studies of various designs, sizes, and quality have been published that evaluate aspects of the disease.

Diagnosis of Osteomyelitis Underlying a Pressure Ulcer

<u>Histopathological analysis and bone culture</u>
Bone biopsy for histopathologic analysis remains the gold standard for confirming the diagnosis of osteomyelitis. Surprisingly, despite classical teaching that exposed bone signifies osteomyelitis is present by definition, when exposed bone has been biopsied in published studies, osteomyelitis was present on histopathology in fewer than half of cases.[20,21,160] Rather, in many

© 2022 Spellberg B et al. *JAMA Network Open.*

instances, what was identified on biopsy was fibrotic bony remodeling with medullary edema, which may be indistinguishable from osteomyelitis on imaging studies.[160-162]

For example, Türk et al. examined histologic autopsy specimens of 28 patients with advanced-grade pressure ulcers, specifically those with visible bone.[160] In 15 cases, osteomyelitis was not detectable histologically. In the remaining 13 cases with osteomyelitis, disease was generally focal and superficial. Osteomyelitis has been described to be absent frequently even in bone exposed for months to years, and no correlation has been found between the duration of bone exposure and the risk of osteomyelitis.[160-162] As bony remodeling rather than osteomyelitis was described the majority of instances of exposed bone,[160-162] perhaps it is not surprising that alternative methods of diagnostic testing have been found to be highly inaccurate at detecting osteomyelitis underlying pressure ulcers.[20]

Skin and open ulcers are invariably colonized by bacteria, so superficial cultures are expected to yield bacterial growth. Thus, wound swabs or cultures taken from superficially debrided material are not helpful in establishing diagnosis, as organisms recovered are often colonizers and not true pathogens.[163,164] Indeed, surface cultures are inaccurate at determining whether infection is present in deeper bone, or, if present, at predicting which bacteria are etiologic for osteomyelitis in deeper bone.[163,164] One systematic review of four studies compared the accuracy of microbial culture to bone histopathology for the diagnosis of osteomyelitis underlying pelvic pressure ulcers.[164] In the four studies, tissue and bone cultures were reported, with the latter obtained by percutaneous or surgical bone biopsy.[161,162,165,166] However, in three of the studies surface material was not debrided prior to the needle biopsy of bone;[161,162,165] in the remaining study, surface material was debrided before a surgical biopsy of bone was obtained.[166] The diagnostic performance of microbial culture varied widely across the studies, with sensitivities of 18% to 100% and specificities of 43% to 100%, likely reflecting different definitions of commensal vs. pathological organisms and/or different methodologies of obtaining cultures.

Thus, rather than having diagnostic utility, the primary purpose of bone cultures is to guide antibiotic selection for therapeutic purposes. As such, we do not recommend routinely obtaining bone cultures unless they are from a deep bone biopsy that confirms osteomyelitis and there is a plan to definitively treat the infection with culture-driven antibiotics (see below). If bone cultures are obtained, it is logical to collect them after debriding surface material to decrease the burden of confounding bacterial colonizers.

Blood biomarkers and acute phase reactants

Soft tissue edema around exposed bone can trigger low level inflammation, and one study found typical biomarkers, such as leukocytosis, ESR, and CRP, to be neither sensitive nor specific for distinguishing the presence or absence of osteomyelitis underneath pressure ulcers.[165] In the absence of any studies indicating that these tests are accurate for diagnosing osteomyelitis underlying pressure ulcers (as for other types of osteomyelitis, see Section 1), we do not recommend routinely ordering biomarkers for this purpose.

Imaging studies

Unfortunately, imaging studies are also not accurate for diagnosing osteomyelitis beneath a pressure ulcer, likely because they cannot distinguish bony remodeling from infectious osteomyelitis.[20,21,164,167] Specifically, plain X-rays, CT scans, and nuclear medicine studies had sensitivities of 60% or less when compared to bone biopsies in observational studies.[164,168-170]

Specificities of these tests varied widely in published studies (ranging from 11% to 100%). While specificities were higher in studies comparing these tests to clinical diagnosis,[164] clinical diagnosis is known to be inaccurate,[164] and hence specificities of imaging tests compared to anything other than bone biopsy are difficult to interpret. The finding of destroyed bone on imaging is likely more specific for osteomyelitis, but is a very late finding, and thus uncommon, which may also account for variations in the specificity in the published observational studies.

Given these limitations, we do not recommend routinely obtaining such imaging modalities for diagnosing osteomyelitis underlying pressure ulcers. If bony destruction is incidentally observed on plain radiography or CT scan obtained for other reasons, it may indeed strongly suggest the presence of osteomyelitis. Such information should then be incorporated into an overall management strategy dependent primarily on whether there is a plan to surgically close the wound (see below).

In several studies, MRI had a sensitivity in excess of 80%-90% for detecting osteomyelitis underlying pressure ulcers.[166,167,171] However, its specificity was very poor (17%-22%) compared to the reference standard of bone biopsy.[164,166,167] Again, this poor specificity is likely due to inability of MRI to distinguish osteomyelitis from the reactive remodeling that occurs in exposed bone. Therefore, despite having a relatively high sensitivity overall, due to poor specificity, MRIs have low positive and negative likelihood ratios (≤2), which are not adequate to meaningfully shift pre-test probability of osteomyelitis underlying a pressure ulcer in most patients.

Summary of diagnostic approach

A critical point is that the diagnosis of osteomyelitis underlying a pressure ulcer is only important to make if such a diagnosis will alter the management plan for the patient.[20,21] As discussed below, absent the intent to surgically debride and definitely close an open wound, it is not clear that treatment of osteomyelitis with antibiotics improves the long-term outcomes of pressure ulcers (but evidence suggests antibiotic therapy may perversely worsen outcomes without closing the wound). A standard wound care and debridement management plan should be implemented irrespective of the presence of osteomyelitis. Thus, it may be futile to order diagnostic testing outside the setting of a plan to close the wound, as detecting osteomyelitis (even if one could accurately do so) would not alter the clinical management plan.

In summary, based on the limited retrospective data available, we do not recommend the routine use of biomarkers (including WBC count, ESR, CRP) or X-rays, CT or nuclear medicine imaging, or surface cultures to diagnose osteomyelitis underlying pressure ulcers. MRIs may only be useful in planning for definitive surgical debridement and wound closure as part of a comprehensive management plan, as delineated below. While it may seem as though a negative MRI could suggest a lack of osteomyelitis, obviating the need for more invasive testing, with a negative likelihood ratio of ≤2, a negative MRI shifts the published pre-test probability of approximately 50% of osteomyelitis for those with stage-4 pressure ulcers to a post-test probability of approximately 33%, which is inadequate to exclude the diagnosis. Even with a pre-test probability of only 25%, a negative MRI would shift the post-test probability to 17%, still inadequate to exclude the diagnosis.

The only diagnostic test that is of demonstrated value for osteomyelitis underlying a pressure ulcer is bone biopsy. Furthermore, in the absence of a plan to provide definitive wound closure, we do not recommend a bone biopsy to establish a diagnosis of osteomyelitis, as it is not clear how such a diagnosis would alter management. In contrast, if there is an intent to

administer definitive therapy, including surgical wound closure, it is reasonable to consider surgical debridement of the wound, enabling deep bone biopsy for histopathology as the primary diagnostic modality to detect osteomyelitis. In this case, bone biopsy cultures should also be sent to enable targeting of antimicrobial therapy as part of the comprehensive management plan.

Treatment of Osteomyelitis Underlying a Pressure Ulcer

Two systematic reviews of pressure-ulcer associated osteomyelitis, published in infectious diseases and orthopedic surgery journals, failed to identify any literature demonstrating a therapeutic benefit of antibiotics alone for osteomyelitis underlying a pressure ulcer; antibiotics had a positive effect only when administered in conjunction with definitive interventions to debride and close the wound.[20,21] Antimicrobial agents may be rationally administered to treat an acute soft tissue infection around the wound, and in this case should be administered for only a brief period of time (e.g., ≤ 1 week) to treat an acute bacterial skin and/or skin structure infection, or sepsis syndrome, rather than an osteomyelitis. Exposure of patients to antibiotics for longer durations without surgical debridement and wound closure could increase their risk of harm (e.g., adverse events), as well as promote the emergence of antibiotic-resistant pathogens that colonize the wound, creating risk for future antibiotic-resistant super-infections.[20]

Indeed, in multiple observational studies of both adults and children, infectious or wound healing outcomes were not influenced by antibiotic administration (including route or duration) or the presence of osteomyelitis, particularly without surgical wound closure.[162,163,168,172-176] However, prolonged antibiotic administration (e.g., 6 weeks), and failure to address pressure off-loading, were associated with increased harm, including more frequent wound breakdown, ulcer recurrence, and longer hospitalization.[172,175,176]

Conversely, multiple observational studies of both adults and children have found substantially better wound and flap healing outcomes of osteomyelitis underlying a pressure ulcer when managed via a comprehensive plan including medical, psychosocial, and surgical approaches.[163,168,172,175-179] Such plans have included surgical debridement to the level of healthy, viable tissue, obtaining deep cultures intraoperatively to guide subsequent antimicrobial therapy, definitive surgical wound closure, and psychosocial/behavioral interventions to alter pressure dynamics to prevent reopening of the wound and/or failure of the flap.[20,21] Of note, in one of the largest observational studies of wound closure by flap (n = 276 patients), multivariate analysis found that low body mass index (likely reflective of malnutrition), smoking, ischial pressure ulcers (vs. other body sites), and presence of osteomyelitis in the wound before flapping predicted wound dehiscence and/or flap failure/ulcer recurrence.[179] For pressure ulcers near the perineum or in locations frequently contaminated by stool, small observational studies have shown that performing an elective colostomy for fecal diversion is associated with decreased ulcer recurrence rate and need for subsequent operations, leading to an improved patient quality of life.[180,181] Thus, risk factors should be considered when optimizing patients for definitive surgical wound closure.

Barring surgical wound closure, no data demonstrate that the presence of osteomyelitis underlying a pressure ulcer predicts a change in likelihood of ulcer healing or recurrence. Even the finding of a positive intraoperative bone culture did not alter the future risk of flap failure in one observational study.[167] Nor do data demonstrate that administration of antibiotics in this setting, or giving longer courses of antibiotics or IV instead of orally, improves the healing or reduces recurrence of ulcers in this setting. However, as mentioned, several studies have found

that giving antibiotics, and longer courses of them, is associated with harm in the absence of surgical closure of the wound, including increased wound breakdown.[172,175]

Thus, in the absence of evidence for a therapeutic benefit and with data demonstrating potential harm, we do not recommend routinely administering antibiotics as a treatment for osteomyelitis underlying a pressure ulcer without an intent to both surgically debride and provide definitive wound closure. The primary therapeutic modalities for patients who are managed conservatively include local wound care, pressure offloading, and addressing the psychosocial/behavioral drivers leading to wound development and resulting in an increased risk of flap failure (e.g., malnutrition, smoking). We also do not recommend routinely ordering testing to diagnose osteomyelitis in this setting, as it will not change clinical care or management.

Furthermore, we note that even with implementation of a multi-modal, multi-disciplinary approach, there are no data to indicate that antibiotic administration reduces the risks of flap failure or recurrence of the wound/ulcer, nor are there data to guide the duration of antibiotic administration in this setting. Nevertheless, given that osteomyelitis exacerbated the risk of subsequent wound flap failure in one large retrospective study,[179] it is reasonable to administer adjunctive antimicrobial therapy to treat biopsy-confirmed osteomyelitis in the setting of a multi-modal, multi-disciplinary approach, including definitive wound closure with curative intent.

In cases where an aggressive strategy is taken, a reasonable multi-pronged approach based on a limited number of observational studies could include: 1) nutritional optimization (to support wound healing); 2) surgical debridement down to healthy tissue followed by obtaining surgical bone Gram stain, cultures, and histopathology; 3) definitive surgical wound closure; and 4) psychosocial interventions that include pressure off-loading, education, and treatment of tobacco use disorder, depression, or other psychological factors that may impede wound healing.[20,21] In situations where surgical wound closure is not intended, the same multi-pronged approach to management still applies, although obtaining bone biopsy for culture and histopathology and prescribing antibiotics should be avoided. Periodic debridement of necrotic or non-viable tissues and wound care remain important strategies for decreasing complications, including acute skin and soft tissue superinfection, even when curative approaches are not pursued.

In the setting of such a multi-faceted approach with surgical wound closure, and with confirmation of osteomyelitis on intra-operative histopathology, durations of antibiotics should generally not exceed 6 weeks (see section 7 for a full discussion on antibiotic durations for osteomyelitis), and some have suggested that courses may be as short as 2 weeks for superficial, cortical osteomyelitis.[163,182,183] Antimicrobial therapy may be administered orally in patients who are likely to absorb the medications and for whom an oral regimen will be active against the etiologic pathogens (see section 5 for a full discussion on oral antibiotic therapy for osteomyelitis).[16,184] The choice of antimicrobial agent should be individualized based on patient comorbid factors, allergies, and microbial type and antimicrobial sensitivities.

We emphasize that this guideline focuses on the diagnosis and management of osteomyelitis underlying pressure ulcers specifically, not including the management of acute skin and soft tissue infections that can complicate pressure ulcers. Diagnostic studies may be indicated to assess for infectious complications of wounds aside from osteomyelitis, such as abscesses, and antimicrobial therapy is appropriate for such infections, even in the absence of multidisciplinary plans for management of potential osteomyelitis.

© 2022 Spellberg B et al. *JAMA Network Open.*

Question 3: When should empiric therapy be administered in the treatment of osteomyelitis?

Executive Summary:
It is desirable to identify a microbial etiology of osteomyelitis whenever possible, as empiric antibiotic treatment without adequate cultures may lead to unnecessarily prolonged, broad-spectrum antibiotic use. Some observational studies suggest that administration of antibiotics prior to bone biopsy may modestly decrease yield of cultures for patients with osteomyelitis, including DFO and PJI. Thus, presuming other microbiologic methods (e.g., blood cultures) have not already established an etiology, it is reasonable to delay initiation of antibiotic therapy until bone or joint microbiological samples can be obtained for culture. However, the data are mixed, as other observational studies have not found an effect of pre-biopsy antibiotics on culture yield. Furthermore, prior antibiotics are unlikely to alter histopathology results. Decisions regarding the delay of empiric therapy therefore balance potential harm due to the risk of progression of life-threatening infection (e.g., sepsis, bacteremia, necrotizing fasciitis) or impending spinal cord compression against the potential benefit of obtaining microbiological data. Even if empiric antibiotics are initiated, subsequent biopsy, tissue, or synovial fluid culture may be helpful to establish a microbial etiology, enabling targeting therapy, as observational studies demonstrate some culture yield despite antecedent antibiotics.

Overall Summary:

Observational Data of Impact of Empiric Antimicrobial Therapy on Osteomyelitis
There are limited data on the yield of bone biopsies in osteomyelitis and the effect of pre-biopsy antibiotics on pathogen recovery. Overall, studies are retrospective in nature, small, and primarily focus on vertebral osteomyelitis. In addition, the durations and/or spectrums of pre-biopsy antibiotics were variable. Nevertheless, several studies suggest that the diagnostic yield of biopsy may be diminished in cases of vertebral osteomyelitis with antecedent antibiotic use.

In a retrospective study of 72 patients with confirmed vertebral osteomyelitis, of whom 40 underwent 46 CT-guided biopsies, culture positivity was significantly lower among patients who had been treated with antibiotics in the previous 48 hours (23% vs. 60%, p = 0.013).[185] Similarly, in a case series of 20 patients with vertebral osteomyelitis, 8 of 20 (40%) patients received antibiotics before the biopsy, with only 2 of 8 (25%) growing an organism after antibiotic use, in comparison to 6 out of 12 (50%) cases in which an organism was isolated without antibiotic use.[186]

However, other studies suggest that pre-biopsy antibiotics may not necessarily impact pathogen recovery.[187-191] For instance, in a retrospective cohort study of 150 adult inpatients with hematogenous vertebral osteomyelitis conducted by Marschall et al., the association of pre-biopsy antibiotics, which was defined as any antibiotic exposure within 14 days prior to biopsy, with negative culture results was not statistically significant (adjusted odds ratio (OR) 2.3; 95% CI, 0.8-6.2; p = 0.1).[187]

Similarly, in a retrospective multicenter study of 104 patients, Wong et al. studied the effect of stopping antibiotics prior to biopsy and found that it had no significant effect on culture positivity when compared to patients with or without pre-biopsy antibiotics.[190] Of note, the authors did not provide data on the precise time the antibiotics were stopped prior to biopsy (e.g., holding for 2 hours vs. 24 hours prior to biopsy might differ in result). Furthermore, subgroup

© 2022 Spellberg B et al. *JAMA Network Open.*

analysis from Lopez Floro et al. found that when comparing patients who received a single dose of an antibiotic with patients who received longitudinal antibiotics prior to biopsy, patients who had multiple doses prior to biopsy had statistically significant lower culture positivity ($p = 0.004$).[188] Thus, single doses of antibiotics pre-biopsy may be less likely to affect culture results than multiple doses. In addition, the match between the antibiotic used prior to the biopsy and the sensitivity profile of the organism can also negatively affect the culture positivity.

In sum, although results from multiple studies are inconsistent and the definitions of pre-biopsy antibiotics are not well-defined, several studies suggest that the culture positivity yield of biopsy may be diminished in cases of vertebral osteomyelitis with antecedent antibiotic use. Furthermore, for other diseases, such as bacteremia, receipt of antibiotics prior to culture generally reduces culture yields,[192] and it is therefore likely that the sensitivity of bone cultures is also reduced by antecedent antibiotics. However, no study demonstrated reduced sensitivity of histopathology results for the diagnosis of osteomyelitis with prior antibiotic therapy.

Observational Data of Impact of Empiric Antimicrobial Therapy on DFO

There is an overall paucity of data on the yield of bone biopsies in DFO and the effect of pre-biopsy antibiotics on pathogen recovery. Studies are retrospective in nature and small in size, with confounding variables and lack of standardization which make it difficult to compare them. In addition, several observational studies that focus on the microbiologic accuracy of bone biopsy excluded patients who received antecedent antibiotics within two weeks prior to bone biopsy, leading to overall minimal data being available on this subject matter.

Among three observational studies including patients who received antibiotics within two weeks of biopsy, the proportion of culture positive percutaneous bone biopsies (PBBs) was high, ranging from 83% to 99%.[193-195] However, these high yields should be interpreted with caution because samples were often collected through the ulcer bed, and thus may include colonizing/contaminating microbes rather than true pathogens. In contrast, in a study including 75 biopsies of non-vertebral bones with clinical concern for osteomyelitis, among patients who received antibiotics within 24 hours of the biopsy, only 24% of cultures were positive.[103] This compared to a positivity rate of 42% among patients who did not receive antibiotics within 24 hours of biopsy, suggesting that pre-procedural therapy lowered culture sensitivity.

In a meta-analysis by Schechter et al., the proportion of patients who received antibiotics within two weeks prior to percutaneous bone biopsy (PBB) for a diagnosis of DFO ranged between 32% and 53%.[102] In their analysis, they found studies that excluded patients who received antibiotics ≤2 weeks before the PBB reported positive cultures in 56% to 87% of patients, with a pooled (95% CI) positive culture rate of 72% (59%-83%).[102,107,196-198] By comparison, studies that included patients who received antibiotics ≤2 weeks before the PBB report higher culture positivity from PBB, ranging from 83% to 99% with pooled (95% CI) positivity of 96% (84%-99%).[193-195]

In other studies, duration and/or spectrum of pre-biopsy antibiotics were unclear. Aragon-Sanchez et al. reviewed 185 patients with osteomyelitis from 2002 to 2007 hospitalized in a diabetic foot unit.[199] Patients initially treated for dry necrosis that became secondarily infected were excluded. Preoperative diagnosis of osteomyelitis was based on PTB test through the ulcer and a radiological study of the foot. All patients without penicillin allergies were given ampicillin-sulbactam, with the first dose at the time of anesthesia induction. Bone culture was collected during surgical intervention. One hundred and thirty-two patients (71.3%) received antibiotics prior to admission. Only 20 cultures were negative; 154 specimens yielded an

organism. The authors concluded that negative cultures were not related to previous antibiotic treatment (p = 0.1). However, the potential impact of dose and duration were not clearly documented.

In a multicenter, small RCT of 40 patients, the authors suggested delaying antibiotic administration until the availability of culture results in DFO did not affect clinical failure rates.[39] This study included patients with DFO treated non-surgically. Empiric therapy, mostly amoxicillin-clavulanate, was prescribed while waiting for culture results if the treating physician considered it necessary, which was the case in 18 patients (45%). For the remaining patients for whom empiric therapy was not given, antibiotic therapy was initiated a median of 14 days (range 5 to 19) after the bone biopsy. Antibiotics were given orally for the full treatment course for 22 patients (55%) or IV therapy was used for 5-7 days, then followed by oral therapy in 18 patients (45%). Patients with or without empiric therapy had similar failure rates (6/18, 33% vs. 8/22, 37%, respectively; p = 0.8).

In contrast, a retrospective multicenter study from France reported that bone culture-based antibiotic therapy was associated with higher remission rates [OR 4.8 (95% CI, 1-22.7), p = 0.04] in patients with non-surgically treated diabetic foot osteomyelitis.[200] Therefore, while the data are mixed, when possible, it may be desirable to delay initiation of antibiotic therapy for stable patients until bone or deep tissue culture can be obtained.

Observational Data of Impact of Empiric Antimicrobial Therapy on PJI

Observational studies have shown preoperative therapeutic antibiotics are associated with a decrease in intraoperative culture positivity in patients with PJI. In a retrospective, case-control study, 135 patients with culture-negative PJI were matched to 135 patients with culture positive PJI.[201] The investigators reported that 64% of patients with culture-negative PJI and 25% of patients with culture-positive PJI received antibiotics within three months before the diagnosis of culture-negative PJI (OR 4.1; 95% CI 2.3-7.2). The median duration of prior antibiotic treatment for culture-negative PJI was 35 days vs. 18 days for culture positive patients. Cefazolin and ciprofloxacin were the most used antimicrobials (16% and 15% respectively). The study found that patients with culture negative PJI were more likely to have received antibiotics within three months of their diagnosis (OR 4.1; 95% CI, 2.3-7.2), suggesting that pre-culture therapy might reduce sensitivity.

Similarly, in a study of 182 patients with late PJI after total knee arthroplasty, in which 65 patients received antibiotics prior to aspiration, the authors found that patients with pre-aspiration antibiotic administration are more likely to have negative culture than those without antecedent antibiotics (26.4% vs. 12.9%; RR 2; 95% CI, 1.1-3.9; p = 0.046).[202] Overall, patients who received pre-aspiration antibiotics also had lower values for serologic and synovial markers for PJI.

Moreover, from a prospective trial of 331 patients with total knee or hip prostheses, in which 79 had PJI and 252 had aseptic failure, Trampuz et al. concluded that preoperative administration of antibiotics lowers the positive yield of both tissue and sonicate-fluid cultures from patients with PJI.[203] The tissue culture sensitivity decreased from 76.9% to 47.8% to 41.2% as the antimicrobial free interval before surgery decreased from greater than 14 days to 4-14 days to less than 3 days (p < 0.001). The same effect was observed in sonicated-fluid culture where culture positivity decreased from 82.1% to 87% to 58.8% as the antimicrobial-free duration trended lower, from greater than 14 days to 4-14 days to less than 3 days prior to

surgery (p = 0.1). Of note, 9 of the 31 patients with negative tissue culture had antibiotic stopped greater than 14 days prior to surgery and 7 out of 9 patients had negative sonicate-fluid cultures.

In contrast, two recent retrospective studies suggested antimicrobial therapy prior to surgery may not negatively impact intraoperative culture positivity.[135,204] Therefore, future studies are needed to determine the optimal duration and/or the effect of withholding antimicrobial therapy prior to obtaining meaningful culture results for patients with PJI.

Overall Conclusions

On balance, although the data are mixed, multiple studies across all types of osteomyelitis have suggested a modest decrease in biopsy culture positivity with prior antibiotics. However, none of these studies have suggested a reduction in positivity of histopathology. Hence the risk of prior therapy is to reduce confirmation of microbial etiology, limiting ability to tailor antimicrobial therapy. If other microbiological methods identify the etiologic pathogen, these considerations become superfluous. Furthermore, for patients who are clinically unstable or have serious or life or limb-threatening infections in addition to suspected osteomyelitis, the risk:benefit of waiting for biopsy to initiate therapy may not be favorable, in which case empiric therapy should be administered without delay.

© 2022 Spellberg B et al. *JAMA Network Open.*

Question 4: Are there preferred antibiotics with which to treat osteomyelitis?

a. Which empiric antimicrobial agents are preferred for osteomyelitis?

Executive Summary:

Based on observational and randomized controlled studies, aerobic gram-positive cocci, primarily *S. aureus,* have been the organisms most frequently isolated from culture in patients with osteomyelitis, including DFO. Enterobacterales have been the predominant group of gram-negative pathogens, with *E. coli* the most common. Thus, when treating osteomyelitis, it is reasonable to empirically cover gram-positive cocci, primarily *Staphylococcus* spp., and gram-negative bacilli if therapy cannot be delayed until culture availability (Table 2). For DFO, many physicians add anaerobic activity; however, data are not available to determine the benefit or harm of this approach. Pseudomonal activity is generally not necessary in treating osteomyelitis unless patients have been exposed to multiple prior courses of antibiotics, the wound is gangrenous, the organism has been previously cultured, the patient underwent a recent (e.g., < 3 months) surgical procedure in a healthcare setting, or the patient has a specific site of infection particularly prone to *P. aeruginosa* (e.g., malignant otitis externa).

For early, late, and hematogenous PJI, *S. aureus* and coagulase-negative *Staphylococcus* have been the most commonly isolated organisms. Gram-negative bacilli, most commonly Enterobacterales, have also been regularly isolated. Thus, reasonable empiric therapy for PJI of all stages generally includes coverage for gram-positive cocci and Enterobacterales. Antibiotic regimens to treat early (< 3 months since procedure), but not later, PJI may include coverage for *P. aeruginosa*, although some authors feel this is not routinely necessary depending on local microbiology. Anaerobes, such as *Peptostreptococcus* and *C. acnes*, are isolated infrequently. *C. acnes* is more often isolated in shoulder PJI compared to other joints, and thus would warrant empiric coverage for shoulder PJI; however, this is usually accomplished with anti-staphylococcal coverage. In all cases, local susceptibility profiles inform empiric therapy.

© 2022 Spellberg B et al. *JAMA Network Open.*

Table 2: Reasonable Empiric Antimicrobial Therapy Options with Published Data*

Types of Osteomyelitis	Empiric IV Antibiotics[†]	Alternative Empiric IV Antibiotics	Empiric Oral Antibiotics[Ψ]
**Osteomyelitis without a Retained Implant**	ceftriaxone<br>±<br>vancomycin	Alternative to β lactam: fluoroquinolone<br><br>Alternative to vancomycin: linezolid, daptomycin, or clindamycin	TMP-SMX or clindamycin[φ] or linezolid[φ] or fluoroquinolone or doxycycline[¥]<br><br>± rifampin
**Diabetic Foot Osteomyelitis (DFO)**	ampicillin-sulbactam or amoxicillin-clavulanate<br>or<br>ceftriaxone[‡] ± metronidazole<br><br>± vancomycin	Alternative to β lactam: fluoroquinolone[‡] ± metronidazole<br><br>Alternative to vancomycin: linezolid, daptomycin, or clindamycin	amoxicillin-clavulanate<br>or<br>TMP-SMX or clindamycin[φ] or linezolid[φ] or fluoroquinolone or doxycycline[¥]<br>± metronidazole[‡]<br>± rifampin
**Osteomyelitis with a Retained Implant (including PJI)**			
**< 3 months since procedure (early)**	(anti-pseudomonal β lactam or ceftriaxone) + vancomycin[ξ]	Alternative to β lactam: fluoroquinolone<br><br>Alternative to vancomycin: linezolid, daptomycin, or clindamycin	fluoroquinolone ± rifampin<br><br>or<br><br>If gram-positive confirmed:<br><br>TMP-SMX or clindamycin[φ] or linezolid[φ] or doxycycline[¥]<br><br>± rifampin
**≥ 3 months after procedure (later onset)**	ceftriaxone + vancomycin[ξ]	Alternative to β lactam: fluoroquinolone<br><br>Alternative to vancomycin: linezolid, daptomycin, or clindamycin	TMP-SMX or clindamycin[φ] or linezolid[φ] or fluoroquinolone or doxycycline[¥]<br><br>± rifampin

* This table addresses reasonable therapies with published data to be administered in the absence of available Gram stain, culture, histopathology, or other guiding information that enable targeted therapy. Biopsies should be obtained for such information prior to initiation of therapy when the

© 2022 Spellberg B et al. *JAMA Network Open.*

risk:benefit ratio is favorable, see question 3 for a thorough discussion of initiation of empiric therapy vs. waiting for biopsy information to target therapy. In all cases, antibiotic selection should be adjusted based on local sensitivities for likely target pathogens. This table is not meant to indicate that other therapeutic options cannot be considered for specific patients based on clinical circumstances.

†Add empiric anti-MRSA coverage (e.g., vancomycin) and/or replace ceftriaxone with an anti-pseudomonal β lactam (e.g., cefepime, piperacillin-tazobactam, etc.) if specific risk factors for MRSA (e.g., colonization, prior MRSA infection, healthcare exposure with endemic MRSA) and/or *P. aeruginosa* (exposed to prior courses of antibiotics, prior cultures with *P. aeruginosa,* gangrenous wounds, recent surgical procedures, specific sites of infection such as malignant otitis externa) are present, respectively, see question 4, sections b and c. When such risk factors are present, the authors unanimously prefer the use of non-carbapenem anti-pseudomonal options for stewardship reasons, unless there is a specific concern for ESBL pathogens. Similarly anti-anaerobic coverage is not routinely needed, but if the wound is gangrenous or there is specific concern for anaerobic infection, metronidazole may be added, or ceftriaxone replaced with ampicillin-sulbactam or amoxicillin-clavulanate. Finally, for patients in whom an MRSA active agent is deemed unnecessary, some authors prefer to add an anti-staphyloccocal β-lactam (e.g., oxacillin, cloxacillin, nafcillin, cefazolin) to ceftriaxone.

‡Anaerobic coverage is routinely added by many practitioners; however, data are not available to demonstrate whether it adds clinical benefit or not.

ξWhile many authors would initiate empiric anti-pseudomonal therapy, some authors do not believe that anti-pseudomonal coverage is routinely needed for early PJI infection, based on the frequency with which the organism is locally encountered. Most authors who would initiate rifampin prefer to wait until oral transition but some authors would consider initiating empiric IV rifampin. If rifampin use is being considered, it may be prudent to wait until bacteremia is cleared (if present) and surgical source control is achieved (if necessary), to reduce the risk of treatment failure.[205] See question 4, section c for a discussion of empiric pseudomonal therapy, and section e for a full discussion of the potential benefits:risks of adjunctive rifampin therapy.

ΨSee question 5 for full discussion of oral therapy, including which agents, and timing of initiation. TMP-SMX = trimethoprim-sulfamethoxazole. Rifampin may be important to add to fluoroquinolones when treating *S. aureus* infections, and possibly when treating *Pseudomonas* or *Acinetobacter* infections, to reduce emergence of resistance. Other uses of rifampin are discussed in question 4, section e.

φAs clindamycin and linezolid have no reliable gram-negative coverage, they should only be used when the clinician is confident that the infection is not likely caused by a gram-negative pathogen, or they should be administered with gram-negative coverage.

¥There are less published data for doxycycline, however it has been used with anecdotal success and was used in a minority of patients in the OVIVA trial,[10] so it may be an alternative agent in individual patients.

Overall Summary:

© 2022 Spellberg B et al. *JAMA Network Open.*

Pyogenic Osteomyelitis in the Absence of an Implant

Observational and randomized controlled studies have concluded that aerobic gram-positive cocci, primarily *S. aureus,* are the most frequently cultured organisms in patients with osteomyelitis.[17,44,187-189,206-209] The rate of MRSA strains has varied by study, ranging from 0%[206] to 46%,[187] depending on geography. Other common aerobic gram-positive cocci isolates included *Streptococcus* spp. and *Enterococcus* spp. Gram-negative bacilli were identified from cultures approximately a quarter of the time, varying by study. The Enterobacterales were the most predominant group of gram-negative pathogens, of which *E. coli* has been the most frequently identified species. *P. aeruginosa* was isolated in cultures at rates of 10% or less in most studies. For pyogenic vertebral osteomyelitis, cultures were typically monomicrobial (86%), with blood cultures positive a little over half the time (58%).[206] Microbiologic etiology may be influenced by patient specific elements such as prior environmental or community exposures, certain risk factors such as intravenous drug use,[188] recent healthcare exposure, or recent antibiotic treatment. Known MRSA colonization is the largest individual risk factor for MRSA infection.[210]

In an observational cohort study of 358 patients with hematogenous vertebral osteomyelitis conducted in five tertiary care hospitals in the Republic of Korea,[207] the most frequently isolated organisms were methicillin-susceptible *S. aureus* (MSSA) (33.5%), followed by MRSA (24.9%) and Enterobacterales (19.3%). *P. aeruginosa* was isolated in only five specimens (1.4%). Moreover, the authors found differences in the proportion of pathogens isolated between community-acquired and healthcare-associated hematogenous vertebral osteomyelitis. MRSA was more frequent in healthcare-associated hematogenous vertebral osteomyelitis (43.6% vs. 13.8%; $p < 0.001$), whereas MSSA and *Streptococcus* spp. were more commonly found in community-acquired hematogenous vertebral osteomyelitis (44% vs. 13.8%; $p < 0.001$, 16% vs. 4.5%; $p = 0.001$ respectively).

Thus, although RCTs assessing empiric antibiotic choice are not available, based on observational data, selecting an empiric antibiotic therapy with coverage of *S. aureus* (including MRSA), *Streptococcus* spp., and Enterobacterales is reasonable for osteomyelitis in the absence of an implant. A reasonable regimen is a third-generation cephalosporin lacking pseudomonal coverage, such as ceftriaxone, with or without addition of vancomycin for MRSA coverage. Where MRSA is uncommon, some experts prefer to replace vancomycin with a β lactam with anti-staphylococcal activity (e.g., oxacillin, flucoxacillin). In those with cephalosporin allergy, TMP-SMX, or a fluoroquinolone, such as levofloxacin or ciprofloxacin, could be used as alternatives, provided that local antibiogram data are favorable. In cases where there is high suspicion of more resistant pathogens, such as extended spectrum β-lactamase (ESBL)-producing gram-negative bacilli, or *P. aeruginosa*, using a carbapenem or cefepime may be reasonable. Consideration of patient specific factors, such as comorbidities, prior healthcare exposure including procedures, known colonization with antibiotic-resistant organisms, severity of illness including sepsis or septic shock, and local epidemiology and resistance patterns is important when selecting an empiric regimen.

DFO

Overall, *S. aureus* has been the most frequently isolated organism from bone biopsy results in patients with DFO; MRSA has varied in isolation between 0%-20% of cultures.[38-40,107,193,196,199,200,211] Coagulase-negative *Staphylococcus* has been the next most frequently

© 2022 Spellberg B et al. *JAMA Network Open.*

isolated organism, although it is difficult to determine if the organism is pathogenic when isolated, as it likely reflects surface colonization/specimen contamination. Other gram-positive organisms, such as *Streptococcus* spp., *Enterococcus* spp., and *Corynebacterium* spp., have also been described from culture results. Isolation of organisms typically considered normal skin flora, such as coagulase-negative *Staphylococcus* or *Corynebacterium* spp., can be of unclear significance, although pairing histology findings with culture results can help delineate whether these organisms are pathogenic. Of aerobic gram-negative bacilli, Enterobacterales were reported in 12%-50% of cultures, with the most common species being *E. coli*, *Klebsiella* spp., and *Proteus* spp. Typically, when aerobic gram-negative bacilli were isolated in culture, the culture was polymicrobial. Rates of polymicrobial cultures ranged from 26%-85%. Obligate anaerobes, such as *Peptococcus*, *Peptostreptococcus*, *Prevotella* spp., *Clostridium spp.,* or *Bacteroides spp.*, were isolated in higher frequency in older studies,[212-215] while more contemporary studies report lower rates (3%-12%).[39,107,196,216] The differences in rates of anaerobic isolation may be due to differences in sampling technique, transport time to the lab, and microbiology lab handling of the samples.[217]

In a single center retrospective study over 10 years in Spain, patients with biopsy proven DFO who had gram-negative bacilli isolated in bone culture (n = 150) were compared to those with other organisms or sterile cultures (n=191).[218] Overall, 58.3% (224/384) of bone specimens isolated gram-positive cocci, 40.6% (156/384) gram-negative bacilli, and 1.1% (4/384) fungi. The most frequent gram-negatives isolated overall were *E. coli* (21.2%), *P. aeruginosa* (15.4%), and *Enterobacter cloacae* (12.8%). Patients whose cultures isolated gram-negative organisms more frequently had fetid odor, necrosis, soft tissue infection accompanying osteomyelitis, and clinically severe infection compared to those without gram-negative organisms. By multivariate analysis, having a glycosylated hemoglobin <7% (OR 2; 95% CI, 1.1-3.5) and a wound caused by traumatic injury (OR 2; 95% CI, 1-3.9) were found to be the most significant predictors to isolate gram-negative bacilli from bone samples. Duration of the foot wound did not affect the likelihood of isolating a gram-negative pathogen.

Thirty-four bone samples from US patients who were hospitalized with moderate-to-severe DFI with a high suspicion of DFO were evaluated by 16S ribosomal ribonucleic acid (rRNA) gene sequencing.[219] *S. aureus* was the most common pathogen isolated, at 50% (13/26) by conventional culture technique and 86.9% (20/23) by sequencing methods. The distribution of other gram-positive organisms identified by 16S sequencing technique included: *Streptococcus* spp. 56.5%, (13/23), unknown Dermabacteriae 34.8% (8/23), and *Corynebacterium* spp. 78.3% (18/23). *Corynebacterium* spp. appeared to have a lower contribution to the total bacterial population compared to *Staphylococcus* spp., and its pathogenic role in DFO is not well described. Gram-negative pathogens that were sequenced included: *Pseudomonas* spp. 21.7% (5/23) and *Enterobacter* spp. 26.1% (6/23). Facultative anaerobes isolated by sequencing included *Actinomyces* 26.1% (6/23) and *Helcococcus* spp. 21.7% (5/23). Obligate anaerobes included *Peptoniphilus* 73.9% (17/23), *Finegoldia* 65.2% (15/23), *Anaerococcus* 52.2% (12/23), *Clostridium* 39.1% (9/23), *Porphyromonas* 30.4% (7/23), and *Prevotella* 21.7% (5/23). Of the three samples that did not sequence, *Stenotrophomonas maltophilia*, *S. aureus*, and *Enterobacter cloacae* were isolated by conventional culture methods. Compared to standard culture methods, 16S rRNA sequencing found significantly more anaerobic pathogens (86.9% vs. 23.1%, p = 0.001), more polymicrobial cultures (91% vs. 64% p

= 0.02), and more gram-positive bacilli (78.3% vs. 3.8%, $p < 0.001$). The clinical significance of these anaerobes remains unclear.

Although aerobic gram-positive cocci remain the predominant pathogens in many studies, one may also take geographic location of the patient into consideration when determining empiric antibiotics. In a meta-analysis by Zenelaj et al., studies of DFI conducted in countries with a warm climate, such as desert or tropical, tended to have a relatively lower percentage of infections caused by *Staphylococcus* spp.[220] Instead, gram-negative bacilli were isolated in higher frequency compared to rates published in European countries or the US.[211,221,222]

In cases of less severe DFO that did not have associated complications (e.g., necrotizing soft tissue infections or peripheral artery disease), oral therapy with either ciprofloxacin, amoxicillin-clavulanic acid, or trimethoprim-sulfamethoxazole (TMP-SMX) has been used.[38] If IV therapy is needed initially, then monotherapy of ampicillin-sulbactam, ceftriaxone, or the fluoroquinolones for patients with β lactam allergies may be reasonable options. If broader gram-positive coverage (i.e., MRSA) is needed, addition of clindamycin (PO or IV), linezolid (PO or IV), doxycycline (PO or IV), or vancomycin (IV) may be also reasonable.

Although anaerobic pathogens are isolated in bone biopsy cultures, there are currently no available data regarding whether empiric anaerobic therapy affects outcomes, either with improved cure rates or potentially higher adverse event rates. Many of the monotherapy options we list have varying degrees of anaerobic coverage. Thus, routine addition of broad anaerobic coverage with drugs like metronidazole for empiric therapy may not be required. Addition of metronidazole is of particular concern for patients with underlying neuropathy, as prolonged therapy can result in drug-induced neuropathy.

Pyogenic Osteomyelitis with a Retained Implant (including PJI)

For PJI, the frequency in which the gram-positive, gram-negative, or anaerobic pathogens are isolated varies by the timing of onset of PJI from the placement of the prosthetic implant. Early PJI (definitions in the literature vary but range from 1-3 months post-op) is acquired due to contamination intraoperatively and is typically caused by virulent organisms. Delayed-onset PJI (definitions used range from 1-12 months post-op) are acquired during time of surgery but are caused by less virulent organisms where the infectious presentation may not present within the immediate postoperative period. Late onset PJI is usually caused by hematogenous route or direct inoculation from other infectious foci.

Overall, *S. aureus* has been the most frequent cause of PJIs, regardless of whether they are early, delayed, late onset, or hematogenous PJI, contributing to approximately one third of cases (range 9%[223]-62%[224]). Rates of MRSA PJI have been low, ranging from 0%[225] (Sweden) to 17%[226] (US) and vary by country. Coagulase-negative *Staphylococcus* has also been isolated in high frequency, ranging from 7%[227] to 50%,[226] with *S. epidermidis* being the most frequently identified species within this group.[228] Other gram-positives, such as *Streptococcus* spp., *Enterococcus* spp., and *Corynebacteirum* spp., have been isolated in decreasing frequency. Of the aerobic gram-negative bacilli, Enterobacterales have been the most common, ranging from 3%[226] to 33%.[223] *P. aeruginosa* (~<10%) and *Acinetobacter* spp. (<3%) were infrequently isolated. Obligate anaerobes, such as *Peptostreptococci* and *Cutibacterium acnes*, were also isolated infrequently. *Cutibacterium acnes* was more often isolated in shoulder PJI compared to other joints, likely due to the organism being common flora in the axilla.[229-236] Cultures may be

polymicrobial up to 39% of the time and were more likely to be a cause of early PJI (<3 months) vs. late PJI.[227,237,238]

In a multicenter, retrospective study of PJIs from Spain (n = 2,524), the four most common organisms found in early postop PJI (<1 month from procedure) were *S. aureus* (35.6%), *S. epidermidis* (15.5%), *E. coli* (15.4%), and *P. aeruginosa* (15.3%).[239] For chronic PJI (>1 month post procedure and symptoms persisting >3 weeks), *S. epidermidis* (33.2%), *S. aureus* (20%), coagulase-negative *Staphylococcus* not identified to species level (16.7%), and *C. acnes* (5.2%) were most commonly isolated. In acute hematogenous infections (symptoms <3 weeks after uneventful procedure), *S. aureus* (39.2%), *E. coli* (12.5%), *S. agalactiae* (10.9%), and viridans group streptococci (4.5%) were the most common. The proportion of PJIs caused by multidrug resistant bacteria increased from 9.3% in 2003-2004 to 15.8% in 2011-2012 (p = 0.008). The increase was primarily due to multidrug resistant gram-negative bacilli (5.3% to 8.2%, p = 0.032) during the time period, rather than MRSA (4.7% to 7.6%, p = 0.2).

In a retrospective study of 112 patients with elbow, ankle, and shoulder PJI in the UK, gam-negative bacilli were more frequently isolated in early PJI (<3 months from prosthesis placement) and late chronic PJI (>12 months from prosthesis placement).[238] Gram-negative bacilli, including Enterobacterales, *Pseudomonas*, and *Acinetobacter* spp., as well as anaerobes were more likely to be isolated in early PJI. However, after three months, the frequency of both decreased. No gram-negative bacilli or anaerobes were isolated between 3-12 months after prosthetic joint placement. Enterobacterales and anaerobes were isolated in 4.2% (1/24) and 8.3% (2/24) of cases occurring >12 months after surgery. The rate of polymicrobial samples also declined, with the highest rate of polymicrobial samples within the first three months of implantation (47%), compared to 9.1% and 20.8% at 3-12 months and >12 months after implantation, respectively.

In a recent observational study from Australia, among 607 patients with prosthetic joint infection, the microbiology differed among patients with early PJI vs. other types.[240] *S. aureus* was the most common pathogen overall, but patients with early PJI had twice the frequency of Gram negative bacterial infections, and including *P. aeruginosa* as compared to later PJI.

Fungal prosthetic joint infections occur infrequently (1%) compared to bacterial causes.[241] Patients with fungal PJI have different risk factors compared to those with bacterial causes including immunosuppression, overuse of antibacterials, presence of indwelling catheters, multiple revision surgeries, and complex reconstructions. *Candida* spp. were the etiology in the majority of fungal PJI. *Aspergillus* spp. and *Rhodotorula spp.* were rare causes.[241-243]

Thus, in order to cover the most likely pathogens for PJI, a reasonable empiric therapy for early PJI (<3 months) could include a combination of a third- or fourth-generation cephalosporin with antipseudomonal activity, or piperacillin-tazobactam, with or without IV vancomycin for MRSA coverage. However, some authors believe that anti-pseudomonal therapy is not routinely needed for early PJI depending on local microbiology of infection. Indeed, as some of the results mentioned above were derived from single center studies and some with small sample sizes, local epidemiology and resistance patterns will generally dictate the need or not for broader coverage for multidrug resistant gram-negative bacilli.

Alternatives to vancomycin can include daptomycin or linezolid,[42,244] while fluoroquinolones may be options for patients with significant penicillin or cephalosporin allergies. For delayed onset PJI, non-pseudomonal gram negative coverage combined with vancomycin IV with may be reasonable, although gram-negative bacilli are less frequently isolated in this group. Empiric therapy for late onset PJI (>12 months) may include vancomycin

© 2022 Spellberg B et al. *JAMA Network Open.*

IV with a third-generation cephalosporin. As late onset PJI is unlikely to include *Pseudomonas* spp., empiric antipseudomonal therapy is not routinelynecessary in the absence of other risk factors. Empiric coverage of fungal etiologies in PJI is likely not needed unless the patient has had a prior PJI with isolation of a fungal pathogen.

b. When should antimicrobial coverage against MRSA be included?

Executive summary:
Based on observational and RCT data, rates of MRSA bone and joint infections vary by country. In areas with low MRSA prevalence, and for patients who are not known to be colonized or previously infected by MRSA, it may be reasonable to hold MRSA coverage, and focus on MSSA coverage. In patients known to be colonized or previously infected by MRSA (the largest individual risk factor for MRSA infection), or at centers with higher rates of MRSA among their *S. aureus* isolates, it is reasonable to initiate an anti-MRSA agent empirically while waiting for culture results, particularly for clinically unstable patients.

Overall summary:
Vertebral Osteomyelitis
Several studies reported that MRSA rates fell dramatically across multiple hospitals between the early 2000s and 2010-2016.[245-247] Despite decreases in MRSA incidence, MRSA infection remains more frequently observed in healthcare-associated settings than in community settings. Indeed, a meta-analysis found that there was a surge of community-acquired MRSA infections between the mid-1990s and 2005 in the US, but with substantially declining rates since then.[248]

As far as risk factors for MRSA in osteomyelitis, an observational cohort study performed in the Republic of Korea has shown that patients who are infected with MRSA hematogenous vertebral osteomyelitis were more likely to be older, have diabetes or a malignancy, and their infections were more frequently hospital-onset.[249] Similarly, a more recent publication in 2019 by Park et al. concluded that MRSA was more frequent in healthcare-associated hematogenous vertebral osteomyelitis than in community-acquired hematogenous vertebral osteomyelitis (43.6% vs. 13.8%; $p < 0.001$).[207] In these studies, healthcare-associated hematogenous vertebral osteomyelitis was defined as onset of symptoms after one month of hospitalization with no evidence of vertebral osteomyelitis at admission, hospital admission within six months before symptoms onset, or ambulatory diagnostic or therapeutic manipulations within six months before symptom onset. Furthermore, a retrospective study which included 586 patients with pyogenic vertebral osteomyelitis suggested MRSA may be more common in patients with chronic kidney disease regardless of being on dialysis (34.4% vs. 14.7%, $p < 0.05$).[250]

In one large study, colonization by MRSA in the nose or rectum far surpassed other factors in predicting MRSA as the etiologic pathogen for bacteremia.[210] These data suggest that colonization may similarly be a predominant predictor of MRSA of osteomyelitis as well.

DFO
Ashong et al. conducted a single-center retrospective review of 131 patients with an initial episode of probable or definite foot osteomyelitis.[251] Bone cultures were collected intraoperatively, percutaneously or with image-guided bone biopsies. Significantly more

© 2022 Spellberg B et al. *JAMA Network Open.*

patients who received insulin therapy were in the MRSA group than non-MRSA group (68.8% vs. 61.6%, p = 0.02). MRSA, MSSA, and other staphylococcal species were isolated in 31 (23%), 27 (20%), and 14 (10.4%) bone cultures, respectively. The studies showed MRSA bone isolates were not associated with a greater risk of treatment failure. Of note, patients who had MRSA isolated in bone culture but did not receive antibiotic therapy targeting it were not at higher risk for treatment failure of DFO. However, vancomycin was part of the study's empiric treatment algorithm.

Another study performed by Aragon-Sanchez et al. compared the outcome of surgical treatment between DFO caused by MRSA vs. MSSA in Spain.[252] The number of surgeries performed in patients with DFO caused by MRSA was significantly greater. However, there were no significant differences in the final outcome of surgical treatment or mortality between the two groups. Similarly, in a French, multicenter, RCT of DFO, MRSA isolation in bone biopsy culture was not associated with patient-level outcomes.[39]

Overall, these results indicate considerable variation in MRSA rates geographically, although they also demonstrate declining rates in many parts of the world. Local rates of MRSA, combined with information regarding colonization status of the patient (via MRSA nasal or perineal swab, or prior culture results), are reasonable to guide the choice of empiric MRSA selection.[210]

c. When should antimicrobial coverage against *P. aeruginosa* be included?

Executive Summary

Observational studies demonstrate that *P. aeruginosa* is an uncommon cause of osteomyelitis outside of patients with specific risk factors. Thus, empiric therapy including antipseudomonal agents can be limited to patients with such risk factors. For example, *Pseudomonas* spp. are more prevalent in patients residing in subtropical and tropical climates than in temperate climates. Other risk factors include the presence of chronic wound infections with multiple prior antibiotic courses, gangrene, a history of positive culture with *Pseudomonas* spp. in the past, a recent (e.g., < 3 months) surgical procedure in a healthcare setting (e.g., early PJI), or specific sites of infection (e.g., malignant otitis externa).

Overall Summary

<u>Osteomyelitis (Including DFO) without a Prosthetic Implant</u>

Multiple studies have shown *P. aeruginosa* is a relatively uncommon isolate in patients with vertebral osteomyelitis or DFO.[17,38-40,44,188,189,200,207-209,212-216] However, some observational studies suggest gram-negative bacilli may be more prevalent in patients who reside in Asian countries with warm and humid climates, have chronic or trauma-related wound infection, have contiguous wounds complicated by gangrene,[250,253] or are suffering peripheral vascular disease.

In studies of patients with DFI from India, Malaysia, Turkey, and Kuwait, gram-negative bacilli were frequently isolated (e.g., 76% from the study in India, 52% from Malaysia, 50% from Kuwait), and *P. aeruginosa* was the most common isolate, causing 22%, 20%, 25%, 30%, and 17% of infections, respectively.[254-257]

In contrast, a retrospective study from Spain did not find a positive correlation between gram-negative organism isolation, warm climate, and duration of the foot infection.[218] The study included 341 patients with DFO. Bone cultures were obtained intraoperatively. The study

© 2022 Spellberg B et al. *JAMA Network Open.*

suggested gram-negative organisms were more frequently isolated from patients with wounds that developed after trauma (p = 0.045).

In a retrospective review of 103 combat veterans with a diagnosis of osteomyelitis, gram-negative organisms were isolated in 91% of cultures of bone and deep wounds taken during initial debridement from patients with combat-related wounds.[258] Twenty-four percent of the specimens grew *P. aeruginosa*. In another retrospective study conducted in a trauma center in Brazil, Cordeiro de Carvalho et al. reviewed the clinical and microbiological profiles of 101 patients with gram-negative osteomyelitis associated with open fracture.[259] *P. aeruginosa* was isolated in 19.8% of these bone cultures.

In a retrospective study of 302 patients, King et al. found that those with peripheral vascular disease had a higher incidence of foot and ankle osteomyelitis caused by gram-negative organisms (OR 2.2; 95% CI, 1.3-3.6; p = 0.004).[247] *Pseudomonas* spp. were the most frequently isolated gram-negative pathogens. The author concluded that longer wound duration and differences in wound environment led to overall incidence of gram-negative organisms isolated in patients with peripheral vascular disease. This conclusion is supported by observational studies reporting an increased risk of *Pseudomonas* spp. infection in diabetic foot ulcers complicated by gangrene.[250,253]

There are also specific sites of infection that have been associated with a particularly high risk of *P. aeruginosa* as a cause of osteomyelitis, generally indicating empiric anti-pseudomonal therapy. For example, in multiple observational studies of patients with malignant otitis externa, *P. aeruginosa* has been a leading cause of infection.[260-262] Nail puncture wounds of the feet may also be associated with pseudomonal infection, although this observation is more anecdotal.[263-265]

PJI

Based on epidemiological studies, isolation of *P. aeruginosa* is more common in early PJI (<3 months) or acute hematogenous PJI than in late PJI.[266] In a large multicenter, retrospective study from Spain (n = 2,524), gram-negative bacilli were seldom isolated in chronic (>1 month post arthroplasty and symptoms >3 weeks in duration) or acute hematogenous PJI (symptoms <3 weeks after an uneventful procedure).[239] In contrast, *Pseudomonas* spp. were the fourth most common organism identified (15.3%) in those with early postoperative PJI <1 month post arthroplasty. Among those with pseudomonal infections, multidrug resistance increased significantly from 0.7% from 2003-2004 to 1.8% in 2011-2012, p = 0.044. Thus, contemporary, local resistance patterns will need to be evaluated to determine the most appropriate empiric choice for anti-pseudomonal coverage, if empiric coverage is needed. In some centers with low pseudomonal rates of infection, empiric coverage may not be routinely indicated for early PJI.

In a US cohort of hip or knee arthroplasty infections seen at a single center, 91 patients had PJI caused by *Pseudomonas* spp. between 1969 and 2012.[267] A little over half (57%) had hip PJI, 22% had a history of diabetes, 22% had history of GI or GU surgery, and 16% were on immunosuppressive medications. Fifteen of 102 PJI episodes (15%) were early PJI (<3 months), 44% were late infections (3 months-2 years), and 41% were delayed onset PJI (>2 years after implantation procedure). Five patients had a history of renal disease and four patients had recurrent UTIs, two of which were due to *P. aeruginosa*. The authors hypothesize that acquisition of *Pseudomonas* spp. as a colonizer in their patients occurred during prior surgeries or procedures. Thus, while acquisition of colonization from *Pseudomonas* spp. from the initial arthroplasty may be the cause of late or delayed onset chronic infections, other surgeries or procedures, patient comorbidities, other infections, or antibiotic exposure should be considered

when determining if empiric antipseudomonal antibiotic therapy is necessary in delayed or late onset PJI.

d. Does "bone penetration" of an antimicrobial agent matter clinically, and should it be used to select therapy?

Executive Summary

Bone penetration of antibiotic agents for the treatment of osteomyelitis is a frequently discussed yet poorly studied drug property (Table 3). There are numerous limitations that need to be considered when evaluating bone penetration studies. While it is intuitive that antibiotics cannot successfully treat an infection if they do not reach the site at a concentration sufficient to inhibit microbial growth, there are limited outcomes data for osteomyelitis to support this concept.

Table 3: Antibiotic Concentrations in Bone

Antibiotic	Time after Last Dose	Mean Bone Concentration (µg/g)	Overall Bone:Serum Concentration Ratio (range)	Bone:Serum Concentration Ratio (range)	
				Cortical	Cancellous
Levofloxacin[268-270]	0.7-2 h	3-7.4[268,269]	0.4-1	0.36-1[268-270]	0.5-0.9[268-270]
Ischemic bone[271]	NR	4.1-6.4	0.3-0.4		
Ciprofloxacin[272-274]	0.5-13 h	1.1-2.9[272,273]	0.3-1.2		
Ischemic bone[275]		NA	0.2-0.3		
Osteomyelitis[272]	1 h	1.4	0.4		
	2-4.5 h				
Ofloxacin[276-278]	0.5-12	0.3-1.1	0.09-1.0		
Moxifloxacin[270,279-281]	1.5 h	1.3-1.9	0.3-1.1	0.4-1.1	0.5-0.9
Azithromycin[282,283]	0.5-6.5 d	1.6-1.9	2.5-6.3		
Clindamycin[284-287]	1-2 h	0.6-3.8	0.2-0.5		
Ischemic bone[271]	NR	0.8-1.2	0.2-0.3		
Rifampicin[288-291]	2-14 h	0.7-5	0.08-0.6	0.2	0.2-0.4
Osteomyelitis[288]	3.5-4.5 h	5	0.6		
Tigecycline[292,293]	4-24 h[292]	0.08	0.4-2.0		
	4-24 h[293]	0.4	NR		
Doxycycline[284,294]	3 h	0.1-2.6	0.02-0.7		
Vancomycin[295-300]	0.7-6 h	1.1-10[296,298,300]	0.05-0.7	0.07[300]	0.1[300]
				0.2[300]	
	1-7 h	3.6-8.4	0.2-0.3[300]		0.3[300]
Osteomyelitis[300,301]				0.2*[301]	
	0-8 h[301]	NA	0.3-0.4		0.5*[301]
Ischemic bone[271]					
	NR	4.3-7.2			
Teicoplanin[302-305]	0.5-3.2 h	1.3-7.1	0.2-0.9		
	4-16 h	7	0.5-0.6		

Daptomycin[306,307]	8 h<br>0-16 h<br>0-24 h	3.3<br>NA<br>NA	0.09<br>1.1*<br>1.2*		0.09
Linezolid[308,309]<br><br>Infected bone[310]<br><br>Osteoarticular tuberculosis[311,312]<br>Ischemic bone[271]	0.5-16 h<br>2.5-24[313]<br>0.9 h<br>1.7-24 h<br><br>NR	8.5-9<br><br>NA<br>4<br><br>0.6-3.9<br><br>10.5-21	0.4-0.5<br>0.8-1.0*<br>0.2<br>0.4-0.5<br><br>0.2-0.3		0.2[310]<br>0.8-1.0*
Dalbavancin[314]	0.5 & 14 d	6.3 & 4.1	0.07 & 0.3		
Fusidic acid[315]<br>Osteomyelitis[316,317]	NR<br>1-13 h	12-25<br>NA	0.5-0.9<br>0.1-0.3		
Fosfomycin[318-320]	0.5-7 h	NA	0.1-0.5		
Trimethoprim-Sulfamethoxazole[321]	1-1.5 h	3.7/19	0.5/0.2		
Amoxicillin-clavulanic acid[322-325]	2 h<br>0.5-6 h<br><br>0.8-2.8 h<br><br>0.6 h<br>1 h	NA<br>NA<br><br>5.9-26 / 0.7-2.5<br><br>NA<br>NA / 17.5-32.5	0.2-0.3 / NR<br>0.03-0.07 / 0.01-0.09<br>0.08-0.2 / 0.04-0.08<br>0.2 / 0.1<br>NR / 1.1-1.8	0.1-1.8 (clavulanic acid)	0.1-1.1 (clavulanic acid)
Ampicillin-sulbactam[326-328]	0.25-4 h	12-20 / 5-7	0.1-0.7 / 0.2-0.7		
Piperacillin-tazobactam[329-331]	1 h<br>1.5 h<br>3 h	21.3 / 3.8<br>15.1-18.9 / 2<br>9 / 1.2	0.2 / 0.2-0.3<br>0.2-0.3 / 0.3<br>0.2 / 0.1	0.2 / 0.2-0.3	0.2-0.3 / 0.3
Flucloxacillin[302,332-334]	0.3-3 h<br>NR	2<br>NA	0.1-1.2<br>0.05-0.08		0.6
Cloxacillin[333,335]	1-3 h	2	0.1-0.6	0.1	0.2
Oxacillin[336]	1 h	2.1	0.11	0.1	
Methicillin[285,336,337]	1-2 h	3.1	0.04-0.2	0.2	
Ertapenem[338]	1.6-23.8 h	0.3-13.2	0.1-0.2	0.1	0.2
Meropenem<br>Ischemic bone[271]	 NR	 19.2-34	 0.7-1.2		
Cefazolin[339-342]	0.25-1.1 h<br><br>2.5-24 h[313]	4.7-32.3<br><br>NA	0.06-0.4<br><br>0.7-1.0*		
Cephalexin[343]	1.5-2 h	2.1	0.2		
Cefuroxime[295,328,344-348]<br><br>Osteomyelitis[348]	0.2-6.5 h<br>0.5-0.75 h<br>1 h	2-36<br>NA<br>15-28	0.09-0.6<br>0.01-0.1<br>0.04-0.08		

© 2022 Spellberg B et al. *JAMA Network Open.*

Cefadroxil[349]	2-5 h	NA	0.2-0.4		
Cefotaxime[350]	0.75-4 h	2.1-5.4	0.02-0.3		
Ceftriaxone[351-353]<br>Osteomyelitis[354]	0.2-24 h<br>1.5-8 h	2.2-20.9<br>9.6-30.8	0.07-0.2	0.05-0.08<br>0.08*<br>100% T>MIC for 24h	0.1-0.2<br>0.2*<br>100% T>MIC for 24h
Ceftazidime[355]<br>Ischemic bone[356,357]<br>Ischemic bone[271]	2 h<br>1-2 h<br>NR	20<br>3.1<br>2.6-3.7	0.5<br>0.04-0.08<br>0.1-0.2		
Cefepime[358]	1-2 h	35.6-52.5		0.5	0.8
Tobramycin[302,359]	0.3<br>14.3	NA<br>NA	0.1<br>0.09		
Gentamicin[334]	NR	NA	0.1		
AUC = area under the curve serum level<br>* = AUC_{bone}/AUC_{plasma} rather than serum					

Overall Summary

<u>Understanding the limitations</u>

The main limitation of most bone penetration studies results from the measurement of total antibiotic concentration in tissue homogenates. This technique disrupts the various compartments within bone and mixes the organic and inorganic bone matrices. Additionally, measuring total concentration does not provide a measurement of unbound drug which is the theoretical concentration of the drug that is available to exert an effect.[360] Uncertainty around these points could create problems from both an antimicrobial and pathogen perspective. Because therapeutic agents do not distribute within each compartment in an identical manner, total homogenate concentration does not represent the available concentration at the actual site of infection.[361] Likewise, pathogens such as *S. aureus* can survive differentially in various compartments within the bone, which again makes interpreting studies that used whole tissue homogenate problematic, as the concentration is not measured at the site of the invading pathogen.[362]

A second significant limitation of many bone penetration studies is the reporting of a single rather than multiple concentrations over time, making the measured concentration highly dependent on the sampling time. By failing to capture the dynamic nature between bone and serum concentration, any single value of a ratio of tissue vs. serum concentration is theoretically possible.[361] Lastly, taking this single value and comparing it to the minimum inhibitory concentration (MIC) of a pathogen to derive an ‘inhibitory quotient’ may lead to erroneous conclusions, as pharmacokinetics/pharmacodynamics (PK/PD) index values for antimicrobial agents are derived from serum rather than tissue concentrations that are obtained without regard to the time course of drug exposure.[361]

Additional limitations of bone penetration studies include variability in what the reported concentrations represent (i.e., mg/kg of total bone mass, organic mass, dry bone mass, total bone volume), small sample size compromising mostly of healthy patients with uninfected bone who receive a single dose of antibiotics prior to undergoing joint replacement, and the conduct of many studies before advances in sample preparation and bioanalytic methodology.[363]

© 2022 Spellberg B et al. *JAMA Network Open.*

Finally, an important point about interpreting bone penetration studies is that for an antimicrobial effect to occur, an absolute amount of drug must be present to inhibit microbial growth; relative ratios of drug in bone vs. blood do not necessarily translate to achieving necessary absolute levels in bone or not. For example, a drug with very low blood levels that achieves a high ratio of reported bone:blood concentrations (e.g., tigecycline) may still not achieve adequate absolute levels to inhibit microbial growth in bone. Conversely, a drug with very high blood levels but low ratio of bone:blood concentrations (e.g., ceftriaxone) may still achieve absolute levels in bone adequate to inhibit microbial growth in bone. All of these limitations should lead to the cautious interpretation of bone penetration literature.

Bone Penetration Studies

Four systematic reviews on antibiotic bone penetration have been published spanning the period of 1978-2018.[16,359,363,364] An additional literature search from 11/1/2018-4/30/2021 was conducted. Methodology for inclusion was similar to Landersdorfer and colleagues[364] in that only human studies were included, a minimum of five patients was required, bone:serum concentration ratios were calculated from reported mean concentrations if not otherwise calculated, and a bone density of 1 kg/L was utilized unless otherwise stated by the authors of the study. Table 3 summarizes the mean bone:serum concentration ratio for available agents which are reported as bone concentration (mg/kg) divided by serum concentration (mg/L). Where possible, the absolute levels in bone are listed as μg/g.

Overall, bone concentrations approach or exceed 50% of the serum concentration for the fluoroquinolones, azithromycin, tigecycline, clindamycin, linezolid, fusidic acid, and rifampin. The concentrations achieved in bone generally exceed the MICs of susceptible organisms except for tigecycline.[16,363] However, significant variability exists among specific agents and within classes. For example, the range in bone:serum and bone concentrations for doxycycline across two studies was 0.02-0.7 and 0.1-2.6 μg/g, respectively.[284,294] The low end of the range would not exceed the MIC_{90} of *S. aureus*. Landersdorfer and colleagues offered disruption of circulation in fractures or slow equilibration between plasma and bone as possible explanations, although the precise reason for the discordance is not fully understood.[364] Slow equilibration time between bone and plasma may also account for variability observed with ciprofloxacin, although study results showing an increase in bone:serum ratio over time are conflicting.[273,274,364]

Cephalosporins, penicillins, and carbapenems generally achieve bone concentrations of 5%-25% of serum. While these agents have a low reported bone:serum ratio, serum levels are high and, as a result, the actual concentrations in bone are quite high, and intravenous agents among these classes are likely to achieve concentrations in excess of the MIC for most susceptible pathogens. Bone concentrations with oral β lactams are more variable and may be less likely to exceed the MIC of specific organisms. Oral flucloxacillin, for example, has demonstrated adequate concentrations to exceed the MIC of *S. aureus* in one study while failing to achieve measurable bone concentrations in a second study.[333,365] Likewise, oral administration of cefuroxime did not result in measured concentrations while intravenous delivery achieves acceptable bone concentrations.[344-348,365]

Vancomycin and daptomycin are generally thought to penetrate bone poorly with serum to bone concentrations of ~5%-30% and <10%, respectively. Bone concentrations for both agents would, however, be expected to exceed the MIC_{90} for *S. aureus*. Concentrations of vancomycin may not exceed the MICs for individual enterococcal strains.[359]

© 2022 Spellberg B et al. *JAMA Network Open.*

Table 3 also presents bone:serum concentration ratios for cortical and cancellous bone. Landersdorfer et al. reported a non-significant, numerically higher median bone:serum concentration in cancellous vs. cortical bone (bone:serum ratio 0.25 and 0.16 for cancellous and cortical bone, respectively; $p = 0.06$).[364] Again, there was significant variability in ratios within classes and agents. Among β lactam agents, cefepime, ceftriaxone, ertapenem, and piperacillin-tazobactam had higher penetration and concentrations in cancellous compared to cortical bone.[359,364]

A minority of agents have been investigated in the setting of ischemia. Compared to studies in non-ischemic bone, the reported penetration and concentrations into ischemic bone are generally decreased.[275,356,357] However, the effect of ischemia is not consistent across all agents. Lozano-Alonso et al. studied 46 patients who had received at least four doses of antibiotics for an infection in the setting of limb ischemia necessitating major amputation.[271] Four measurements of transcutaneous pressure of oxygen were conducted ranging from the thigh, which had the best perfusion, to the distal foot, which had the worst perfusion, with a measurement in the chest being the control. A serum sample as well as bone biopsies at each of the three lower limb sites were obtained. Clindamycin, vancomycin, and meropenem showed decreased bone:serum ratios as ischemia worsened, while linezolid, levofloxacin, and ceftazidime did not show decreasing ratios. Except for clindamycin, all agents would have achieved bone concentrations in excess of the MIC for typical target pathogens.

Three studies across four agents were identified that utilized microdialysis techniques to obtain multiple bone concentrations over 24 hours that were then paired with serum concentrations.[301,307,313] This allowed for a comparison of area under the curve (AUC) concentration from bone to that of plasma. As mentioned previously, inclusion of concentration over time is a more robust measure as it accounts for distribution between compartments and provides concentration at the site of infection.

For example, Traunmuller et al. measured daptomycin bone concentrations in 10 patients with DFI who had received multiple doses of 6 mg/kg.[307] The 24-hour $fAUC_{bone}/AUC_{plasma}$ was 1.2 and equilibration between plasma and bone occurred within three hours of the infusion start. Cmax in the metatarsal bone was 4.7 μg/mL. A second study by Andreas et al. measured sternal bone concentrations in nine patients who received 6,000 mg cefazolin and 1,200 mg linezolid over a 24-hour period during which they underwent coronary artery bypass grafting with left mammary artery harvesting.[313] Mean bone concentrations of cefazolin were 112 μg/ml and 159 μg/ml while linezolid were 10.9 μg/ml and 12.6 μg/ml on the left and right, respectively. Mean cefazolin AUC_{bone}/AUC_{plasma} was 0.7 on the left and 1.0 on the right while linezolid penetration was 0.8 and 1.0 on the left and right, respectively. Lastly, Bue et al. measured vancomycin concentrations over 24 hours in 10 male patients undergoing total knee revision whom had received 1,000 mg vancomycin as antibiotic prophylaxis.[301] The AUC_{bone}/AUC_{plasma} ratio for vancomycin was higher in the cancellous bone, 0.5, compared to cortical bone, 0.2.

A mean concentration of 2 μg/mL but not 4 μg/mL was able to be achieved in cortical bone. In cancellous bone it took < 1 hour to achieve a mean concentration of 4 μg/mL with a Cmax of 10.6 μg/mL noted. Clinical outcomes were not provided in any of the studies.

Clinical Outcomes

Comparative Studies

Four trials of patients with chronic osteomyelitis have compared clinical outcomes of treatment with agents that have high vs. low bone penetration.[29-32] In these trials, an oral fluoroquinolone (ofloxacin or ciprofloxacin) was compared to either parenteral cephalosporins (cefazolin or ceftazidime) or antistaphylococcal penicillins with or without an aminoglycoside or clindamycin. Nonsignificant differences in clinical cures were observed in the fluoroquinolone group for all four trials (77%, 74%, 79%, 50% for fluoroquinolone vs. 79%, 86%, 83%, 68% for alternative therapy).[29-32]

Three additional sub-studies of DFO patients from larger DFI studies have compared agents with high bone penetration to those with lower bone penetration.[36,37,366] Two of three studies again showed lower but non-significantly different clinical cure rates in the groups with high bone penetration. In contrast, Lauf et al. reported a very low clinical response rate to tigecycline, which has low bone penetration, compared to ertapenem (32% vs. 54%).[366] Based on the range of MICs and MIC_{90} data presented in the paper for organisms such as, *E. faecalis* and MSSA, and bone concentrations reported in a prior PK study, tigecycline concentrations in bone were too low to exert an antimicrobial effect.[366]

In a second open label RCT, Lipsky and colleagues compared linezolid (oral or parenteral) to an aminopenicillin and β lactamase combination (ampicillin-sulbactam or amoxicillin-clavulanate) in patients with DFO, with a cure rate of 61% vs. 69%, respectively, the difference of which was not statistically significant [37]. Most patients were started on oral therapy and the predominant organisms were *Staphylococcus* spp. Given the low serum concentrations (Cmax 3.5-4.5 mg/L) and bone penetration, it is unlikely that bone concentrations of amoxicillin-clavulanate would have exceeded the MIC_{90} of *S. aureus* and coagulase negative staphylococci, yet clinical cure was high.[293]

High cure rates with predominantly amoxicillin-clavulanate in DFO were also shown in another recent RCT.[40] Finally, a trial comparing parenteral followed by oral ofloxacin to a combination of aminopenicillin and β lactamase inhibitor (ampicillin-sulbactam followed by amoxicillin-clavulanate) demonstrated a higher rate of cure/improvement in the ofloxacin group (75% vs. 60%, respectively).[36] The number of patients in both groups is small with only five patients in the aminopenicillin group. More patients in the ofloxacin group underwent bone debridement, although overall there did not appear to be a difference in cure/improvement between patients who underwent bone debridement vs. those that did not (73% vs. 67%, respectively).

While there are limited data on oral administration of β lactam antibiotics other than amoxicillin-clavulanate, several case series in the 1970s and early 1980s were published on the use of cephalexin in chronic osteomyelitis. Cephalexin has low bone penetration (0.2) and concentrations in bone (1.3-3.1 mg/L), which would not be expected to exceed the MIC_{90} of most organisms.[343,367] Nonetheless, satisfactory clinical response was noted in these case series, ranging from 79%-85%.[368-371]

In summary, there are insufficient trial data to determine whether measured bone concentrations are sufficient to predict antibiotic activity. There are certainly examples, such as tigecycline, where low concentrations may have contributed to excess failures. However, data for amoxicillin-clavulanate and cephalexin would seemingly argue against the notion that low predicted bone levels result in failure, as success was achieved in several studies despite low predicted bone concentrations. Ultimately, it is treatment success in clinical trials that should be prioritized for selecting antimicrobial regimens. It may be reasonable to consider bone

concentrations in choosing antibiotics after first considering drugs with established efficacy in clinical studies.

e. Does adjunctive rifampin alter osteomyelitis treatment outcomes; for which organisms is rifampin therapy potentially useful, and if it is used, is there a preferred dosing?

Executive Summary

Numerous observational studies and three small RCTs found that patients with osteomyelitis, with or without a retained implant, had improved clinical success rates, due to reduced relapse, when treated with adjunctive rifampin (rifampin monotherapy is never advisable due to concerns about emergence of resistance on therapy). However, other observational studies and one small RCT did not find a benefit of adjunctive rifampin. Meta-analysis of the four RCTs suggests a benefit of rifampin therapy (Figures 1-2). However, given the small size of these studies and the heterogeneity in results, patient populations, rifampin dosing, and background antibiotic therapy, these data remain hypothesis-generating, and a Clear Recommendation cannot be made for or against such therapy. A large RCT is necessary to clarify or disprove efficacy. In the meantime, it may be reasonable to consider adjunctive rifampin therapy for osteomyelitis caused by gram-positive cocci or non-fermenting gram-negative bacilli, with or without a retained implant, in individual patients based on risk:benefit assessment. Such assessment should include the uncertainty of the efficacy data balanced against potential drug interactions and adverse events of rifampin. If used, the dosing of rifampin has varied widely in studies. However, 450-600 mg per dose likely increases PD target attainment and adherence, and hence may be preferred, compared to 300 mg multiple daily dosing. Whether dose escalation to 900 mg once daily or 600 twice daily improves efficacy and/or worsens safety for treating osteomyelitis is unknown. To minimize emergence of resistance and treatment failure, it may be prudent to initiate rifampin only after bacteremia is cleared and surgical source control is achieved if it is necessary.

	Rifampin		Control			Risk Difference	
Study or Subgroup	Events	Total	Events	Total	Weight	M-H, Fixed, 95% CI	Year
Van der Auwera 1985	9	10	8	13	20.1%	0.28 [-0.04, 0.61]	1985
Norden 1986	8	10	4	8	15.8%	0.30 [-0.13, 0.73]	1986
Zimmerli 1998	12	12	7	12	21.4%	0.42 [0.13, 0.70]	1998
Karlsen 2020	17	23	18	25	42.7%	0.02 [-0.23, 0.27]	2020
**Total (95% CI)**		**55**		**58**	**100.0%**	**0.20 [0.04, 0.36]**	
Total events	46		37				
Heterogeneity: Chi² = 4.62, df = 3 (P = 0.20); I² = 35%							
Test for overall effect: Z = 2.52 (P = 0.01)							

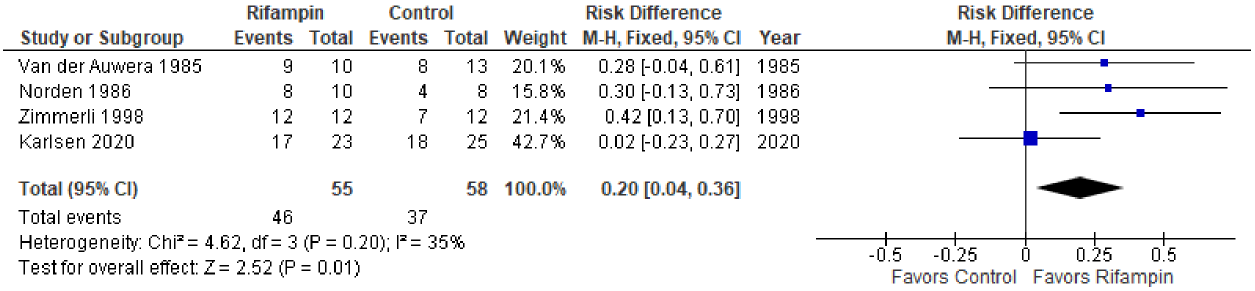

Figure 1: Forest plot of RCTs comparing success rates of patients treated with adjunctive rifampin or not for *S. aureus* osteomyelitis, with or without prosthetic implants.

The first two RCTs[372,373] included osteomyelitis without retained implants, while the third and fourth were of patients with PJI.[374,375] The first and third trials were double-blinded and placebo-controlled; the second and fourth were open label. All four RCTs used different rifampin dosing and frequency; the first and third trials administered rifampin in individual doses of 450 to 600 mg, which may be important to improve AUC serum levels, while the second and fourth trials administered 300 mg individual doses. Finally, the first, second, and fourth trials administered β lactam or vancomycin as the primary antibiotic, while the third trial administered a fluoroquinolone.

© 2022 Spellberg B et al. *JAMA Network Open.*

Osteomyelitis (non-PJI)

Study or Subgroup	Rifampin Events	Rifampin Total	Control Events	Control Total	Weight	Risk Difference M-H, Fixed, 95% CI	Year
Van der Auwera 1985	9	10	8	13	56.0%	0.28 [-0.04, 0.61]	1985
Norden 1986	8	10	4	8	44.0%	0.30 [-0.13, 0.73]	1986
**Total (95% CI)**		**20**		**21**	**100.0%**	**0.29 [0.03, 0.55]**	
Total events	17		12				

Heterogeneity: Chi² = 0.00, df = 1 (P = 0.95); I² = 0%
Test for overall effect: Z = 2.19 (P = 0.03)

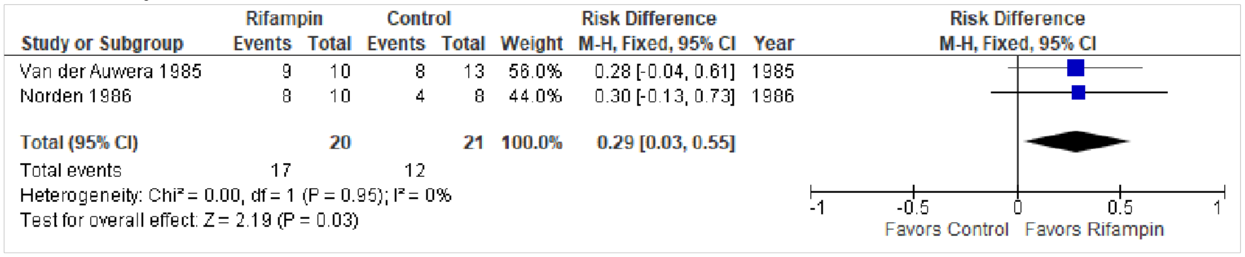

PJI

Study or Subgroup	Rifampin Events	Rifampin Total	Control Events	Control Total	Weight	Risk Difference M-H, Random, 95% CI	Year
Zimmerli 1998	12	12	7	12	48.4%	0.42 [0.13, 0.70]	1998
Karlsen 2020	17	23	18	25	51.6%	0.02 [-0.23, 0.27]	2020
**Total (95% CI)**		**35**		**37**	**100.0%**	**0.21 [-0.19, 0.61]**	
Total events	29		25				

Heterogeneity: Tau² = 0.06; Chi² = 4.32, df = 1 (P = 0.04); I² = 77%
Test for overall effect: Z = 1.04 (P = 0.30)

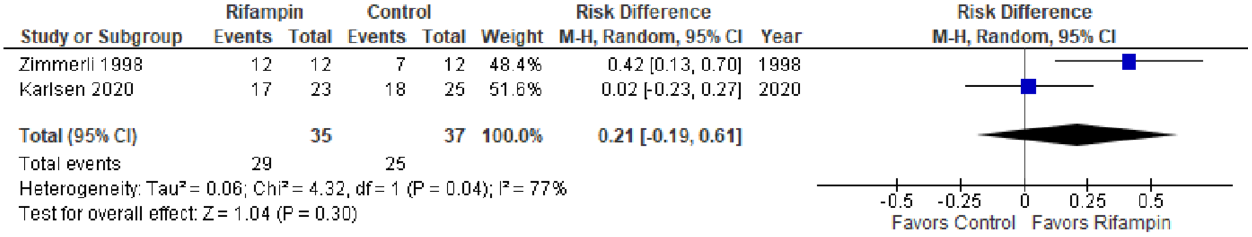

Figure 2: Forest plots of subsets of RCTs comparing rifampin vs. no rifampin for only osteomyelitis without PJI or only osteomyelitis with PJI.
No heterogeneity was found for the non-PJI subset, and a fixed effect model was used for this analysis. Significant heterogeneity was found for the PJI subset, and a random effects model was used for this analysis.

Potential Role for Adjunctive Rifampin

The primary potential role for rifampin in the treatment of osteomyelitis, with or without foreign body/implants, is as adjunctive therapy with another antibiotic to reduce the risk of relapse/long term clinical failure. Rifampin should not be used as monotherapy due to its low barrier to resistance.

Relapse is a common cause of long-term clinical failure of osteomyelitis treatment. Even with appropriate treatment, osteomyelitis has a long-term relapse rate of 10%-30%.[376-380] Observational studies have described even higher rates of failure, possibly up to 50% with long-term follow up, for infections caused by *S. aureus* treated with vancomycin or monotherapy fluoroquinolones, or for infections caused by non-fermenting gram-negative bacilli, such as *P. aeruginosa*.[16,374,381-387]

Although the precise pathophysiology of this high relapse rate is unknown, several lines of evidence suggest that slowly or non-replicating bacterial persister/small colony variants play a role.[379,388] First, relapses after monomicrobial osteomyelitis are well described after multiple decades, with several reports occurring even 50 to 80 years after the original infection (often caused by *S. aureus*).[389-394] It is difficult to conceive of bacteria actively replicating in bone for multiple decades with no resulting inflammatory response or signs or symptoms of infection. Such cases strongly suggest pathogenesis involving prolonged periods of a very slowly or non-replicating bacterial metabolic state in bone.

Second, with the exception of infections caused by *S. aureus* specifically treated with monotherapy quinolones,[16,374,385-387] relapsing strains have been reported to remain susceptible to the antibiotics with which the patient was originally treated.[379,388] Failure to develop resistance after exposure to antibiotics is a hallmark of non-replicating persisters, as these bacteria do not

© 2022 Spellberg B et al. *JAMA Network Open.*

express the biochemical targets of the antibiotic, and thus experience no selective pressure from the drugs.[395] Finally, studies of animal models and patients increasingly describe the role of small colony variants, which adopt a slowly or non-replicating phenotype, in *S. aureus* persistence during osteomyelitis.[396-402]

Rifampin is one of the few antibiotics that possesses the ability to reliably kill non-replicating persister bacteria.[396,403-407] Thus, there is a potential, biologically plausible basis for the hypothesis that adjunctive rifampin could help reduce the relapse rate for osteomyelitis, even in the absence of prosthetic material.

<u>Preclinical Data Suggesting Adjunctive Rifampin May Be of Benefit</u>

Consistent with this hypothesis, rifampin has been repeatedly shown to be more effective than a wide array of other antibiotics at eradicating bacteria from bone in preclinical models of infection, despite having less impressive activity than these other drugs during log phase, planktonic growth *in vitro*.[408-413]

<u>Observational Clinical Data Regarding Adjunctive Rifampin</u>

The preclinical data are mirrored by numerous observational or retrospective studies in patients. For example, among a cohort of patients who had had multiple relapses of osteomyelitis over 15 years or more, use of regimens that included adjunctive rifampin led to cessation of relapses in most patients.[410] The only relapses observed with adjunctive rifampin treatment occurred in patients infected with gram-negative bacilli (primarily Enterobacterales) that were resistant to the non-rifampin agent. Similarly, in a retrospective review of 35 patients with vertebral osteomyelitis, relapses occurred in 0/15 patients treated with adjunctive rifampin vs. 5/20 patients not treated with rifampin ($p = 0.048$).[414] More recently, a large retrospective cohort study from the Veteran's Health Administration found that patients with DFO treated with adjunctive rifampin had a significant reduction in long-term amputation and death compared to patients not treated with rifampin.[415]

Multiple studies have also found that patients with PJI had reduced relapses when treated with adjunctive rifampin treatment vs. not.[205,225,416-424] In each of the three largest of these retrospective studies, totaling more than 1,500 patients, by multivariate analysis, patients treated with adjunctive rifampin had significant reductions in relapse/late failure compared to patients not treated with rifampin.[205,418,419]

However, the data are mixed, as other observational studies have not reported significant differences in relapse rates in patients treated with adjunctive rifampin.[425-430] One meta-analysis of 13 observational studies of adjunctive rifampin therapy for PJI found no clear benefit, and emphasized that the individual studies were highly subject to selection bias.[431] A more recent meta-analysis of rifampin therapy for the treatment of staphylococcal PJI included one RCT which did not show benefit (discussed below) and 63 observational studies.[432] They reported that adjunctive rifampin use was associated with a relatively small but significant benefit, with a pooled risk ratio for effectiveness of 1.10 (95% CI, 1.00–1.22). However, the analysis was subject to the same concerns about significant heterogeneity, and several types of bias. Thus, even meta-analyses of rifampin effect based on observational studies have conflicted in their conclusions.

<u>RCTs of Adjunctive Rifampin Therapy</u>

Three small RCTs have demonstrated potential therapeutic benefit of adjunctive rifampin therapy for osteomyelitis with or without PJI.[372-374] In the first, double-blinded, placebo-controlled trial by Van der Auwera et al., 101 patients with invasive *S. aureus* infection, of which 23 had biopsy-confirmed osteomyelitis, were randomized to treatment with oxacillin plus rifampin (600 mg twice daily) vs. oxacillin plus placebo.[372] Clinical success among the osteomyelitis patients occurred in 90% (9/10) treated with rifampin vs. 62% (8/13) treated with placebo (Figures 1-2). In the second, open-label trial by Norden et al., 18 patients with *S. aureus* osteomyelitis were randomized to nafcillin plus weight-based rifampin (300 twice daily, 300 thrice daily, or 600 twice daily for <50 kg, 50-74 kg, >74 kg, respectively) or nafcillin alone (no placebo).[373] Treatment success rates were 80% (8/10) and 50% (4/8) for the rifampin vs. control group. Finally, in the third, double-blinded trial by Zimmerli et al., patients with *S. aureus* osteomyelitis in the setting of PJI were randomized to receive ciprofloxacin plus either rifampin (450 mg twice daily) or placebo.[374] In the per-protocol population, cure rates were 100% (12/12) for rifampin-treated vs. 58% (7/12) for placebo-treated patients ($p < 0.02$). Of particular importance, four of the five treatment failures in the placebo arm were caused by relapse associated with the development of resistance to fluoroquinolones while on therapy (i.e., ciprofloxacin monotherapy). In contrast, no relapses, and no resistance, were detected in the adjunctive rifampin group.[374]

However, a fourth, more recent, open-label RCT of rifampin for PJI by Karlsen et al. had twice the sample size of the prior PJI RCT and showed no benefit of adjunctive rifampin therapy.[375] Forty-eight evaluable patients were randomized to adjunctive rifampin (dosed at 300 mg thrice daily) therapy or not. At a median of two years of follow-up, treatment success rates were 74% (17/23) in the rifampin arm and 72% (18/25) in the no rifampin arm. The Kaplan-Meier curve of time to failure did separate initially, but the difference waned as follow-up time elapsed.

Collectively across these four small RCTs, treatment success occurred in 84% (46/55) of patients treated with rifampin vs. 64% (37/58) not. By meta-analysis, the adjusted difference in success rate is 20% (95% CI, 4%-36%), $p = 0.01$, suggesting benefit (Figure 1). Subgroup analyses focusing just on osteomyelitis without PJI demonstrated treatment success rates of 85% (17/20) vs. 50% (12/21), with an adjusted difference in cure of 29% (95% CI, 3%-55%). For the PJI subgroup analysis, a random effects meta-analysis model was used due to significant heterogeneity across the two available RCTs. Composite treatment success rates were 83% (29/35) vs. 68% (25/37), for an adjusted difference in success rate of 21% (95% CI, -19% to +61%).

It must be emphasized that the dosing of rifampin varied across the four studies, the primary antibiotic varied across the two trials involving PJI (β lactam in one study, ciprofloxacin in the other), and that two studies were double-blinded whereas two were open-label. Hence variations in trials results could be due to small sample sizes, resulting in overlapping confidence intervals, or to drug selection and dosing variances, or other patient- or provider-assessment variations.

Cumulatively, these RCTs provide some support for the hypothesis that adjunctive rifampin therapy may be of benefit for both for osteomyelitis and PJI. However, all four RCTs were small, with different patient populations, antibiotic regimens, and designs, and one was discordant. Ultimately, a large RCT is needed to provide a hypothesis-confirming level of evidence either for or against rifampin benefit for osteomyelitis.

© 2022 Spellberg B et al. *JAMA Network Open.*

Dosing Considerations

If rifampin is administered to patients with osteomyelitis, there are no clinical outcomes data to guide optimal dosing. However, at doses of approximately 450 mg, rifampin biliary clearance becomes saturated, such that greater than proportionate increases in serum levels occur above that individual dose.[22,23] Since the best predictor of rifampin efficacy is thought to be AUC_{24} divided by the MIC (AUC_{24}/MIC),[24,25] increasing serum levels has the potential to increase the chance of reaching target attainment to optimize outcomes. Indeed, for *S. aureus* infections in mice, an AUC_{24}/MIC ratio of >950 optimized outcomes.[25] This target was predicted to be difficult to achieve in a modeling study of human dosing, underscoring the need to optimize serum levels to treat bone infections.[26] Higher doses also result in higher peak levels, which may improve bone penetration. It may be advisable, therefore, to administer rifampin at individual doses above 300 mg to improve peak levels, AUC_{24}/MIC target attainment, and antimicrobial effects. However, no clinical data are available to confirm this hypothesis. Nevertheless, once daily dosing is also easier for patients. For these reasons, 600 mg once per day may be preferred to 300 mg twice or thrice daily, although, again, clinical data are not available to validate this assertion.

Whether or not dose escalation to 900 or 1200 mg per day (in divided doses) might be of benefit or result in excess toxicity, compared to a 600 mg total daily dose, remains uncertain. In three RCTs of dose escalation rifampin for the treatment of tuberculosis, the impact of dosing on microbicidal effects varied, but no excess toxicity was seen with higher dosing. Specifically, two RCTs found that doses of 900 mg once per day or 35 mg/kg per day resulted in more rapid declines in bacterial density than 600 mg once per day or 10 mg/kg/day.[433,434] However, in none of the three trials did higher doses (900 mg per day, 1200 mg per day, or 35 mg/kg per day) result in higher rates of ultimate microbiological eradication, nor improve clinical cure.[433-435] Nor were higher doses associated with a higher rate of adverse events in any of the studies.

Whether these results translate to treatment of osteomyelitis is unclear. However, in a large retrospective study of PJI, 450 or 600 mg twice daily of adjunctive rifampin was not associated with superior cure rates by multivariate analysis compared to 600 mg once daily.[205] Furthermore, in contrast to the trials of patients with tuberculosis, in the retrospective studies of PJI, doses above 10 mg/kg/day were associated with progressively higher adverse event rates.[205,420,436] Thus, a dose of 600 mg once per day may be a reasonable balance between safety and efficacy, although dosing to 8 to 10 mg/kg may be considered in heavier individuals.

When to initiate the rifampin and duration of rifampin therapy are also not certain. However, one observational study found that patients treated with adjunctive rifampin for >14 days resulted in reduced relapse rates compared <14 days.[420] It is reasonable to administer rifampin during the total duration of antimicrobial therapy, provided the drug is tolerated. Such a strategy may limit patient confusion about changing regimens. It may also be reasonable to hold initiation of adjunctive rifampin until bacteremia is cleared (if present) and source control is achieved to reduce the potential for emergence of resistance to rifampin. Indeed, in one large observational study in which rifampin use was associated with increased treatment success in the treatment of PJI, initiation of rifampin within the first 5 days of surgical debridement was independently associated with treatment failure as compared to starting the rifampin later.[205]

Clinicians should be cautious of co-administering linezolid and rifampin due to a pharmacological interaction that lowers linezolid bioavailability and resulting blood levels via a variety of mechanisms.[437,438] This interaction has been associated with increased failures in the treatment of PJI compared to treatment with either monotherapy linezolid or other drug

© 2022 Spellberg B et al. *JAMA Network Open.*

combinations including rifampin.[439] Similarly, rifampin appears to lower clindamycin blood levels when the latter drug is co-administered orally (but not intravenously),[440-442] and also may lower fusidic acid levels.[443] Nevertheless, two studies from a single center in Australia have reported high levels of treatment success with rifampin plus fusidic acid for PJI treated with DAIR.[444,445] Similarly, rifampin may lower trimethoprim levels, but without compelling evidence that the combination decreases clinical efficacy.[446,447]

Organism Considerations

Most data for adjunctive rifampin therapy come from the treatment of *S. aureus* infections. However, rifampin is broadly active, observational studies have described superior outcomes in patients with streptococcal osteomyelitis and PJI treated with adjunctive rifampin,[419,421] and there is a biologically plausible basis for its use as adjunctive therapy for other gram-positive organisms and for non-fermenting gram-negative bacilli (e.g., *Pseudomonas* and *Acinetobacter*), the latter of which have particularly high relapse rates.[448-450] It is less clear that rifampin would be appropriate as an adjunctive agent for Enterobacterales, which tend to have substantially higher rifampin MICs, and given relapses of Enterobacterales despite adjunctive rifampin therapy in one small, uncontrolled study.[410]

Summary

There is a biologically plausible mechanism by which adjunctive rifampin may be of advantage for reducing relapse for osteomyelitis with or without foreign body implants: killing of slowly replicating or non-replicating/small colony variant strains in bone. Multiple preclinical models by many groups over several decades have found that rifampin is a more effective antimicrobial at sterilizing bone infections than any other antibiotic tested. Some observational clinical studies further support the potential for adjunctive rifampin therapy to reduce relapse rates. Finally, three small RCTs, each of which were individually under-powered and had different trial populations and designs, were concordant, suggesting that patients randomized to receive rifampin therapy had improved treatment success rates/reduced relapse rates. However, the data are mixed, as other retrospective studies and one small RCT of patients with PJIs are discordant. When these disparate trials were meta-analyzed, the results suggest potential benefit of rifampin, although not to the level of hypothesis-confirmation.

If adjunctive rifampin is to be used, its potential benefit should be balanced against its known toxicities and drug interactions when making a risk:benefit decision in individual patients. A large RCT is necessary and desired to provide more definitive, confirmatory evidence. In the meantime, it is reasonable to consider the use of rifampin for this purpose in individual patients, with careful risk:benefit considerations, but its use should not be considered standard of care.

Rifampin should not be used in patients with concomitant medications that would pose risks for serious drug interactions, and hence medication reconciliation/rationalization and assessment for drug interactions should always be conducted before initiation of rifampin therapy. Similarly, patients with active liver disease may not be appropriate candidates for adjunctive rifampin therapy. Given the remaining equipoise on risk:benefit for rifampin in this setting, involving the patient in shared decision-making, documentation of the reasons supporting its use, and its potentially favorable risk:benefit ratio in individual cases, is a prudent step.

© 2022 Spellberg B et al. *JAMA Network Open.*

f. What is the role of long-acting glycopeptide antibiotics?

Executive Summary:

The two long-acting glycopeptides available on the market, dalbavancin and oritavancin, are not licensed for the treatment of osteomyelitis, but are licensed for the treatment of acute bacterial skin and soft tissue infection (ABSSI). One RCT of dalbavancin (n = 70 patients) vs. standard of care, which was largely vancomycin (n = 10), showed similar cure rates for non-vertebral osteomyelitis without prosthetic material, and a shorter length of hospital stay in the dalbavancin arm. No other randomized trial data are available for long-acting glycopeptides and osteomyelitis. Multiple, small, single-center, observational studies (all n < 50) have reported similar outcomes with both dalbavancin and oritavancin and comparator regimens. Few safety concerns were raised in these studies, and the glycopeptides were rarely stopped due to adverse events. There are currently no data to suggest that long-acting glycopeptides would have superiority over other regimens, including oral therapy options. Thus, based on available evidence, the most likely role for long-acting glycopeptides in osteomyelitis is for patients with non-vertebral osteomyelitis: a) who are unlikely/unable to take an oral regimen, or b) where an oral regimen is contraindicated (e.g., due to resistance patterns). There is minimal evidence of long-acting glycopeptide therapy for osteomyelitis in the presence of prosthetic material and for vertebral osteomyelitis, so caution is warranted in these settings.

Overall Summary

There are two long-acting glycopeptides currently available for routine use: dalbavancin and oritavancin.[451] Both of these are licensed by the US Food and Drug Administration (FDA) and European Medicines Agency (EMA) for the treatment of ABSSSI, but not for other indications at present. Both agents maintain serum and tissue concentrations for prolonged periods of time (days to weeks) due to the half-lives of the drugs (10-14 days).[452,453] PK data from a single study of dalbavancin suggested therapeutic levels of drug (against susceptible gram-positive pathogens) would be maintained for up to eight weeks in bone and plasma after two doses one week apart.[314]

Due to their convenient dosing and favorable PK, there is significant interest in the use of long-acting glycopeptides in osteomyelitis, with relevant studies summarized below.

<u>Randomized trial data</u>

In a phase 2 RCT, Rappo et al. randomized patients (n = 80) with a clinical-radiological diagnosis of osteomyelitis at a single center in Ukraine to dalbavancin or standard of care at a 7:1 ratio, meaning 70 patients received dalbavancin and 10 standard of care.[27] All patients had baseline debridement and open biopsy, with histology supportive of chronic osteomyelitis in around 60% of patients. Patients with vertebral osteomyelitis or infections associated with prosthetic material were not included. More patients had a diagnosis of diabetes in the standard of care arm (50%) compared to the dalbavancin arm (14%). Standard of care was largely IV vancomycin followed by oral linezolid or levofloxacin. The major pathogen was MSSA (43/80, 54%), with the next most common pathogen being coagulase-negative staphylococci (16/80, 20%), followed by a variety of other pathogens. Importantly, in the dalbavancin arm, 23/70 cultured pathogens were either gram-negative, anaerobes, or mixed pathogens in which we would not expect any reliable activity of dalbavancin (given that its spectrum of activity is limited to gram-positive organisms). Only 3/11 patients with gram-negative infection in the

dalbavancin group received adjunctive aztreonam. Notably, vancomycin was used in the standard of care rather than β-lactam therapy even though MSSA was the most common pathogen.

Multiple population endpoints were used in the trial, including a modified intention-to-treat (mITT) population consisting of those who had known or suspected gram-positive osteomyelitis, a clinically evaluable population (the subset that could be evaluated at day 42), and a microbiological mITT population that only included those that grew gram-positive pathogens from bone and/or blood. In all populations, dalbavancin was non-inferior to standard of care, with a 97% cure rate by day 42 vs 88% in the standard of care arm. Similar results were seen in other analyses. Length of stay was significantly reduced in those receiving dalbavancin (15.8 vs. 33.3 days).

In summary, dalbavancin appeared as safe and effective as a standard of care arm largely comprised of vancomycin as the primary treatment for gram-positive osteomyelitis without prosthetic material and excluding infections of the spine. Limitations include that the study was single-centered, small in size, and so vulnerable to baseline imbalances in patient characteristics, and the directed standard of care therapy could be considered suboptimal. Also, all patients received source control prior to enrollment, limiting generalizability outside of this setting.

We found no published RCTs of oritavancin for the treatment of osteomyelitis.

<u>Observational data</u>

There are a significant number of studies evaluating real world experience of dalbavacin for osteomyelitis[454-465] (reviewed in [28]). The majority of these were small (all n < 50), single center experiences from the US or Europe of dalbavancin in which a proportion of patients with osteomyelitis were included. They generally described similar clinical success of dalbavancin to standard of care regimes. In two studies, lower clinical success was identified, although both of these were small (n = 7[460] and n = 11[458]) and included some patients who did not receive the full treatment course. In summary, the limited real world published experience supports dalbavancin having similar efficacy to other agents in the treatment of osteomyelitis.

Data are even more limited with oritavancin. Although there is a growing literature in skin and soft tissue infection, there was initial concern about higher rates of progression to osteomyelitis in patients with skin and soft tissue infections when treated with oritavancin in early clinical trials (0.6%, 6/796 with oritavancin vs. 0.1%, 1/983 with vancomycin; OR for osteomyelitis 7.4, 95% CI, 0.9-61.6; p = 0.06), leading the FDA to issue a package insert warning about the risk of osteomyelitis when treating skin and soft tissue infection.[466,467] However, all diagnoses were made within nine days of initiation of therapy, suggesting these patients may have had pre-existing, occult osteomyelitis rather than development of osteomyelitis on therapy.

A few small studies have been published on real-world experience of oritavancin in osteomyelitis.[457,468-471] Similar to the dalbavancin data, most patients achieved clinical cure, and in the matched studies, at similar rates to those treated with standard of care. The largest study included 134 patients across 20 different centers in six US states, with an 88% clinical success at the end of dose evaluation and with a similar proportion achieving longer term cure.[470]

<u>Adverse events</u>

In all studies, adverse events were relatively mild and rarely required treatment discontinuation.

Summary

The two long-acting glycopeptides on the market, oritavancin and dalbavancin, have a small amount of real-world evidence in the treatment of osteomyelitis. Nearly all studies were single-centered and retrospective, with a large number of varied comparator agents, definitions of disease, and definitions of cure. Adverse events with these agents were limited, and in the one RCT of dalbavancin, length of stay was much shorter in the dalbavancin group. There is no current evidence to suggest long-acting glycopeptides are more effective than other agents available for the treatment of osteomyelitis.

© 2022 Spellberg B et al. *JAMA Network Open.*

Question 5: Is oral therapy appropriate for the treatment of osteomyelitis, and if so, what are reasonable patient selection criteria for administration?

Executive Summary:

Eight published RCTs of more than 1,300 patients have demonstrated that oral antibiotic therapy is at least as effective as IV for the treatment of osteomyelitis, including with PJI (Figure 3).[17,29-35] Nine additional RCTs that compared oral antibiotics in both arms, using different drugs, or different durations of therapy, achieved high treatment success rates for vertebral osteomyelitis, DFO, and PJI, including those treated surgically with DAIR.[36-44] These RCTs are concordant with more than 40 observational studies (Table 4) and pharmacology data, collectively demonstrating that various oral regimens are reasonable therapeutic options for osteomyelitis. Conversely, no contrary data have been published establishing superior outcomes of IV vs. oral therapy for osteomyelitis.

We therefore recommend oral therapy for patients who: 1) are clinically stable (hemodynamically and at the site of infection, e.g., no spinal instability); 2) have adequate source control (i.e., not requiring further procedural drainage and no persistent bacteremia); 3) are likely to absorb oral medications from a functioning GI tract; 4) have an available regimen used in published studies to cover likely target pathogens; and 5) have no psychosocial reasons that preclude the safe use of oral therapy. Fluoroquinolones and TMP-SMX have the most published data in adults, with clindamycin and linezolid also used in multiple studies; amoxicillin-clavulanate has been the most frequently used in studies of DFO. It may be prudent to avoid monotherapy with a fluoroquinolone to treat staphylococcal osteomyelitis due to a high rate of relapse and emergence of resistance on therapy; there is insufficient evidence regarding delafloxacin as of 2021 for the use of osteomyelitis. Use of other agents (TMP-SMX, linezolid, clindamycin) or adjunctive rifampin with a fluoroquinolone are reasonable alternatives. The role of oral β lactams (except for amoxicillin-clavulanate for DFO), doxycycline, fusidic acid, and fosfomycin are less established (particularly for the oral sachet powder formulation of fosfomycin in the US, for which there are no data for osteomyelitis), but they may be reasonable options in specific cases, depending on availability in different countries.

	Oral		IV			Risk Difference	
Study or Subgroup	Events	Total	Events	Total	Weight	M-H, Random, 95% CI	Year
Greenberg 1987	7	14	11	16	1.2%	-0.19 [-0.53, 0.16]	1987
Mader 1990	11	14	10	12	1.7%	-0.05 [-0.35, 0.25]	1990
Gentry 1990	24	31	22	28	3.3%	-0.01 [-0.22, 0.20]	1990
Gentry 1991	14	19	12	14	2.1%	-0.12 [-0.39, 0.15]	1991
Gomis 1999	11	16	8	16	1.3%	0.19 [-0.15, 0.52]	1999
Schrenzel 2004	18	22	11	17	1.9%	0.17 [-0.11, 0.45]	2004
Euba 2009	17	21	21	27	2.8%	0.03 [-0.20, 0.26]	2009
Li 2019	457	527	450	527	85.6%	0.01 [-0.03, 0.06]	2019
**Total (95% CI)**		**664**		**657**	**100.0%**	**0.01 [-0.03, 0.05]**	
Total events	559		545				

Heterogeneity: Tau² = 0.00; Chi² = 4.74, df = 7 (P = 0.69); I² = 0%

Test for overall effect: Z = 0.61 (P = 0.54)

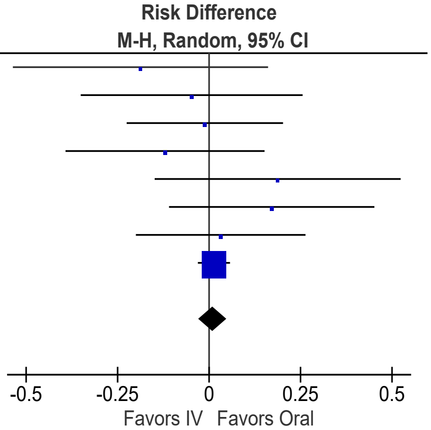

Figure 3. Random effects meta-analysis forest plot of RCTs comparing long-term clinical success rates of oral vs. IV antibiotic therapy for osteomyelitis in adults.[17,29-35] Reproduced with permission from the American Journal of Medicine.[18]

Table 4. Treatment Success Rates in Observational Studies of Oral Treatment of Osteomyelitis with or without Infected Prosthesis in Adults

Drug	Dose/Duration	Follow up	Cure*	Comment	Ref.
Fluoroquinolones					
Ciprofloxacin	500-750 mg PO bid x 3-4 months	1 yr	81% (30/37)	All cured patients had foreign material removed; 1/3 underwent debridement	472
Ciprofloxacin	750 mg PO bid x 3-4 months	6 mo	91% (21/23)	Cure defined as resolved or improved	473
Ciprofloxacin	750 mg PO bid x 3 months	7-21 mo	65% (13/20)	15/20 previously failed therapy; 3 patients with sternal osteomyelitis; cured only 7/13 *Pseudomonas*; all debrided	474
Ciprofloxacin	750 mg PO bid x 2-4 months	1-17 mo	77% (17/22)	4 of the non-cured infected with *Pseudomonas*; 20 debrided	475
Ciprofloxacin	750 mg PO bid x 1-6 months	0-22 mo	48% (14/29)	7/12 *Pseudomonas* & 4/9 *S. aureus* cured	476
Ciprofloxacin or Nafcillin, Clindamycin, or Gentamicin	750 mg PO bid x 12-64 d<br><br>varying dose & durations	25-39 mo	11/14 (79%) ciprofloxacin vs. 10/12 (83%) IV therapy	Not randomized; patients were sequentially enrolled in the two arms	31
Ciprofloxacin	200 mg IV bid, then 750 mg PO bid	?	67% (6/9)	Unknown duration of treatment; 5/7 *Pseudomonas* cured	477
Ciprofloxacin	200 mg IV bid, then 750 mg PO bid	?	83% (10/12)	Unknown duration of treatment	478
Ciprofloxacin	500-1500 mg PO bid x 0.5-18 months	?	65% (22/34)	20/28 *Pseudomonas* eradicated microbiologically	479

© 2022 Spellberg B et al. *JAMA Network Open.*

Pefloxacin<br><br><br><br>Ofloxacin<br><br>Ciprofloxaci n	400 mg IV q 12 h x 4 doses, then 400 mg PO q 12 h<br>200 mg PO q8-12 h<br>500-750 mg PO q 12 h<br>All for 3-6 mo	?	76% (29/38)	All cured patients had foreign material removed; 1/3 underwent debridement; 88% (15/17) treatment success for gram-negative bacteria vs. 67% (14/21) for gram-positive	480
Ofloxacin	200 mg PO tid x 4-6 weeks	>6 mo	85% (98/115)	3/15 *Pseudomonas* and 5/74 *S. aureus* failed; 113 debrided	481
Ciprofloxaci n	750-1000 mg PO bid x 3 mos	12 mo	61% (19/31)	No benefit from higher dose; all had soft tissue, but not bone, debrided	482†
Ciprofloxaci n<br><br>Lomefloxaci n<br>Levofloxaci n	750 BID<br><br>800 BID<br>500 once daily	Variable, most > 1 year	2/5 (40%)<br><br>5/7 (71%)<br>9/15 (60%)	6 patients infected with *S. aureus* and 1 *Pseudomonas* relapsed	483
Ofloxacin<br>+<br>Rifampin	200 mg PO tid<br><br>300 mg PO tid<br>both for 6-9 mo	> 60 mo	35/49 (71%)	All infections of prostheses with *Staphylococcus*	484
Levofloxaci n<br>+<br>Rifampin	500 mg PO qd<br><br>600 mg PO qd both for >6 weeks	> 6 mo	18/25 (72%)	All had prosthetic bone implants; mean duration of therapy 5 months for those cured and 2.6 months for those who failed to be cured	485
Rifampin<br>+<br>(Ofloxacin or<br>Fusidic acid)	900 mg PO qd<br><br>200 mg PO tid<br><br>500 mg PO tid x 5 d, then PO	Average 24 mos (range 12-36 mo)	11/20 (55%)<br><br>11/22 (50%)	All patients had orthopaedic implants, only 14 of which were removed; patients were assigned to treatment arm by year of birth (ofloxacin for even years, fusidic acid for odd years)	486

© 2022 Spellberg B et al. *JAMA Network Open.*

	bid, both for >6 mo				
Rifampin + Fluoro-quinolone vs. Other	When used, rifampin at 20 mg/kg divided bid (not to exceed 1800 mg/d)	Average 44 +/- 32 mos	37/39 (98%)<br><br>vs.<br>40/59 (68%)	All had *S. aureus* prosthetic joint infections; 29 patients received rifampin in combination with non-quinolone antibiotics; in multi-variate analysis rifampin-quinolone combination had an odds ratio of 0.4 (0.17-0.97) for failure	417
Rifampin + Levofloxacin (prospective) vs. Historical cohort with variable antibiotics without vs. with Rifampin	Prospective rifampin at 900 mg PO qd x 3-6 mos	?	13/14 (93%)<br><br>vs.<br><br>34/56 (63%)<br><br>vs.<br>21/31 (68%)	All had retained prosthetic joints; by multivariate analysis, hazard ratio for treatment failure 1.0 for historical cohort without rifampin, 0.55 (0.25-1.26) for historical cohort with rifampin, 0.11 (0.01-0.84) for prospective rifampin cohort, p = 0.03.	416
**<u>Other Agents</u>**					
Rifampin + Various other antibiotics	600 mg PO qd x 6 mos	Variable	50% (7/14)	All cases refractory to prior therapy	410
Linezolid	600 mg PO q12 h	?	60% (45/89)	Compassionate use program	487
Linezolid	600 mg PO bid	Variable	77% (17/22)	Post arthroplasty (10), ortho trauma (8), other (4)	488
Clindamycin	50-150 mg PO q 6 h x mean 16 weeks	Variable	42% (5/12)		489
Clindamycin	600 mg tid	1 year	67% (31/46)	Combined with rifampin (37), fusidic acid (4), or fluoroquinolone (4), including 40% of patients with prosthetic	490

© 2022 Spellberg B et al. *JAMA Network Open.*

				infections	
Clindamycin	600 mg tid or qid by body weight +/- other antibiotics	3-6 weeks	83% (111/133)	Clindamycin alone (31), with rifampin (27), levofloxacin (61), other (51)	491
TMP-SMX	1-2 DS tab PO bid	?	83% (5/6)	None had debridement	492
TMP-SMX	1 DS tab PO bid x 4-8 weeks	11-70 mos	45% (30/66)	55% of patients had debridement	493
TMP-SMX + Rifampin	3.5 mg/kg (TMP) PO bid<br>600-1200 mg PO qd both x mean 5 weeks	6 mo to 5 yrs	100% (27/27)	All patients had debridement	494
TMP-SMX +/- Rifampin	DS PO BID<br><br>300-450 mg PO bid both for median 10 weeks	2 years	82% (28/34)	10 patients had debridement, all of whom were cured	495
TMP-SMX	5 mg/kg (TMP) PO bid x 6-9 mos	24-75 mos	67% (26/39)	11 patients had device removed	496
TMP-SMX	Dose unclear, treated for 6 mos	12-60 mos	98% (59/60)	All patients had debridement	497
TMP-SMX	4-6 mg/kg (TMP) PO	6-7 wks	78% (40/51)	76% with prosthetic infections, 47% caused by gram-negative bacteria	498
(TMP-SMX or Linezolid) + Rifampin	8 mg/kg (TMP) PO 600 mg bid<br><br>10 mg/kg bid all given iv x 1 week and then	≥12 mos	89% (37/41)<br><br>79% (29/38)	20 patients with chronic osteomyelitis and 56 with orthopaedic implant infections; mean (range) treatment durations were 15 (1-53) weeks for TMP-SMX based therapy and 18 (8-36 weeks) for linezolid-based therapy; adverse event rates similar	499

© 2022 Spellberg B et al. *JAMA Network Open.*

	oral			(46% vs. 43%), discontinuation rates similar (14% vs. 21%)	
Fosfomycin	10g x 1, then 5 g tid	5-28 days	47% (29/60)	Outcome defined as "very good", mean 37 month follow up	500
Fosfomycin	4 to 8 g per day	IV or PO	29/37 (78%)	23 debrided	501
Fosfomycin	8 to 16 g IV, then 2-4 g PO per day	IV or PO	99/99 (100%)	39 debrided, started IV or IM, then transitioned to oral	502
Fusidic acid	Varied	PO, varied	73/80 (91%)[‡]	Review of numerous case reports and small case series	503
Fusidic acid	20 mg/kg	PO	19/20 (95%)	15 received other antibiotics with fusidic acid, 5 fusidic acid alone	504
**Diabetic Foot Osteomyelitis**					
Clindamycin, Amoxicillin/ clavulanate, Metronidazole, Fusidic acid, Ciprofloxacin,	Oral with some IV lead in	Varied	17/22 (77%)	Varied treatment, varied durations	505
Floxacillin, Amoxicillin/ clavulanate, Cephalo-sporins, Flouro-quinolones, Clindamycin, Metroniazole	Varied	Oral with IV lead in	35/50 (70%)	Treatment with a mean of 3 weeks IV followed by 6 weeks oral	506
Ofloxacin + Rifampin	200 mg PO tid + 600 mg PO bid	Oral	13/17 (76%)	Treated for 3 to 10 months	507

Metronidazole, Flouroquinolones, TMP-SMX, Amoxicillin/ clavulanate, Clindamycin, Cephalexin	Varied	Oral (with some IV lead in)	75/93 (82%)	Culture guided antibiotics, mean duration 6 weeks	508
Amoxicillin-clavulanate, Flouro-quinolones, Clindamycin, TMP-SMX, Rifampin	Varied	Oral (with some IV lead in)	264/339 (78%)	Numerous regimens used, however, amoxicillin-clavulanate was the most common (N = 301)	509

*Definition of cure varied among the studies.
†This was a randomized study of ciprofloxacin at 750 mg vs. 1000 mg twice per day. Because no comparator therapy was utilized, it is included in the non-randomized study section. DS = double strength tablet.
‡Based on a literature review, total case numbers >80, but difficult to count precisely from the review.

© 2022 Spellberg B et al. *JAMA Network Open.*

Table 5. Summary of Oral Antibiotic Doses Used in Published Studies for Osteomyelitis

Drug	Dose	Comment/References
Ciprofloxacin	500-750 mg BID	• Higher dose for *Pseudomonas* [29-31,38,472-478,482,483]
Levofloxacin	750 mg once daily	• Levofloxacin dosing based on [41,416,510]; L-enantiomer of ofloxacin, the latter of which was widely studied for osteomyelitis[36,480,481,484,486]
TMP-SMX	7.5-10 TMP mg/kg/d divided twice or thrice daily (e.g., 2 DS tablets twice daily for a 70 kg adult)	• Most studies used 7.5-10 mg/kg/d, [35,38,494,496,499]; 2 studies[493,498] used 4-6 mg/kg/d, with lower cure rates in one of them[493]
Clindamycin	600 mg TID; 900 TID or 600 QID for larger patients	• 450 mg QID may be used but was not favored in published studies[490,491,511]
Linezolid	600 mg BID	• Standard dosing,[37,487,488,499] monitor for reversible hematotoxicity after 2 weeks, and irreversible neurotoxicity after 4 weeks
Amoxicillin/ Clavulanate	500 mg TID or 875 mg BID	• Specifically for DFO[36-38]
Rifampin	600 mg once daily	• Doses studied include 600 once per day.[35,41,410,485,494] 900 mg once daily[416,486] or 600 mg BID,[417,499] unclear if efficacy or toxicity differs<br>• 300 mg doses less desirable due to lower AUC levels and less convenience for patients[22,23]
Fosfomycin*	4 to 16 g per day	• Various doses studied with formulations available outside the US, not studied with the sachet powder formulation in the US[500-502]
*There are no published data for the treatment of osteomyelitis with the sachet powder oral formulation of fosfomycin available in the US		

© 2022 Spellberg B et al. *JAMA Network Open.*

Overall Summary:

Observational Data for Osteomyelitis Including DFO and PJI

The historical basis of requiring IV therapy for the treatment of osteomyelitis was the relatively poor outcomes achieved with parenteral penicillin and aminoglycosides in the 1940s and 1950s.[512] During that early period in the history of antibiotics, the only drugs that were available for oral administration had limited bioavailability and/or spectra of activity (e.g., sulfanilamide, erythromycin, tetracycline). By the time advanced oral formulations of penicillin derivatives and other modern antibiotics became available in the late 1950s and 1960s, medicine had already long adopted a traditional, non-evidence-based culture of requiring IV-only therapy for osteomyelitis.

However, extensive pharmacology studies in the modern era have demonstrated that numerous oral antibacterial agents, including clindamycin, the fluoroquinolones, fosfomycin, fusidic acid, linezolid, metronidazole, rifampin, and TMP-SMX, achieve levels in bone with standard oral dosing that are well above the MICs of susceptible pathogens (as discussed in Section 4d). Oral amoxicillin may also penetrate into bone to achieve peak levels well above the MICs for sensitive gram-positive pathogens, at least when administered at a 1-2 g dose.[363] The data for other oral β lactams and tetracyclines are less clear regarding the reliability of exceeding target MICs in bone. However, clinical experience, discussed below, suggests that these drugs may be effective in some cases.

Concordant with the pharmacology data, more than 40 observational studies have demonstrated that oral administration of antibiotics resulted in treatment success rates for osteomyelitis similar to those historically experienced with IV therapy (Table 4).[16,483,488,490,510,513] These studies evaluated a wide variety of patients with adult osteomyelitis, including long bone, vertebral, skull based, DFO, and PJI.

The study drugs in the majority of these reports were fluoroquinolones, with or without adjunctive rifampin.[472-486,510] However, second in frequency of study has been TMP-SMX, which has been shown in at least seven observational studies of adults to be associated with excellent treatment success rates, again with or without adjunctive rifampin.[493-499] While the majority of these infections were caused by staphylococci, outcomes were similar in the remaining infections caused by streptococci or gram-negative bacilli. TMP-SMX has also been shown to be safe and effective for treating osteomyelitis in children.[514] Linezolid, clindamycin, and fosfomycin (the latter with an oral formulation available outside of North America; not studied with the sachet powder formulation) have also been studied as oral therapy, with reasonably high cure rates,[487-491,500,501,515] and clindamycin has been studied widely in children, including in an RCT,[516] with favorable outcomes.

Randomized Controlled Trials of Osteomyelitis Including DFO and PJI

Concordant with the observational data, eight RCTs in adults have unanimously demonstrated similar efficacy outcomes of oral vs. IV therapy in more than 1,300 patients with osteomyelitis, including with vertebral or long bone osteomyelitis and PJI (Figure 3).[17,18,29-35] Furthermore, in the largest RCT, patients receiving oral therapy reported better mobility, self-care, and activity levels, and less pain, discomfort, anxiety, and depression than patients receiving IV therapy.[17] Finally, cost was reduced by more than $3,500 for oral vs. IV therapy. Four of these RCTs explicitly included orthopaedic implants/PJIs, which constituted more than half of the enrolled

© 2022 Spellberg B et al. *JAMA Network Open.*

population in the largest RCT.[17,30,31,35] Vertebral osteomyelitis was also included in the largest RCT, comprising approximately 10% of the enrolled patients.[17]

In addition, nine RCTs have been published in which oral antibiotics constituted the large majority of therapy in both arms for the treatment of osteomyelitis, with excellent outcomes.[36-44] These RCTs compared different durations of oral therapy or different oral antibiotic agents, and included patients with vertebral osteomyelitis, DFO, and PJI, with only short IV-lead in periods before patients were switched to oral therapy.

For example, in an RCT comparing 6 vs. 12 weeks of therapy for 359 patients with vertebral osteomyelitis, more than 90% were treated with oral antibiotics in both the 6- and 12-week arms.[44] Treatment success rates were 91% in both arms and did not differ between patients treated with <1 week or >1 week of IV lead-in antibiotics.

In an RCT of patients with PJI, <1 week of other antibiotics were administered before patients were switched to oral levofloxacin at 750 mg plus rifampin 600 mg once daily. The per protocol (n = 44) treatment success rates were 92% and 95% in the short vs. long therapy arm at a median of 862 days of follow-up.[41] In a second RCT of PJI involving 123 patients, a mean of only four days of IV antibiotic lead-in was administered before patients were switched to oral therapy, which consisted of a variety of antibiotics, including a relatively even blend of quinolones, clindamycin, doxycycline, amoxicillin-clavulanate, and TMP-SMX.[42] Treatment success rates were 94% and 95% in the 4 vs. 6 week therapy arms. In a third, large RCT of 384 patients with PJI, the median duration of IV therapy administered was only nine days, with the remainder of therapy of 6 vs. 12 weeks of therapy being administered orally, with a wide variety of agents.[43] Treatment success rates were 83% and 93% in the 6- vs. 12-week arm at a median of two years of follow-up. Thus, not only do RCTs comparing outcomes of oral vs. IV therapy confirm oral efficacy with PJIs with little IV lead-in, but very high rates of treatment success have been seen in RCTs comparing durations of therapy predominantly administered orally.

Perhaps because of the long-standing, general acceptability of oral step-down therapy for DFO (dating back to the early to mid-1980s in observational studies), there have not been published RCTs comparing IV-only to oral therapy specifically for DFO. However, there have been RCTs that compared various oral regimens in both arms for treating DFO. Amoxicillin-clavulanate, clindamycin, or cephalosporins or fluoroquinolones with or without metronidazole or rifampin have been typically used in such studies, as described below.

For example, in 1997, Lipsky et al. randomized 88 evaluable patients with DFI to treatment with IV to oral step-down therapy with ofloxacin vs. ampicillin-sulbactam followed by amoxillin-clavulanate.[36] Twenty-six (30%) of these patients had osteomyelitis. The mean duration of IV therapy was approximately one week, followed by a mean of approximately two weeks of oral therapy for both arms. There was no significant difference in long-term treatment success between the two arms (85% vs. 83%), indicating that both options were reasonable.

In 2004, Lipsky et al. conducted another RCT comparing linezolid vs. ampicillin/sulbactam or amoxicillin-clavulanate in patients with DFI.[37] Treating physicians were able to decide the route (oral or IV) of administration, and this could change according to their judgment over the treatment course.[37] Of 361 study patients, 77 (21%) had osteomyelitis. The mean durations of treatment were 8 vs. 10 days IV, and 17 vs. 17 days total, for linezolid vs. the β lactam arms. The success rates for the osteomyelitis patients were 61% (27/44) vs. 69% (11/16) for the linezolid vs. β lactam arms.

In 2014, Lazaro-Martinez et al. randomized 46 patients with DFO to treatment with 90 days of antibiotics without surgical management vs. conservative surgical debridement (defined

as removal of infected bone without amputation) plus 10 days of antibiotic therapy.[38] All antibiotics were administered orally, consisting of twice daily ciprofloxacin 500 mg, amoxicillin-clavulanate 875/125 mg, or TMP-SMX 160 mg/800 mg. Overall outcomes were excellent, as treatment success was achieved in 86% (19/22) of patients treated with 10 days of antibiotics vs. 75% (18/24) of patients treated with 90 days of antibiotics. No difference in outcome was described by type of antibiotic.

Finally, two RCTs of longer vs. shorter therapy for patients with DFO were based on the use of oral therapy, with or without IV lead-in, with a wide variety of agents, including amoxicillin-clavulanate, quinolones, TMP-SMX, doxycycline, linezolid, with or without adjunctive rifampin.[39,40] Treatment outcomes were good in both arms of both studies, with overall 39/64 (61%) and 50/69 (72%) patients respectively achieving successful clinical outcomes.

Particularly striking is the absence of any contrary published data, whether observational or RCT, that demonstrates superior outcomes with IV therapy.[18] The numerous studies of oral therapy cannot necessarily encompass every conceivable iteration of osteomyelitis (body site, organism, resistance profile, type of foreign implant, patient factors, etc). Nevertheless, the data demonstrating safety and efficacy of oral therapy are sufficiently robust and concordant across numerous patient and disease types, including vertebral osteomyelitis, DFO, and PJI, that combined with the absence of any data indicating superior outcomes with IV therapy, they provide a reasonable basis for determining that oral therapy is a generally acceptable option when patients become clinically stable and can tolerate oral medications.

Published Antibiotic Selection

As for the observational data, the majority of the RCTs comparing oral to IV therapy studied fluoroquinolones with or without rifampin therapy, while one trial also specifically studied TMP-SMX plus rifampin.[35] However, as mentioned, in other RCTs comparing various oral regimens, myriad antibiotic types were administered. Overall, fluoroquinolones and TMP-SMX have the most published data. Clindamycin is also reasonable to consider based on observational studies, its inclusion in the largest oral vs. IV RCT as an option, its use in studies comparing different oral regimens for PJI, and extensive pediatric data.[17] Metronidazole is also a reasonable option for anaerobic coverage based on published observational data.[16]

Unfortunately, there is a high rate of relapse (in some studies more than 50%), typically with emergence to resistance, among osteomyelitis caused by staphylococci treated with fluoroquinolone monotherapy.[16,374,483] Thus, it seems prudent to avoid fluoroquinolone monotherapy in treating staphylococcal osteomyelitis. An alternative agent (e.g., TMP-SMX, clindamycin, linezolid), or addition of rifampin to fluoroquinolone therapy, are reasonable options.

Furthermore, fluoroquinolones may be less desirable than other available agents due to increasing reports of various potentially serious toxicities,[517,518] and the need to preserve them as oral step-down agents for broad gram-negative bacterial coverage. Thus, alternative options for gram-positive bacteria may often be preferred and, if the etiologic organism is likely susceptible to TMP-SMX, the latter may be a reasonable option for gram-negative osteomyelitis as well. Of course, TMP-SMX has also been associated with a variety of potentially serious adverse events (e.g., allergic reactions, hepatitis, hyperkalemia, etc.).[519]

Both linezolid and metronidazole (more commonly the former) may cause irreversible neuropathies after >4 weeks of therapy, and these agents should generally be avoided for

prolonged therapeutic periods if other agents or safer treatment options are available. If these agents are to be administered for more than 2-3 weeks, patients should be counseled regarding these potential side effects. Hematologic side effects of linezolid should be monitored for after two weeks of therapy.

There is less published experience with most oral β lactams, doxycycline, fosfomycin, and fusidic acid for the treatment of osteomyelitis. Amoxicillin-clavulanate is an exception; it has been widely used in the treatment of DFO, including in RCTs, as described above. Fosfomycin (again not studied with the sachet powder formulation available in North America) and fusidic acid have favorable bone PK and resulted in favorable outcomes in several published observational studies (Table 4). Furthermore, doxycycline is standard of care for treatment of some forms of atypical osteomyelitis, such as those caused by *Coxiella burnetii* and *Brucella spp.*[520-522] While this experience may not be directly extrapolatable to pyogenic osteomyelitis, it does support the concept that doxycycline can get into bone adequately to cure infection.

Pharmacological considerations may be less favorable for most β lactams (excepting amoxicillin) and doxycycline. Nevertheless, in the largest RCT, oral penicillins and doxycycline were each administered to more than 10% of patients, and outcomes were not described to differ in these patients.[17] Thus, limited data for oral β lactams, doxycycline, fosfomycin, and fusidic may suggest potential usefulness. Such data are not yet robust enough to enable a recommendation for or against their use, except for amoxicillin-clavulanate, which has resulted in high success rates in several RCTs for DFO. Nevertheless, given their spectra of activity, high potency against susceptible pathogens, and limited data in an RCT, other β lactam agents may be considered for individual patients.

All these oral options, including linezolid, are generic and relatively inexpensive. Hence, cost is generally not a relevant factor for selection among them (while it is decidedly a relevant factor favoring oral options over IV agents).

<u>Published Antibiotic Dosing (Table 5)</u>

In osteomyelitis studies, doses of ciprofloxacin have typically ranged from 500 to 750 mg twice daily (the latter especially for *Pseudomonas*).[29-31,472-478,482,483] Many of the fluoroquinolone studies were of ofloxacin, which has been replaced clinically by its active L-enantiomer, levofloxacin. A dose of 750 mg of levofloxacin once daily is reasonable for osteomyelitis.[41,416,510] TMP-SMX has generally been studied at approximately 7.5-10 mg/kg per day of trimethoprim in divided doses (e.g., two DS tablets twice daily for a 70 kg adult).[35,38,494,496,499] It has not been established that these higher doses of TMP-SMX are necessary to affect cure; in two studies doses of 4-6 mg/kg per day of TMP-SMX were administered,[493,498] but cure rates were notably lower in one of them.[493] Attention to renal function and potassium are important with higher doses of TMP-SMX and concomitant exposure to other agents which are potassium-sparing (e.g., angiotensin-converting enzyme inhibitors and angiotensin receptor blockers) should be evaluated and considered.[523]

General dosing of oral clindamycin has been 600 mg thrice daily, with escalation to 900 mg thrice daily or 600 mg four times daily in larger patients.[490,491,511] Linezolid has been studied at 600 mg twice daily.[37,487,488,499] If rifampin is to be administered, 600 mg once per day may be preferred to 300 mg twice daily or 450 mg once daily both due to dramatically superior AUC PK,[22,23,524,525] the fact that the PK/PD driver that best correlates with the drug's antimicrobial activity is total AUC/MIC,[24,25,526] and the simpler regimen of once vs. twice daily (discussed at

length in Section 4e). Whether dosing rifampin at higher levels (900-1,200 mg per day in divided doses) alters efficacy is unclear, although it may increase toxicity.

As discussed in question 4, section 3, caution should be taken if rifampin is to be administered with linezolid, due to a substantial pharmacokinetic interaction, lowering linezolid levels, which may be associated with a higher clinical failure rate.[437-439] Similarly, co-administration with rifampin may lower clindamycin and fusidic acid, although there is no evidence that clinical failures are more likely with these pairings.[440-445]

Amoxicillin-clavulanate has been dosed at either 500/125 mg thrice daily[36,37] or 875/125 mg twice daily[37,38] in individual studies for DFO (and some studies gave the option of either[37]), with no apparent distinction in outcomes. There are few published data for amoxicillin-clavulanate for osteomyelitis outside the context of DFO.

Of note, dosing of antibiotics should be adjusted for renal function or other clinical factors based on prescribing recommendations for each drug.

<u>Patient Selection Criteria for Oral Therapy</u>

Reasonable clinical criteria can be applied to select patients eligible for oral therapy. The duration of IV therapy prior to initiation of oral therapy has varied in the RCTs. In some studies, no IV lead-in was administered per the study protocol.[35,38-40] In the largest studies, a mean of only 9 or 10 days of IV therapy were administered before switching to oral agents for multiple subsequent weeks of therapy.[17,43] Thus, the cumulative data do not indicate that it is necessary to begin with IV therapy, nor for how long to administer it, before switching to oral therapy.

Nevertheless, patients who are clinically unstable (e.g., hemodynamically unstable, spinal instability, etc) should generally receive IV therapy, due to concerns about the ability to administer and absorb oral regimens and the desire to achieve more rapid therapeutic levels. Patients who will require procedural source-control typically require inpatient care, and often require withholding oral intake to prevent aspiration during procedures. Therefore, these patients are likely better suited to receive IV therapy. While RCTs have demonstrated efficacy of oral therapy for bacteremia and endocarditis,[18,527] persistence of bacteremia on therapy portends a poor prognosis, may indicate source control is needed, and likely necessitates ongoing inpatient care to ensure bacteria clear from the blood. Hence, IV therapy may be preferred until clearance of bacteremia. Finally, there may be psychosocial reasons why IV therapy is preferred in individual patients. For example, patients who are unlikely to be willing to take oral therapy, and/or who are otherwise likely to be in need of skilled nursing care, may benefit from IV therapy to help justify the higher level of care.

Collectively, therefore, it is reasonable to consider administration of oral antibiotics for the treatment of osteomyelitis when the patient meets all of the below criteria[18,527]:

1) clinically stable (e.g., hemodynamically stable, no spinal instability, etc);
2) does not require (or no longer requires) procedural source control and without persistent bacteremia;
3) is likely to absorb oral medications from a functioning GI tract;
4) there is an available regimen used in published studies to cover likely target pathogen(s); and
5) there are not psychosocial (e.g., need to justify specific levels of care, adherence concerns, etc.) reasons that preclude the safe use of oral therapy

Question 6: What is the role and optimal utilization of serial biomarkers and/or imaging studies for assessing treatment response in osteomyelitis?

Executive Summary: No RCTs have been performed to establish the role of repeated biomarker or imaging assessment in altering treatment decisions in patients with osteomyelitis. Retrospective/observational studies suggest that routine monitoring with inflammatory biomarkers (e.g., ESR, CRP) or serial imaging studies has poor predictive value for individual patients' long-term treatment success. Inflammatory biomarker activity changes with therapy and, at a population level, may be statistically predictive of relapse. However, it is not clear how the information biomarkers provide differs from clinical observations of success or failure over time at the individual patient level and, hence, how they might change management compared to clinical observation alone. Furthermore, their sensitivity and specificity are poor at predicting outcomes for individual patients (likelihood ratios <5), and these tests are thus of unclear benefit for altering care plans. Imaging studies, including MRIs, PET scans, and other nuclear imaging, are highly sensitive and often continue to show abnormal marrow signal for many months, including in patients who have achieved or ultimately will achieve treatment success. Therefore, there is no established role for routine monitoring of inflammatory biomarkers or imaging studies during treatment of osteomyelitis, either in the presence or absence of foreign bodies/implants. The only potentially identifiable roles for such studies are the use of repeat imaging in patients who are not responding to antimicrobial therapy, in order to determine if source control is needed, enable a reconsideration of the etiologic pathogen, or reconsider the accuracy of the diagnosis of osteomyelitis or PJI.

Overall Summary:

Observational Studies of Serial Biomarkers in Assessing Treatment Response in Osteomyelitis

ESR and CRP are the two most commonly used serum biomarkers in establishing diagnosis of osteomyelitis and its response to therapy in conjunction with clinical acumen, history or physical examination findings, and diagnostic imaging. Other serum biomarkers such as procalcitonin have not been shown to have better sensitivity.

No RCTs have been published to assess the impact of serial biomarkers on the treatment outcomes of osteomyelitis or joint infections. However, numerous observational studies of various designs, sizes, and quality have been published that evaluate the utility of such tests.

Vertebral Osteomyelitis

Among many studies, Carragee et al. conducted a retrospective chart review of 44 cases to describe the clinical use of ESR in monitoring outcomes for pyogenic vertebral osteomyelitis.[528] The 44 patients had ESR tested at or before time of diagnosis and at least twice during the following month. The study revealed a correlation of ESR with response to treatment, in that those with a decline in ESR were unlikely to have clinical failure. Indeed, a rapid decline of ESR (> 50% in the first month) was rarely seen in treatment failure. However, failure to decline did not predict failure. Indeed, by approximately two weeks after antibiotic treatment, 19 of 32 patients had ESRs that were actually higher than at the time of diagnosis. Yet these patients

went on to achieve clinical cure without surgery. Thus, the accuracy of the ESR at predicting who would fail and require some modification to the treatment regimen was poor. It is unclear how the test could alter therapy compared to clinical observation alone.

Similarly, in observational studies of 345, 79, and 38 patients with vertebral osteomyelitis, ESR and CRP levels were assessed and compared between patients who did or did not achieve long-term treatment success.[529-531] There was no relationship between ESR and CRP and risk of subsequent relapse at any time point.

In a study of 45 patients, Yoon et al. found that an ESR >55 mm/h and CRP > 27.5 mg/L at four weeks after antibiotic treatment was associated with a higher rate of treatment failure, defined as disease progression or recurrence, with an OR (95% CI) of 5.2 (1.0-26.6; $p = 0.04$).[532] However, the maximal sensitivity and specificity for ESR was 70% and 40%, respectively, and for CRP was 40% and 86%, respectively (+LR ≤ 3 and -LR ≥ 0.7). Thus, while the odds ratios for treatment success at the population level may have statistically correlated with short-term failure, accuracy of the tests for shifting post-test probabilities at the level of individual patients was poor.

Babouee Flury et al. conducted a retrospective study of 61 patients with vertebral osteomyelitis and found that the only independent predictor of switch to oral antibiotics was a lower CRP at two weeks compared with baseline, with an OR of 0.7 per 10 mg/L increase in CRP ($p = 0.041$).[533] Thus, CRP correlated with physicians' clinically-based assessments that patients were improving. Furthermore, nearly all patients achieved clinical response, whether their CRP fell or not. Again, it is unclear how biomarker information could have changed management.

Similarly, in a retrospective analysis of 21 patients with postoperative wound infections after spinal surgery, Khan et al. found that ESR levels did not correlate with clinical improvement.[534] However, the authors reported that CRP levels tracked well with clinical response: decreases in CRP levels paralleled patients clinically responding, while CRP levels remained elevated in patients who were clinically failing. Patients with clinical failure demonstrated persistent sinus tract drainage of pus while on therapy, required repeat surgical debridement, and/or had persistent erythema at the infection site. Yet, all of the clinically failing patients were known to be failing anyway based on physical exam, so it is unclear what new information the CRP added. In other words, CRP did not provide additional, practice-altering information over and above physical exam findings that were consistent with treatment failure.

DFO

Michail et al. conducted a prospective study to examine the performance of serum inflammatory markers in the diagnosis and monitoring of patients with DFI.[535] Of 61 patients (average age 63) with untreated foot infection, 27 had a diagnosis of osteomyelitis based on clinical exam and confirmed with imaging. The remainder (n = 34) were diagnosed with soft tissue infection only. Serologic markers (e.g., ESR, CRP) were obtained in all patients at baseline, one week, three weeks, and three months. At baseline, serologic markers were significantly higher in patients with osteomyelitis compared to those with soft tissue infection. After initiation of antibiotics, serologic markers declined. While it took, on average, seven days for CRP to return to near normal, ESR remained high until month three in those with underlying osteomyelitis. Unfortunately, outcomes are not described in this study, so it cannot be ascertained whether either marker accurately predicted clinical response to therapy, and in a manner distinct from clinical observation alone.

© 2022 Spellberg B et al. *JAMA Network Open.*

Tardaguila-Garcia et al. conducted an observational cohort study to analyze the predictive role of inflammatory markers in the healing time of DFO either managed by surgery or antibiotic treatment.[536] They found no correlation of inflammatory markers with healing time regardless of treatment group.

Van Asten et al. conducted a cohort study of 24 patients with DFO to determine if inflammatory markers could be used to monitor the treatment of DFI.[537] The biomarkers of interest included ESR, CRP, PCT, interleukins (IL-2, IL-6, IL-8), and TNF. The authors reported that CRP, ESR, PCT, and IL-6 levels significantly declined in the group with osteomyelitis after starting therapy. However, outcomes were not assessed, and it was therefore again not possible to determine how such levels could have altered outcomes or management compared to clinical observation alone.

In a second, longitudinal cohort study, Van Asten et al. evaluated trajectories of biomarkers, including ESR, CRP, and WBC count in 122 patients treated for DFO.[537] Initial inflammatory levels did not correlate with long-term outcomes. The authors found that CRP and ESR fell less rapidly in patients who had poor outcomes compared to those who healed with therapy. However, no formal ROCs were calculated, and the graphs demonstrate considerable overlap between the values over time, suggesting accuracy was low at distinguishing outcomes in individual patients.

<u>Non-vertebral Osteomyelitis</u>

Lin et al. sought to determine the association between both ESR and CRP and osteomyelitis recurrence.[538] They reviewed records of 81 males and 27 females with a median age of 54 years (range 10 to 87) who underwent antibiotic and surgical treatment for primary (n=68) or recurrent (n=40) osteomyelitis that was related (n=26) or unrelated (n=82) to a prosthesis. Of the 40 cases of osteomyelitis recurrence at a median 23.4 (range, 0.6-74.0) months of follow up, 7 and 33 were related and unrelated to a prosthesis, respectively. Risk factors for osteomyelitis recurrence were ESR ≥ 20 mm/h, infection with MRSA, and infection in the lower limb. Evaluating numerous cut-points of both ESR and CRP by regression analyses, they were able to find statistically significant relationships between individual test levels and hazard ratios of recurrence of osteomyelitis among the entire cohort. However, the sensitivity and specificity for both tests at predicting relapse in individual patients ranged from 50% to 85% (with most values being in the 60%-70% range), resulting in relatively poor +LR and -LR < 5 and > 0.6, respectively, at all cut-point values analyzed. Thus, irrespective of odds ratios for predicting the proportion of patients who would relapse across a population, the tests remained relatively inaccurate for predicting who would relapse among individual patients.

Faizal et al. reported on 51 adult patients with skull-base osteomyelitis, for whom ESR and CRP were ordered at initiation of therapy and at end of therapy, between week 6 and 8.[539] Upon completion of eight weeks of antibiotic therapy, 30 of the 51 (59%) patients were asymptomatic. Of these 30 patients, only three had achieved normal ESR and CRP values. Yet all 30 of these patients continued to be asymptomatic throughout the period of follow up, indicating the testing was not useful. Furthermore, the authors tried to establish best cut-off values for ESR or CRP, which, while still reflecting abnormal levels, had fallen enough that they could be considered indicative of treatment success. The best sensitivity and specificity they could achieve were 70%-80% and 60% (+LR and -LR <3 and >0.3), respectively, and the best correlation between ESR/CRP and PET scan was 60%-70%.

PJI

Ghani et al. examined the usefulness of CRP testing in determining whether a PJI had been treated successfully.[540] They found no difference between the mean CRP values of successful vs. unsuccessful treatment groups. Similar studies suggest that serial CRP monitoring is not reliable in determining infection specifically in two-stage revision procedures. Ghanem et al. sought to determine the usefulness of CRP as a test to determine the eradication of infection and the success in DAIR and single-stage revision.[541] The optimal ROC was 0.55 (poor capacity to distinguish), which was not statistically significant. They concluded that CRP often does not normalize even when the infection is eradicated.

Shukla et al. performed a study looking at 87 infected total hip arthroplasties treated with antibiotic spacer and six weeks antibiotics.[542] ESR and CRP were obtained before reimplantation. They came to a similar conclusion that ESR and CRP were not sufficiently rigorous tests and frequently remained elevated in patients whose infection had been eradicated.

Recently, Maier et al. evaluated the ESR:CRP ratio (ECR) as a marker for predicting infection resolution in 179 patients with acute PJI, acute hematogenous PJI, or chronic PJI who underwent DAIR.[543] The area under the ROC was calculated to evaluate ECR as a diagnostic marker for predicting postoperative reinfection in patients who underwent DAIR. Statistically significant differences in ECR were found in patients who underwent DAIR revision vs. total joint arthroplasty for chronic infection (1.23 vs. 2.33; $p = 0.04$). There was no significant difference in ECR in patients who underwent DAIR for acute infection ($p = 0.7$) and acute hematogenous infection ($p = 0.6$). In patients who underwent DAIR for chronic PJI, ECR demonstrated a sensitivity and specificity of 75% and 84%, respectively, for the prediction of postoperative reinfection, which was significantly higher than that of ESR alone (sensitivity, 67%; specificity, 47%; $p < 0.001$) or CRP alone (sensitivity, 50%; specificity, 26%; $p < 0.001$). Nevertheless, that superior accuracy still resulted in marginally useful likelihood ratios (+LR < 5, -LR > 0.3).

Finally, in one of the largest studies conducted to date, Bejon et al. also came to a similar conclusion.[544] They analyzed 3,732 serially obtained CRPs from 151 total joint arthroplasty patients (71 hip, 76 knee, and four elbow revisions) who had undergone two-stage revision for PJI, and 109 patients who had undergone DAIR (51 hip replacement, 50 knee replacements, and eight other joints). They reported that CRP values and changes in values were inaccurate at predicting treatment success, with poor ROCs. As Dr. Bejon and colleagues noted in their discussion, “CRP could not be recommended as a diagnostic test based on the sensitivity and specificity values indicated by ROCs. This does not reflect limited power of the study, but the wide scatter of individual readings in both outcome groups, as found in previous studies.”

Biomarker Summary

Collectively, the data are not compelling that inflammatory biomarker values over time can accurately predict osteomyelitis outcomes in a manner distinct from clinical observation, or inform a change in management to improve outcomes in individual patients. These lab tests may be used more for clinician psychological reassurance than to inform beneficial patient care decisions. If so, they are potential examples of wasteful, low-value care. Absent more compelling prospective data that demonstrate their ability to alter clinical decision-making in a manner that improves outcomes, we do not recommend their routine monitoring to assess response to therapy.

© 2022 Spellberg B et al. *JAMA Network Open.*

Observational Studies of Serial Imaging Studies in Assessing Treatment Response in Osteomyelitis

MRIs

In a study of osteomyelitis in children, 164 MRIs were ordered over time for 59 patients.[545] All repeat MRIs continued to show evidence of osteomyelitis due to abnormal marrow signal, including in patients who went on to treatment success. Of the 104 repeat MRIs (subtracting out the 59 baseline MRIs), 28 were ordered within the first two weeks of therapy, all due to "worsening clinical course." Eight (29%) of these resulted in a change in management. Of the remaining 76 repeat MRIs that were ordered after the first two weeks of therapy, only three (4%) changed management. Thus, 8/11 (72%) studies that changed management did so within the first two weeks of therapy. Overall, 10 of the 11 studies that changed management were triggered because of clinical signs and symptoms of failure of response to therapy. Thus, in this uncontrolled case series, MRI was informative only to confirm clinical suspicion of failure of response to therapy, and to guide changes in management; MRI was not informative as general surveillance in patients with clinical response.

In a case series of 79 patients with vertebral osteomyelitis who had repeat imaging, the median duration of antimicrobial therapy was 58 days.[529] The median follow up was 739 days. Imaging was repeated by physician choice at 4 to 8 weeks, likely triggered by clinical concerns of treatment failure. The finding of an improved MRI at 4 to 8 weeks was predictive of long-term clinical success, achieved in 26 of 27 (96%) patients with improved MRI. However, those patients were clinically improved anyway, so it is unclear how the MRI information could have changed management. Furthermore, when including patients with stable MRI findings at 4 to 8 weeks, the positive predictive value fell dramatically, to 47 of 65 (72%). Worsening MRI findings failed to reliably predict poor outcome, as only 5 out of 14 (35%) patients with worsening MRI at 4 to 8 weeks experienced clinical failure at long-term follow up. By univariate analysis, the strongest predictor of long-term treatment success was clinical improvement at follow-up.

In an additional study of 29 patients with vertebral osteomyelitis, all patients had baseline MRIs and repeat MRIs at three months, and 22 patients had additional repeat imaging at six months.[546] Antibiotic therapy was administered for an average of 14 weeks. All patients were described to have treatment success at 18 months of follow up. Nevertheless, abnormal MRIs, principally due to marrow edema, persisted in 67% of patients at three months of therapy, and in 15% at six months of therapy. None of those patients experienced clinical failure, and there were no differences in imaging studies in patients who had persistent pain or neurological sequelae from infection compared to those who did not. Finally, 30% of patients had epidural abscesses on imaging at baseline, and all had resolved by three months, in parallel with clinical response. Persistence of MRI bony abnormalities that did not predict clinical failure on subsequent imaging in improving patients has been described in multiple other case series as well.[547-549]

Imaging Studies: PET Scans, CT Scans, and Nuclear Imaging

Nuclear medicine scans have also failed to distinguish patients with osteomyelitis who went on to have long term treatment success from those who did not—generally because the tests were overly sensitive and continued to show bone abnormalities in responding patients.[550,551] Studies of CT scans have been inconclusive due to small sample size and no correlation of changes in radiographic results with long term treatment success.[552]

© 2022 Spellberg B et al. *JAMA Network Open.*

In a study of 51 adult patients with skull-base osteomyelitis, PET scans were obtained at initiation of therapy and at end of therapy, between weeks 6 and 8.[539] After completion of initial antibiotics, the PET scan was repeated every three months, until it was normal or the patient was asymptomatic and had normal ESR and CRP. Among the 21 patients who continued to have symptoms at eight weeks, nine were continued on antibiotic therapy for up to six months, and four received treatment for up to 15 months. Whether or not it was necessary to continue therapy because of positive PET scans could not be determined. Overall, this study found that biomarkers and PET scans did not predict clinical failure in patients who were clinically responding, did not correlate well with one another, and appeared to result in extreme prolongation of therapy in a subset of patients, without clear benefit.

In two studies totaling 35 patients with vertebral osteomyelitis who had serial PET scans, PET scan uptake tended to reduce on antibiotic therapy, consistent with response to therapy.[548,549] Yet, results significantly overlapped, making it impossible to distinguish someone adequately treated from someone who was not. Furthermore, the sample size, variable follow-up, and variable antibiotic treatments administered made it impossible to discern if the PET scan results precipitated a change in clinical outcomes.

In a third study of 38 patients with vertebral osteomyelitis, serial PET scans were more sensitive and specific than ESR or CRP, achieving approximately an 80% sensitivity and specificity for predicting "response".[530] That combination of sensitivity and specificity results in +LR and -LR of about 4 and 0.3, which reflects only a modest ability to change post-test probability. Furthermore, the definition of "response" was vague, defined as, "assessed during therapy on the basis of clinical status and inflammatory indexes." Yet, ESR and CRP may not be relevant to assessing therapeutic response. The only meaningful definition of success is: did patients achieve long term success without *clinical* evidence of relapse (of signs and symptoms of infection)?

Imaging Summary

Cumulatively, no data indicate that routine surveillance imaging of any type, including MRIs or PET scans, are clinically impactful, resulting in improved patient outcomes. Marrow signal abnormalities can persist for many months, even in patients who are successfully treated, and cannot accurately distinguish those who will achieve treatment success from those who will not. The use of imaging studies that are overly sensitive to monitor therapy may have the tendency to trigger inappropriately and unnecessarily long courses of antibiotic therapy, exposing patients to harm from drug side effects and selection for antibiotic resistance. The primary driver of clinical decision-making (e.g., whether to prolong antibiotics from a standard six-week course of therapy, whether to evaluate for the need for a source control procedure, etc) should be clinical response to therapy.

Therefore, we do not recommend routine serial imaging in patients with osteomyelitis to determine response to therapy. However, it is rational to repeat imaging in patients who are clinically not responding to antimicrobial therapy to evaluate the need for and feasibility of achieving source control, or to reconsider the initial diagnosis.

© 2022 Spellberg B et al. *JAMA Network Open.*

Question 7: What is the appropriate duration of therapy for typical cases of osteomyelitis?

Executive Summary:

Osteomyelitis (including DFO) without a Retained Implant

We recommend a maximum of six weeks of antibiotic therapy for hematogenous or contiguous osteomyelitis (including DFO), presuming adequate source control (i.e., no undrained abscesses too large to be treated with antibiotics alone, e.g., > 2-3 cm diameter) and no retained foreign body, reiterating that this guideline only addresses osteomyelitis caused by typical, pyogenic pathogens (Table 6). This Clear Recommendation is based on two RCTs demonstrating similar clinical outcomes for vertebral or DFO treated with 6 vs. 12 weeks of antibiotics (Figure 4).[39,44] Additional RCTs suggest that three or four weeks of therapy may result in comparable long-term treatment success rates as six weeks of therapy for debrided DFO[40] or osteomyelitis with removal of an orthopedic implant;[42] however, confirmatory data are needed. Finally, multiple observational studies and a small RCT[38] suggest that no antibiotics may be required, and demonstrate no advantage of antimicrobial durations >2-10 days, after total bone resection with clear margins. If the treating clinicians are confident all infected bone has been resected, we generally favor no postoperative antibiotics in this setting and do not recommend durations of more than five days.

Study or Subgroup	Shorter Events	Shorter Total	Longer Events	Longer Total	Weight	Risk Difference M-H, Random, 95% CI
Bernard 2015	160	176	159	175	96.0%	0.00 [-0.06, 0.06]
Tone 2015	12	20	14	20	4.0%	-0.10 [-0.39, 0.19]
**Total (95% CI)**		**196**		**195**	**100.0%**	**-0.00 [-0.06, 0.06]**
Total events	172		173			

Heterogeneity: Tau² = 0.00; Chi² = 0.47, df = 1 (P = 0.49); I² = 0%
Test for overall effect: Z = 0.12 (P = 0.91)

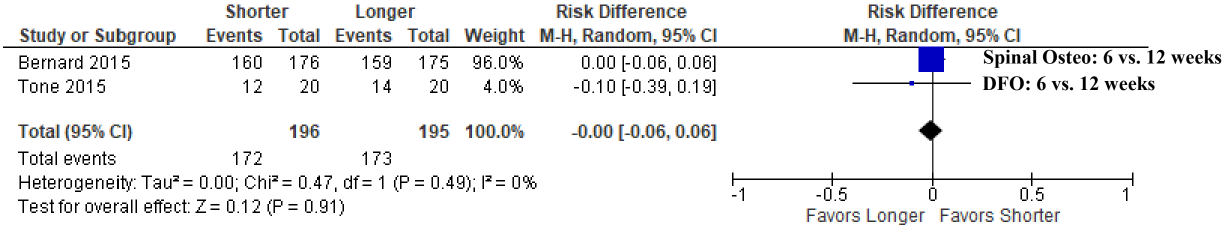

Figure 4. Random effects forest plot of RCTs comparing shorter vs. longer courses of antibiotic therapy for vertebral osteomyelitis and DFO in adults.[39,44]

Osteomyelitis with a Retained Implant (including PJI)

A large RCT of PJI (DATIPO) clearly demonstrated superiority of 12 vs. 6 weeks of antibiotic therapy for PJI.[43] Thus, participating experts unanimously agree that 12 weeks of therapy is preferred for PJI patients managed surgically with DAIR. However, the absence of a confirmatory, second study precludes making a Clear Recommendation, particularly in the prosthetic exchange cohorts. Based on the DATIPO RCT, some experts also clearly prefer 12 weeks of therapy for all prosthetic exchange PJI patients. However, most failures in the 6-week therapy arm occurred in the DAIR cohort, rather than the prosthetic exchange (1- and 2-stage) cohorts. Furthermore, there were more infections caused by *S. aureus* in the 6-week therapy arm. These accounted for 72% of the excess failures across all surgical management cohorts in the 6-week therapy arm. Additionally, in a small, second RCT that included 39 patients with PJI treated with 2-stage exchanges,[42] 4 vs. 6 weeks of antibiotics were similarly effective. Thus, the majority of authors felt that equipoise remains regarding 6 vs. 12 weeks of therapy for prosthetic exchange patients, particularly if *S. aureus* is not the etiologic pathogen, and for 1-stage exchanges or 2-stage exchanges with negative cultures prior to re-implantation; more data are needed to confirm duration in these settings.

© 2022 Spellberg B et al. *JAMA Network Open.*

The appropriate duration of therapy for other forms of retained implants is not clear from controlled studies. One reasonable strategy, without evidence for or against, may be to treat with antibiotics until the bone heals sufficiently enough that the implants can be removed.

Finally, chronic oral suppression may be generally tolerated and suitable for high-risk patients with retained infected implants who are poor repeat surgical candidates. While there are no prospective, controlled trials available to confirm that this practice is either safe or effective, for some patients, long-term oral suppressive therapy may offer an improved quality of life over long-term intravenous therapy, or the risk of relapse from no therapy.

Table 6. Summary of Antibiotic Durations for Osteomyelitis

	**Clear Recommendation**	**Clinical Review**
Osteomyelitis without retained implant (including DFO)	Maximum 6 weeks	• 3-4 weeks may be adequate with debridement; confirmatory studies desired
Osteomyelitis with total resection of infected bone		• No antibiotics is a reasonable option<br>• We do not recommend exceeding 5 days
PJI with DAIR		• All participating experts prefer 12 weeks<br>• A confirmatory, 2nd study is needed to enable a Clear Recommendation
PJI with Exchange		• 12 weeks favored by some experts<br>• Other experts believe equipoise remains for 6 vs. 12 weeks<br>➢ 6 weeks may be reasonable for non *S. aureus* pathogens, particularly for 1-stage exchanges<br>➢ 6 weeks may be reasonable for 2-stage exchange, although there is controversy about the need for further antibiotics after the second stage (reimplantation)

© 2022 Spellberg B et al. *JAMA Network Open.*

Overall Summary:
The duration of therapy for osteomyelitis has long been based on anecdote, case series, and tradition. However, multiple observational studies and seven RCTs have now begun to provide evidence to resolve this question. We reiterate that this guideline refers only to pyogenic osteomyelitis and does not consider durations of therapy for atypical causes (e.g., TB, fungal, *Brucella*).

Osteomyelitis (including DFO) without a Retained Implant

Observational Data of Spinal and Long Bone Osteomyelitis
Roblot et al. conducted a retrospective study of 120 patients with vertebral osteomyelitis to evaluate the impact of duration of therapy.[553] Receipt of ≤6 weeks of antibiotics was not associated with an increased risk of clinical failure compared to >6 weeks. Indeed, at a mean of 3.5 years of follow up, there was no significant difference in relapse or mortality for patients receiving ≤ 6 vs. >6 weeks of therapy.

Park et al. evaluated duration of antimicrobial therapy and outcomes among 345 patients with hematogenous vertebral osteomyelitis.[531] Source control was obtained in more than half of patients, either by surgery or by needle drainage. In the pre-planned multivariate analysis, end stage renal failure, infection with MRSA, and undrained abscess (paraspinal or psoas) were the only predictors of recurrence/treatment failure; receipt of < 6 weeks of antibiotic therapy was not associated with recurrence/failure. Having seen the results of their first multivariate analysis, the authors then constructed a post-hoc multivariate model to attempt to refine their results. In this post-hoc model, they found that receipt of <6 weeks of antibiotics was associated with increased risk of recurrence. However, the post-hoc model raises concerns about variable selection bias and multiple comparisons issues. Based on the pre-planned multivariate analysis, prolonging antibiotics for > 6 weeks was not associated with a decreased risk of clinical failure, while undrained abscesses and MRSA infection were. These factors might indicate the need to treat for > 6 weeks if source control cannot be achieved, and the desirability of finding alternative therapies for MRSA in lieu of vancomycin (see Section 5). In the context of osteomyelitis, there are no specific data to define inadequacy of source control; we suggest that the presence of undrained abscesses that are too large to be treated with antibiotics alone (e.g., more than 2-3 cm in diameter) is a reasonable definition.

Another study of 49 patients with chronic osteomyelitis who underwent debridement evaluated the impact of duration of therapy after surgical intervention.[554] The median number of debridements was 2 (range 1-10), and the median duration of antibiotic therapy post-debridement was 8 weeks (range 4 to 14 weeks). At a minimum of two years of follow up, 80% of patients had persistent treatment success. By multivariate analysis, neither administration of 1 vs. 2 vs. 3 weeks of intravenous antibiotics, nor administration of ≤6 or > 6 weeks of total antibiotics post-debridement, was associated with treatment success.

Other studies also suggest that courses < 6 weeks may result in similar efficacy to ≥ 6 weeks of antibiotic therapy. For example, Meißner et al. (first-author's last name indexed as Meissner on PubMed) treated 53 evaluable patients with chronic, post-traumatic (including motor vehicle accidents, gunshot wounds, war wounds) osteomyelitis of the long bones with oral fosfomycin (not the sachet formulation available in the US) for between 5 and 28 days.[500] These patients had very complex histories, with a mean of 37 months of persistence of osteomyelitis before treatment with fosfomycin was initiated. Etiologic pathogens were highly varied,

including *S. aureus,* coagulase-negative staphylococci, streptococci, enterococci, and a variety of Gram-negative bacilli, including *P. aeruginosa.* Yet, the ≤ 4 weeks of fosfomycin therapy achieved a 73% long term success rate at follow up. Similarly, Shcherbin et al. studied 33 patients who had an average of seven years of osteomyelitis following gunshot injuries.[555] In addition to debridement, patients were treated with lincomycin and gentamicin for ≤22 days (range 7-22), resulting in an 88% (23/26) long term success rate at a mean of four years of follow-up.

Finally, Haidar et al. systematically reviewed the literature for reports of patients treated with shorter durations of antibiotics for osteomyelitis.[378] In their summary of these small, uncontrolled case series, they describe a total of 21 other patients (non-redundant with the studies described above) treated with 1-4 weeks of a variety of antibiotics, of whom 18 (86%) achieved clinical success at last follow-up.

Observational Data of DFO

Several observational studies have evaluated the potential impact of therapy duration on DFO, with or without resection of infected bone. In a large observational study of 1,018 patients with DFI, 392 patients had confirmed osteomyelitis who underwent debridement or amputation, with antimicrobial therapy.[556] There was no difference in the mean duration of antimicrobial therapy for patients who developed recurrent osteomyelitis or not (mean 31 days vs. 34 days). Furthermore, there was no difference in the proportion of patients who received <3 weeks of total antimicrobial therapy vs. more among patients who developed recurrent osteomyelitis (27% vs. 32%). By multivariate analysis, total duration of therapy or receipt of <3 weeks of antibiotics vs. more were not associated with risk of recurrence of osteomyelitis.

In a study of 184 patients with DFO who underwent surgical resection, administration of <7 days of antibiotics in the presence of a positive post-operative margin by histopathology (indicating infection extended to the margin) was independently associated with a significant increase in risk of additional resection or amputation by multivariate analysis.[557] In a smaller, prospective observational study, 15 patients with diabetic foot osteomyelitis who underwent amputation or resection of infected bone were identified that had negative culture from the margin of the resection.[558] These patients were considered to have "clean margins", and were administered antibiotics for 8 +/-6 days post-operatively. Eighty percent (n = 12) achieved healing without osteomyelitis recurrence by six months of follow up.

Kowalski et al. evaluated outcomes in 111 patients who underwent bone resection treatment for DFO.[559] Of these patients, 39 had positive margins by histopathology, indicating residual osteomyelitis at the resection site. There was considerable overlap between the durations of antibiotics administered post-resection in patients with positive vs. negative margins, mean 19 days (range 10-134 days) vs. 14 days (range 2-63 days). There was no difference in long-term clinical failure between the two cohorts. However, more patients with positive margins required subsequent, more proximate amputation (44% vs. 15%). Of the patients with positive margins, the duration of antibiotics did not differ between those who progressed to failure and/or required further amputation vs. those who did not. Overall, these results suggest that the primary predictor of failure, including need for re-amputation, is surgical, rather than medical, management. This study does not support the practice of prolonging therapy post-amputation.

Finally, in the largest observational study of amputations specifically, Rossel et al. followed 482 patients with DFI who underwent amputation of various types.[560] Of these, 239

patients had a diagnosis of DFO. Amputation sites varied and included the metatarsals (n = 155), midfoot (n = 280), and hindfoot (n = 47). A median of 7 days (range 1-16 days) of antibiotics were administered post amputation. The investigators conducted a multivariate analysis and reported that neither duration of antibiotics, nor receipt of any antibiotics at all post amputation were associated with risk of clinical failure. They recommended that no antibiotics be administered after amputation with negative margins.

Cumulatively, these observational studies have not indicated an increased risk of clinical failure when ≤ 6 weeks of antibiotics were administered for various types of osteomyelitis, presuming source control is adequately achieved. Indeed, after resection of infected bone with clean margins (defined histopathologically or by negative cultures), it is not clear that any antibiotics are required. When administered in this setting, multiple observational studies have found no difference in outcomes in patients treated with a week or less of antibiotics. Even with debridement that does not remove all infected bone, observational data suggest 3 weeks may result in similar long-term success vs. longer courses. However, one study suggested that, when infected bone is retained, treatment courses of <1 week may result in higher failure rates.

<u>RCTs for Hematogenous or Contiguous Pyogenic Osteomyelitis without a Retained Implant (including DFO)</u>

Bernard et al. conducted an open-label RCT of vertebral osteomyelitis in which 351 patients were randomized to 6 vs. 12 weeks of antimicrobial therapy at 71 centers in France.[44] The specific antimicrobial agents used were left to investigator discretion, from a list of acceptable options. A wide variety of pathogens were etiologic in the infected patients, including *S. aureus,* coagulase negative staphylococci, streptococci, enterococci, Enterobacterales, and anaerobes. One year following treatment, the clinical success rates were 90.9% (160/176) vs. 90.8% (159/175) in the 6- vs. 12-week arms. All subgroup analyses were concordant.

Tone et al. conducted an open-label RCT of 6 vs. 12 weeks of antibiotic therapy for 40 patients with biopsy-proven DFO across 5 centers in France.[39] The patients were all managed without amputation. Again, a wide variety of antimicrobial regimens were used. *Staphylococci* and gram-negative bacteria were the predominant pathogens encountered. At a mean of 12 months of post-treatment follow up, treatment success rates were 60% (12/20) vs. 70% (14/20) in the 6- vs. 12-week groups. Fewer patients in the 6-week cohort experienced adverse events related to antimicrobial therapy compared with patients treated for 12 weeks.

Lazaro-Martinez et al. randomized 46 patients with DFO to 10 days of antibiotic therapy plus conservative surgical debridement (defined as removal of infected bone without amputation) vs. 90 days of antibiotics without debridement.[38] Treatment success, defined as persistent ulcer healing, occurred in 86% (19/22) of patients treated with 10 days of antibiotics plus debridement vs. 75% (18/24) of patients treated with 90 days of antibiotics alone.

Similarly, Gariani et al. randomized 93 patients with DFO to treatment with 3 vs. 6 weeks of antibiotics.[40] All patients underwent debridement to remove necrotic tissue, but in contrast to the study by Lazaro-Martinez et al.,[38] there was no intent to remove all infected bone. Indeed, in the Methods the authors specifically comment that removal of all infected bone was an exclusion. After a median of 11 months of follow up, treatment success rates were 84% (37/44) vs. 73% (36/49), while antibiotic-related adverse events were reported in 9% (4/44) vs. 14% (7/49) for the 3- vs. 6-week therapy arms, respectively.

Finally, Benkabouche et al. randomized 123 patients with osteomyelitis and various orthopaedic implants to 4 vs. 6 weeks of antibiotic therapy.[42] All infected implants were

surgically removed.[42] Prosthetic materials included 44 orthopaedic plates, 11 orthopaedic nails, 39 prosthetic joints, and 30 miscellaneous orthopaedic hardware. A wide variety of antibiotics were used. The overall treatment success rate at a median of 2.2 years of follow up was 94% (58/62) vs. 95% (58/61) in the 4- vs. 6-week therapy arms. Removing the 39 patients who had PJI, the success rates in the 4- vs. 6-week therapy arms were 94% (44/47) and 100% (37/37), respectively.

Thus, the available RCT data are concordant with observational data in demonstrating that six weeks of therapy is adequate for osteomyelitis, irrespective of hematogenous or contiguous routes of infection and presuming adequate source control. Furthermore, specifically in the setting of DFO, two small RCTs indicated that shorter regimens may be effective, ranging from 10 days after complete removal of infected bone to three weeks with debridement of necrotic tissue. Additionally, one RCT suggested that with removal of implanted materials with debridement, four weeks of antibiotics may be adequate to treat osteomyelitis. These data are concordant with observational studies suggesting that four weeks may be adequate for routine cases presuming source control is achieved, and it is conceivable that no therapy—and certainly no more than 2-10 days of therapy—is needed after total resection of infection with clear margins.

Therefore, we make a Clear Recommendation that no more than six weeks of antibiotic therapy should be administered for hematogenous or contiguous pyogenic osteomyelitis, presuming adequate source control. Some clinicians may prefer to use a 3- or 4-week regimen for appropriate cases with adequate debridement of bone, with small RCTs in support. However, sufficient data are lacking to definitively establish a Clear Recommendation between 4 vs. 6 weeks of therapy. We are also unable to make a Clear Recommendation regarding the duration of therapy after complete resection of infected bone with clear margins. Available data suggest that it may be reasonable to administer no antibiotics in this setting if the treating clinicians are confident all infected bone was removed, and if antibiotics are administered, durations beyond 10 days have not been shown to be of benefit, and we do not recommend exceeding 5 days of therapy based on consensus.

Pyogenic Osteomyelitis with a Retained Implant (including PJI)

<u>Observational Data</u>

Observational data on durations of IV antibiotic therapy, total duration of therapy, or use of chronic suppression after completion of initial therapy, have demonstrated mixed results in patients with PJI, resulting in equipoise without a clear evidence of consistent benefit of longer therapy.[223,224,420,439,538,561-569]

Among several larger studies, Chaussade et al. retrospectively reported the outcomes of 87 patients with PJI who underwent DAIR and were treated with 6 or 12 weeks of total antimicrobial therapy.[565] They reported no difference in long-term treatment success between these two groups at a median of more than 3 years of follow up. Similarly, Tornero et al. evaluated 143 patients who underwent DAIR.[439] They, too, reported no relationship between duration of therapy (median 77 days) and treatment failure. Specifically, patients who experienced treatment success vs. failure had similar durations of antibiotic therapy, and a similar proportion of patients who were treated for >75 or >100 days with antibiotics. Furthermore, duration of antibiotic therapy was not associated with treatment failure by

multivariable analysis. Rodriguez et al. described 50 patients with hematogenous PJI.[566] Neither antibiotic treatment for >8 weeks or >12 weeks was associated with differences in treatment outcomes by multivariable analysis, either in the overall cohort (including the 16 patients treated with a two-stage joint replacement), or the DAIR cohort of 34 patients.

Finally, Byren et al. evaluated 112 patients with PJI who underwent DAIR.[567] During a mean follow up of 2.3 years, 20 (18%) patients experienced treatment failure. There was no relationship identified between the duration of the initial course of antibiotic therapy and the risk of treatment failure. Treatment failures were then placed on chronic oral suppressive antibiotic therapy (mean of 1.5 years of therapy during follow up). After chronic suppressive therapy stopped, there was an increase in the risk of relapsed infection over the ensuing several months. Nonetheless, the total proportion of failures was relatively low (<15%), indicating that most patients did not fail after stopping chronic suppression.

However, other studies have suggested an advantage of longer therapy courses. One case series of 60 patients with PJI found that post-debridement antibiotic durations of < 3 months were associated with an increase in treatment failures by multivariable analysis.[570] Other observational studies have reported that the clinical success of chronic oral suppression after a 4 to 6 week course of "induction" antibiotic therapy can result in higher rates of treatment success at long-term follow up.[226,571,572]

Shah et al. conducted multivariable analysis for treatment success among 108 patients with PJI treated with DAIR, of whom 47% received chronic oral suppression after an initial IV course of antibiotics.[573] Use of chronic suppression was associated with a significantly higher rate of long-term treatment success than not (HR 2.5; $p < 0.009$). However, there was no difference in treatment success rates for patients treated with <1 year or > 1 year of oral suppression. There was also no difference in the rate of adverse events associated with antibiotic use between those who were on antibiotics for ≤ 6 weeks vs. > 6 weeks. In another study, 89 patients with infected orthopaedic implants, including spinal hardware, internal fixation devices, and PJIs, were studied to determine if chronic oral antibiotic suppression altered risk of long-term treatment failure.[574] By multivariate analysis, receipt of 3 months of chronic oral suppressive antibiotics was associated with a reduced risk of relapse, however extending chronic suppression to 6 months was not.

Finally, a larger study from 2020 described the outcomes of 302 patients with PJI treated with chronic antibiotic suppressive therapy.[569] The mean patient age was 75 years, and more than a quarter of the patients were aged >85 years. Suppressive therapy was administered for a median of 3 years, with an interquartile range of 1.7 to 5 years. Tetracyclines and TMP-SMX were the most commonly administered agents, followed by β lactams and fluoroquinolones. The overall success rate was 59%; the 2-year follow-up success rate was 75% and declined to 50% at 5-year follow-up. By multivariable analysis the primary predictors of failure were age >70 years and infection caused by gram-positive cocci compared to other pathogens. Twenty-seven percent of patients suffered from adverse events, primarily gastrointestinal and cutaneous; 1% of patients developed *C. difficile* colitis. Adverse events were severe enough to necessitate cessation of suppression in 6% of patients and change to an alternative antibiotic in 15%.

A systematic review of the literature in 2020 found that the evidence in favor of chronic suppression with antibiotics after PJI treated with DAIR was limited and of low quality; therefore, it was insufficient to draw meaningful conclusions regarding safety and efficacy.[575] They described a 15% rate of adverse events (the nature of which were not well delineated in the review), and a 75% long-term treatment success rate, which is not substantively different than

other studies (such as those described above) that did not use chronic suppression. A second systematic review in 2021 reported an 8%-43% adverse event rate for chronic suppression depending on the study, and suggested that chronic suppression could be considered for patients whose implants cannot be removed, including unfavorable surgical risk:benefit ratio, short life-expectancy, or patient refusal.[576]

For implants that are intended to be removed following bone healing, it may be possible to treat until the bone is stable enough for removal, and then complete a 4-6 week course of therapy after the implant is removed. Furthermore, providing the bone heals to stability, it may be reasonable to stop therapy even without removal of the implant. In the event of a relapse, the surgeon may then remove the implant without the need for complex reconstructions, after which a 6-week course of therapy could be applied. We did not find data evaluating the efficacy or tolerability of such a practice. Nevertheless, it may be an option to spare the potential for chronic oral suppression for patients who can safely have implants removed. Indeed, wherever possible, it is likely preferable to remove the implant to allow a higher chance of long-term remission as opposed to retaining the implant followed by prolonged chronic oral suppression or 'test of cure' with the implant still in place.

Ultimately, delaying relapse for patients who are too high-risk to tolerate repeat surgery may be an important goal. This goal must be weighed against the potential harm of the antibiotics and any suppressive treatment should be reviewed at regular intervals, since new treatment toxicities may tip the harm:benefit balance. As Byren et al. wrote, “One might conclude that most patients cured of PJI by DAIR are cured early on, and that prolonged antibiotic therapy does not prevent treatment failures in those who are not cured, but merely postpones them...Life-long antibiotics might simply postpone, rather than prevent, treatment failure, but this may be all that is required for older patients with limited life expectancy. For patients in whom further surgery might be limb- or life-threatening, postponing this outcome with indefinite antibiotic treatment is also justified”.[567]

<u>RCTs</u>

In an open label study, Lora-Tamayo et al. randomized 63 patients with early onset PJI managed surgically with DAIR to eight weeks vs. three months of treatment with levofloxacin and rifampin.[41] Early onset infection was defined as occurring within 30 days of implantation. They found very high success rates in both arms of the study in the per protocol analysis, with 92% (22/24) and 95% (19/20) success rates at a median of 862 days of follow up in the short vs. longer therapy arms. However, there were seven more dropouts from the intention-to-treat (ITT) population in the longer therapy arm, due to higher rates of adverse events (13% vs. 18%), orthopaedic failure of the implant (0% vs. 6%), and lost to follow up (3% vs. 12%). As a result, in the ITT population, the short course therapy regimen had a more favorable success rate than longer therapy at 73% (22/30) vs. 58% (19/33). Indeed, a meta-analysis of this RCT with nine other retrospective studies concluded that shorter course therapy regimens had similar outcomes to longer, and that eight weeks of therapy was adequate for hip PJIs and 75 days (just under 7 weeks) was adequate for knee PJIs treated with DAIR.[577]

As discussed above, an RCT of 4 vs. 6 weeks of antibiotics for patients with infected implants included 39 patients with PJI.[42] These PJI patients all underwent two-stage replacements, and hence removal of the initially infected implant. On inquiry with the corresponding author, the treatment success rates in the 4 vs. 6 weeks arms for these 39 patients were 93% (14/15) vs. 88% (21/24), respectively, at two years of follow up.

However, most recently Bernard et al. conducted a larger, open-label, multi-centered study (DATIPO) of 410 patients with PJI randomized to receive 6 vs. 12 weeks of antibiotic therapy.[43] A wide variety of antibiotic therapy was used, with 70% of patients receiving rifampin therapy, 68% a fluoroquinolone, and 51% both. The median duration of IV therapy administered was only nine days, with the remainder of therapy in both arms administered orally. Approximately 41% of patients were treated surgically with DAIR, 37% with one stage prosthetic implant exchange, and 22% with two stage exchange. The primary endpoint of treatment success in the modified ITT population was significantly lower in the short course therapy arm, at 82% (158/193) vs. 91% (173/191), for an adjusted difference of 9% (2%-16%). Treatment success in the per protocol population was also significantly lower for the short-course therapy arm at 82% (136/165) vs. 93% (149/160), for an adjusted difference of 11% (4%-18%). Given the very high treatment success rate (91% in the ITT population, 93% in the per protocol) with a 12-week antibiotic course, there would likely be diminishing returns to prolonging therapy beyond 12 weeks.

However, the treatment difference was most dramatic in the DAIR cohort, with a success rate 16% (3-30%) higher in the 12-week arm. Indeed, 23 of 32 of the treatment failures in the 6-week arm occurred in the DAIR cohort (with only six in the 2-stage and three in the 1-stage exchange cohorts). The differences in efficacy between long vs. short therapy for the one and two stage exchanges were not statistically significant, at 1.2% better (95% CI, 5% worse to 8% better) and 10% better (95% CI, 3% worse to 23% better) for longer therapy, respectively. Furthermore, among the patients treated with six weeks who underwent knee prosthetic exchanges, there were no failures, whereas the infection rates were higher for hip prosthesis exchanges. For patients undergoing 2-stage exchange, in addition to 6 vs. 12 weeks of antibiotics, the time between exchanges also varied at 6 vs. 12 weeks. Thus, the morbidity associated with a further six weeks prior to definitive prosthesis replacement should be considered. Thus, there may remain a role for six weeks of therapy in some patients undergoing prosthetic exchanges.

Another note of caution about the DATIPO RCT is that there were important imbalances in pathogens causing infections in the 6- vs. 12-week therapy arms. Specifically, the 6-week arm had 20 more infections caused by *S. aureus* which has, in general, higher rates of treatment failure. The clinical failure rate for *S. aureus* infection was 23% (21/90) vs. 13% (9/70) in the 6- vs. 12-week arms, suggesting that 12 weeks of therapy may improve outcomes of *S. aureus* infection, specifically. The 12 more clinical failures due to persistent *S. aureus* infections in the shorter therapy arm accounted for 71% (12/17) of all excess failures in the 6-week arm, across all surgical subtypes (DAIR, 1-, or 2-stage exchanges).

Conclusions

Overall, based on clear superiority in the largest RCT conducted, all participating experts unanimously prefer 12 weeks of therapy for PJI treated with DAIR; a Clear Recommendation is not made because WikiGuidelines™ evidentiary standards require two prospective, controlled studies, and a second study is not yet available to address this question.

Some experts also prefer 12 weeks of therapy for PJI treated with 1- or 2-stage exchanges given the trends to favorable outcomes in the DATIPO RCT, particularly for patients with hip prosthesis (as opposed to knee) or with *S. aureus*. However, because microbiological imbalances in the larger RCT could have disadvantaged the 6-week therapy arm, failures were not seen in patients undergoing knee exchanges, and very high cure rates were achieved with either 4 or 6

weeks of antibiotics in patients undergoing 2-stage exchanges in the smaller RCT,[42] some equipoise remains regarding antibiotic durations for PJI caused by pathogens other than *S. aureus*, particularly for patients managed surgically with 1-stage exchanges or 2-stage exchanges with negative cultures prior to reimplantation.

Of note, for patients undergoing 2-stage exchanges, duration may also be affected by the nature of the care intervening the exchanges. Surgical management without a spacer after explantation may rationally support a 4-6 week duration of therapy.[42] However, this procedure was not used in the DATIPO RCT, in which patients undergoing 2 stage revisions may have had a spacer between procedures. In DATIPO, patients undergoing such exchanges of the hip had an imbalance in failures favoring the 12-week regimen.

More RCT data are needed to definitively establish the optimal treatment duration of patients with PJI undergoing prosthetic exchange.

© 2022 Spellberg B et al. *JAMA Network Open.*

Discussion

Limitations

The establishment of only two Clear Recommendations highlights the need for additional high-quality studies of osteomyelitis. In particular, studies are needed regarding new approaches to diagnostics; to determine the potential benefit or harm of adding anti-anaerobic antimicrobial therapy for DFO; to elucidate the comparative effectiveness of various antimicrobial options; to determine if adjunctive rifampin therapy has benefit; to identify which patients are more likely to relapse after completion of therapy; to further clarify antibiotic durations of therapy; to define the role and optimal methodologies of surgical management; and to define the role if any of non-antimicrobial adjunctive strategies (e.g., hyperbaric oxygen therapy). We would also like to incorporate authors from LMIC countries in future revisions to ensure the WikiGuidelines™ are broadly applicable to these settings.

Conclusions

WikiGuidelines™ represent a novel approach to guideline construction, clearly delineating evidenced-based recommendations from opinions based on lower quality data. Resulting changes in management of pyogenic osteomyelitis include recognizing the low value and high burden that plain X-rays incur if routinely ordered for all patients, reducing the routine ordering of low value, low accuracy blood biomarkers, increasing adoption of oral therapy, and limiting the duration of therapy to the shortest necessary for optimizing cure.

These guidelines are based on published data available as of March 1, 2022. Clinicians who believe other evidence should be considered may contact any of the authors to initiate possible revisions to the guidelines, which the authors intend to complete in close to real time.

WikiGuidelines™ participants understand that no clinical trial can extrapolate to all possible patient care scenarios. Thus, we expect that these guidelines should not establish medicolegal standards of care or replace clinician judgment for individual patients.

© 2022 Spellberg B et al. *JAMA Network Open.*

References

1. Packer M. The Room Where It Happens: A Skeptic's Analysis of the New Heart Failure Guidelines. *J Card Fail*. Sep 2016;22(9):726-30. doi:10.1016/j.cardfail.2016.07.433
2. Kavanagh BP. The GRADE system for rating clinical guidelines. *PLoS medicine*. Sep 2009;6(9):e1000094. doi:10.1371/journal.pmed.1000094
3. Shaneyfelt TM, Centor RM. Reassessment of clinical practice guidelines: go gently into that good night. *JAMA : the journal of the American Medical Association*. Feb 25 2009;301(8):868-9. doi:10.1001/jama.2009.225
4. Miles KE, Rodriguez R, Gross AE, Kalil AC. Strength of Recommendation and Quality of Evidence for Recommendations in Current Infectious Diseases Society of America Guidelines. *Open forum infectious diseases*. Feb 2021;8(2):ofab033. doi:10.1093/ofid/ofab033
5. Wright WF, Jorgensen SCJ, Spellberg B. Heaping the Pelion of Vancomycin on the Ossa of MRSA: Back to Basics in Clinical Care and Guidelines. *Clin Infect Dis*. Sep 9 2020;doi:10.1093/cid/ciaa1360
6. Brass EP, Hiatt WR. Aspirin Monotherapy Should Not Be Recommended for Cardioprotection in Patients With Symptomatic Peripheral Artery Disease. *Circulation*. Aug 29 2017;136(9):785-786. doi:10.1161/CIRCULATIONAHA.117.028888
7. Spellberg B, Wright WF, Shaneyfelt TM, Centor RM. The Future of Medical Guidelines—Standardizing Clinical Care with the Humility of Uncertainty. *Annals of internal medicine*. 2021;174:1740-42.
8. Kremers HM, Nwojo ME, Ransom JE, Wood-Wentz CM, Melton LJ, 3rd, Huddleston PM, 3rd. Trends in the epidemiology of osteomyelitis: a population-based study, 1969 to 2009. *J Bone Joint Surg Am*. May 20 2015;97(10):837-45. doi:10.2106/JBJS.N.01350
9. Lindbloom BJ, James ER, McGarvey WC. Osteomyelitis of the foot and ankle: diagnosis, epidemiology, and treatment. *Foot Ankle Clin*. Sep 2014;19(3):569-88. doi:10.1016/j.fcl.2014.06.012
10. Zardi EM, Franceschi F. Prosthetic joint infection. A relevant public health issue. *J Infect Public Health*. Dec 2020;13(12):1888-1891. doi:10.1016/j.jiph.2020.09.006
11. Stanley CM, Rutherford GW, Morshed S, Coughlin RR, Beyeza T. Estimating the healthcare burden of osteomyelitis in Uganda. *Trans R Soc Trop Med Hyg*. Feb 2010;104(2):139-42. doi:10.1016/j.trstmh.2009.05.014
12. Geraghty T, LaPorta G. Current health and economic burden of chronic diabetic osteomyelitis. *Expert Rev Pharmacoecon Outcomes Res*. Jun 2019;19(3):279-286. doi:10.1080/14737167.2019.1567337
13. Ferguson J, McNally M, Stubbs D. THE FINANCIAL BURDEN OF TREATING OSTEOMYELITIS IN THE UK. *Orthopaedic Proceedings*. 2019;101-B(S14):ePub.
14. Toscano CM, Sugita TH, Rosa MQM, Pedrosa HC, Rosa RDS, Bahia LR. Annual Direct Medical Costs of Diabetic Foot Disease in Brazil: A Cost of Illness Study. *Int J Environ Res Public Health*. Jan 8 2018;15(1)doi:10.3390/ijerph15010089
15. Moodie RL. Osteomyelitis in the Permian. *Science*. Apr 8 1921;53(1371):333. doi:53/1371/333 [pii]

10.1126/science.53.1371.333

16. Spellberg B, Lipsky BA. Systemic antibiotic therapy for chronic osteomyelitis in adults. *Clin Infect Dis*. Feb 2012;54(3):393-407. doi:cir842 [pii]

© 2022 Spellberg B et al. *JAMA Network Open.*

10.1093/cid/cir842
17. Li HK, Rombach I, Zambellas R, et al. Oral versus Intravenous Antibiotics for Bone and Joint Infection. *N Engl J Med*. Jan 31 2019;380(5):425-436. doi:10.1056/NEJMoa1710926
18. Wald-Dickler N, Holtom P, Phillips MC, et al. Oral Is the New IV--Challenging Decades of Blood and Bone Infection Dogma: A Systematic Review. *Am J Med*. 2021;ePublished
19. High Value Care: ACP's High Value Care initiative aims to improve health, avoid harms, and eliminate wasteful practices. American College of Physicians. Accessed July 18, 2021. https://www.acponline.org/clinical-information/high-value-care
20. Wong D, Holtom P, Spellberg B. Osteomyelitis Complicating Sacral Pressure Ulcers: Whether or Not to Treat With Antibiotic Therapy. *Clin Infect Dis*. Jan 7 2019;68(2):338-342. doi:10.1093/cid/ciy559
21. Crespo A, Stevens NM, Chiu E, Pham V, Leucht P. Incidence of Osteomyelitis in Sacral Decubitus Ulcers and Recommendations for Management. *JBJS Rev*. Jun 2020;8(6):e0187. doi:10.2106/JBJS.RVW.19.00187
22. Peloquin CA, Jaresko GS, Yong CL, Keung AC, Bulpitt AE, Jelliffe RW. Population pharmacokinetic modeling of isoniazid, rifampin, and pyrazinamide. *Antimicrob Agents Chemother*. Dec 1997;41(12):2670-9.
23. Acocella G. Pharmacokinetics and metabolism of rifampin in humans. *Reviews of infectious diseases*. Jul-Aug 1983;5 Suppl 3:S428-32.
24. Jayaram R, Gaonkar S, Kaur P, et al. Pharmacokinetics-pharmacodynamics of rifampin in an aerosol infection model of tuberculosis. *Antimicrob Agents Chemother*. Jul 2003;47(7):2118-24. doi:10.1128/AAC.47.7.2118-2124.2003
25. Hirai J, Hagihara M, Kato H, et al. Investigation on rifampicin administration from the standpoint of pharmacokinetics/pharmacodynamics in a neutropenic murine thigh infection model. *J Infect Chemother*. Jun 2016;22(6):387-94. doi:10.1016/j.jiac.2016.02.011
26. Marsot A, Menard A, Dupouey J, Allanioux L, Blin O, Guilhaumou R. Evaluation of current dosing guidance for oral rifampicin treatment in adult patients with osteoarticular infections. *Br J Clin Pharmacol*. Nov 2020;86(11):2319-2324. doi:10.1111/bcp.14319
27. Rappo U, Puttagunta S, Shevchenko V, et al. Dalbavancin for the Treatment of Osteomyelitis in Adult Patients: A Randomized Clinical Trial of Efficacy and Safety. *Open forum infectious diseases*. Jan 2019;6(1):ofy331. doi:10.1093/ofid/ofy331
28. Almangour TA, Alhifany AA. Dalbavancin for the management of osteomyelitis: a major step forward? *J Antimicrob Chemother*. Oct 1 2020;75(10):2717-2722. doi:10.1093/jac/dkaa188
29. Greenberg RN, Tice AD, Marsh PK, et al. Randomized trial of ciprofloxacin compared with other antimicrobial therapy in the treatment of osteomyelitis. *Am J Med*. Apr 27 1987;82(4A):266-9.
30. Gentry LO, Rodriguez GG. Oral ciprofloxacin compared with parenteral antibiotics in the treatment of osteomyelitis. *Antimicrob Agents Chemother*. Jan 1990;34(1):40-3.
31. Mader JT, Cantrell JS, Calhoun J. Oral ciprofloxacin compared with standard parenteral antibiotic therapy for chronic osteomyelitis in adults. *J Bone Joint Surg Am*. Jan 1990;72(1):104-10.
32. Gentry LO, Rodriguez-Gomez G. Ofloxacin versus parenteral therapy for chronic osteomyelitis. *Antimicrob Agents Chemother*. Mar 1991;35(3):538-41.

© 2022 Spellberg B et al. *JAMA Network Open.*

33. Gomis M, Barberan J, Sanchez B, Khorrami S, Borja J, Garcia-Barbal J. Oral ofloxacin versus parenteral imipenem-cilastatin in the treatment of osteomyelitis. *Rev Esp Quimioter*. Sep 1999;12(3):244-9.
34. Schrenzel J, Harbarth S, Schockmel G, et al. A randomized clinical trial to compare fleroxacin-rifampicin with flucloxacillin or vancomycin for the treatment of staphylococcal infection. *Clin Infect Dis*. Nov 1 2004;39(9):1285-92. doi:10.1086/424506
35. Euba G, Murillo O, Fernandez-Sabe N, et al. Long-term follow-up trial of oral rifampin-cotrimoxazole combination versus intravenous cloxacillin in treatment of chronic staphylococcal osteomyelitis. *Antimicrob Agents Chemother*. Jun 2009;53(6):2672-6. doi:AAC.01504-08 [pii]

10.1128/AAC.01504-08
36. Lipsky BA, Baker PD, Landon GC, Fernau R. Antibiotic therapy for diabetic foot infections: comparison of two parenteral-to-oral regimens. *Clin Infect Dis*. Apr 1997;24(4):643-8.
37. Lipsky BA, Itani K, Norden C. Treating foot infections in diabetic patients: a randomized, multicenter, open-label trial of linezolid versus ampicillin-sulbactam/amoxicillin-clavulanate. *Clin Infect Dis*. Jan 1 2004;38(1):17-24.
38. Lazaro-Martinez JL, Aragon-Sanchez J, Garcia-Morales E. Antibiotics versus conservative surgery for treating diabetic foot osteomyelitis: a randomized comparative trial. *Diabetes care*. 2014;37(3):789-95. doi:10.2337/dc13-1526
39. Tone A, Nguyen S, Devemy F, et al. Six-week versus twelve-week antibiotic therapy for nonsurgically treated diabetic foot osteomyelitis: a multicenter open-label controlled randomized study. *Diabetes care*. Feb 2015;38(2):302-7. doi:10.2337/dc14-1514
40. Gariani K, Pham TT, Kressmann B, et al. Three versus six weeks of antibiotic therapy for diabetic foot osteomyelitis: A prospective, randomized, non-inferiority pilot trial. *Clin Infect Dis*. Nov 26 2020;doi:10.1093/cid/ciaa1758
41. Lora-Tamayo J, Euba G, Cobo J, et al. Short- versus long-duration levofloxacin plus rifampicin for acute staphylococcal prosthetic joint infection managed with implant retention: a randomised clinical trial. *Int J Antimicrob Agents*. Sep 2016;48(3):310-6. doi:10.1016/j.ijantimicag.2016.05.021
42. Benkabouche M, Racloz G, Spechbach H, Lipsky BA, Gaspoz JM, Uckay I. Four versus six weeks of antibiotic therapy for osteoarticular infections after implant removal: a randomized trial. *J Antimicrob Chemother*. Aug 1 2019;74(8):2394-2399. doi:10.1093/jac/dkz202
43. Bernard L, Arvieux C, Brunschweiler B, et al. Antibiotic Therapy for 6 or 12 Weeks for Prosthetic Joint Infection. *N Engl J Med*. May 27 2021;384(21):1991-2001. doi:10.1056/NEJMoa2020198
44. Bernard L, Dinh A, Ghout I, et al. Antibiotic treatment for 6 weeks versus 12 weeks in patients with pyogenic vertebral osteomyelitis: an open-label, non-inferiority, randomised, controlled trial. *Lancet*. Nov 5 2015;385:875-82. doi:10.1016/S0140-6736(14)61233-2
45. Llewellyn A, Jones-Diette J, Kraft J, Holton C, Harden M, Simmonds M. Imaging tests for the detection of osteomyelitis: a systematic review. *Health Technol Assess*. Oct 2019;23(61):1-128. doi:10.3310/hta23610
46. Ryan EC, Ahn J, Wukich DK, Kim PJ, La Fontaine J, Lavery LA. Diagnostic Utility of Erythrocyte Sedimentation Rate and C-Reactive Protein in Osteomyelitis of the Foot in Persons Without Diabetes. *J Foot Ankle Surg*. May 2019;58(3):484-488. doi:10.1053/j.jfas.2018.09.025

© 2022 Spellberg B et al. *JAMA Network Open.*

47. Ghassibi M, Yen TC, Harris S, Si Z, Leary E, Choma TJ. Responsiveness of routine diagnostic tests for vertebral osteomyelitis may be influenced by the infecting organism. *Spine J*. Apr 20 2021;doi:10.1016/j.spinee.2021.04.001
48. Wu Y, Lu X, Hong J, et al. Detection of extremity chronic traumatic osteomyelitis by machine learning based on computed-tomography images: A retrospective study. *Medicine (Baltimore)*. Feb 2020;99(9):e19239. doi:10.1097/MD.0000000000019239
49. Pupaibool J, Vasoo S, Erwin PJ, Murad MH, Berbari EF. The utility of image-guided percutaneous needle aspiration biopsy for the diagnosis of spontaneous vertebral osteomyelitis: a systematic review and meta-analysis. *Spine J*. Jan 1 2015;15(1):122-31. doi:10.1016/j.spinee.2014.07.003
50. Llewellyn A, Kraft J, Holton C, Harden M, Simmonds M. Imaging for detection of osteomyelitis in people with diabetic foot ulcers: A systematic review and meta-analysis. *Eur J Radiol*. Oct 2020;131:109215. doi:10.1016/j.ejrad.2020.109215
51. Lauri C, Tamminga M, Glaudemans A, et al. Detection of Osteomyelitis in the Diabetic Foot by Imaging Techniques: A Systematic Review and Meta-analysis Comparing MRI, White Blood Cell Scintigraphy, and FDG-PET. *Diabetes care*. Aug 2017;40(8):1111-1120. doi:10.2337/dc17-0532
52. Xu J, Cheng F, Li Y, Zhang J, Feng S, Wang P. Erythrocyte Sedimentation Rate Combined With the Probe-to-Bone Test for Fast and Early Diagnosis of Diabetic Foot Osteomyelitis. *Int J Low Extrem Wounds*. Jun 15 2020:1534734620923278. doi:10.1177/1534734620923278
53. Moallemi SK, Niroomand M, Tadayon N, Forouzanfar MM, Fatemi A. Diagnostic Value of Erythrocyte Sedimentation Rate and C Reactive Protein in detecting Diabetic Foot Osteomyelitis; a Cross-sectional Study. *Arch Acad Emerg Med*. 2020;8(1):e71.
54. Lavery LA, Ahn J, Ryan EC, et al. What are the Optimal Cutoff Values for ESR and CRP to Diagnose Osteomyelitis in Patients with Diabetes-related Foot Infections? *Clin Orthop Relat Res*. Jul 2019;477(7):1594-1602. doi:10.1097/CORR.0000000000000718
55. Victoria van Asten SA, Geradus Peters EJ, Xi Y, Lavery LA. The Role of Biomarkers to Diagnose Diabetic Foot Osteomyelitis. A Meta-analysis. *Curr Diabetes Rev*. 2016;12(4):396-402. doi:10.2174/1573399811666150713104401
56. Markanday A. Acute Phase Reactants in Infections: Evidence-Based Review and a Guide for Clinicians. *Open forum infectious diseases*. Sep 2015;2(3):ofv098. doi:10.1093/ofid/ofv098
57. Lam K, van Asten SA, Nguyen T, La Fontaine J, Lavery LA. Diagnostic Accuracy of Probe to Bone to Detect Osteomyelitis in the Diabetic Foot: A Systematic Review. *Clin Infect Dis*. Oct 1 2016;63(7):944-8. doi:10.1093/cid/ciw445
58. Sconfienza LM, Signore A, Cassar-Pullicino V, et al. Diagnosis of peripheral bone and prosthetic joint infections: overview on the consensus documents by the EANM, EBJIS, and ESR (with ESCMID endorsement). *Eur Radiol*. Dec 2019;29(12):6425-6438. doi:10.1007/s00330-019-06326-1
59. Galley J, Sutter R, Stern C, Filli L, Rahm S, Pfirrmann CWA. Diagnosis of Periprosthetic Hip Joint Infection Using MRI with Metal Artifact Reduction at 1.5 T. *Radiology*. Jul 2020;296(1):98-108. doi:10.1148/radiol.2020191901
60. Schwaiger BJ, Gassert FT, Suren C, et al. Diagnostic accuracy of MRI with metal artifact reduction for the detection of periprosthetic joint infection and aseptic loosening of total hip arthroplasty. *Eur J Radiol*. Oct 2020;131:109253. doi:10.1016/j.ejrad.2020.109253

© 2022 Spellberg B et al. *JAMA Network Open.*

61. Ikeuchi M, Okanoue Y, Izumi M, et al. Diagnostic value of triple-phase bone scintigraphy for the diagnosis of infection around antibiotic-impregnated cement spacers. *Springerplus*. 2013;2:401. doi:10.1186/2193-1801-2-401
62. Nagoya S, Kaya M, Sasaki M, Tateda K, Yamashita T. Diagnosis of peri-prosthetic infection at the hip using triple-phase bone scintigraphy. *J Bone Joint Surg Br*. Feb 2008;90(2):140-4. doi:10.1302/0301-620X.90B2.19436
63. Ouyang Z, Li H, Liu X, Zhai Z, Li X. Prosthesis infection: diagnosis after total joint arthroplasty with three-phase bone scintigraphy. *Ann Nucl Med*. Dec 2014;28(10):994-1003. doi:10.1007/s12149-014-0899-5
64. Erba PA, Glaudemans AW, Veltman NC, et al. Image acquisition and interpretation criteria for 99mTc-HMPAO-labelled white blood cell scintigraphy: results of a multicentre study. *Eur J Nucl Med Mol Imaging*. Apr 2014;41(4):615-23. doi:10.1007/s00259-013-2631-4
65. Teiler J, Ahl M, Akerlund B, et al. Is 99mTc-HMPAO-leukocyte imaging an accurate method in evaluating therapy result in prosthetic joint infection and diagnosing suspected chronic prosthetic joint infection? *Q J Nucl Med Mol Imaging*. Mar 2020;64(1):85-95. doi:10.23736/S1824-4785.19.03040-1
66. Kiran M, Donnelly TD, Armstrong C, Kapoor B, Kumar G, Peter V. Diagnostic utility of fluorodeoxyglucose positron emission tomography in prosthetic joint infection based on MSIS criteria. *Bone Joint J*. Aug 2019;101-B(8):910-914. doi:10.1302/0301-620X.101B8.BJJ-2018-0929.R2
67. Kwee TC, Kwee RM, Alavi A. FDG-PET for diagnosing prosthetic joint infection: systematic review and metaanalysis. *Eur J Nucl Med Mol Imaging*. Nov 2008;35(11):2122-32. doi:10.1007/s00259-008-0887-x
68. Jin H, Yuan L, Li C, Kan Y, Hao R, Yang J. Diagnostic performance of FDG PET or PET/CT in prosthetic infection after arthroplasty: a meta-analysis. *Q J Nucl Med Mol Imaging*. Mar 2014;58(1):85-93.
69. Berbari E, Mabry T, Tsaras G, et al. Inflammatory blood laboratory levels as markers of prosthetic joint infection: a systematic review and meta-analysis. *J Bone Joint Surg Am*. Sep 1 2010;92(11):2102-9. doi:10.2106/JBJS.I.01199
70. Perez-Prieto D, Portillo ME, Puig-Verdie L, et al. C-reactive protein may misdiagnose prosthetic joint infections, particularly chronic and low-grade infections. *Int Orthop*. Jul 2017;41(7):1315-1319. doi:10.1007/s00264-017-3430-5
71. Qu X, Zhai Z, Liu X, et al. Evaluation of white cell count and differential in synovial fluid for diagnosing infections after total hip or knee arthroplasty. *PLoS One*. 2014;9(1):e84751. doi:10.1371/journal.pone.0084751
72. Lee YS, Koo KH, Kim HJ, et al. Synovial Fluid Biomarkers for the Diagnosis of Periprosthetic Joint Infection: A Systematic Review and Meta-Analysis. *J Bone Joint Surg Am*. Dec 20 2017;99(24):2077-2084. doi:10.2106/JBJS.17.00123
73. Zhou AK, Girish M, Thahir A, Lim JA, Chen X, Krkovic M. Radiological evaluation of postoperative osteomyelitis in long bones: Which is the best tool? *J Perioper Pract*. Mar 9 2021:1750458920961347. doi:10.1177/1750458920961347
74. Lee YJ, Sadigh S, Mankad K, Kapse N, Rajeswaran G. The imaging of osteomyelitis. *Quant Imaging Med Surg*. Apr 2016;6(2):184-98. doi:10.21037/qims.2016.04.01
75. Fattore J, Goh DSL, Al-Hindawi A, Andresen D. Revisiting the important role of magnetic resonance imaging (MRI) in long bone acute osteomyelitis: A case report of methicillin resistant Staphylococcus aureus acute tibial osteomyelitis with conventional radiography,

© 2022 Spellberg B et al. *JAMA Network Open.*

computed tomography, and MRI. *Radiol Case Rep*. Oct 2020;15(10):2003-2008. doi:10.1016/j.radcr.2020.07.079
76. Pugmire BS, Shailam R, Gee MS. Role of MRI in the diagnosis and treatment of osteomyelitis in pediatric patients. *World J Radiol*. Aug 28 2014;6(8):530-7. doi:10.4329/wjr.v6.i8.530
77. Howard CB, Einhorn M, Dagan R, Yagupski P, Porat S. Fine-needle bone biopsy to diagnose osteomyelitis. *J Bone Joint Surg Br*. Mar 1994;76(2):311-4.
78. Lee SJ, Won KS, Choi HJ, Choi YY. Early-Phase SPECT/CT for Diagnosing Osteomyelitis: A Retrospective Pilot Study. *Korean J Radiol*. Apr 2021;22(4):604-611. doi:10.3348/kjr.2019.0746
79. Gornitzky AL, Kim AE, O'Donnell JM, Swarup I. Diagnosis and Management of Osteomyelitis in Children: A Critical Analysis Review. *JBJS Rev*. Jun 2020;8(6):e1900202. doi:10.2106/JBJS.RVW.19.00202
80. Abdel Razek AAK, Mahmoud W. Prediction of skull base osteomyelitis in necrotising otitis externa with diffusion-weighted imaging. *J Laryngol Otol*. May 2020;134(5):404-408. doi:10.1017/S0022215120001073
81. Lesser FD, Derbyshire SG, Lewis-Jones H. Can computed tomography and magnetic resonance imaging differentiate between malignant pathology and osteomyelitis in the central skull base? *J Laryngol Otol*. Sep 2015;129(9):852-9. doi:10.1017/S0022215115001991
82. Tran K, Mierzwinski-Urban M. *Serial X-Ray Radiography for the Diagnosis of Osteomyelitis: A Review of Diagnostic Accuracy, Clinical Utility, Cost-Effectiveness, and Guidelines*. 2020. *CADTH Rapid Response Reports*.
83. Scharrenberg JS, Yagdiran A, Brinkmann J, et al. The diagnostic value of soluble urokinase-type plasminogen activator receptor (suPAR) for the discrimination of vertebral osteomyelitis and degenerative diseases of the spine. *J Orthop Surg Res*. Nov 14 2019;14(1):367. doi:10.1186/s13018-019-1420-6
84. Nickerson EK, Sinha R. Vertebral osteomyelitis in adults: an update. *Br Med Bull*. Mar 2016;117(1):121-38. doi:10.1093/bmb/ldw003
85. Amsilli M, Epaulard O. How is the microbial diagnosis of bacterial vertebral osteomyelitis performed? An 11-year retrospective study. *Eur J Clin Microbiol Infect Dis*. Nov 2020;39(11):2065-2076. doi:10.1007/s10096-020-03929-1
86. Avenel G, Guyader P, Fiaux E, et al. Microbiological diagnosis of suspected vertebral osteomyelitis with a focus on the yield of percutaneous needle biopsy: a 10-year cohort study. *Eur J Clin Microbiol Infect Dis*. Feb 2021;40(2):297-302. doi:10.1007/s10096-020-04022-3
87. Beroukhim G, Shah R, Bucknor MD. Factors Predicting Positive Culture in CT-Guided Bone Biopsy Performed for Suspected Osteomyelitis. *AJR Am J Roentgenol*. Mar 2019;212(3):620-624. doi:10.2214/AJR.18.20125
88. Garg V, Kosmas C, Young PC, Togaru UK, Robbin MR. Computed tomography-guided percutaneous biopsy for vertebral osteomyelitis: a department's experience. *Neurosurg Focus*. Aug 2014;37(2):E10. doi:10.3171/2014.6.FOCUS14134
89. Diffre C, Jousset C, Roux AL, et al. Predictive factors for positive disco-vertebral biopsy culture in pyogenic vertebral osteomyelitis, and impact of fluoroscopic versus scanographic guidance. *BMC Infect Dis*. Jul 16 2020;20(1):512. doi:10.1186/s12879-020-05223-z

© 2022 Spellberg B et al. *JAMA Network Open.*

90. Lee HT, Pukenas BA, Sebro R. Change in Bone CT Attenuation and C-reactive Protein Are Predictors of Bone Biopsy Culture Positivity in Patients With Vertebral Discitis/Osteomyelitis. *Spine (Phila Pa 1976)*. Sep 1 2020;45(17):1208-1214. doi:10.1097/BRS.0000000000003504
91. Schiro S, Foreman SC, Bucknor M, Chin CT, Joseph GB, Link TM. Diagnostic Performance of CT-Guided Bone Biopsies in Patients with Suspected Osteomyelitis of the Appendicular and Axial Skeleton with a Focus on Clinical and Technical Factors Associated with Positive Microbiology Culture Results. *J Vasc Interv Radiol*. Mar 2020;31(3):464-472. doi:10.1016/j.jvir.2019.08.013
92. Chang CY, Simeone FJ, Nelson SB, Taneja AK, Huang AJ. Is Biopsying the Paravertebral Soft Tissue as Effective as Biopsying the Disk or Vertebral Endplate? 10-Year Retrospective Review of CT-Guided Biopsy of Diskitis-Osteomyelitis. *AJR Am J Roentgenol*. Jul 2015;205(1):123-9. doi:10.2214/AJR.14.13545
93. Kasalak O, Wouthuyzen-Bakker M, Adams HJA, et al. CT-guided biopsy in suspected spondylodiscitis: microbiological yield, impact on antimicrobial treatment, and relationship with outcome. *Skeletal Radiol*. Oct 2018;47(10):1383-1391. doi:10.1007/s00256-018-2944-2
94. Lim E, Walter W, Gyftopoulos S, Samim M. Does image-guided biopsy of discitis-osteomyelitis provide meaningful information to impact clinical management? *Skeletal Radiol*. Jul 2021;50(7):1325-1336. doi:10.1007/s00256-020-03675-7
95. Choi SH, Sung H, Kim SH, et al. Usefulness of a direct 16S rRNA gene PCR assay of percutaneous biopsies or aspirates for etiological diagnosis of vertebral osteomyelitis. *Diagn Microbiol Infect Dis*. Jan 2014;78(1):75-8. doi:10.1016/j.diagmicrobio.2013.10.007
96. Hase R, Hirooka T, Itabashi T, Endo Y, Otsuka Y. Vertebral Osteomyelitis Caused by Helicobacter cinaedi Identified Using Broad-range Polymerase Chain Reaction with Sequencing of the Biopsied Specimen. *Intern Med*. May 15 2018;57(10):1475-1477. doi:10.2169/internalmedicine.0012-17
97. Alkhatib NJ, Younis MH, Alobaidi AS, Shaath NM. An unusual osteomyelitis caused by Moraxella osloensis: A case report. *Int J Surg Case Rep*. 2017;41:146-149. doi:10.1016/j.ijscr.2017.10.022
98. Shibata S, Tanizaki R, Watanabe K, et al. Escherichia coli Vertebral Osteomyelitis Diagnosed According to Broad-range 16S rRNA Gene Polymerase Chain Reaction (PCR). *Intern Med*. 2015;54(24):3237-40. doi:10.2169/internalmedicine.54.5066
99. Aragon-Sanchez J, Lipsky BA, Lazaro-Martinez JL. Diagnosing diabetic foot osteomyelitis: is the combination of probe-to-bone test and plain radiography sufficient for high-risk inpatients? *Diabet Med*. Feb 2011;28(2):191-4. doi:10.1111/j.1464-5491.2010.03150.x
100. Morales Lozano R, Gonzalez Fernandez ML, Martinez Hernandez D, Beneit Montesinos JV, Guisado Jimenez S, Gonzalez Jurado MA. Validating the probe-to-bone test and other tests for diagnosing chronic osteomyelitis in the diabetic foot. *Diabetes Care*. Oct 2010;33(10):2140-5. doi:10.2337/dc09-2309
101. Soleimani Z, Amighi F, Vakili Z, Momen-Heravi M, Moravveji SA. Diagnostic value of procalcitonin, erythrocyte sedimentation rate (ESR), quantitative C-reactive protein (CRP) and clinical findings associated with osteomyelitis in patients with diabetic foot. *Hum Antibodies*. 2021;29(2):115-121. doi:10.3233/HAB-210439

102. Schechter MC, Ali MK, Risk BB, et al. Percutaneous Bone Biopsy for Diabetic Foot Osteomyelitis: A Systematic Review and Meta-Analysis. *Open forum infectious diseases*. Oct 2020;7(10):ofaa393. doi:10.1093/ofid/ofaa393
103. Wu JS, Gorbachova T, Morrison WB, Haims AH. Imaging-guided bone biopsy for osteomyelitis: are there factors associated with positive or negative cultures? *AJR Am J Roentgenol*. Jun 2007;188(6):1529-34. doi:10.2214/AJR.06.1286
104. Tardaguila-Garcia A, Sanz-Corbalan I, Garcia-Morales E, Garcia-Alvarez Y, Molines-Barroso RJ, Lazaro-Martinez JL. Diagnostic Accuracy of Bone Culture Versus Biopsy in Diabetic Foot Osteomyelitis. *Adv Skin Wound Care*. Apr 1 2021;34(4):204-208. doi:10.1097/01.ASW.0000734376.32571.20
105. Meyr AJ, Singh S, Zhang X, et al. Statistical reliability of bone biopsy for the diagnosis of diabetic foot osteomyelitis. *J Foot Ankle Surg*. Nov-Dec 2011;50(6):663-7. doi:S1067-2516(11)00446-7 [pii]

10.1053/j.jfas.2011.08.005

106. Cecilia-Matilla A, Lazaro-Martinez JL, Aragon-Sanchez J, Garcia-Morales E, Garcia-Alvarez Y, Beneit-Montesinos JV. Histopathologic characteristics of bone infection complicating foot ulcers in diabetic patients. *J Am Podiatr Med Assoc*. Jan-Feb 2013;103(1):24-31. doi:10.7547/1030024
107. Senneville E, Melliez H, Beltrand E, et al. Culture of percutaneous bone biopsy specimens for diagnosis of diabetic foot osteomyelitis: concordance with ulcer swab cultures. *Clin Infect Dis*. Jan 1 2006;42(1):57-62. doi:10.1086/498112
108. Senneville EM, Lipsky BA, van Asten SAV, Peters EJ. Diagnosing diabetic foot osteomyelitis. *Diabetes Metab Res Rev*. Mar 2020;36 Suppl 1:e3250. doi:10.1002/dmrr.3250
109. Malone M, Fritz BG, Vickery K, et al. Analysis of proximal bone margins in diabetic foot osteomyelitis by conventional culture, DNA sequencing and microscopy. *APMIS*. Oct 2019;127(10):660-670. doi:10.1111/apm.12986
110. McNally M, Sousa R, Wouthuyzen-Bakker M, et al. The EBJIS definition of periprosthetic joint infection. *Bone Joint J*. Jan 2021;103-B(1):18-25. doi:10.1302/0301-620X.103B1.BJJ-2020-1381.R1
111. Parvizi J, Gehrke T, International Consensus Group on Periprosthetic Joint I. Definition of periprosthetic joint infection. *J Arthroplasty*. Jul 2014;29(7):1331. doi:10.1016/j.arth.2014.03.009
112. Parvizi J, Tan TL, Goswami K, et al. The 2018 Definition of Periprosthetic Hip and Knee Infection: An Evidence-Based and Validated Criteria. *J Arthroplasty*. May 2018;33(5):1309-1314 e2. doi:10.1016/j.arth.2018.02.078
113. Parvizi J, Zmistowski B, Berbari EF, et al. New definition for periprosthetic joint infection: from the Workgroup of the Musculoskeletal Infection Society. *Clin Orthop Relat Res*. Nov 2011;469(11):2992-4. doi:10.1007/s11999-011-2102-9
114. Schwarz EM, Parvizi J, Gehrke T, et al. 2018 International Consensus Meeting on Musculoskeletal Infection: Research Priorities from the General Assembly Questions. *J Orthop Res*. May 2019;37(5):997-1006. doi:10.1002/jor.24293
115. Gomez-Urena EO, Tande AJ, Osmon DR, Berbari EF. Diagnosis of Prosthetic Joint Infection: Cultures, Biomarker and Criteria. *Infect Dis Clin North Am*. Jun 2017;31(2):219-235. doi:10.1016/j.idc.2017.01.008

© 2022 Spellberg B et al. *JAMA Network Open.*

116. Tsaras G, Osmon DR, Mabry T, et al. Incidence, secular trends, and outcomes of prosthetic joint infection: a population-based study, olmsted county, Minnesota, 1969-2007. *Infection control and hospital epidemiology : the official journal of the Society of Hospital Epidemiologists of America*. Dec 2012;33(12):1207-12. doi:10.1086/668421
117. Romano CL, Petrosillo N, Argento G, et al. The Role of Imaging Techniques to Define a Peri-Prosthetic Hip and Knee Joint Infection: Multidisciplinary Consensus Statements. *J Clin Med*. Aug 6 2020;9(8)doi:10.3390/jcm9082548
118. Plate A, Weichselbaumer V, Schupbach R, et al. Diagnostic accuracy of (99m)Tc-antigranulocyte SPECT/CT in patients with osteomyelitis and orthopaedic device-related infections: A retrospective analysis. *International journal of infectious diseases : IJID : official publication of the International Society for Infectious Diseases*. Feb 2020;91:79-86. doi:10.1016/j.ijid.2019.11.017
119. Wenter V, Muller JP, Albert NL, et al. The diagnostic value of [(18)F]FDG PET for the detection of chronic osteomyelitis and implant-associated infection. *Eur J Nucl Med Mol Imaging*. Apr 2016;43(4):749-61. doi:10.1007/s00259-015-3221-4
120. Ahmad SS, Shaker A, Saffarini M, Chen AF, Hirschmann MT, Kohl S. Accuracy of diagnostic tests for prosthetic joint infection: a systematic review. *Knee Surg Sports Traumatol Arthrosc*. Oct 2016;24(10):3064-3074. doi:10.1007/s00167-016-4230-y
121. Shahi A, Kheir MM, Tarabichi M, Hosseinzadeh HRS, Tan TL, Parvizi J. Serum D-Dimer Test Is Promising for the Diagnosis of Periprosthetic Joint Infection and Timing of Reimplantation. *J Bone Joint Surg Am*. Sep 6 2017;99(17):1419-1427. doi:10.2106/JBJS.16.01395
122. Margaretten ME, Kohlwes J, Moore D, Bent S. Does this adult patient have septic arthritis? *JAMA : the journal of the American Medical Association*. Apr 4 2007;297(13):1478-88. doi:10.1001/jama.297.13.1478
123. Christensen CP, Bedair H, Della Valle CJ, Parvizi J, Schurko B, Jacobs CA. The natural progression of synovial fluid white blood-cell counts and the percentage of polymorphonuclear cells after primary total knee arthroplasty: a multicenter study. *J Bone Joint Surg Am*. Dec 4 2013;95(23):2081-7. doi:10.2106/JBJS.L.01646
124. Parvizi J, Jacovides C, Antoci V, Ghanem E. Diagnosis of periprosthetic joint infection: the utility of a simple yet unappreciated enzyme. *J Bone Joint Surg Am*. Dec 21 2011;93(24):2242-8. doi:10.2106/JBJS.J.01413
125. Li Z, Zhang Q, Shi L, Gao F, Sun W, Li Z. Alpha-Defensin versus Leukocyte Esterase in Periprosthetic Joint Infection: An Updated Meta-Analysis. *Biomed Res Int*. 2020;2020:3704285. doi:10.1155/2020/3704285
126. Tischler EH, Plummer DR, Chen AF, Della Valle CJ, Parvizi J. Leukocyte Esterase: Metal-on-Metal Failure and Periprosthetic Joint Infection. *J Arthroplasty*. Oct 2016;31(10):2260-3. doi:10.1016/j.arth.2016.03.012
127. Sayan A, Kopiec A, Shahi A, Chowdhry M, Bullock M, Oliashirazi A. The Expanding Role of Biomarkers in Diagnosing Infection in Total Joint Arthroplasty: A Review of Current Literature. *Arch Bone Jt Surg*. Jan 2021;9(1):33-43. doi:10.22038/abjs.2020.42989.2169
128. Wang C, Wang Q, Li R, Duan JY, Wang CB. Synovial Fluid C-reactive Protein as a Diagnostic Marker for Periprosthetic Joint Infection: A Systematic Review and Meta-analysis. *Chin Med J (Engl)*. Aug 20 2016;129(16):1987-93. doi:10.4103/0366-6999.187857

© 2022 Spellberg B et al. *JAMA Network Open.*

129. Xie K, Dai K, Qu X, Yan M. Serum and Synovial Fluid Interleukin-6 for the Diagnosis of Periprosthetic Joint Infection. *Sci Rep*. May 4 2017;7(1):1496. doi:10.1038/s41598-017-01713-4
130. Zhao X, Guo C, Zhao GS, Lin T, Shi ZL, Yan SG. Ten versus five polymorphonuclear leukocytes as threshold in frozen section tests for periprosthetic infection: a meta-analysis. *J Arthroplasty*. Jun 2013;28(6):913-7. doi:10.1016/j.arth.2012.10.015
131. Barrack R, Bhimani S, Blevins JL, et al. General Assembly, Diagnosis, Laboratory Test: Proceedings of International Consensus on Orthopedic Infections. *J Arthroplasty*. Feb 2019;34(2S):S187-S195. doi:10.1016/j.arth.2018.09.070
132. Aggarwal VK, Higuera C, Deirmengian G, Parvizi J, Austin MS. Swab cultures are not as effective as tissue cultures for diagnosis of periprosthetic joint infection. *Clin Orthop Relat Res*. Oct 2013;471(10):3196-203. doi:10.1007/s11999-013-2974-y
133. Atkins BL, Athanasou N, Deeks JJ, et al. Prospective evaluation of criteria for microbiological diagnosis of prosthetic-joint infection at revision arthroplasty. The OSIRIS Collaborative Study Group. *J Clin Microbiol*. Oct 1998;36(10):2932-9. doi:10.1128/JCM.36.10.2932-2939.1998
134. Gamie Z, Karthikappallil D, Gamie E, Stamiris S, Kenanidis E, Tsiridis E. Molecular sequencing technologies in the diagnosis and management of prosthetic joint infections. *Expert Rev Mol Diagn*. Mar 1 2021;doi:10.1080/14737159.2021.1894929
135. Stephan A, Thurmer A, Glauche I, Nowotny J, Zwingenberger S, Stiehler M. Does preoperative antibiotic prophylaxis affect sonication-based diagnosis in implant-associated infection? *J Orthop Res*. Feb 23 2021;doi:10.1002/jor.25015
136. Sambri A, Cadossi M, Giannini S, et al. Is Treatment With Dithiothreitol More Effective Than Sonication for the Diagnosis of Prosthetic Joint Infection? *Clin Orthop Relat Res*. Jan 2018;476(1):137-145. doi:10.1007/s11999.0000000000000060
137. Sebastian S, Malhotra R, Sreenivas V, Kapil A, Chaudhry R, Dhawan B. Sonication of orthopaedic implants: A valuable technique for diagnosis of prosthetic joint infections. *J Microbiol Methods*. Mar 2018;146:51-54. doi:10.1016/j.mimet.2018.01.015
138. Prieto-Borja L, Aunon A, Blanco A, et al. Evaluation of the use of sonication of retrieved implants for the diagnosis of prosthetic joint infection in a routine setting. *Eur J Clin Microbiol Infect Dis*. Apr 2018;37(4):715-722. doi:10.1007/s10096-017-3164-8
139. Tani S, Lepetsos P, Stylianakis A, Vlamis J, Birbas K, Kaklamanos I. Superiority of the sonication method against conventional periprosthetic tissue cultures for diagnosis of prosthetic joint infections. *Eur J Orthop Surg Traumatol*. Jan 2018;28(1):51-57. doi:10.1007/s00590-017-2012-y
140. Dudareva M, Barrett L, Figtree M, et al. Sonication versus Tissue Sampling for Diagnosis of Prosthetic Joint and Other Orthopedic Device-Related Infections. *J Clin Microbiol*. Dec 2018;56(12)doi:10.1128/JCM.00688-18
141. Yan Q, Karau MJ, Greenwood-Quaintance KE, et al. Comparison of Diagnostic Accuracy of Periprosthetic Tissue Culture in Blood Culture Bottles to That of Prosthesis Sonication Fluid Culture for Diagnosis of Prosthetic Joint Infection (PJI) by Use of Bayesian Latent Class Modeling and IDSA PJI Criteria for Classification. *J Clin Microbiol*. Jun 2018;56(6)doi:10.1128/JCM.00319-18
142. Romano CL, Trentinaglia MT, De Vecchi E, et al. Cost-benefit analysis of antibiofilm microbiological techniques for peri-prosthetic joint infection diagnosis. *BMC Infect Dis*. Apr 2 2018;18(1):154. doi:10.1186/s12879-018-3050-8

© 2022 Spellberg B et al. *JAMA Network Open.*

143. Lausmann C, Kolle KN, Citak M, et al. How reliable is the next generation of multiplex-PCR for diagnosing prosthetic joint infection compared to the MSIS criteria? Still missing the ideal test. *Hip Int*. Sep 2020;30(1_suppl):72-77. doi:10.1177/1120700020938576
144. Huang Z, Li W, Lee GC, et al. Metagenomic next-generation sequencing of synovial fluid demonstrates high accuracy in prosthetic joint infection diagnostics: mNGS for diagnosing PJI. *Bone Joint Res*. Jul 2020;9(7):440-449. doi:10.1302/2046-3758.97.BJR-2019-0325.R2
145. Cai Y, Fang X, Chen Y, et al. Metagenomic next generation sequencing improves diagnosis of prosthetic joint infection by detecting the presence of bacteria in periprosthetic tissues. *International journal of infectious diseases : IJID : official publication of the International Society for Infectious Diseases*. Jul 2020;96:573-578. doi:10.1016/j.ijid.2020.05.125
146. Suren C, Feihl S, Cabric S, et al. Improved pre-operative diagnostic accuracy for low-grade prosthetic joint infections using second-generation multiplex Polymerase chain reaction on joint fluid aspirate. *Int Orthop*. Sep 2020;44(9):1629-1637. doi:10.1007/s00264-020-04552-7
147. Stylianakis A, Schinas G, Thomaidis PC, et al. Combination of conventional culture, vial culture, and broad-range PCR of sonication fluid for the diagnosis of prosthetic joint infection. *Diagn Microbiol Infect Dis*. Sep 2018;92(1):13-18. doi:10.1016/j.diagmicrobio.2018.04.008
148. Wang CX, Huang Z, Fang X, Li W, Yang B, Zhang W. Comparison of broad-range polymerase chain reaction and metagenomic next-generation sequencing for the diagnosis of prosthetic joint infection. *International journal of infectious diseases : IJID : official publication of the International Society for Infectious Diseases*. Jun 2020;95:8-12. doi:10.1016/j.ijid.2020.03.055
149. Lane MA, Ganeshraj N, Gu A, Warren DK, Burnham CD. Lack of Additional Diagnostic Yield of 16s rRNA Gene PCR for Prosthetic Joint Infections. *J Appl Lab Med*. Sep 2019;4(2):224-228. doi:10.1373/jalm.2018.027003
150. Sebastian S, Malhotra R, Sreenivas V, Kapil A, Chaudhry R, Dhawan B. Utility of 16S rRNA PCR in the Synovial Fluid for the Diagnosis of Prosthetic Joint Infection. *Ann Lab Med*. Nov 2018;38(6):610-612. doi:10.3343/alm.2018.38.6.610
151. Larsen LH, Khalid V, Xu Y, Thomsen TR, Schonheyder HC, the PSG. Differential Contributions of Specimen Types, Culturing, and 16S rRNA Sequencing in Diagnosis of Prosthetic Joint Infections. *J Clin Microbiol*. May 2018;56(5)doi:10.1128/JCM.01351-17
152. Malandain D, Bemer P, Leroy AG, et al. Assessment of the automated multiplex-PCR Unyvero i60 ITI((R)) cartridge system to diagnose prosthetic joint infection: a multicentre study. *Clin Microbiol Infect*. Jan 2018;24(1):83 e1-83 e6. doi:10.1016/j.cmi.2017.05.017
153. Bemer P, Plouzeau C, Tande D, et al. Evaluation of 16S rRNA gene PCR sensitivity and specificity for diagnosis of prosthetic joint infection: a prospective multicenter cross-sectional study. *J Clin Microbiol*. Oct 2014;52(10):3583-9. doi:10.1128/JCM.01459-14
154. Ryu SY, Greenwood-Quaintance KE, Hanssen AD, Mandrekar JN, Patel R. Low sensitivity of periprosthetic tissue PCR for prosthetic knee infection diagnosis. *Diagn Microbiol Infect Dis*. Aug 2014;79(4):448-53. doi:10.1016/j.diagmicrobio.2014.03.021
155. Zhang Y, Feng S, Chen W, Zhang QC, Shi SF, Chen XY. Advantages of 16S rRNA PCR for the diagnosis of prosthetic joint infection. *Exp Ther Med*. Oct 2020;20(4):3104-3113. doi:10.3892/etm.2020.9082

156. Li M, Zeng Y, Wu Y, Si H, Bao X, Shen B. Performance of Sequencing Assays in Diagnosis of Prosthetic Joint Infection: A Systematic Review and Meta-Analysis. *J Arthroplasty*. Jul 2019;34(7):1514-1522 e4. doi:10.1016/j.arth.2019.02.044
157. Liu K, Fu J, Yu B, Sun W, Chen J, Hao L. Meta-analysis of sonication prosthetic fluid PCR for diagnosing periprosthetic joint infection. *PLoS One*. 2018;13(4):e0196418. doi:10.1371/journal.pone.0196418
158. Ivy MI, Thoendel MJ, Jeraldo PR, et al. Direct Detection and Identification of Prosthetic Joint Infection Pathogens in Synovial Fluid by Metagenomic Shotgun Sequencing. *J Clin Microbiol*. Sep 2018;56(9)doi:10.1128/JCM.00402-18
159. Thoendel MJ, Jeraldo PR, Greenwood-Quaintance KE, et al. Identification of Prosthetic Joint Infection Pathogens Using a Shotgun Metagenomics Approach. *Clin Infect Dis*. Oct 15 2018;67(9):1333-1338. doi:10.1093/cid/ciy303
160. Turk EE, Tsokos M, Delling G. Autopsy-based assessment of extent and type of osteomyelitis in advanced-grade sacral decubitus ulcers: a histopathologic study. *Arch Pathol Lab Med*. Dec 2003;127(12):1599-602. doi:10.1043/1543-2165(2003)127<1599:AAOEAT>2.0.CO;2
161. Darouiche RO, Landon GC, Klima M, Musher DM, Markowski J. Osteomyelitis associated with pressure sores. *Arch Intern Med*. Apr 11 1994;154(7):753-8.
162. Sugarman B, Hawes S, Musher DM, Klima M, Young EJ, Pircher F. Osteomyelitis beneath pressure sores. *Arch Intern Med*. Apr 1983;143(4):683-8.
163. Russell CD, Tsang SJ, Simpson A, Sutherland RK. Outcomes, Microbiology and Antimicrobial Usage in Pressure Ulcer-Related Pelvic Osteomyelitis: Messages for Clinical Practice. *J Bone Jt Infect*. 2020;5(2):67-75. doi:10.7150/jbji.41779
164. Chicco M, Singh P, Beitverda Y, Williams G, Hirji H, Rao GG. Diagnosing pelvic osteomyelitis in patients with pressure ulcers: a systematic review comparing bone histology with alternative diagnostic modalities. *J Bone Jt Infect*. 2020;6(1):21-32. doi:10.5194/jbji-6-21-2020
165. Thornhill-Joynes M, Gonzales F, Stewart CA, et al. Osteomyelitis associated with pressure ulcers. *Arch Phys Med Rehabil*. May 1986;67(5):314-8.
166. Brunel AS, Lamy B, Cyteval C, et al. Diagnosing pelvic osteomyelitis beneath pressure ulcers in spinal cord injured patients: a prospective study. *Clin Microbiol Infect*. Mar 2016;22(3):267 e1-8. doi:10.1016/j.cmi.2015.11.005
167. McCarthy J, Hartmann E, Bentz ML, et al. Seeing is Believing? Preoperative Magnetic Resonance Imaging for Pressure Ulcers: Implications for Surgical Management. *Plast Reconstr Surg Glob Open*. Mar 2017;5(3):e1263. doi:10.1097/GOX.0000000000001263
168. Larson DL, Gilstrap J, Simonelic K, Carrera GF. Is there a simple, definitive, and cost-effective way to diagnose osteomyelitis in the pressure ulcer patient? *Plast Reconstr Surg*. Feb 2011;127(2):670-6. doi:10.1097/PRS.0b013e3181fed66e
169. Lewis VL, Jr., Bailey MH, Pulawski G, Kind G, Bashioum RW, Hendrix RW. The diagnosis of osteomyelitis in patients with pressure sores. *Plast Reconstr Surg*. Feb 1988;81(2):229-32.
170. Merine D, Fishman EK, Magid D. CT detection of sacral osteomyelitis associated with pelvic abscesses. *J Comput Assist Tomogr*. Jan-Feb 1988;12(1):118-21. doi:10.1097/00004728-198801000-00023

© 2022 Spellberg B et al. *JAMA Network Open.*

171. Huang AB, Schweitzer ME, Hume E, Batte WG. Osteomyelitis of the pelvis/hips in paralyzed patients: accuracy and clinical utility of MRI. *J Comput Assist Tomogr*. May-Jun 1998;22(3):437-43. doi:10.1097/00004728-199805000-00017
172. Marriott R, Rubayi S. Successful truncated osteomyelitis treatment for chronic osteomyelitis secondary to pressure ulcers in spinal cord injury patients. *Ann Plast Surg*. Oct 2008;61(4):425-9. doi:10.1097/SAP.0b013e318162f257
173. Jugun K, Richard JC, Lipsky BA, et al. Factors Associated With Treatment Failure of Infected Pressure Sores. *Ann Surg*. Aug 2016;264(2):399-403. doi:10.1097/SLA.0000000000001497
174. Goodman CM, Cohen V, Armenta A, Thornby J, Netscher DT. Evaluation of results and treatment variables for pressure ulcers in 48 veteran spinal cord-injured patients. *Ann Plast Surg*. Jun 1999;42(6):665-72. doi:10.1097/00000637-199906000-00015
175. Firriolo JM, Ganske IM, Pike CM, et al. Long-term Outcomes After Flap Reconstruction in Pediatric Pressure Ulcers. *Ann Plast Surg*. Feb 2018;80(2):159-163. doi:10.1097/SAP.0000000000001226
176. Larson DL, Hudak KA, Waring WP, Orr MR, Simonelic K. Protocol management of late-stage pressure ulcers: a 5-year retrospective study of 101 consecutive patients with 179 ulcers. *Plast Reconstr Surg*. Apr 2012;129(4):897-904. doi:10.1097/PRS.0b013e3182442197
177. Cunha BA. Osteomyelitis in elderly patients. *Clin Infect Dis*. Aug 1 2002;35(3):287-93. doi:10.1086/341417
178. Dudareva M, Ferguson J, Riley N, Stubbs D, Atkins B, McNally M. Osteomyelitis of the Pelvic Bones: A Multidisciplinary Approach to Treatment. *J Bone Jt Infect*. 2017;2(4):184-193. doi:10.7150/jbji.21692
179. Bamba R, Madden JJ, Hoffman AN, et al. Flap Reconstruction for Pressure Ulcers: An Outcomes Analysis. *Plast Reconstr Surg Glob Open*. Jan 2017;5(1):e1187. doi:10.1097/GOX.0000000000001187
180. Cooper EA, Bonne Lee B, Muhlmann M. Outcomes following stoma formation in patients with spinal cord injury. *Colorectal Dis*. Dec 2019;21(12):1415-1420. doi:10.1111/codi.14753
181. de la Fuente SG, Levin LS, Reynolds JD, et al. Elective stoma construction improves outcomes in medically intractable pressure ulcers. *Dis Colon Rectum*. Nov 2003;46(11):1525-30. doi:10.1007/s10350-004-6808-6
182. Parsons B, Strauss E. Surgical management of chronic osteomyelitis. *American journal of surgery*. Jul 2004;188(1A Suppl):57-66. doi:10.1016/S0002-9610(03)00292-7
183. Mader JT, Cripps MW, Calhoun JH. Adult posttraumatic osteomyelitis of the tibia. *Clin Orthop Relat Res*. Mar 1999;(360):14-21.
184. Scarborough M, Li HK, Rombach I, et al. ORAL VERSUS INTRAVENOUS ANTIBIOTICS FOR THE TREATMENT OF BONE AND JOINT INFECTION (OVIVA): A MULTICENTRE RANDOMISED CONTROLLED TRIAL. *Orthopaedic Proceedings*. 2018;ePub
185. de Lucas EM, Gonzalez Mandly A, Gutierrez A, et al. CT-guided fine-needle aspiration in vertebral osteomyelitis: true usefulness of a common practice. *Clin Rheumatol*. Mar 2009;28(3):315-20. doi:10.1007/s10067-008-1051-5

© 2022 Spellberg B et al. *JAMA Network Open.*

186. Rankine JJ, Barron DA, Robinson P, Millner PA, Dickson RA. Therapeutic impact of percutaneous spinal biopsy in spinal infection. *Postgraduate medical journal*. Oct 2004;80(948):607-9. doi:10.1136/pgmj.2003.017863
187. Marschall J, Bhavan KP, Olsen MA, Fraser VJ, Wright NM, Warren DK. The impact of prebiopsy antibiotics on pathogen recovery in hematogenous vertebral osteomyelitis. *Clin Infect Dis*. Apr 1 2011;52(7):867-72. doi:10.1093/cid/cir062
188. Lopes Floro K, Munckhof W, Coucher J. Retrospective review of CT-guided intervertebral disc biopsies performed at a tertiary referral centre for suspected osteodiscitis. *J Med Imaging Radiat Oncol*. Jun 2018;62(3):307-312. doi:10.1111/1754-9485.12686
189. Agarwal V, Wo S, Lagemann GM, Tsay J, Delfyett WT. Image-guided percutaneous disc sampling: impact of antecedent antibiotics on yield. *Clin Radiol*. Mar 2016;71(3):228-34. doi:10.1016/j.crad.2015.10.031
190. Wong H, Tarr GP, Rajpal K, Sweetman L, Doyle A. The impact of antibiotic pre-treatment on diagnostic yield of CT-guided biopsy for spondylodiscitis: A multi-centre retrospective study and meta-analysis. *J Med Imaging Radiat Oncol*. Apr 2021;65(2):146-151. doi:10.1111/1754-9485.13118
191. McNamara AL, Dickerson EC, Gomez-Hassan DM, Cinti SK, Srinivasan A. Yield of Image-Guided Needle Biopsy for Infectious Discitis: A Systematic Review and Meta-Analysis. *AJNR Am J Neuroradiol*. Oct 2017;38(10):2021-2027. doi:10.3174/ajnr.A5337
192. Cheng MP, Stenstrom R, Paquette K, et al. Blood Culture Results Before and After Antimicrobial Administration in Patients With Severe Manifestations of Sepsis: A Diagnostic Study. *Annals of internal medicine*. Oct 15 2019;171(8):547-554. doi:10.7326/M19-1696
193. Lesens O, Desbiez F, Vidal M, et al. Culture of per-wound bone specimens: a simplified approach for the medical management of diabetic foot osteomyelitis. *Clin Microbiol Infect*. Feb 2011;17(2):285-91. doi:10.1111/j.1469-0691.2010.03194.x
194. Letertre-Gibert P, Desbiez F, Vidal M, et al. Blood cultures after bone biopsy in diabetic foot osteomyelitis. *Diagn Microbiol Infect Dis*. Sep 2017;89(1):78-79. doi:10.1016/j.diagmicrobio.2017.02.011
195. Couturier A, Chabaud A, Desbiez F, et al. Comparison of microbiological results obtained from per-wound bone biopsies versus transcutaneous bone biopsies in diabetic foot osteomyelitis: a prospective cohort study. *Eur J Clin Microbiol Infect Dis*. Jul 2019;38(7):1287-1291. doi:10.1007/s10096-019-03547-6
196. Senneville E, Morant H, Descamps D, et al. Needle puncture and transcutaneous bone biopsy cultures are inconsistent in patients with diabetes and suspected osteomyelitis of the foot. *Clin Infect Dis*. Apr 1 2009;48(7):888-93. doi:10.1086/597263
197. Aslangul E, M'Bemba J, Caillat-Vigneron N, et al. Diagnosing diabetic foot osteomyelitis in patients without signs of soft tissue infection by coupling hybrid 67Ga SPECT/CT with bedside percutaneous bone puncture. *Diabetes care*. Aug 2013;36(8):2203-10. doi:10.2337/dc12-2108
198. Ducloux R, Tazi O, Abou-Rjeili A, Abouth S, al. e. Percutaneous Bone Biopsy to Identify Pathogens in Diabetic Foot Chronic Osteitis: Useful and Harmless *Wounds*. 2016;28:182-193.
199. Aragon-Sanchez FJ, Cabrera-Galvan JJ, Quintana-Marrero Y, et al. Outcomes of surgical treatment of diabetic foot osteomyelitis: a series of 185 patients with histopathological

© 2022 Spellberg B et al. *JAMA Network Open.*

confirmation of bone involvement. *Diabetologia*. Nov 2008;51(11):1962-70. doi:10.1007/s00125-008-1131-8

200. Senneville E, Lombart A, Beltrand E, et al. Outcome of diabetic foot osteomyelitis treated nonsurgically: a retrospective cohort study. *Diabetes care*. Apr 2008;31(4):637-42. doi:dc07-1744 [pii]

10.2337/dc07-1744

201. Malekzadeh D, Osmon DR, Lahr BD, Hanssen AD, Berbari EF. Prior use of antimicrobial therapy is a risk factor for culture-negative prosthetic joint infection. *Clin Orthop Relat Res*. Aug 2010;468(8):2039-45. doi:10.1007/s11999-010-1338-0
202. Shahi A, Deirmengian C, Higuera C, et al. Premature Therapeutic Antimicrobial Treatments Can Compromise the Diagnosis of Late Periprosthetic Joint Infection. *Clin Orthop Relat Res*. Jul 2015;473(7):2244-9. doi:10.1007/s11999-015-4142-z
203. Trampuz A, Piper KE, Jacobson MJ, et al. Sonication of removed hip and knee prostheses for diagnosis of infection. *N Engl J Med*. Aug 16 2007;357(7):654-63. doi:10.1056/NEJMoa061588
204. Watanabe S, Kobayashi N, Tomoyama A, Choe H, Yamazaki E, Inaba Y. Clinical characteristics and risk factors for culture-negative periprosthetic joint infections. *J Orthop Surg Res*. May 3 2021;16(1):292. doi:10.1186/s13018-021-02450-1
205. Beldman M, Lowik C, Soriano A, et al. If, When, and How to Use Rifampin in Acute Staphylococcal Periprosthetic Joint Infections, a Multicentre Observational Study. *Clin Infect Dis*. Nov 2 2021;73(9):1634-1641. doi:10.1093/cid/ciab426
206. Mylona E, Samarkos M, Kakalou E, Fanourgiakis P, Skoutelis A. Pyogenic vertebral osteomyelitis: a systematic review of clinical characteristics. *Semin Arthritis Rheum*. Aug 2009;39(1):10-7. doi:10.1016/j.semarthrit.2008.03.002
207. Park KH, Kim DY, Lee YM, et al. Selection of an appropriate empiric antibiotic regimen in hematogenous vertebral osteomyelitis. *PLoS One*. 2019;14(2):e0211888. doi:10.1371/journal.pone.0211888
208. Bhavan KP, Marschall J, Olsen MA, Fraser VJ, Wright NM, Warren DK. The epidemiology of hematogenous vertebral osteomyelitis: a cohort study in a tertiary care hospital. *BMC Infect Dis*. Jun 7 2010;10:158. doi:10.1186/1471-2334-10-158
209. Kim CJ, Song KH, Park WB, et al. Microbiologically and clinically diagnosed vertebral osteomyelitis: impact of prior antibiotic exposure. *Antimicrob Agents Chemother*. Apr 2012;56(4):2122-4. doi:10.1128/AAC.05953-11
210. Butler-Laporte G, Cheng MP, McDonald EG, Lee TC. Screening swabs surpass traditional risk factors as predictors of MRSA bacteremia. *BMC Infect Dis*. Jun 11 2018;18(1):270. doi:10.1186/s12879-018-3182-x
211. Elamurugan TP, Jagdish S, Kate V, Chandra Parija S. Role of bone biopsy specimen culture in the management of diabetic foot osteomyelitis. *Int J Surg*. 2011;9(3):214-6. doi:10.1016/j.ijsu.2010.11.011
212. Wheat LJ, Allen SD, Henry M, et al. Diabetic foot infections. Bacteriologic analysis. *Arch Intern Med*. Oct 1986;146(10):1935-40.
213. Bamberger DM, Daus GP, Gerding DN. Osteomyelitis in the feet of diabetic patients. Long-term results, prognostic factors, and the role of antimicrobial and surgical therapy. *Am J Med*. Oct 1987;83(4):653-60. doi:10.1016/0002-9343(87)90894-1

© 2022 Spellberg B et al. *JAMA Network Open.*

214. Lavery LA, Sariaya M, Ashry H, Harkless LB. Microbiology of osteomyelitis in diabetic foot infections. *J Foot Ankle Surg*. Jan-Feb 1995;34(1):61-4. doi:10.1016/S1067-2516(09)80103-8
215. Tan JS, Friedman NM, Hazelton-Miller C, Flanagan JP, File TM, Jr. Can aggressive treatment of diabetic foot infections reduce the need for above-ankle amputation? *Clin Infect Dis*. Aug 1996;23(2):286-91. doi:10.1093/clinids/23.2.286
216. Lesens O, Desbiez F, Theis C, et al. Staphylococcus aureus-Related Diabetic Osteomyelitis: Medical or Surgical Management? A French and Spanish Retrospective Cohort. *Int J Low Extrem Wounds*. Sep 2015;14(3):284-90. doi:10.1177/1534734614559931
217. Charles PG, Uckay I, Kressmann B, Emonet S, Lipsky BA. The role of anaerobes in diabetic foot infections. *Anaerobe*. Aug 2015;34:8-13. doi:10.1016/j.anaerobe.2015.03.009
218. Aragon-Sanchez J, Lipsky BA, Lazaro-Martinez JL. Gram-negative diabetic foot osteomyelitis: risk factors and clinical presentation. *Int J Low Extrem Wounds*. Mar 2013;12(1):63-8. doi:10.1177/1534734613477423
219. van Asten SA, La Fontaine J, Peters EJ, Bhavan K, Kim PJ, Lavery LA. The microbiome of diabetic foot osteomyelitis. *Eur J Clin Microbiol Infect Dis*. Feb 2016;35(2):293-8. doi:10.1007/s10096-015-2544-1
220. Zenelaj B, Bouvet C, Lipsky BA, Uckay I. Do diabetic foot infections with methicillin-resistant Staphylococcus aureus differ from those with other pathogens? *Int J Low Extrem Wounds*. Dec 2014;13(4):263-72. doi:10.1177/1534734614550311
221. Widatalla AH, Mahadi SE, Shawer MA, Mahmoud SM, Abdelmageed AE, Ahmed ME. Diabetic foot infections with osteomyelitis: efficacy of combined surgical and medical treatment. *Diabet Foot Ankle*. 2012;3doi:10.3402/dfa.v3i0.18809
222. Parvez N, Dutta P, Ray P, et al. Microbial profile and utility of soft tissue, pus, and bone cultures in diagnosing diabetic foot infections. *Diabetes Technol Ther*. Aug 2012;14(8):669-74. doi:10.1089/dia.2012.0039
223. Crockarell JR, Hanssen AD, Osmon DR, Morrey BF. Treatment of infection with debridement and retention of the components following hip arthroplasty. *J Bone Joint Surg Am*. Sep 1998;80(9):1306-13. doi:10.2106/00004623-199809000-00009
224. Berdal JE, Skramm I, Mowinckel P, Gulbrandsen P, Bjornholt JV. Use of rifampicin and ciprofloxacin combination therapy after surgical debridement in the treatment of early manifestation prosthetic joint infections. *Clin Microbiol Infect*. Oct 2005;11(10):843-5. doi:10.1111/j.1469-0691.2005.01230.x
225. Holmberg A, Thorhallsdottir VG, Robertsson O, A WD, Stefansdottir A. 75% success rate after open debridement, exchange of tibial insert, and antibiotics in knee prosthetic joint infections. *Acta Orthop*. 2015;86(4):457-62. doi:10.3109/17453674.2015.1026756
226. Rao N, Crossett LS, Sinha RK, Le Frock JL. Long-term suppression of infection in total joint arthroplasty. *Clin Orthop Relat Res*. Sep 2003;(414):55-60. doi:10.1097/01.blo.0000087321.60612.cf
227. Geurts JA, Janssen DM, Kessels AG, Walenkamp GH. Good results in postoperative and hematogenous deep infections of 89 stable total hip and knee replacements with retention of prosthesis and local antibiotics. *Acta Orthop*. Dec 2013;84(6):509-16. doi:10.3109/17453674.2013.858288
228. Harris LG, El-Bouri K, Johnston S, et al. Rapid identification of staphylococci from prosthetic joint infections using MALDI-TOF mass-spectrometry. *Int J Artif Organs*. Sep 2010;33(9):568-74. doi:10.1177/039139881003300902

© 2022 Spellberg B et al. *JAMA Network Open.*

229. Sperling JW, Kozak TK, Hanssen AD, Cofield RH. Infection after shoulder arthroplasty. *Clin Orthop Relat Res*. Jan 2001;(382):206-16. doi:10.1097/00003086-200101000-00028
230. Cuff DJ, Virani NA, Levy J, et al. The treatment of deep shoulder infection and glenohumeral instability with debridement, reverse shoulder arthroplasty and postoperative antibiotics. *J Bone Joint Surg Br*. Mar 2008;90(3):336-42. doi:10.1302/0301-620X.90B3.19408
231. Coste JS, Reig S, Trojani C, Berg M, Walch G, Boileau P. The management of infection in arthroplasty of the shoulder. *J Bone Joint Surg Br*. Jan 2004;86(1):65-9.
232. Verhelst L, Stuyck J, Bellemans J, Debeer P. Resection arthroplasty of the shoulder as a salvage procedure for deep shoulder infection: does the use of a cement spacer improve outcome? *J Shoulder Elbow Surg*. Dec 2011;20(8):1224-33. doi:10.1016/j.jse.2011.02.003
233. Piper KE, Jacobson MJ, Cofield RH, et al. Microbiologic diagnosis of prosthetic shoulder infection by use of implant sonication. *J Clin Microbiol*. Jun 2009;47(6):1878-84. doi:10.1128/JCM.01686-08
234. Sabesan VJ, Ho JC, Kovacevic D, Iannotti JP. Two-stage reimplantation for treating prosthetic shoulder infections. *Clin Orthop Relat Res*. Sep 2011;469(9):2538-43. doi:10.1007/s11999-011-1774-5
235. Dodson CC, Craig EV, Cordasco FA, et al. Propionibacterium acnes infection after shoulder arthroplasty: a diagnostic challenge. *J Shoulder Elbow Surg*. Mar 2010;19(2):303-7. doi:10.1016/j.jse.2009.07.065
236. Klatte TO, Junghans K, Al-Khateeb H, et al. Single-stage revision for peri-prosthetic shoulder infection: outcomes and results. *Bone Joint J*. Mar 2013;95-B(3):391-5. doi:10.1302/0301-620X.95B3.30134
237. Tornero E, Soriano A. Importance of selection and duration of antibiotic regimen in prosthetic joint infections treated with debridement and implant retention-authors' response. *J Antimicrob Chemother*. Dec 2016;71(12):3627. doi:10.1093/jac/dkw438
238. Moran E, Masters S, Berendt AR, McLardy-Smith P, Byren I, Atkins BL. Guiding empirical antibiotic therapy in orthopaedics: The microbiology of prosthetic joint infection managed by debridement, irrigation and prosthesis retention. *J Infect*. Jul 2007;55(1):1-7. doi:10.1016/j.jinf.2007.01.007
239. Benito N, Franco M, Ribera A, et al. Time trends in the aetiology of prosthetic joint infections: a multicentre cohort study. *Clin Microbiol Infect*. Aug 2016;22(8):732 e1-8. doi:10.1016/j.cmi.2016.05.004
240. Davis JS, Metcalf S, Paterson DL, Robinson JO, Clarke B, Manning L. Proposed empiric antibiotic therapy for prosthetic joint infections: an analysis of the Prosthetic Joint Infection in Australia and New Zealand, Observational (PIANO) cohort. *Intern Med J*. Feb 2022;52(2):322-325. doi:10.1111/imj.15677
241. Azzam K, Parvizi J, Jungkind D, et al. Microbiological, clinical, and surgical features of fungal prosthetic joint infections: a multi-institutional experience. *J Bone Joint Surg Am*. Nov 2009;91 Suppl 6:142-9. doi:10.2106/JBJS.I.00574
242. Cutrona AF, Shah M, Himes MS, Miladore MA. Rhodotorula minuta: an unusual fungal infection in hip-joint prosthesis. *Am J Orthop (Belle Mead NJ)*. Mar 2002;31(3):137-40.
243. Savini V, Sozio F, Catavitello C, et al. Femoral prosthesis infection by Rhodotorula mucilaginosa. *J Clin Microbiol*. Oct 2008;46(10):3544-5. doi:10.1128/JCM.00873-08
244. Byren I, Rege S, Campanaro E, et al. Randomized controlled trial of the safety and efficacy of Daptomycin versus standard-of-care therapy for management of patients with

osteomyelitis associated with prosthetic devices undergoing two-stage revision arthroplasty. *Antimicrob Agents Chemother*. Nov 2012;56(11):5626-32. doi:10.1128/AAC.00038-12
245. Kourtis AP, Hatfield K, Baggs J, et al. Vital Signs: Epidemiology and Recent Trends in Methicillin-Resistant and in Methicillin-Susceptible Staphylococcus aureus Bloodstream Infections - United States. *MMWR Morbidity and mortality weekly report*. Mar 8 2019;68(9):214-219. doi:10.15585/mmwr.mm6809e1
246. David MZ, Daum RS, Bayer AS, et al. Staphylococcus aureus bacteremia at 5 US academic medical centers, 2008-2011: significant geographic variation in community-onset infections. *Clin Infect Dis*. Sep 15 2014;59(6):798-807. doi:10.1093/cid/ciu410
247. King CM, Castellucci-Garza FM, Lyon L, Doyle MD, Nimick C, Williams ML. Microorganisms Associated With Osteomyelitis of the Foot and Ankle. *J Foot Ankle Surg*. May - Jun 2020;59(3):491-494. doi:10.1053/j.jfas.2019.08.032
248. Dukic VM, Lauderdale DS, Wilder J, Daum RS, David MZ. Epidemics of community-associated methicillin-resistant Staphylococcus aureus in the United States: a meta-analysis. *PLoS One*. 2013;8(1):e52722. doi:10.1371/journal.pone.0052722
249. Park KH, Chong YP, Kim SH, et al. Clinical characteristics and therapeutic outcomes of hematogenous vertebral osteomyelitis caused by methicillin-resistant Staphylococcus aureus. *J Infect*. Dec 2013;67(6):556-64. doi:10.1016/j.jinf.2013.07.026
250. Kim JJ, Lydecker A, Dave R, Bork JT, Roghmann MC. Diabetic Foot Infections: Local Prevalence of and Case-Control Study of Risk Factors for Methicillin-Resistant Staphylococcus aureus and Pseudomonas aeruginosa. *Open forum infectious diseases*. Oct 2020;7(10):ofaa412. doi:10.1093/ofid/ofaa412
251. Ashong CN, Raheem SA, Hunter AS, Mindru C, Barshes NR. Methicillin-Resistant Staphylococcus aureus in Foot Osteomyelitis. *Surgical infections*. Feb/Mar 2017;18(2):143-148. doi:10.1089/sur.2016.165
252. Aragon-Sanchez J, Lazaro-Martinez JL, Quintana-Marrero Y, et al. Are diabetic foot ulcers complicated by MRSA osteomyelitis associated with worse prognosis? Outcomes of a surgical series. *Diabet Med*. May 2009;26(5):552-5. doi:10.1111/j.1464-5491.2009.02714.x
253. Farkas A, Lin F, Bui K, et al. Development of predictive nomograms for clinical use to quantify the risk of isolating resistance prone organisms in patients with infected foot ulcers. *Epidemiol Infect*. Jan 2019;147:e157. doi:10.1017/S0950268818003667
254. Bansal E, Garg A, Bhatia S, Attri AK, Chander J. Spectrum of microbial flora in diabetic foot ulcers. *Indian journal of pathology & microbiology*. Apr-Jun 2008;51(2):204-8. doi:10.4103/0377-4929.41685
255. Ramakant P, Verma AK, Misra R, et al. Changing microbiological profile of pathogenic bacteria in diabetic foot infections: time for a rethink on which empirical therapy to choose? *Diabetologia*. Jan 2011;54(1):58-64. doi:10.1007/s00125-010-1893-7
256. Raja NS. Microbiology of diabetic foot infections in a teaching hospital in Malaysia: a retrospective study of 194 cases. *Journal of microbiology, immunology, and infection = Wei mian yu gan ran za zhi*. Feb 2007;40(1):39-44.
257. Turhan V, Mutluoglu M, Acar A, et al. Increasing incidence of Gram-negative organisms in bacterial agents isolated from diabetic foot ulcers. *Journal of infection in developing countries*. Oct 15 2013;7(10):707-12. doi:10.3855/jidc.2967
258. Yun HC, Branstetter JG, Murray CK. Osteomyelitis in military personnel wounded in Iraq and Afghanistan. *J Trauma*. Feb 2008;64(2 Suppl):S163-8; discussion S168. doi:10.1097/TA.0b013e318160868c

© 2022 Spellberg B et al. *JAMA Network Open.*

259. Carvalho VC, Oliveira PR, Dal-Paz K, Paula AP, Felix Cda S, Lima AL. Gram-negative osteomyelitis: clinical and microbiological profile. *Braz J Infect Dis*. Jan-Feb 2012;16(1):63-7.
260. Roland PS, Stroman DW. Microbiology of acute otitis externa. *Laryngoscope*. Jul 2002;112(7 Pt 1):1166-77. doi:10.1097/00005537-200207000-00005
261. Ninkovic G, Dullo V, Saunders NC. Microbiology of otitis externa in the secondary care in United Kingdom and antimicrobial sensitivity. *Auris Nasus Larynx*. Dec 2008;35(4):480-4. doi:10.1016/j.anl.2007.09.013
262. Dibb WL. Microbial aetiology of otitis externa. *J Infect*. May 1991;22(3):233-9. doi:10.1016/s0163-4453(05)80004-0
263. Miller EH, Semian DW. Gram-negative osteomyelitis following puncture wounds of the foot. *J Bone Joint Surg Am*. Jun 1975;57(4):535-7.
264. Fitzgerald RH, Jr., Cowan JD. Puncture wounds of the foot. *Orthop Clin North Am*. Oct 1975;6(4):965-72.
265. Lavery LA, Harkless LB, Felder-Johnson K, Mundine S. Bacterial pathogens in infected puncture wounds in adults with diabetes. *J Foot Ankle Surg*. Jan-Feb 1994;33(1):91-7.
266. Ceriotti F, Marino I, Motta A, Carobene A. Analytical evaluation of the performances of Diazyme and BRAHMS procalcitonin applied to Roche Cobas in comparison with BRAHMS PCT-sensitive Kryptor. *Clin Chem Lab Med*. Nov 27 2017;56(1):162-169. doi:10.1515/cclm-2017-0159
267. Shah NB, Osmon DR, Steckelberg JM, et al. Pseudomonas Prosthetic Joint Infections: A Review of 102 Episodes. *J Bone Jt Infect*. 2016;1:25-30. doi:10.7150/jbji.15722
268. von Baum H, Bottcher S, Abel R, Gerner HJ, Sonntag HG. Tissue and serum concentrations of levofloxacin in orthopaedic patients. *Int J Antimicrob Agents*. Oct 2001;18(4):335-40.
269. Rimmele T, Boselli E, Breilh D, et al. Diffusion of levofloxacin into bone and synovial tissues. *J Antimicrob Chemother*. Mar 2004;53(3):533-5.
270. Metallidis S, Topsis D, Nikolaidis J, et al. Penetration of moxifloxacin and levofloxacin into cancellous and cortical bone in patients undergoing total hip arthroplasty. *J Chemother*. Dec 2007;19(6):682-7. doi:10.1179/joc.2007.19.6.682
271. Lozano-Alonso S, Linares-Palomino JP, Vera-Arroyo B, Bravo-Molina A, Hernandez-Quero J, Ros-Die E. [Evaluation of the tissue diffusion capacity of antibiotics in lower limb ischaemia]. *Enferm Infecc Microbiol Clin*. Oct 2016;34(8):477-83. Evaluacion de la capacidad de difusion tisular de antibioticos en isquemia de miembros inferiores. doi:10.1016/j.eimc.2015.10.012
272. Fong IW, Ledbetter WH, Vandenbroucke AC, Simbul M, Rahm V. Ciprofloxacin concentrations in bone and muscle after oral dosing. *Antimicrob Agents Chemother*. Mar 1986;29(3):405-8.
273. Massias L, Buffe P, Cohen B, et al. Study of the distribution of oral ciprofloxacin into the mucosa of the middle ear and the cortical bone of the mastoid process. *Chemotherapy*. 1994;40 Suppl 1:3-7. doi:10.1159/000239309
274. Leone M, Sampol-Manos E, Santelli D, et al. Brain tissue penetration of ciprofloxacin following a single intravenous dose. *J Antimicrob Chemother*. Oct 2002;50(4):607-9. doi:10.1093/jac/dkf179
275. Kitzes-Coehn R, Erde M, Sharvit E, al. e. Ciprofloxacin levels in malperfused tissue of patients with arterial occlusive disease [abstract]. *3rd International Symposium on New Quinolones ; Jul 12-14: Vancouver (BC)*. 1990;

© 2022 Spellberg B et al. *JAMA Network Open.*

276. Tolsdorff P. Tissue and serum concentrations of ofloxacin in the ear region following a single daily oral dose of 400 mg. *Infection*. Jan-Feb 1993;21(1):63-5. doi:10.1007/BF01739319
277. Tolsdorff P. Concentrations of ofloxacin in ear and nasal tissues. *Eur Arch Otorhinolaryngol*. 1993;250 Suppl 1:S7-11. doi:10.1007/BF02540109
278. Meissner A, Borner K, Koeppe P. Concentrations of ofloxacin in human bone and in cartilage. *J Antimicrob Chemother*. Nov 1990;26 Suppl D:69-74. doi:10.1093/jac/26.suppl_d.69
279. Landersdorfer C, Holzgrabe U, Kinzig-Schippers M, al e. Concept of internal standard used to standardize tissue level measurements and allow valid comparison between agents [abstract]. *44th Interscience Conference on Antimicrobial Agents and Chemotherapy 2004; Oct 30-Nov 2: Washington DC*. 2004;
280. Landersdorfer CB, Kinzig M, Hennig FF, et al. Penetration of moxifloxacin into bone evaluated by Monte Carlo simulation. *Antimicrob Agents Chemother*. May 2009;53(5):2074-81. doi:AAC.01056-08 [pii]

10.1128/AAC.01056-08
281. Malincarne L, Ghebregzabher M, Moretti MV, et al. Penetration of moxifloxacin into bone in patients undergoing total knee arthroplasty. *J Antimicrob Chemother*. May 2006;57(5):950-4.
282. Malizia T, Batoni G, Ghelardi E, et al. Interaction between piroxicam and azithromycin during distribution to human periodontal tissues. *J Periodontol*. Sep 2001;72(9):1151-6. doi:10.1902/jop.2000.72.9.1151
283. Malizia T, Tejada MR, Ghelardi E, et al. Periodontal tissue disposition of azithromycin. *J Periodontol*. Dec 1997;68(12):1206-9. doi:10.1902/jop.1997.68.12.1206
284. Bystedt H, A DA, Dornbusch K, Nord CE. Concentrations of azidocillin, erythromycin, doxycycline and clindamycin in human mandibular bone. *Int J Oral Surg*. Oct 1978;7(5):442-9.
285. Schurman DJ, Johnson BL, Jr., Finerman G, Amstutz HC. Antibiotic bone penetration. Concentrations of methicillin and clindamycin phosphate in human bone taken during total hip replacement. *Clin Orthop*. Sep 1975;(111):142-6.
286. Dornbusch K, Carlstrom A, Hugo H, Lidstrom A. Antibacterial activity of clindamycin and lincomycin in human bone. *J Antimicrob Chemother*. Mar 1977;3(2):153-60. doi:10.1093/jac/3.2.153
287. Nicholas P, Meyers BR, Levy RN, Hirschman SZ. Concentration of clindamycin in human bone. *Antimicrob Agents Chemother*. Aug 1975;8(2):220-1.
288. Roth B. Penetration of parenterally administered rifampicin into bone tissue. *Chemotherapy*. 1984;30(6):358-65.
289. Cluzel RA, Lopitaux R, Sirot J, Rampon S. Rifampicin in the treatment of osteoarticular infections due to staphylococci. *J Antimicrob Chemother*. Jun 1984;13 Suppl C:23-9. doi:10.1093/jac/13.suppl_c.23
290. Sirot J, Prive L, Lopitaux R, Glanddier Y. [Diffusion of rifampicin into spongy and compact bone tissue during total hip prosthesis operation]. *Pathol Biol (Paris)*. May 1983;31(5):438-41. Etude de la diffusion de la rifampicine dans le tissu osseux spongieux et compact au cours de protheses totales de hanche.

© 2022 Spellberg B et al. *JAMA Network Open.*

291. Sirot J, Lopitaux R, Cluzel R, Delisle JJ, Sauvezie B. [Rifampicin diffusion in non-infected human bone (author's transl)]. *Ann Microbiol (Paris)*. Aug-Sep 1977;128(2):229-36. Diffusion de la rifampicine dans l'os non infecte chez l'homme.
292. Rodvold KA, Gotfried MH, Cwik M, Korth-Bradley JM, Dukart G, Ellis-Grosse EJ. Serum, tissue and body fluid concentrations of tigecycline after a single 100 mg dose. *J Antimicrob Chemother*. Dec 2006;58(6):1221-9. doi:10.1093/jac/dkl403
293. MacGregor RR, Graziani AL. Oral administration of antibiotics: a rational alternative to the parenteral route. *Clin Infect Dis*. Mar 1997;24(3):457-67. doi:10.1093/clinids/24.3.457
294. Gnarpe H, Dornbusch K, Hagg O. Doxycycline concentration levels in bone, soft tissue and serum after intravenous infusion of doxycycline. A clinical study. *Scandinavian journal of infectious diseases Supplementum*. 1976;(9):54-7.
295. Vuorisalo S, Pokela R, Satta J, Syrjala H. Internal Mammary Artery Harvesting and Antibiotic Concentrations in Sternal Bone During Coronary Artery Bypass. *Int J Angiol*. Mar 2000;9(2):78-81. doi:10.1007/BF01617045
296. Kitzes-Cohen R, Farin D, Piva G, et al. Pharmacokinetics of vancomycin administered as prophylaxis before cardiac surgery. *Therapeutic drug monitoring*. Dec 2000;22(6):661-7. doi:10.1097/00007691-200012000-00004
297. Massias L, Dubois C, de Lentdecker P, Brodaty O, Fischler M, Farinotti R. Penetration of vancomycin in uninfected sternal bone. *Antimicrob Agents Chemother*. Nov 1992;36(11):2539-41.
298. Martin C, Alaya M, Mallet MN, et al. Penetration of vancomycin into mediastinal and cardiac tissues in humans. *Antimicrob Agents Chemother*. Feb 1994;38(2):396-9.
299. Borner K, Hahn F, Koeppe P. Concentrations of vancomycin in human bone. *J Chemother*. Jul 1989;1(4 Suppl):588-9.
300. Graziani AL, Lawson LA, Gibson GA, Steinberg MA, MacGregor RR. Vancomycin concentrations in infected and noninfected human bone. *Antimicrob Agents Chemother*. Sep 1988;32(9):1320-2.
301. Bue M, Tottrup M, Hanberg P, et al. Bone and subcutaneous adipose tissue pharmacokinetics of vancomycin in total knee replacement patients. *Acta Orthop*. Feb 2018;89(1):95-100. doi:10.1080/17453674.2017.1373497
302. Wilson AP, Taylor B, Treasure T, et al. Antibiotic prophylaxis in cardiac surgery: serum and tissue levels of teicoplanin, flucloxacillin and tobramycin. *J Antimicrob Chemother*. Feb 1988;21(2):201-12. doi:10.1093/jac/21.2.201
303. de Lalla F, Novelli A, Pellizzer G, et al. Regional and systemic prophylaxis with teicoplanin in monolateral and bilateral total knee replacement procedures: study of pharmacokinetics and tissue penetration. *Antimicrob Agents Chemother*. Dec 1993;37(12):2693-8. doi:10.1128/AAC.37.12.2693
304. Nehrer S, Thalhammer F, Schwameis E, Breyer S, Kotz R. Teicoplanin in the prevention of infection in total hip replacement. *Arch Orthop Trauma Surg*. 1998;118(1-2):32-6. doi:10.1007/s004020050306
305. Wilson AP. Clinical pharmacokinetics of teicoplanin. *Clin Pharmacokinet*. Sep 2000;39(3):167-83. doi:10.2165/00003088-200039030-00001
306. Montange D, Berthier F, Leclerc G, et al. Penetration of daptomycin into bone and synovial fluid in joint replacement. *Antimicrob Agents Chemother*. Jul 2014;58(7):3991-6. doi:10.1128/AAC.02344-14

307. Traunmuller F, Schintler MV, Metzler J, et al. Soft tissue and bone penetration abilities of daptomycin in diabetic patients with bacterial foot infections. *J Antimicrob Chemother*. Jun 2010;65(6):1252-7. doi:dkq109 [pii]

10.1093/jac/dkq109
308. Lovering AM, Zhang J, Bannister GC, et al. Penetration of linezolid into bone, fat, muscle and haematoma of patients undergoing routine hip replacement. *J Antimicrob Chemother*. Jul 2002;50(1):73-7.
309. Rana B, Butcher I, Grigoris P, Murnaghan C, Seaton RA, Tobin CM. Linezolid penetration into osteo-articular tissues. *J Antimicrob Chemother*. Nov 2002;50(5):747-50.
310. Kutscha-Lissberg F, Hebler U, Muhr G, Koller M. Linezolid penetration into bone and joint tissues infected with methicillin-resistant staphylococci. *Antimicrob Agents Chemother*. Dec 2003;47(12):3964-6.
311. Wen S, Zhang T, Yu X, et al. Bone penetration of linezolid in osteoarticular tuberculosis patients of China. *International journal of infectious diseases : IJID : official publication of the International Society for Infectious Diseases*. Feb 2021;103:364-369. doi:10.1016/j.ijid.2020.11.203
312. Li Y, Huang H, Dong W, et al. Penetration of linezolid into bone tissue 24 h after administration in patients with multidrug-resistant spinal tuberculosis. *PLoS One*. 2019;14(10):e0223391. doi:10.1371/journal.pone.0223391
313. Andreas M, Zeitlinger M, Wisser W, et al. Cefazolin and linezolid penetration into sternal cancellous bone during coronary artery bypass grafting. *Eur J Cardiothorac Surg*. Nov 2015;48(5):758-64. doi:10.1093/ejcts/ezu491
314. Dunne MW, Puttagunta S, Sprenger CR, Rubino C, Van Wart S, Baldassarre J. Extended duration dosing and distribution of dalbavancin into bone and articular tissue. *Antimicrob Agents Chemother*. Jan 5 2015;doi:10.1128/AAC.04550-14
315. Hierholzer G, Knothe H, Rehn J. [Penetration of fusidic acid into aseptic bone tissue]. *Arzneimittel-Forschung*. Oct 1970;20(10):1473-6. Penetration der Fusidinsaure in aspetisches Knochengewebe.
316. Hierholzer G, Knothe H, Rehn J, Koch F. [Fusidic acid concentrations in chronically inflamed tissue. Studies of chronic posttraumatic osteomyelitis]. *Arzneimittel-Forschung*. Nov 1966;16(11):1549-52. Fusidinsaure-Konzentrationen in chronisch entzundetem Gewebe. Untersuchungen zur chronischen posttraumatischen Osteomyelitis.
317. Chater EH, Flynn J, Wilson AL. Fucidin levels in osteomyelitis. *J Ir Med Assoc*. Oct 21 1972;65(20):506-8.
318. Sirot J, Lopitaux R, Dumont C, Rampon S, Cluzel R. [Diffusion of fosfomycin into bone tissue in man]. *Pathol Biol (Paris)*. Jun 1983;31(6):522-4. Diffusion de la fosfomycine dans le tissu osseux chez l'homme.
319. Plaue R, Muller O, Fabricius K, al. e. Untersuchugen uber die Diffusionsrate von Fosfomycin in verschiedene menschliche Gewebe. *Therapiewoche*. 1980;30:8329-33.
320. Wittmann DH. Chemotherapeutic Principles of Difficult-to-Treat Infections in Surgery: II. Bone and Joint Infections. *Infection*. 1980;8:330-3.
321. Saux MC, Le Rebeller A, Leng B, Mintrosse J. Bone diffusion of trimethoprim and sulfamethoxazole high pressure liquid chromatography (HPLC) (author's transl). *Pathol Biol (Paris)*. Jun 1982;30(6):385-8. Diffusion osseuse de l'association sulfamethoxazole-trimethoprime. Dosages effectues en chromatographie liquide a haute performance (CLHP).

322. Akimoto Y, Kaneko K, Tamura T. Amoxicillin concentrations in serum, jaw cyst, and jawbone following a single oral administration. *J Oral Maxillofac Surg*. May 1982;40(5):287-93. doi:10.1016/0278-2391(82)90220-8
323. Grimer RJ, Karpinski MR, Andrews JM, Wise R. Penetration of amoxycillin and clavulanic acid into bone. *Chemotherapy*. 1986;32(3):185-91. doi:10.1159/000238414
324. Weismeier K, Adam D, Heilmann HD, Koeppe P. Penetration of amoxycillin/clavulanate into human bone. *J Antimicrob Chemother*. Nov 1989;24 Suppl B:93-100. doi:10.1093/jac/24.suppl_b.93
325. Adam D, Heilmann HD, Weismeier K. Concentrations of ticarcillin and clavulanic acid in human bone after prophylactic administration of 5.2 g of timentin. *Antimicrob Agents Chemother*. Jun 1987;31(6):935-9. doi:10.1128/AAC.31.6.935
326. Warnke JP, Wildfeuer A, Eibel G, Pfaff G, Klammer A. Pharmacokinetics of ampicillin/sulbactam in patients undergoing spinal microneurosurgical procedures. *Int J Clin Pharmacol Ther*. May 1998;36(5):253-7.
327. Wildfeuer A, Mallwitz J, Gotthardt H, et al. Pharmacokinetics of ampicillin, sulbactam and cefotiam in patients undergoing orthopedic surgery. *Infection*. Jul-Aug 1997;25(4):258-62.
328. Dehne MG, Muhling J, Sablotzki A, Nopens H, Hempelmann G. Pharmacokinetics of antibiotic prophylaxis in major orthopedic surgery and blood-saving techniques. *Orthopedics*. Jul 2001;24(7):665-9.
329. Incavo SJ, Ronchetti PJ, Choi JH, Wu H, Kinzig M, Sorgel F. Penetration of piperacillin-tazobactam into cancellous and cortical bone tissues. *Antimicrob Agents Chemother*. Apr 1994;38(4):905-7.
330. Boselli E, Breilh D, Debon R, et al. Penetration of piperacillin/tazobactam (4 g/500 mg) into synovial tissue. *J Chemother*. Feb 2002;14(1):54-8. doi:10.1179/joc.2002.14.1.54
331. Al-Nawas B, Kinzig-Schippers M, Soergel F, Shah PM. Concentrations of piperacillin-tazobactam in human jaw and hip bone. *J Craniomaxillofac Surg*. Dec 2008;36(8):468-72. doi:10.1016/j.jcms.2008.06.003
332. Unsworth PF, Heatley FW, Phillips I. Flucloxacillin in bone. *J Clin Pathol*. Aug 1978;31(8):705-11. doi:10.1136/jcp.31.8.705
333. Kondell PA, Nord CE, Nordenram A. Concentrations of cloxacillin, dicloxacillin and flucloxacillin in dental alveolar serum and mandibular bone. *Int J Oral Surg*. Feb 1982;11(1):40-3. doi:10.1016/s0300-9785(82)80047-1
334. Torkington MS, Davison MJ, Wheelwright EF, et al. Bone penetration of intravenous flucloxacillin and gentamicin as antibiotic prophylaxis during total hip and knee arthroplasty. *Bone Joint J*. Mar 2017;99-B(3):358-364. doi:10.1302/0301-620X.99B3.BJJ-2016-0328.R1
335. Sirot J, Lopitaux R, Dumont C, Delisle JJ, Rampon S, Cluzel R. [Penetration of cloxacillin into human bone tissue after oral administration (author's transl)]. *Pathol Biol (Paris)*. Jun 1982;30(6):332-5. Diffusion de la cloxacilline dans le tissu osseux humain apres administration par voie orale.
336. Fitzgerald RH, Jr., Kelly PJ, Snyder RJ, Washington JA, 2nd. Penetration of methicillin, oxacillin, and cephalothin into bone and synovial tissues. *Antimicrob Agents Chemother*. Nov 1978;14(5):723-6. doi:10.1128/AAC.14.5.723
337. Sorensen TS, Colding H, Schroeder E, Rosdahl VT. The penetration of cefazolin, erythromycin and methicillin into human bone tissue. *Acta Orthop Scand*. Dec 1978;49(6):549-53. doi:10.3109/17453677808993236

© 2022 Spellberg B et al. *JAMA Network Open.*

338. Boselli E, Breilh D, Djabarouti S, Bel JC, Saux MC, Allaouchiche B. Diffusion of ertapenem into bone and synovial tissues. *J Antimicrob Chemother*. Oct 2007;60(4):893-6. doi:10.1093/jac/dkm296
339. Polk R, Hume A, Kline BJ, Cardea J. Penetration of moxalactam and cefazolin into bone following simultaneous bolus or infusion. *Clin Orthop*. Jul-Aug 1983;(177):216-21.
340. Yamada K, Matsumoto K, Tokimura F, Okazaki H, Tanaka S. Are bone and serum cefazolin concentrations adequate for antimicrobial prophylaxis? *Clin Orthop Relat Res*. Dec 2011;469(12):3486-94. doi:10.1007/s11999-011-2111-8
341. Cunha BA, Gossling HR, Pasternak HS, Nightingale CH, Quintiliani R. Penetration of cephalosporins into bone. *Infection*. Mar-Apr 1984;12(2):80-4.
342. Williams DN, Gustilo RB, Beverly R, Kind AC. Bone and serum concentrations of five cephalosporin drugs. Relevance to prophylaxis and treatment in orthopedic surgery. *Clin Orthop*. Oct 1983;(179):253-65.
343. Akimoto Y, Uda A, Omata H, et al. Cephalexin concentrations in human serum, gingiva, and mandibular bone following a single oral administration. *Gen Pharmacol*. 1990;21(5):621-3. doi:10.1016/0306-3623(90)91007-e
344. Renneberg J, Christensen OM, Thomsen NO, Torholm C. Cefuroxime concentrations in serum, joint fluid and bone in elderly patients undergoing arthroplasty after administration of cefuroxime axetil. *J Antimicrob Chemother*. Nov 1993;32(5):751-5. doi:10.1093/jac/32.5.751
345. Leigh DA, Marriner J, Nisbet D, Powell HD, Church JC, Wise K. Bone concentrations of cefuroxime and cefamandole in the femoral head in 96 patients undergoing total hip replacement surgery. *J Antimicrob Chemother*. Apr 1982;9(4):303-11. doi:10.1093/jac/9.4.303
346. Cain TJ, Jones GT, Woods P. Bone levels of cephradine and cefuroxime after intravenous administration in patients undergoing total hip replacement. *Int Orthop*. 1987;11(1):61-3. doi:10.1007/BF00266059
347. Lovering AM, Perez J, Bowker KE, Reeves DS, MacGowan AP, Bannister G. A comparison of the penetration of cefuroxime and cephamandole into bone, fat and haematoma fluid in patients undergoing total hip replacement. *J Antimicrob Chemother*. Jul 1997;40(1):99-104. doi:10.1093/jac/40.1.99
348. Ketterl R, Wittwer W. [Possibilities for the use of the basic cephalosporin cefuroxime in bone surgery. Tissue levels, effectiveness and tolerance]. *Infection*. 1993;21 Suppl 1:S21-7. Einsatzmoglichkeiten des Basiscephalosporins Cefuroxim in der Knochenchirurgie. Gewebespiegel, Wirksamkeit und Vertraglichkeit. doi:10.1007/BF01710340
349. Quintiliani R. A review of the penetration of cefadroxil into human tissue. *J Antimicrob Chemother*. Sep 1982;10 Suppl B:33-8. doi:10.1093/jac/10.suppl_b.33
350. Braga PC, Pozzato C, Cedrini C, et al. Clinical pharmacokinetics of cefotaxime and penetration in sputum, bone, and prostatic tissue. *Clin Ther*. 1982;5(1):69-78.
351. Lovering AM, Walsh TR, Bannister GC, MacGowan AP. The penetration of ceftriaxone and cefamandole into bone, fat and haematoma and relevance of serum protein binding to their penetration into bone. *J Antimicrob Chemother*. Apr 2001;47(4):483-6.
352. Scaglione F, De Martini G, Peretto L, et al. Pharmacokinetic study of cefodizime and ceftriaxone in sera and bones of patients undergoing hip arthroplasty. *Antimicrob Agents Chemother*. Oct 1997;41(10):2292-4.

© 2022 Spellberg B et al. *JAMA Network Open.*

353. Soudry B, Sirot J, Lopitaux R, Dumont C, Delisle JJ, Parenton M. Diffusion of ceftriaxone in human bone tissue. *Pathol Biol (Paris)*. Sep 1986;34(7):859-62. Diffusion de la ceftriaxone dans le tissu osseux humain.
354. Garazzino S, Aprato A, Baietto L, et al. Ceftriaxone bone penetration in patients with septic non-union of the tibia. *International journal of infectious diseases : IJID : official publication of the International Society for Infectious Diseases*. Jun 2011;15(6):e415-21. doi:10.1016/j.ijid.2011.03.003
355. Adam D, Reichart B, Williams KJ. Penetration of ceftazidime into human tissue in patients undergoing cardiac surgery. *J Antimicrob Chemother*. Jul 1983;12 Suppl A:269-73. doi:10.1093/jac/12.suppl_a.269
356. Raymakers JT, Schaper NC, van der Heyden JJ, Tordoir JH, Kitslaar PJ. Penetration of ceftazidime into bone from severely ischaemic limbs. *J Antimicrob Chemother*. Oct 1998;42(4):543-5.
357. Raymakers JT, Houben AJ, van der Heyden JJ, Tordoir JH, Kitslaar PJ, Schaper NC. The effect of diabetes and severe ischaemia on the penetration of ceftazidime into tissues of the limb. *Diabet Med*. Mar 2001;18(3):229-34.
358. Breilh D, Boselli E, Bel JC, Chassard D, Saux MC, Allaouchiche B. Diffusion of cefepime into cancellous and cortical bone tissue. *J Chemother*. Apr 2003;15(2):134-8.
359. Boselli E, Allaouchiche B. [Diffusion in bone tissue of antibiotics]. *Presse Med*. Dec 18-25 1999;28(40):2265-76. Diffusion osseuse des antibiotiques.
360. Drusano GL. Antimicrobial pharmacodynamics: critical interactions of 'bug and drug'. *Nat Rev Microbiol*. Apr 2004;2(4):289-300. doi:10.1038/nrmicro862
361. Mouton JW, Theuretzbacher U, Craig WA, Tulkens PM, Derendorf H, Cars O. Tissue concentrations: do we ever learn? *J Antimicrob Chemother*. Feb 2008;61(2):235-7. doi:10.1093/jac/dkm476
362. Ahmed S, Meghji S, Williams RJ, Henderson B, Brock JH, Nair SP. Staphylococcus aureus fibronectin binding proteins are essential for internalization by osteoblasts but do not account for differences in intracellular levels of bacteria. *Infection and immunity*. May 2001;69(5):2872-7. doi:10.1128/IAI.69.5.2872-2877.2001
363. Thabit AK, Fatani DF, Bamakhrama MS, Barnawi OA, Basudan LO, Alhejaili SF. Antibiotic penetration into bone and joints: An updated review. *International journal of infectious diseases : IJID : official publication of the International Society for Infectious Diseases*. Apr 2019;81:128-136. doi:10.1016/j.ijid.2019.02.005
364. Landersdorfer CB, Bulitta JB, Kinzig M, Holzgrabe U, Sorgel F. Penetration of antibacterials into bone: pharmacokinetic, pharmacodynamic and bioanalytical considerations. *Clin Pharmacokinet*. 2009;48(2):89-124. doi:2 [pii]
365. Alvarez Ferrero MM, Vree TB, Baars AM, Slooff TJ. Plasma and bone concentrations of cefuroxime and flucloxacillin. Oral versus parenteral administration in 20 arthroplasties. *Acta Orthop Scand*. Oct 1993;64(5):525-9. doi:10.3109/17453679308993684
366. Lauf L, Ozsvar Z, Mitha I, et al. Phase 3 study comparing tigecycline and ertapenem in patients with diabetic foot infections with and without osteomyelitis. *Diagn Microbiol Infect Dis*. Apr 2014;78(4):469-80. doi:10.1016/j.diagmicrobio.2013.12.007
367. Jalava S, Saarimaa H, Elfving R. Cephalexin levels in serum, synovial fluid and joint tissues after oral administration. *Scand J Rheumatol*. 1977;6(4):250-2. doi:10.3109/03009747709095460

© 2022 Spellberg B et al. *JAMA Network Open.*

368. Hughes SP, Nixon J, Dash CH. Cephalexin in chronic osteomyelitis. *J R Coll Surg Edinb*. Nov 1981;26(6):335-9.
369. Hernandez B, Bravo PA, Zarraga J, et al. [Cephalexin monohydrate in the treatment of bone and soft tissue infections]. *Prensa Med Mex*. Sep-Oct 1970:32-5. Monohidrato de cefalexina en el tratamiento de infecciones oseas y de partes blandas.
370. Endler M, Hackel H. [Oral cephalexin therapy of osteomyelitis (author's transl)]. *Wien Med Wochenschr*. Feb 28 1979;129(4):107-11. Orale Antibiotikatherapie der Osteomyelitis mit Cephalexin.
371. Evrard J. [Cephalexin in the treatment of bone and joint infections]. *Nouv Presse Med*. May 19 1973;2(20):1349-50. La cefalexine dans le traitement des infections osteo-articulaires.
372. Van der Auwera P, Klastersky J, Thys JP, Meunier-Carpentier F, Legrand JC. Double-blind, placebo-controlled study of oxacillin combined with rifampin in the treatment of staphylococcal infections. *Antimicrob Agents Chemother*. Oct 1985;28(4):467-72.
373. Norden CW, Bryant R, Palmer D, Montgomerie JZ, Wheat J. Chronic osteomyelitis caused by Staphylococcus aureus: controlled clinical trial of nafcillin therapy and nafcillin-rifampin therapy. *South Med J*. Aug 1986;79(8):947-51.
374. Zimmerli W, Widmer AF, Blatter M, Frei R, Ochsner PE. Role of rifampin for treatment of orthopedic implant-related staphylococcal infections: a randomized controlled trial. Foreign-Body Infection (FBI) Study Group. *JAMA : the journal of the American Medical Association*. May 20 1998;279(19):1537-41.
375. Karlsen OE, Borgen P, Bragnes B, et al. Rifampin combination therapy in staphylococcal prosthetic joint infections: a randomized controlled trial. *J Orthop Surg Res*. Aug 28 2020;15(1):365. doi:10.1186/s13018-020-01877-2
376. Norden CW. Lessons learned from animal models of osteomyelitis. *Reviews of infectious diseases*. Jan-Feb 1988;10(1):103-10.
377. Waldvogel FA, Papageorgiou PS. Osteomyelitis: the past decade. *N Engl J Med*. Aug 14 1980;303(7):360-70. doi:10.1056/NEJM198008143030703
378. Haidar R, Der Boghossian A, Atiyeh B. Duration of post-surgical antibiotics in chronic osteomyelitis: empiric or evidence-based? *International journal of infectious diseases : IJID : official publication of the International Society for Infectious Diseases*. Sep 2010;14(9):e752-8. doi:S1201-9712(10)02331-3 [pii]

10.1016/j.ijid.2010.01.005
379. McHenry MC, Easley KA, Locker GA. Vertebral osteomyelitis: long-term outcome for 253 patients from 7 Cleveland-area hospitals. *Clin Infect Dis*. May 15 2002;34(10):1342-50. doi:10.1086/340102
380. Wang X, Fang L, Wang S, et al. Antibiotic treatment regimens for bone infection after debridement: a study of 902 cases. *BMC Musculoskelet Disord*. Apr 7 2020;21(1):215. doi:10.1186/s12891-020-03214-4
381. Tice AD, Hoaglund PA, Shoultz DA. Risk factors and treatment outcomes in osteomyelitis. *J Antimicrob Chemother*. May 2003;51(5):1261-8.
382. Tice AD, Hoaglund PA, Shoultz DA. Outcomes of osteomyelitis among patients treated with outpatient parenteral antimicrobial therapy. *Am J Med*. Jun 15 2003;114(9):723-8.
383. Dombrowski JC, Winston LG. Clinical failures of appropriately-treated methicillin-resistant Staphylococcus aureus infections. *J Infect*. Aug 2008;57(2):110-5. doi:S0163-4453(08)00150-3 [pii]

© 2022 Spellberg B et al. *JAMA Network Open.*

10.1016/j.jinf.2008.04.003
384. Liang SY, Khair HN, McDonald JR, Babcock HM, Marschall J. Daptomycin versus vancomycin for osteoarticular infections due to methicillin-resistant Staphylococcus aureus (MRSA): a nested case-control study. *Eur J Clin Microbiol Infect Dis*. Apr 2014;33(4):659-64. doi:10.1007/s10096-013-2001-y
385. MacGregor RR, Gentry LO. Imipenem/cilastatin in the treatment of osteomyelitis. *Am J Med*. Jun 7 1985;78(6A):100-3.
386. Bach MC, Cocchetto DM. Ceftazidime as single-agent therapy for gram-negative aerobic bacillary osteomyelitis. *Antimicrob Agents Chemother*. Oct 1987;31(10):1605-8.
387. Conrad DA, Williams RR, Couchman TL, Lentnek AL. Efficacy of aztreonam in the treatment of skeletal infections due to Pseudomonas aeruginosa. *Reviews of infectious diseases*. May-Jun 1991;13 Suppl 7:S634-9.
388. Ciampolini J, Harding KG. Pathophysiology of chronic bacterial osteomyelitis. Why do antibiotics fail so often? *Postgraduate medical journal*. Aug 2000;76(898):479-83.
389. Gallie WE. First recurrence of osteomyelitis eighty years after infection. *J Bone Joint Surg Br*. Feb 1951;33-B(1):110-1.
390. Donati L, Quadri P, Reiner M. Reactivation of osteomyelitis caused by Staphylococcus aureus after 50 years. *J Am Geriatr Soc*. Aug 1999;47(8):1035-7.
391. Al-Maiyah M, Hemmady MV, Shoaib A, Morgan-Jones RL. Recurrence of chronic osteomyelitis in a regenerated fibula after 65 years. *Orthopedics*. May 2007;30(5):403-4.
392. Libraty DH, Patkar C, Torres B. Staphylococcus aureus reactivation osteomyelitis after 75 years. *N Engl J Med*. Feb 2 2012;366(5):481-2. doi:10.1056/NEJMc1111493
393. Clerc A, Zeller V, Marmor S, et al. Hematogenous osteomyelitis in childhood can relapse many years later into adulthood: A retrospective multicentric cohort study in France. *Medicine (Baltimore)*. May 2020;99(20):e19617. doi:10.1097/MD.0000000000019617
394. Korovessis P, Fortis AP, Spastris P, Droutsas P. Acute osteomyelitis of the patella 50 years after a knee fusion for septic arthritis. A case report. *Clin Orthop Relat Res*. Nov 1991;(272):205-7.
395. Gollan B, Grabe G, Michaux C, Helaine S. Bacterial Persisters and Infection: Past, Present, and Progressing. *Annu Rev Microbiol*. Sep 8 2019;73:359-385. doi:10.1146/annurev-micro-020518-115650
396. Tuchscherr L, Kreis CA, Hoerr V, et al. Staphylococcus aureus develops increased resistance to antibiotics by forming dynamic small colony variants during chronic osteomyelitis. *J Antimicrob Chemother*. Feb 2016;71(2):438-48. doi:10.1093/jac/dkv371
397. Kahl BC. Small colony variants (SCVs) of Staphylococcus aureus--a bacterial survival strategy. *Infect Genet Evol*. Jan 2014;21:515-22. doi:10.1016/j.meegid.2013.05.016
398. Kahl BC, Becker K, Loffler B. Clinical Significance and Pathogenesis of Staphylococcal Small Colony Variants in Persistent Infections. *Clin Microbiol Rev*. Apr 2016;29(2):401-27. doi:10.1128/CMR.00069-15
399. Gimza BD, Cassat JE. Mechanisms of Antibiotic Failure During Staphylococcus aureus Osteomyelitis. *Front Immunol*. 2021;12:638085. doi:10.3389/fimmu.2021.638085
400. Proctor RA, van Langevelde P, Kristjansson M, Maslow JN, Arbeit RD. Persistent and relapsing infections associated with small-colony variants of Staphylococcus aureus. *Clin Infect Dis*. Jan 1995;20(1):95-102. doi:10.1093/clinids/20.1.95

401. Tuchscherr L, Geraci J, Loffler B. Staphylococcus aureus Regulator Sigma B is Important to Develop Chronic Infections in Hematogenous Murine Osteomyelitis Model. *Pathogens*. Jul 15 2017;6(3)doi:10.3390/pathogens6030031
402. Yang D, Wijenayaka AR, Solomon LB, et al. Novel Insights into Staphylococcus aureus Deep Bone Infections: the Involvement of Osteocytes. *mBio*. Apr 24 2018;9(2)doi:10.1128/mBio.00415-18
403. Drusano GL, Sgambati N, Eichas A, Brown DL, Kulawy R, Louie A. The combination of rifampin plus moxifloxacin is synergistic for suppression of resistance but antagonistic for cell kill of Mycobacterium tuberculosis as determined in a hollow-fiber infection model. *mBio*. 2010;1(3)doi:10.1128/mBio.00139-10
404. Lechner S, Patra P, Klumpp S, Bertram R. Interplay between population dynamics and drug tolerance of Staphylococcus aureus persister cells. *J Mol Microbiol Biotechnol*. 2012;22(6):381-91. doi:10.1159/000346073
405. Alexander C, Guru A, Pradhan P, et al. MazEF-rifampicin interaction suggests a mechanism for rifampicin induced inhibition of persisters. *BMC Mol Cell Biol*. Oct 27 2020;21(1):73. doi:10.1186/s12860-020-00316-8
406. Conlon BP, Nakayasu ES, Fleck LE, et al. Activated ClpP kills persisters and eradicates a chronic biofilm infection. *Nature*. Nov 21 2013;503(7476):365-70. doi:10.1038/nature12790
407. Keren I, Mulcahy LR, Lewis K. Persister eradication: lessons from the world of natural products. *Methods in enzymology*. 2012;517:387-406. doi:10.1016/B978-0-12-404634-4.00019-X
408. O'Reilly T, Kunz S, Sande E, Zak O, Sande MA, Tauber MG. Relationship between antibiotic concentration in bone and efficacy of treatment of staphylococcal osteomyelitis in rats: azithromycin compared with clindamycin and rifampin. *Antimicrob Agents Chemother*. Dec 1992;36(12):2693-7.
409. Norden CW, Shaffer M. Treatment of experimental chronic osteomyelitis due to staphylococcus aureus with vancomycin and rifampin. *J Infect Dis*. Feb 1983;147(2):352-7.
410. Norden CW, Fierer J, Bryant RE. Chronic staphylococcal osteomyelitis: treatment with regimens containing rifampin. *Reviews of infectious diseases*. Jul-Aug 1983;5 Suppl 3:S495-501.
411. Dworkin R, Modin G, Kunz S, Rich R, Zak O, Sande M. Comparative efficacies of ciprofloxacin, pefloxacin, and vancomycin in combination with rifampin in a rat model of methicillin-resistant Staphylococcus aureus chronic osteomyelitis. *Antimicrob Agents Chemother*. Jun 1990;34(6):1014-6.
412. Jorgensen NP, Skovdal SM, Meyer RL, Dagnaes-Hansen F, Fuursted K, Petersen E. Rifampicin-containing combinations are superior to combinations of vancomycin, linezolid and daptomycin against Staphylococcus aureus biofilm infection in vivo and in vitro. *Pathog Dis*. Jun 2016;74(4):ftw019. doi:10.1093/femspd/ftw019
413. Albac S, Labrousse D, Hayez D, et al. Activity of Different Antistaphylococcal Therapies, Alone or Combined, in a Rat Model of Methicillin-Resistant Staphylococcus epidermidis Osteitis without Implant. *Antimicrob Agents Chemother*. Jan 27 2020;64(2)doi:10.1128/AAC.01865-19
414. Livorsi DJ, Daver NG, Atmar RL, Shelburne SA, White AC, Jr., Musher DM. Outcomes of treatment for hematogenous Staphylococcus aureus vertebral osteomyelitis in the MRSA ERA. *J Infect*. Aug 2008;57(2):128-31. doi:S0163-4453(08)00163-1 [pii]

© 2022 Spellberg B et al. *JAMA Network Open.*

10.1016/j.jinf.2008.04.012
415. Wilson BM, Bessesen MT, Doros G, et al. Adjunctive Rifampin Therapy For Diabetic Foot Osteomyelitis in the Veterans Health Administration. *JAMA Netw Open*. Nov 1 2019;2(11):e1916003. doi:10.1001/jamanetworkopen.2019.16003
416. El Helou OC, Berbari EF, Lahr BD, et al. Efficacy and safety of rifampin containing regimen for staphylococcal prosthetic joint infections treated with debridement and retention. *Eur J Clin Microbiol Infect Dis*. Aug 2010;29(8):961-7. doi:10.1007/s10096-010-0952-9
417. Senneville E, Joulie D, Legout L, et al. Outcome and predictors of treatment failure in total hip/knee prosthetic joint infections due to Staphylococcus aureus. *Clin Infect Dis*. Aug 2011;53(4):334-40. doi:cir402 [pii]

10.1093/cid/cir402
418. Lora-Tamayo J, Murillo O, Iribarren JA, et al. A large multicenter study of methicillin-susceptible and methicillin-resistant Staphylococcus aureus prosthetic joint infections managed with implant retention. *Clin Infect Dis*. Jan 2013;56(2):182-94. doi:10.1093/cid/cis746
419. Lora-Tamayo J, Senneville E, Ribera A, et al. The Not-So-Good Prognosis of Streptococcal Periprosthetic Joint Infection Managed by Implant Retention: The Results of a Large Multicenter Study. *Clin Infect Dis*. Jun 15 2017;64(12):1742-1752. doi:10.1093/cid/cix227
420. Becker A, Kreitmann L, Triffaut-Fillit C, et al. Duration of rifampin therapy is a key determinant of improved outcomes in early-onset acute prosthetic joint infection due to Staphylococcus treated with a debridement, antibiotics and implant retention (DAIR): a retrospective multicenter study in France. *J Bone Jt Infect*. 2020;5(1):28-34. doi:10.7150/jbji.40333
421. Fiaux E, Titecat M, Robineau O, et al. Outcome of patients with streptococcal prosthetic joint infections with special reference to rifampicin combinations. *BMC Infect Dis*. Oct 13 2016;16(1):568. doi:10.1186/s12879-016-1889-0
422. Lesens O, Ferry T, Forestier E, et al. Should we expand the indications for the DAIR (debridement, antibiotic therapy, and implant retention) procedure for Staphylococcus aureus prosthetic joint infections? A multicenter retrospective study. *Eur J Clin Microbiol Infect Dis*. Oct 2018;37(10):1949-1956. doi:10.1007/s10096-018-3330-7
423. Munoz-Gallego I, Viedma E, Esteban J, et al. Genotypic and Phenotypic Characteristics of Staphylococcus aureus Prosthetic Joint Infections: Insight on the Pathogenesis and Prognosis of a Multicenter Prospective Cohort. *Open Forum Infect Dis*. Sep 2020;7(9):ofaa344. doi:10.1093/ofid/ofaa344
424. Ascione T, Pagliano P, Mariconda M, et al. Factors related to outcome of early and delayed prosthetic joint infections. *J Infect*. Jul 28 2014;doi:10.1016/j.jinf.2014.07.008
425. Achermann Y, Kusejko K, Aunon A, et al. The impact of surgical strategy and rifampin on treatment outcome in Cutibacterium periprosthetic joint infections. *Clin Infect Dis*. Dec 10 2020;doi:10.1093/cid/ciaa1839
426. Akgun D, Trampuz A, Perka C, Renz N. High failure rates in treatment of streptococcal periprosthetic joint infection: results from a seven-year retrospective cohort study. *Bone Joint J*. May 2017;99-B(5):653-659. doi:10.1302/0301-620X.99B5.BJJ-2016-0851.R1
427. Mahieu R, Dubee V, Seegers V, et al. The prognosis of streptococcal prosthetic bone and joint infections depends on surgical management-A multicenter retrospective study. *Int J Infect Dis*. Aug 2019;85:175-181. doi:10.1016/j.ijid.2019.06.012

© 2022 Spellberg B et al. *JAMA Network Open.*

428. Morata L, Senneville E, Bernard L, et al. A Retrospective Review of the Clinical Experience of Linezolid with or Without Rifampicin in Prosthetic Joint Infections Treated with Debridement and Implant Retention. *Infect Dis Ther*. Dec 2014;3(2):235-43. doi:10.1007/s40121-014-0032-z
429. Vilchez HH, Escudero-Sanchez R, Fernandez-Sampedro M, et al. Prosthetic Shoulder Joint Infection by Cutibacterium acnes: Does Rifampin Improve Prognosis? A Retrospective, Multicenter, Observational Study. *Antibiotics (Basel)*. Apr 21 2021;10(5)doi:10.3390/antibiotics10050475
430. Davis JR, Nsengiyumva A, Igiraneza D, et al. Predictors of Survival: A Retrospective Review of Gastroschisis and Intestinal Atresia in Rwanda. *The Journal of surgical research*. Jan 24 2022;273:138-146. doi:10.1016/j.jss.2021.12.035
431. Aydin O, Ergen P, Ozturan B, Ozkan K, Arslan F, Vahaboglu H. Rifampin-accompanied antibiotic regimens in the treatment of prosthetic joint infections: a frequentist and Bayesian meta-analysis of current evidence. *Eur J Clin Microbiol Infect Dis*. Apr 2021;40(4):665-671. doi:10.1007/s10096-020-04083-4
432. Scheper H, Gerritsen LM, Pijls BG, Van Asten SA, Visser LG, De Boer MGJ. Outcome of Debridement, Antibiotics, and Implant Retention for Staphylococcal Hip and Knee Prosthetic Joint Infections, Focused on Rifampicin Use: A Systematic Review and Meta-Analysis. *Open forum infectious diseases*. Jul 2021;8(7):ofab298. doi:10.1093/ofid/ofab298
433. Boeree MJ, Heinrich N, Aarnoutse R, et al. High-dose rifampicin, moxifloxacin, and SQ109 for treating tuberculosis: a multi-arm, multi-stage randomised controlled trial. *Lancet Infect Dis*. Jan 2017;17(1):39-49. doi:10.1016/S1473-3099(16)30274-2
434. Velasquez GE, Brooks MB, Coit JM, et al. Efficacy and Safety of High-Dose Rifampin in Pulmonary Tuberculosis. A Randomized Controlled Trial. *Am J Respir Crit Care Med*. Sep 1 2018;198(5):657-666. doi:10.1164/rccm.201712-2524OC
435. Aarnoutse RE, Kibiki GS, Reither K, et al. Pharmacokinetics, Tolerability, and Bacteriological Response of Rifampin Administered at 600, 900, and 1,200 Milligrams Daily in Patients with Pulmonary Tuberculosis. *Antimicrob Agents Chemother*. Nov 2017;61(11)doi:10.1128/AAC.01054-17
436. Tonnelier M, Bouras A, Joseph C, et al. Impact of rifampicin dose in bone and joint prosthetic device infections due to Staphylococcus spp: a retrospective single-center study in France. *BMC Infect Dis*. Feb 12 2021;21(1):174. doi:10.1186/s12879-021-05832-2
437. Gandelman K, Zhu T, Fahmi OA, et al. Unexpected effect of rifampin on the pharmacokinetics of linezolid: in silico and in vitro approaches to explain its mechanism. *J Clin Pharmacol*. Feb 2011;51(2):229-36. doi:10.1177/0091270010366445
438. Okazaki F, Tsuji Y, Seto Y, Ogami C, Yamamoto Y, To H. Effects of a rifampicin pre-treatment on linezolid pharmacokinetics. *PLoS One*. 2019;14(9):e0214037. doi:10.1371/journal.pone.0214037
439. Tornero E, Morata L, Martinez-Pastor JC, et al. Importance of selection and duration of antibiotic regimen in prosthetic joint infections treated with debridement and implant retention. *J Antimicrob Chemother*. May 2016;71(5):1395-401. doi:10.1093/jac/dkv481
440. Curis E, Pestre V, Jullien V, et al. Pharmacokinetic variability of clindamycin and influence of rifampicin on clindamycin concentration in patients with bone and joint infections. *Infection*. Aug 2015;43(4):473-81. doi:10.1007/s15010-015-0773-y

© 2022 Spellberg B et al. *JAMA Network Open.*

441. Zeller V, Magreault S, Heym B, et al. Influence of the clindamycin administration route on the magnitude of clindamycin-rifampicin interaction: a prospective pharmacokinetic study. *Clin Microbiol Infect*. Apr 26 2021;doi:10.1016/j.cmi.2021.04.017
442. Bernard A, Kermarrec G, Parize P, et al. Dramatic reduction of clindamycin serum concentration in staphylococcal osteoarticular infection patients treated with the oral clindamycin-rifampicin combination. *J Infect*. Aug 2015;71(2):200-6. doi:10.1016/j.jinf.2015.03.013
443. Pushkin R, Iglesias-Ussel MD, Keedy K, et al. A Randomized Study Evaluating Oral Fusidic Acid (CEM-102) in Combination With Oral Rifampin Compared With Standard-of-Care Antibiotics for Treatment of Prosthetic Joint Infections: A Newly Identified Drug-Drug Interaction. *Clin Infect Dis*. Dec 15 2016;63(12):1599-1604. doi:10.1093/cid/ciw665
444. Aboltins CA, Page MA, Buising KL, et al. Treatment of staphylococcal prosthetic joint infections with debridement, prosthesis retention and oral rifampicin and fusidic acid. *Clin Microbiol Infect*. Jun 2007;13(6):586-91. doi:10.1111/j.1469-0691.2007.01691.x
445. Peel TN, Buising KL, Dowsey MM, et al. Outcome of debridement and retention in prosthetic joint infections by methicillin-resistant staphylococci, with special reference to rifampin and fusidic acid combination therapy. *Antimicrob Agents Chemother*. Jan 2013;57(1):350-5. doi:10.1128/AAC.02061-12
446. Ribera E, Pou L, Fernandez-Sola A, et al. Rifampin reduces concentrations of trimethoprim and sulfamethoxazole in serum in human immunodeficiency virus-infected patients. *Antimicrob Agents Chemother*. Nov 2001;45(11):3238-41. doi:10.1128/AAC.45.11.3238-3241.2001
447. Harbarth S, von Dach E, Pagani L, et al. Randomized non-inferiority trial to compare trimethoprim/sulfamethoxazole plus rifampicin versus linezolid for the treatment of MRSA infection. *J Antimicrob Chemother*. Jan 2015;70(1):264-72. doi:10.1093/jac/dku352
448. Korvick JA, Peacock JE, Jr., Muder RR, Wheeler RR, Yu VL. Addition of rifampin to combination antibiotic therapy for Pseudomonas aeruginosa bacteremia: prospective trial using the Zelen protocol. *Antimicrob Agents Chemother*. Mar 1992;36(3):620-5.
449. Durante-Mangoni E, Signoriello G, Andini R, et al. Colistin and Rifampicin Compared With Colistin Alone for the Treatment of Serious Infections Due to Extensively Drug-Resistant Acinetobacter baumannii: A Multicenter, Randomized Clinical Trial. *Clin Infect Dis*. Aug 2013;57(3):349-58. doi:cit253 [pii]

10.1093/cid/cit253
450. Aydemir H, Akduman D, Piskin N, et al. Colistin vs. the combination of colistin and rifampicin for the treatment of carbapenem-resistant Acinetobacter baumannii ventilator-associated pneumonia. *Epidemiol Infect*. Sep 7 2012:1-9. doi:10.1017/S095026881200194X
451. Krsak M, Morrisette T, Miller M, et al. Advantages of Outpatient Treatment with Long-Acting Lipoglycopeptides for Serious Gram-Positive Infections: A Review. *Pharmacotherapy*. May 2020;40(5):469-478. doi:10.1002/phar.2389
452. Package Insert: orbactiv-prescribing-information.pdf. www.orbactiv.com/pdfs/orbactiv-prescribing-information.pdf
453. Dalbavancin package insert. https://www.accessdata.fda.gov/drugsatfda_docs/label/2016/021883s003lbl.pdf
454. Almangour TA, Perry GK, Alhifany AA. Dalbavancin versus standard of care for the treatment of osteomyelitis in adults: A retrospective matched cohort study. *Saudi Pharm J*. Apr 2020;28(4):460-464. doi:10.1016/j.jsps.2020.02.007

455. Streifel AC, Sikka MK, Bowen CD, Lewis JS, 2nd. Dalbavancin use in an academic medical centre and associated cost savings. *Int J Antimicrob Agents*. Nov 2019;54(5):652-654. doi:10.1016/j.ijantimicag.2019.08.007
456. Tobudic S, Forstner C, Burgmann H, et al. Real-world experience with dalbavancin therapy in gram-positive skin and soft tissue infection, bone and joint infection. *Infection*. Dec 2019;47(6):1013-1020. doi:10.1007/s15010-019-01354-x
457. Morrisette T, Miller MA, Montague BT, Barber GR, McQueen RB, Krsak M. On- and off-label utilization of dalbavancin and oritavancin for Gram-positive infections. *J Antimicrob Chemother*. Aug 1 2019;74(8):2405-2416. doi:10.1093/jac/dkz162
458. Bork JT, Heil EL, Berry S, et al. Dalbavancin Use in Vulnerable Patients Receiving Outpatient Parenteral Antibiotic Therapy for Invasive Gram-Positive Infections. *Infect Dis Ther*. Jun 2019;8(2):171-184. doi:10.1007/s40121-019-0247-0
459. Almangour TA, Perry GK, Terriff CM, Alhifany AA, Kaye KS. Dalbavancin for the management of gram-positive osteomyelitis: Effectiveness and potential utility. *Diagn Microbiol Infect Dis*. Mar 2019;93(3):213-218. doi:10.1016/j.diagmicrobio.2018.10.007
460. Bryson-Cahn C, Beieler AM, Chan JD, Harrington RD, Dhanireddy S. Dalbavancin as Secondary Therapy for Serious Staphylococcus aureus Infections in a Vulnerable Patient Population. *Open forum infectious diseases*. Feb 2019;6(2):ofz028. doi:10.1093/ofid/ofz028
461. Bouza E, Valerio M, Soriano A, et al. Dalbavancin in the treatment of different gram-positive infections: a real-life experience. *Int J Antimicrob Agents*. Apr 2018;51(4):571-577. doi:10.1016/j.ijantimicag.2017.11.008
462. Almangour TA, Fletcher V, Alessa M, Alhifany AA, Tabb D. Multiple Weekly Dalbavancin Dosing for the Treatment of Native Vertebral Osteomyelitis Caused by Methicillin-Resistant Staphylococcus Aureus: A Case Report. *Am J Case Rep*. Dec 9 2017;18:1315-1319. doi:10.12659/ajcr.905930
463. Wunsch S, Krause R, Valentin T, et al. Multicenter clinical experience of real life Dalbavancin use in gram-positive infections. *International journal of infectious diseases : IJID : official publication of the International Society for Infectious Diseases*. Apr 2019;81:210-214. doi:10.1016/j.ijid.2019.02.013
464. Morata L, Cobo J, Fernandez-Sampedro M, et al. Safety and Efficacy of Prolonged Use of Dalbavancin in Bone and Joint Infections. *Antimicrob Agents Chemother*. May 2019;63(5)doi:10.1128/AAC.02280-18
465. Cain AR, Bremmer DN, Carr DR, et al. Effectiveness of Dalbavancin Compared With Standard of Care for the Treatment of Osteomyelitis: A Real-world Analysis. *Open forum infectious diseases*. Feb 2022;9(2):ofab589. doi:10.1093/ofid/ofab589
466. Corey GR, Good S, Jiang H, et al. Single-dose oritavancin versus 7-10 days of vancomycin in the treatment of gram-positive acute bacterial skin and skin structure infections: the SOLO II noninferiority study. *Clin Infect Dis*. Jan 15 2015;60(2):254-62. doi:10.1093/cid/ciu778
467. Corey GR, Kabler H, Mehra P, et al. Single-dose oritavancin in the treatment of acute bacterial skin infections. *N Engl J Med*. Jun 5 2014;370(23):2180-90. doi:10.1056/NEJMoa1310422
468. Brownell LE, Adamsick ML, McCreary EK, et al. Clinical Outcomes and Economic Impact of Oritavancin for Gram-Positive Infections: A Single Academic Medical Center Health System Experience. *Drugs Real World Outcomes*. Jun 2020;7(Suppl 1):13-19. doi:10.1007/s40801-020-00192-w

© 2022 Spellberg B et al. *JAMA Network Open.*

469. Chastain DB, Davis A. Treatment of chronic osteomyelitis with multidose oritavancin: A case series and literature review. *Int J Antimicrob Agents*. Apr 2019;53(4):429-434. doi:10.1016/j.ijantimicag.2018.11.023
470. Van Hise NW, Chundi V, Didwania V, et al. Treatment of Acute Osteomyelitis with Once-Weekly Oritavancin: A Two-Year, Multicenter, Retrospective Study. *Drugs Real World Outcomes*. Jun 2020;7(Suppl 1):41-45. doi:10.1007/s40801-020-00195-7
471. Scoble PJ, Reilly J, Tillotson GS. Real-World Use of Oritavancin for the Treatment of Osteomyelitis. *Drugs Real World Outcomes*. Jun 2020;7(Suppl 1):46-54. doi:10.1007/s40801-020-00194-8
472. Nix DE, Cumbo TJ, Kuritzky P, DeVito JM, Schentag JJ. Oral ciprofloxacin in the treatment of serious soft tissue and bone infections. Efficacy, safety, and pharmacokinetics. *Am J Med*. Apr 27 1987;82(4A):146-53.
473. Lesse AJ, Freer C, Salata RA, Francis JB, Scheld WM. Oral ciprofloxacin therapy for gram-negative bacillary osteomyelitis. *Am J Med*. Apr 27 1987;82(4A):247-53.
474. Gilbert DN, Tice AD, Marsh PK, Craven PC, Preheim LC. Oral ciprofloxacin therapy for chronic contiguous osteomyelitis caused by aerobic gram-negative bacilli. *Am J Med*. Apr 27 1987;82(4A):254-8.
475. Hessen MT, Ingerman MJ, Kaufman DH, et al. Clinical efficacy of ciprofloxacin therapy for gram-negative bacillary osteomyelitis. *Am J Med*. Apr 27 1987;82(4A):262-5.
476. Greenberg RN, Kennedy DJ, Reilly PM, et al. Treatment of bone, joint, and soft-tissue infections with oral ciprofloxacin. *Antimicrob Agents Chemother*. Feb 1987;31(2):151-5.
477. Giamarellou H, Galanakis N. Use of intravenous ciprofloxacin in difficult-to-treat infections. *Am J Med*. Apr 27 1987;82(4A):346-51.
478. Scully BE, Neu HC. Treatment of serious infections with intravenous ciprofloxacin. *Am J Med*. Apr 27 1987;82(4A):369-75.
479. Therapy of acute and chronic gram-negative osteomyelitis with ciprofloxacin. Report from a Swedish Study Group. *J Antimicrob Chemother*. Aug 1988;22(2):221-8.
480. Dellamonica P, Bernard E, Etesse H, Garraffo R, Drugeon HB. Evaluation of pefloxacin, ofloxacin and ciprofloxacin in the treatment of thirty-nine cases of chronic osteomyelitis. *Eur J Clin Microbiol Infect Dis*. Dec 1989;8(12):1024-30.
481. Ketterl R, Beckurts T, Stubinger B, Claudi B. Use of ofloxacin in open fractures and in the treatment of post-traumatic osteomyelitis. *J Antimicrob Chemother*. Sep 1988;22 Suppl C:159-66.
482. Peterson LR, Lissack LM, Canter K, Fasching CE, Clabots C, Gerding DN. Therapy of lower extremity infections with ciprofloxacin in patients with diabetes mellitus, peripheral vascular disease, or both. *Am J Med*. Jun 1989;86(6 Pt 2):801-8.
483. Greenberg RN, Newman MT, Shariaty S, Pectol RW. Ciprofloxacin, lomefloxacin, or levofloxacin as treatment for chronic osteomyelitis. *Antimicrob Agents Chemother*. Jan 2000;44(1):164-6.
484. Drancourt M, Stein A, Argenson JN, Zannier A, Curvale G, Raoult D. Oral rifampin plus ofloxacin for treatment of Staphylococcus-infected orthopedic implants. *Antimicrob Agents Chemother*. Jun 1993;37(6):1214-8.
485. Barberan J, Aguilar L, Gimenez MJ, Carroquino G, Granizo JJ, Prieto J. Levofloxacin plus rifampicin conservative treatment of 25 early staphylococcal infections of osteosynthetic devices for rigid internal fixation. *Int J Antimicrob Agents*. Aug 2008;32(2):154-7. doi:S0924-8579(08)00135-0 [pii]

© 2022 Spellberg B et al. *JAMA Network Open.*

10.1016/j.ijantimicag.2008.03.003
486. Drancourt M, Stein A, Argenson JN, Roiron R, Groulier P, Raoult D. Oral treatment of Staphylococcus spp. infected orthopaedic implants with fusidic acid or ofloxacin in combination with rifampicin. *J Antimicrob Chemother*. Feb 1997;39(2):235-40.
487. Birmingham MC, Rayner CR, Meagher AK, Flavin SM, Batts DH, Schentag JJ. Linezolid for the treatment of multidrug-resistant, gram-positive infections: experience from a compassionate-use program. *Clin Infect Dis*. Jan 15 2003;36(2):159-68.
488. Joel J, Graham SM, Peckham-Cooper A, Korres N, Tsouchnica H, Tsiridis E. Clinical results of linezolid in arthroplasty and trauma MRSA related infections. *World J Orthop*. Apr 18 2014;5(2):151-7. doi:10.5312/wjo.v5.i2.151
489. Pontifex AH, McNaught DR. The treatment of chronic osteomyelitis with clindamycin. *Can Med Assoc J*. Jul 21 1973;109(2):105-7.
490. Bonnaire A, Vernet-Garnier V, Lebrun D, et al. Clindamycin combination treatment for the treatment of bone and joint infections caused by clindamycin-susceptible, erythromycin-resistant Staphylococcus spp. *Diagn Microbiol Infect Dis*. Jan 2021;99(1):115225. doi:10.1016/j.diagmicrobio.2020.115225
491. Courjon J, Demonchy E, Cua E, Bernard E, Roger PM. Efficacy and safety of clindamycin-based treatment for bone and joint infections: a cohort study. *Eur J Clin Microbiol Infect Dis*. Dec 2017;36(12):2513-2518. doi:10.1007/s10096-017-3094-5
492. Craven JL, Pugsley DJ, Blowers R. Trimethoprim-sulphamethoxazole in acute osteomyelitis due to penicillin-resistant staphylococci in Uganda. *British medical journal*. Jul 25 1970;3(716):201-3.
493. Saengnipanthkul S, Pongvivat T, Mahaisavariya B, Laupattarakasem W. Co-trimoxazole in the treatment of chronic osteomyelitis. *Journal of the Medical Association of Thailand = Chotmaihet thangphaet*. Apr 1988;71(4):186-91.
494. Sanchez C, Matamala A, Salavert M, et al. Cotrimoxazole plus rifampicin in the treatment of staphylococcal osteoarticular infection. *Enferm Infecc Microbiol Clin*. Jan 1997;15(1):10-3. Cotrimoxazol mas rifampicina en el tratamiento de la infeccion osteoarticular estafilococica.
495. Javaloyas de Morlius M, Monreal Portella M. Oral antibiotic therapy in the adult bacterial osteomyelitis: results after two years of follow-up. *Med Clin (Barc)*. Oct 23 1999;113(13):488-9. Tratamiento antibiotico por via oral de la osteomielitis bacteriana del adulto: resultados tras dos anos de seguimiento.
496. Stein A, Bataille JF, Drancourt M, et al. Ambulatory treatment of multidrug-resistant Staphylococcus-infected orthopedic implants with high-dose oral co-trimoxazole (trimethoprim-sulfamethoxazole). *Antimicrob Agents Chemother*. Dec 1998;42(12):3086-91.
497. de Barros JW, Calapodopulos CJ, Oliveira DJ, Miki Junior P. The treatment of chronic osteomyelitis. *Rev Soc Bras Med Trop*. Oct-Dec 1992;25(4):235-9. Tratamento das osteomielites cronicas.
498. Deconinck L, Dinh A, Nich C, et al. Efficacy of cotrimoxazole (Sulfamethoxazole-Trimethoprim) as a salvage therapy for the treatment of bone and joint infections (BJIs). *PLoS One*. 2019;14(10):e0224106. doi:10.1371/journal.pone.0224106
499. Nguyen S, Pasquet A, Legout L, et al. Efficacy and tolerance of rifampicin-linezolid compared with rifampicin-cotrimoxazole combinations in prolonged oral therapy for bone and joint infections. *Clin Microbiol Infect*. Dec 2009;15(12):1163-9. doi:CLM2761 [pii]

© 2022 Spellberg B et al. *JAMA Network Open.*

10.1111/j.1469-0691.2009.02761.x
500. Meissner A, Haag R, Rahmanzadeh R. Adjuvant fosfomycin medication in chronic osteomyelitis. *Infection*. May-Jun 1989;17(3):146-51.
501. Fernandez-Valencia JE, Saban T, Canedo T, Olay T. Fosfomycin in Osteomyelitis. *Chemotherapy*. 1976;22(2):121-34.
502. Hernandez Casado V. Fosfomycin in a traumatological department. *Chemotherapy*. 1977;23 Suppl 1:403-10. doi:10.1159/000222082
503. Wang JL, Tang HJ, Hsieh PH, et al. Fusidic acid for the treatment of bone and joint infections caused by meticillin-resistant Staphylococcus aureus. *Int J Antimicrob Agents*. Aug 2012;40(2):103-7. doi:10.1016/j.ijantimicag.2012.03.010
504. Coombs RR, Menday AP. Fusidic acid in orthopaedic infections due to coagulase-negative staphylococci. *Current medical research and opinion*. 1985;9(9):587-90. doi:10.1185/03007998509109638
505. Venkatesan P, Lawn S, Macfarlane RM, Fletcher EM, Finch RG, Jeffcoate WJ. Conservative management of osteomyelitis in the feet of diabetic patients. *Diabet Med*. Jun 1997;14(6):487-90. doi:10.1002/(SICI)1096-9136(199706)14:6<487::AID-DIA373>3.0.CO;2-Q
506. Pittet D, Wyssa B, Herter-Clavel C, Kursteiner K, Vaucher J, Lew PD. Outcome of diabetic foot infections treated conservatively: a retrospective cohort study with long-term follow-up. *Arch Intern Med*. Apr 26 1999;159(8):851-6.
507. Senneville E, Yazdanpanah Y, Cazaubiel M, et al. Rifampicin-ofloxacin oral regimen for the treatment of mild to moderate diabetic foot osteomyelitis. *J Antimicrob Chemother*. Dec 2001;48(6):927-30. doi:10.1093/jac/48.6.927
508. Embil JM, Rose G, Trepman E, et al. Oral antimicrobial therapy for diabetic foot osteomyelitis. *Foot Ankle Int*. Oct 2006;27(10):771-9. doi:10.1177/107110070602701003
509. Gariani K, Lebowitz D, Kressmann B, et al. Oral amoxicillin-clavulanate for treating diabetic foot infections. *Diabetes Obes Metab*. Jun 2019;21(6):1483-1486. doi:10.1111/dom.13651
510. Senneville E, Poissy J, Legout L, et al. Safety of prolonged high-dose levofloxacin therapy for bone infections. *J Chemother*. Dec 2007;19(6):688-93. doi:10.1179/joc.2007.19.6.688
511. Bouazza N, Pestre V, Jullien V, et al. Population pharmacokinetics of clindamycin orally and intravenously administered in patients with osteomyelitis. *Br J Clin Pharmacol*. Dec 2012;74(6):971-7. doi:10.1111/j.1365-2125.2012.04292.x
512. Waldvogel FA, Medoff G, Swartz MN. Osteomyelitis: a review of clinical features, therapeutic considerations and unusual aspects. *N Engl J Med*. Jan 22 1970;282(4):198-206.
513. Suzuki H, Mosher HJ, Heintz BH, Livorsi DJ. Implementation of a consensus protocol for antibiotic use for bone and joint infection to reduce unnecessary outpatient parenteral antimicrobial therapy: A quality improvement initiative. *Antimicrob Stewardship Healthcare Epidemiol*. 2022;2(1):E6. doi:10.1017/ash.2021.250
514. Messina AF, Namtu K, Guild M, Dumois JA, Berman DM. Trimethoprim-sulfamethoxazole therapy for children with acute osteomyelitis. *The Pediatric infectious disease journal*. Dec 2011;30(12):1019-21. doi:10.1097/INF.0b013e31822db658
515. Theil C, Schmidt-Braekling T, Gosheger G, et al. Clinical use of linezolid in periprosthetic joint infections - a systematic review. *J Bone Jt Infect*. 2020;6(1):7-16. doi:10.5194/jbji-6-7-2020

© 2022 Spellberg B et al. *JAMA Network Open.*

516. Peltola H, Paakkonen M, Kallio P, Kallio MJ, Group O-SS. Clindamycin vs. first-generation cephalosporins for acute osteoarticular infections of childhood--a prospective quasi-randomized controlled trial. *Clin Microbiol Infect*. Jun 2012;18(6):582-9. doi:10.1111/j.1469-0691.2011.03643.x
517. FDA warns about increased risk of ruptures or tears in the aorta blood vessel with fluoroquinolone antibiotics in certain patients: FDA Drug Safety Communication. US Food and Drug Administration; 2019. Accessed March 19, 2021. https://www.fda.gov/drugs/drug-safety-and-availability/fda-warns-about-increased-risk-ruptures-or-tears-aorta-blood-vessel-fluoroquinolone-antibiotics
518. Kuula LSM, Viljemaa KM, Backman JT, Blom M. Fluoroquinolone-related adverse events resulting in health service use and costs: A systematic review. *PLoS One*. 2019;14(4):e0216029. doi:10.1371/journal.pone.0216029
519. Ho JMW, Juurlink DN. Considerations when prescribing trimethoprim–sulfamethoxazole. *CMAJ*. 2011;183:1851-8.
520. Pourbagher A, Pourbagher MA, Savas L, et al. Epidemiologic, clinical, and imaging findings in brucellosis patients with osteoarticular involvement. *AJR Am J Roentgenol*. Oct 2006;187(4):873-80. doi:10.2214/AJR.05.1088
521. Colmenero JD, Ruiz-Mesa JD, Plata A, et al. Clinical findings, therapeutic approach, and outcome of brucellar vertebral osteomyelitis. *Clin Infect Dis*. Feb 1 2008;46(3):426-33. doi:10.1086/525266
522. Ghanem-Zoubi N, Karram T, Kagna O, Merhav G, Keidar Z, Paul M. Q fever vertebral osteomyelitis among adults: a case series and literature review. *Infect Dis (Lond)*. Apr 2021;53(4):231-240. doi:10.1080/23744235.2020.1871508
523. Fralick M, Macdonald EM, Gomes T, et al. Co-trimoxazole and sudden death in patients receiving inhibitors of renin-angiotensin system: population based study. *Bmj*. Oct 30 2014;349:g6196. doi:10.1136/bmj.g6196
524. Ruslami R, Nijland HM, Alisjahbana B, Parwati I, van Crevel R, Aarnoutse RE. Pharmacokinetics and tolerability of a higher rifampin dose versus the standard dose in pulmonary tuberculosis patients. *Antimicrob Agents Chemother*. Jul 2007;51(7):2546-51. doi:10.1128/AAC.01550-06
525. Chirehwa MT, Rustomjee R, Mthiyane T, et al. Model-Based Evaluation of Higher Doses of Rifampin Using a Semimechanistic Model Incorporating Autoinduction and Saturation of Hepatic Extraction. *Antimicrob Agents Chemother*. Jan 2016;60(1):487-94. doi:10.1128/AAC.01830-15
526. Stott KE, Pertinez H, Sturkenboom MGG, et al. Pharmacokinetics of rifampicin in adult TB patients and healthy volunteers: a systematic review and meta-analysis. *J Antimicrob Chemother*. Sep 1 2018;73(9):2305-2313. doi:10.1093/jac/dky152
527. Spellberg B, Chambers HF, Musher DM, Walsh TL, Bayer AS. Evaluation of a Paradigm Shift From Intravenous Antibiotics to Oral Step-Down Therapy for the Treatment of Infective Endocarditis: A Narrative Review. *JAMA internal medicine*. Mar 30 2020;doi:10.1001/jamainternmed.2020.0555
528. Carragee EJ, Kim D, van der Vlugt T, Vittum D. The clinical use of erythrocyte sedimentation rate in pyogenic vertebral osteomyelitis. *Spine (Phila Pa 1976)*. Sep 15 1997;22(18):2089-93. doi:10.1097/00007632-199709150-00005

© 2022 Spellberg B et al. *JAMA Network Open.*

529. Kowalski TJ, Berbari EF, Huddleston PM, Steckelberg JM, Osmon DR. Do follow-up imaging examinations provide useful prognostic information in patients with spine infection? *Clin Infect Dis*. Jul 15 2006;43(2):172-9. doi:10.1086/505118
530. Nanni C, Boriani L, Salvadori C, et al. FDG PET/CT is useful for the interim evaluation of response to therapy in patients affected by haematogenous spondylodiscitis. *Eur J Nucl Med Mol Imaging*. Oct 2012;39(10):1538-44. doi:10.1007/s00259-012-2179-8
531. Park KH, Cho OH, Lee JH, et al. Optimal Duration of Antibiotic Therapy in Patients With Hematogenous Vertebral Osteomyelitis at Low Risk and High Risk of Recurrence. *Clin Infect Dis*. May 15 2016;62(10):1262-1269. doi:10.1093/cid/ciw098
532. Yoon SH, Chung SK, Kim KJ, Kim HJ, Jin YJ, Kim HB. Pyogenic vertebral osteomyelitis: identification of microorganism and laboratory markers used to predict clinical outcome. *Eur Spine J*. Apr 2010;19(4):575-82. doi:10.1007/s00586-009-1216-1
533. Babouee Flury B, Elzi L, Kolbe M, et al. Is switching to an oral antibiotic regimen safe after 2 weeks of intravenous treatment for primary bacterial vertebral osteomyelitis? *BMC Infect Dis*. Apr 27 2014;14:226. doi:10.1186/1471-2334-14-226
534. Khan MH, Smith PN, Rao N, Donaldson WF. Serum C-reactive protein levels correlate with clinical response in patients treated with antibiotics for wound infections after spinal surgery. *Spine J*. May-Jun 2006;6(3):311-5. doi:10.1016/j.spinee.2005.07.006
535. Michail M, Jude E, Liaskos C, et al. The performance of serum inflammatory markers for the diagnosis and follow-up of patients with osteomyelitis. *Int J Low Extrem Wounds*. Jun 2013;12(2):94-9. doi:10.1177/1534734613486152
536. Tardaguila-Garcia A, Garcia Alvarez Y, Garcia-Morales E, Alvaro-Afonso FJ, Sanz-Corbalan I, Lazaro-Martinez JL. Utility of Blood Parameters to Detect Complications during Long-Term Follow-Up in Patients with Diabetic Foot Osteomyelitis. *J Clin Med*. Nov 22 2020;9(11)doi:10.3390/jcm9113768
537. van Asten SA, Jupiter DC, Mithani M, La Fontaine J, Davis KE, Lavery LA. Erythrocyte sedimentation rate and C-reactive protein to monitor treatment outcomes in diabetic foot osteomyelitis. *International wound journal*. Feb 2017;14(1):142-148. doi:10.1111/iwj.12574
538. Lin Z, Vasudevan A, Tambyah PA. Use of erythrocyte sedimentation rate and C-reactive protein to predict osteomyelitis recurrence. *J Orthop Surg (Hong Kong)*. Apr 2016;24(1):77-83. doi:10.1177/230949901602400118
539. Faizal B, Surendran B, Kumar M. Comparative study of reliability of inflammatory markers over 18-FDG-PET CT scan in monitoring skull base osteomyelitis. *Braz J Otorhinolaryngol*. Oct 31 2020;doi:10.1016/j.bjorl.2020.09.012
540. Ghani R, Hutt J, Mitchell P, Granger L, Sandiford NA. Serial C-reactive Protein Monitoring in Prosthetic Joint Infection: A Powerful Predictor or Potentially Pointless? *Cureus*. Feb 12 2020;12(2):e6967. doi:10.7759/cureus.6967
541. Ghanem E, Azzam K, Seeley M, Joshi A, Parvizi J. Staged revision for knee arthroplasty infection: what is the role of serologic tests before reimplantation? *Clin Orthop Relat Res*. Jul 2009;467(7):1699-705. doi:10.1007/s11999-009-0742-9
542. Shukla SK, Ward JP, Jacofsky MC, Sporer SM, Paprosky WG, Della Valle CJ. Perioperative testing for persistent sepsis following resection arthroplasty of the hip for periprosthetic infection. *J Arthroplasty*. Sep 2010;25(6 Suppl):87-91. doi:10.1016/j.arth.2010.05.006

© 2022 Spellberg B et al. *JAMA Network Open.*

543. Maier SP, Klemt C, Tirumala V, Oganesyan R, van den Kieboom J, Kwon YM. Elevated ESR/CRP Ratio Is Associated With Reinfection After Debridement, Antibiotics, and Implant Retention in Chronic Periprosthetic Joint Infections. *J Arthroplasty*. Nov 2020;35(11):3254-3260. doi:10.1016/j.arth.2020.06.007
544. Bejon P, Byren I, Atkins BL, et al. Serial measurement of the C-reactive protein is a poor predictor of treatment outcome in prosthetic joint infection. *J Antimicrob Chemother*. Jul 2011;66(7):1590-3. doi:10.1093/jac/dkr182
545. Courtney PM, Flynn JM, Jaramillo D, Horn BD, Calabro K, Spiegel DA. Clinical indications for repeat MRI in children with acute hematogenous osteomyelitis. *J Pediatr Orthop*. Dec 2010;30(8):883-7. doi:10.1097/BPO.0b013e3181fcbae5
546. Zarrouk V, Feydy A, Salles F, et al. Imaging does not predict the clinical outcome of bacterial vertebral osteomyelitis. *Rheumatology (Oxford)*. Feb 2007;46(2):292-5. doi:10.1093/rheumatology/kel228
547. De Korvin B, Provensol T, Le Dantec P, et al. [Aspects and value of MRI in the diagnosis and follow-up of common microbes infectious spondylodiscitis. Apropos of 25 clinically and biologically suspected patients]. *J Radiol*. May 1994;75(5):267-77. Aspects et interet de l'IRM dans le diagnostic et le suivi des spondylodiscites infectieuses a germes banals. A propos de 25 patients suspects cliniquement et biologiquement.
548. Riccio SA, Chu AK, Rabin HR, Kloiber R. Fluorodeoxyglucose Positron Emission Tomography/Computed Tomography Interpretation Criteria for Assessment of Antibiotic Treatment Response in Pyogenic Spine Infection. *Can Assoc Radiol J*. May 2015;66(2):145-52. doi:10.1016/j.carj.2014.08.004
549. Niccoli Asabella A, Iuele F, Simone F, et al. Role of (18)F-FDG PET/CT in the evaluation of response to antibiotic therapy in patients affected by infectious spondylodiscitis. *Hell J Nucl Med*. Sep-Dec 2015;18 Suppl 1:17-22.
550. Scoles PV, Hilty MD, Sfakianakis GN. Bone scan patterns in acute osteomyelitis. *Clin Orthop Relat Res*. Nov-Dec 1980;(153):210-7.
551. Alazraki N, Fierer J, Resnick D. Chronic osteomyelitis: monitoring by 99mTc phosphate and 67Ga citrate imaging. *AJR Am J Roentgenol*. Oct 1985;145(4):767-71. doi:10.2214/ajr.145.4.767
552. Kattapuram SV, Phillips WC, Boyd R. CT in pyogenic osteomyelitis of the spine. *AJR Am J Roentgenol*. Jun 1983;140(6):1199-201. doi:10.2214/ajr.140.6.1199
553. Roblot F, Besnier JM, Juhel L, et al. Optimal duration of antibiotic therapy in vertebral osteomyelitis. *Semin Arthritis Rheum*. Apr 2007;36(5):269-77. doi:10.1016/j.semarthrit.2006.09.004
554. Rod-Fleury T, Dunkel N, Assal M, et al. Duration of post-surgical antibiotic therapy for adult chronic osteomyelitis: a single-centre experience. *Int Orthop*. Nov 2011;35(11):1725-31. doi:10.1007/s00264-011-1221-y
555. Shcherbin FG, Makhson NE, Petrakov AA. [Antibacterial chemotherapy in anaerobic osteomyelitis caused by gunshot wounds]. *Antibiot Khimioter*. May 1990;35(5):40-1. Antibakterial'naia khimioterapiia anaerobnykh ognestrel'nykh osteomielitov.
556. Gariani K, Lebowitz D, von Dach E, Kressmann B, Lipsky BA, Uckay I. Remission in diabetic foot infections: Duration of antibiotic therapy and other possible associated factors. *Diabetes Obes Metab*. Feb 2019;21(2):244-251. doi:10.1111/dom.13507

557. Barshes NR, Mindru C, Ashong C, Rodriguez-Barradas M, Trautner BW. Treatment Failure and Leg Amputation Among Patients With Foot Osteomyelitis. *Int J Low Extrem Wounds*. Dec 2016;15(4):303-312. doi:10.1177/1534734616661058
558. Julien G, Francois M, Vernet-Garnier V, et al. Short duration of post-amputation antibiotic therapy in diabetic foot patients with total resection of osteomyelitis. *Med Mal Infect*. Aug 2020;50(5):433-435. doi:10.1016/j.medmal.2020.04.015
559. Kowalski TJ, Matsuda M, Sorenson MD, Gundrum JD, Agger WA. The effect of residual osteomyelitis at the resection margin in patients with surgically treated diabetic foot infection. *J Foot Ankle Surg*. Mar-Apr 2011;50(2):171-5. doi:S1067-2516(10)00508-9 [pii]

10.1053/j.jfas.2010.12.009
560. Rossel A, Lebowitz D, Gariani K, et al. Stopping antibiotics after surgical amputation in diabetic foot and ankle infections-A daily practice cohort. *Endocrinol Diabetes Metab*. Apr 2019;2(2):e00059. doi:10.1002/edm2.59
561. Pansu N, Hamoui M, Manna F, et al. Implant retention and high rate of treatment failure in hematogenous acute knee and hip prosthetic joint infections. *Med Mal Infect*. Nov 2020;50(8):702-708. doi:10.1016/j.medmal.2019.11.005
562. Laffer RR, Graber P, Ochsner PE, Zimmerli W. Outcome of prosthetic knee-associated infection: evaluation of 40 consecutive episodes at a single centre. *Clin Microbiol Infect*. May 2006;12(5):433-9. doi:10.1111/j.1469-0691.2006.01378.x
563. Marculescu CE, Berbari EF, Hanssen AD, et al. Outcome of prosthetic joint infections treated with debridement and retention of components. *Clin Infect Dis*. Feb 15 2006;42(4):471-8. doi:10.1086/499234
564. Brandt CM, Sistrunk WW, Duffy MC, et al. Staphylococcus aureus prosthetic joint infection treated with debridement and prosthesis retention. *Clin Infect Dis*. May 1997;24(5):914-9. doi:10.1093/clinids/24.5.914
565. Chaussade H, Uckay I, Vuagnat A, et al. Antibiotic therapy duration for prosthetic joint infections treated by Debridement and Implant Retention (DAIR): Similar long-term remission for 6 weeks as compared to 12 weeks. *International journal of infectious diseases : IJID : official publication of the International Society for Infectious Diseases*. Oct 2017;63:37-42. doi:10.1016/j.ijid.2017.08.002
566. Rodriguez D, Pigrau C, Euba G, et al. Acute haematogenous prosthetic joint infection: prospective evaluation of medical and surgical management. *Clin Microbiol Infect*. Dec 2010;16(12):1789-95. doi:10.1111/j.1469-0691.2010.03157.x
567. Byren I, Bejon P, Atkins BL, et al. One hundred and twelve infected arthroplasties treated with 'DAIR' (debridement, antibiotics and implant retention): antibiotic duration and outcome. *J Antimicrob Chemother*. Jun 2009;63(6):1264-71. doi:10.1093/jac/dkp107
568. Silva M, Tharani R, Schmalzried TP. Results of direct exchange or debridement of the infected total knee arthroplasty. *Clin Orthop Relat Res*. Nov 2002;(404):125-31. doi:10.1097/00003086-200211000-00022
569. Escudero-Sanchez R, Senneville E, Digumber M, et al. Suppressive antibiotic therapy in prosthetic joint infections: a multicentre cohort study. *Clin Microbiol Infect*. Apr 2020;26(4):499-505. doi:10.1016/j.cmi.2019.09.007
570. Letouvet B, Arvieux C, Leroy H, et al. Predictors of failure for prosthetic joint infections treated with debridement. *Med Mal Infect*. Feb 2016;46(1):39-43. doi:10.1016/j.medmal.2015.11.007

© 2022 Spellberg B et al. *JAMA Network Open.*

571. Segreti J, Nelson JA, Trenholme GM. Prolonged suppressive antibiotic therapy for infected orthopedic prostheses. *Clin Infect Dis*. Oct 1998;27(4):711-3. doi:10.1086/514951
572. Siqueira MB, Saleh A, Klika AK, et al. Chronic Suppression of Periprosthetic Joint Infections with Oral Antibiotics Increases Infection-Free Survivorship. *J Bone Joint Surg Am*. Aug 5 2015;97(15):1220-32. doi:10.2106/JBJS.N.00999
573. Shah NB, Hersh BL, Kreger A, et al. Benefits and Adverse Events Associated With Extended Antibiotic Use in Total Knee Arthroplasty Periprosthetic Joint Infection. *Clin Infect Dis*. Feb 3 2020;70(4):559-565. doi:10.1093/cid/ciz261
574. Keller SC, Cosgrove SE, Higgins Y, Piggott DA, Osgood G, Auwaerter PG. Role of Suppressive Oral Antibiotics in Orthopedic Hardware Infections for Those Not Undergoing Two-Stage Replacement Surgery. *Open forum infectious diseases*. Oct 2016;3(4):ofw176. doi:10.1093/ofid/ofw176
575. Malahias MA, Gu A, Harris EC, et al. The Role of Long-Term Antibiotic Suppression in the Management of Peri-Prosthetic Joint Infections Treated With Debridement, Antibiotics, and Implant Retention: A Systematic Review. *J Arthroplasty*. Apr 2020;35(4):1154-1160. doi:10.1016/j.arth.2019.11.026
576. Cobo J, Escudero-Sanchez R. Suppressive Antibiotic Treatment in Prosthetic Joint Infections: A Perspective. *Antibiotics (Basel)*. 2021;10:743-750.
577. Yen HT, Hsieh RW, Huang CY, et al. Short-course versus long-course antibiotics in prosthetic joint infections: a systematic review and meta-analysis of one randomized controlled trial plus nine observational studies. *J Antimicrob Chemother*. Sep 1 2019;74(9):2507-2516. doi:10.1093/jac/dkz166

© 2022 Spellberg B et al. *JAMA Network Open.*
