# Supplementary material for: Use of Novel Strategies to Develop Guidelines for Management of Pyogenic Osteomyelitis in Adults: A WikiGuidelines Group Consensus Statement
Source: JAMA Netw Open. 2022 May 10;5(5):e2211321. doi: 10.1001/jamanetworkopen.2022.11321 (PMC9092201; doi:10.1001/jamanetworkopen.2022.11321)
Supplement: Supplement 3. — Nonauthor Collaborators [file jamanetwopen-e2211321-s003.pdf]

Supplemental Online Content: Nonauthor Collaborators

*Indicates required information. Only first name, last name, and suffix will appear in PubMed.

*Group Name(s): WikiGuidelines Group

***First Name and Middle Initial(s)**	***Last Name**	***Suffix (eg, Jr, III)**	Academic Degrees	Institution	Location (city, state/province, country)	Role or Contribution, eg, chair, principal investigator	Group (if more than 1 Group listed in the byline) and/or Subgroup (eg, Steering Committee)
Rachel	Baden		MD	LAC+USC Medical Center	Los Angeles, CA, USA	WikiGuidelines Member, Charter Development	
Samuel	Bedard-Dallare		MD	Universite de Sherbrooke	Sherbrooke, Quebec, Cana	WikiGuidelines Member, Charter Development	
Claudia	Beltran		MD	Universidad Autonoma de Colombia	Bogota, Colombia	WikiGuidelines Member, Charter Development	
Michelle	Blythe		MD	Tulane University School of Medicine	New Orleans, LA, USA	WikiGuidelines Member, Charter Development	
Eric	Brass		MD PhD	University of California, Los Angeles	Los Angeles, CA, USA	WikiGuidelines Member, Charter Development	
Sharon	Chi		DO	Tripler Army Medical Center	Honolulu, HI, USA	WikiGuidelines Member, Charter Development	
Chase	Coffey	Jr	MD	LAC+USC Medical Center	Los Angeles, CA, USA	WikiGuidelines Member, Charter Development	
Mallory	Cowart		PharmD	Orlando Health	Orlando, FL, USA	WikiGuidelines Member, Charter Development	
Alejandro	Diaz		MD	Universidad, CES	Medellin, Colombia	WikiGuidelines Member, Charter Development	
John	Dwyer		DO	Tulane University School of Medicine	New Orleans, LA, USA	WikiGuidelines Member, Charter Development	
Alejandro	Jordan Villegas		MD	Orlando Health Arnold Palmer Hospit	Orlando, FL, USA	WikiGuidelines Member, Charter Development	
Ezza	Khan		MD	Hunterdon Healthcare	Flemington, NJ, USA	WikiGuidelines Member, Charter Development	
Jose	Martinez		MD	Clínica Universitaria de Podología	Madrin, Spain	WikiGuidelines Member, Charter Development	
Arun	Mattappallil		PharmD	University Hospital	Newark, NJ, USA	WikiGuidelines Member, Charter Development	
Nessa	Meshkaty		MD	Oceanview Medical Specialists	Ventura, CA, USA	WikiGuidelines Member, Charter Development	
Arun	Patel		MD JD ME	Los Angeles County Department of H	Los Angeles, CA, USA	WikiGuidelines Member, Charter Development	
Matthew	Pullen		MD	University of Minnesota	Minneapolis, MN, USA	WikiGuidelines Member, Charter Development	
Sujatha	Rajan		MD	Cohen Children's Northwell Health Ph	New Hyde Park, NY, USA	WikiGuidelines Member, Charter Development	
Lynora	Saxinger		MD	University of Alberta School of Medic	Edmonton, Alberta, Canada	WikiGuidelines Member, Charter Development	
Rhagavendra	Tirupathi		MD	Keystone Health	Chambersburg, PA, USA	WikiGuidelines Member, Charter Development	
Julie	Trivedi		MD	UT Southwestern School of Medicine	Dallas, TX, USA	WikiGuidelines Member, Charter Development	
Gabriel	Vilchez-Molina		MD	Pikeville Medical Center	Pikeville, KY, USA	WikiGuidelines Member, Charter Development	
Dominique	Werge		PharmD	LAC+USC Medical Center	Los Angeles, CA, USA	WikiGuidelines Member, Charter Development	
